# Preparation of multiblock copolymers *via* step-wise addition of l-lactide and trimethylene carbonate†
†Electronic supplementary information (ESI) available: NMR spectra, SEC traces, DSC, DMA, X-ray, and DFT calculation data. CCDC 1580591. For ESI and crystallographic data in CIF or other electronic format see DOI: 10.1039/c7sc04507g


**DOI:** 10.1039/c7sc04507g

**Published:** 2018-01-11

**Authors:** Mark Abubekerov, Junnian Wei, Kevin R. Swartz, Zhixin Xie, Qibing Pei, Paula L. Diaconescu

**Affiliations:** a Department of Chemistry and Biochemistry , University of California , Los Angeles , CA 90095 , USA . Email: pld@chem.ucla.edu; b Department of Materials Science and Engineering , University of California , Los Angeles , CA 90095 , USA

## Abstract

Poly(l-lactide) (PLA) is a bioderived and biodegradable polymer that has limited applications due to its hard and brittle nature. Incorporation of 1,3-trimethylene carbonate into PLA, in a block copolymer fashion, improves the mechanical properties, while retaining the biodegradability of the polymer, and broadens its range of applications. However, the preparation of 1,3-trimethylene carbonate (TMC)/l-lactide (LA) copolymers beyond diblock and triblock structures has not been reported, with explanations focusing mostly on thermodynamic reasons that impede the copolymerization of TMC after lactide. We discuss the preparation of multiblock copolymers *via* the ring opening polymerization (ROP) of LA and TMC, in a step-wise addition, by a ferrocene-chelating heteroscorpionate zinc complex, {[fc(PPh_2_)(BH[(3,5-Me)_2_pz]_2_)]Zn(μ-OCH_2_Ph)}_2_ ([(fc^P,B^)Zn(μ-OCH_2_Ph)]_2_, fc = 1,1′-ferrocenediyl, pz = pyrazole). The synthesis of up to pentablock copolymers, from various combinations of LA and TMC, was accomplished and the physical, thermal, and mechanical properties of the resulting copolymers evaluated.

## Abstract 2

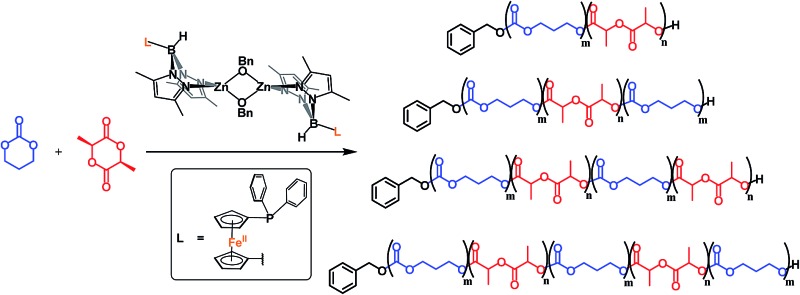
The synthesis of up to pentablock copolymers from various combinations of l-lactide and trimethylene carbonate was accomplished using a dinuclear zinc complex, and the physical, thermal, and mechanical properties of the resulting copolymers evaluated.

## Introduction

Growing concerns over the environmental damage caused by petroleum-based plastic waste^1^ and the associated health effects due to petroleum processing^2^ necessitate a shift to environmentally benign commodity plastics.^3^–^6^ As a result, biodegradable plastics obtained from bio-renewable sources, in particular poly(l-lactide) (PLA),^7^,^8^ have received much attention in the past decades.^3^,^9^–^11^ Currently, applications of PLA vary widely from specialty plastics in biomedical devices^12^–^15^ to commodity plastics in food packaging.^14^–^16^ The mechanical properties of PLA resemble those of polystyrene;^11^ it is a hard material with good tensile strength and high modulus.^10^ Unfortunately, due to its low toughness, its overall applications are limited.^17^ A potential way of enhancing the toughness of PLA is through copolymerization with 1,3-trimethylene carbonate (TMC), which gives a soft and amorphous homopolymer.^18^ In this regard, Guerin *et al.*^19^ and Leng *et al.*^20^ performed detailed studies on the influence of block TMC incorporation into PLA. These reports concluded that a *ca.* 20% weight of TMC into TMC/LA block copolymers is optimal. The resulting thermoplastic elastomers,^19^,^20^ of PLA-*b*-PTMC and PLA-*b*-PTMC-*b*-PLA compositions,^19^ were shown to display both moderate elongation at break and moderate Young's modulus values. However, these copolymers are predominantly prepared *via* an initial TMC polymerization followed by the sequential addition of LA, in the presence of various organic or metal-based catalysts;^19^–^29^ a few examples, capable of polymerizing TMC faster than LA when mixtures of the two monomers are used^30^ or of polymerizing TMC after the polymerization of LA, were reported.^31^ As a result, only a small number of LA/TMC block combinations have been investigated and the influence of more complicated block structures on the mechanical properties of these copolymers is rather underexplored.^19^ In the course of studying the redox switchable reactivity^32^–^46^ of the ferrocene-chelating heteroscorpionate zinc complex {[fc(PPh_2_)(BH[(3,5-Me)_2_pz]_2_)]Zn(μ-OCH_2_Ph)}_2_ ([(fc^P,B^)Zn(μ-OCH_2_Ph)]_2_)^37^ toward various monomers, we discovered that it can perform the ring opening polymerization (ROP) of LA and TMC regardless of the addition order. Based on our interest in the ROP of cyclic esters and carbonates, we set out to prepare multiblock copolymers of l-lactide and 1,3-trimethylene carbonate to examine their physical, thermal, and mechanical properties, and we discuss our results herein.

## Results and discussion

### Polymerization studies

Because of the unique behavior of [(fc^P,B^)Zn(μ-OCH_2_Ph)] toward the ROP of LA and TMC, *i.e.*, its ability to polymerize TMC after LA, we began by studying the solid state molecular structure and the solution behavior of the metal complex. The isolation of [(fc^P,B^)Zn(μ-OCH_2_Ph)]_2_ as yellow crystals in a 68.5% yield (eqn (1)) was achieved *via* the addition of (fc^P,B^)ZnCl·(C_7_H_8_)^37^ to *in situ* generated KOCH_2_Ph in THF.

**Fig. 1:**
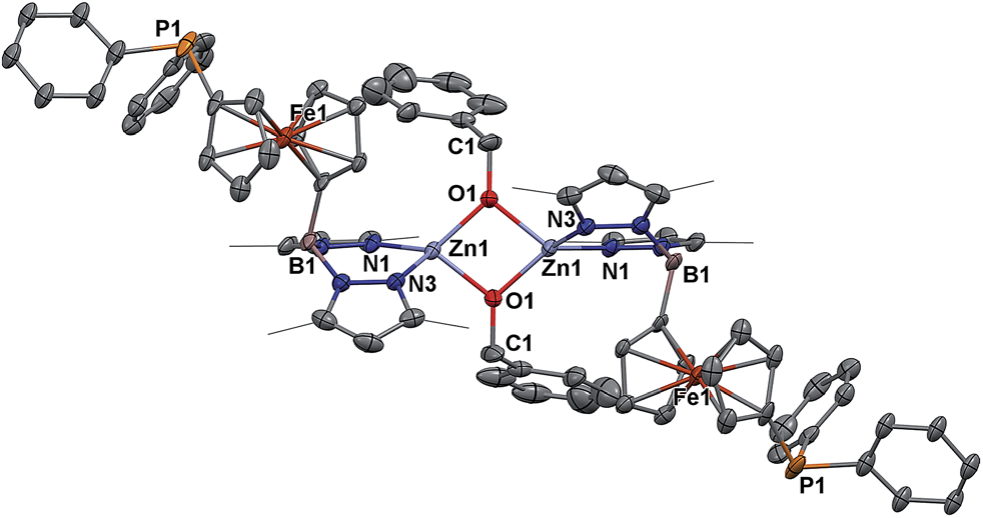
Molecular structure drawing of [(fc^P,B^)Zn(μ-OCH_2_Ph)]_2_ with thermal ellipsoids at 50% probability; hydrogen atoms and disordered counterparts are omitted for clarity.

The solid state molecular structure of [(fc^P,B^)Zn(μ-OCH_2_Ph)]_2_ was determined using single-crystal X-ray diffraction (Fig. 1). The coordination environment around each zinc center is a distorted tetrahedron (*τ* = 0.75).^47^ The supporting ligands are bound in a κ^2^ fashion *via* the pyrazole nitrogens, while the phosphine moieties are not coordinated and the benzoxide groups are in a bridging position between the two metal centers.1
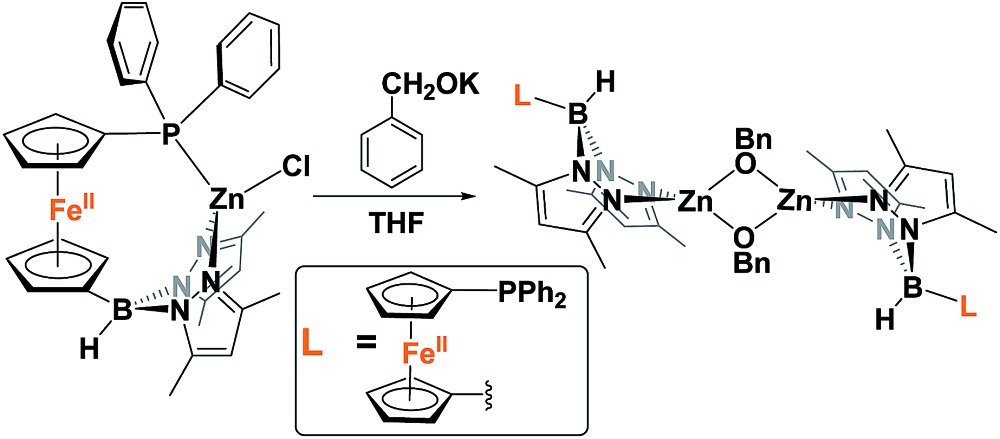



In solution, a single species is observed by NMR spectroscopy (Fig. S1–S4†), with the resonance signals similar to those of previously reported (fc^P,B^)Zn complexes.^37^ For example, the ^31^P{^1^H} NMR spectrum of [(fc^P,B^)Zn(μ-OCH_2_Ph)]_2_ shows a singlet at *δ* = –15.5 ppm. Similar chemical shifts of *δ* = –16.4 and –15.5 ppm were observed for a coordinated phosphine in (fc^P,B^)ZnCl and a free phosphine in fc(PPh_2_)[B(OMe_3_)_2_], respectively.^37^ Such minor differences in the ^31^P{^1^H} NMR spectra between free and zinc(ii)-coordinated phosphines are commonly observed and are attributed to weak interactions between the soft phosphine ligands and the oxophilic zinc(ii) centers.^48^ Diffusion ordered spectroscopy (DOSY) NMR^49^ experiments were conducted with (fc^P,B^)ZnCl and [(fc^P,B^)Zn(μ-OCH_2_Ph)]_2_ (Fig. S15 and S16†) to determine if the latter exists as a dimer in solution. Based on the Stokes–Einstein relationship,^49^ the ratio of the radii of [(fc^P,B^)Zn(μ-OCH_2_Ph)]_2_ to (fc^P,B^)ZnCl is 1.63. This value is somewhat below the expected value of 2 for the dimer, as derived from the comparison of volumes from the solid state structures. However, ^1^H Nuclear Overhauser Effect Spectroscopy (NOESY) studies of [(fc^P,B^)Zn(μ-OCH_2_Ph)]_2_ show a binding motif similar to that observed in the solid state structure. Interactions between the protons of the pyrazole methyl groups and the benzoxide ligand are observed in the 2D plot, while the interactions between the phosphine phenyl groups and the benzoxide are not observed (Fig. S10 and S11†). Additionally, a variable temperature NMR study was performed. The spectra of [(fc^P,B^)Zn(μ-OCH_2_Ph)]_2_ show no significant changes in the range of 298–352 K (Fig. S9†), suggesting that the speciation of the complex remains the same in solution even at elevated temperatures. The addition of an excess of a hard Lewis base, such as pyridine, to [(fc^P,B^)Zn(μ-OCH_2_Ph)]_2_ in C_6_D_6_ yields a simple mixture of the two compounds at ambient temperature (Fig. S14†). A lack of an interaction between the zinc complex and pyridine suggests that Lewis bases, similar to monomers prior to being ring opened, do not disrupt the dimeric structure of the zinc complex.

The stability of [(fc^P,B^)Zn(μ-OCH_2_Ph)]_2_ was evaluated both in the presence and absence of a substrate. In the absence of a monomer, [(fc^P,B^)Zn(μ-OCH_2_Ph)]_2_ slowly decomposes in benzene at ambient temperature, reaching 7.0% decomposition after 24 h (Fig. S26†). Heating the compound at 100 °C in benzene results in 34% decomposition after 1.5 h (Fig. S27†). However, in the presence of a monomer, no decomposition is observed, even at elevated temperatures (70 °C) for 3 h (Fig. S28†).

**Fig. 2:**
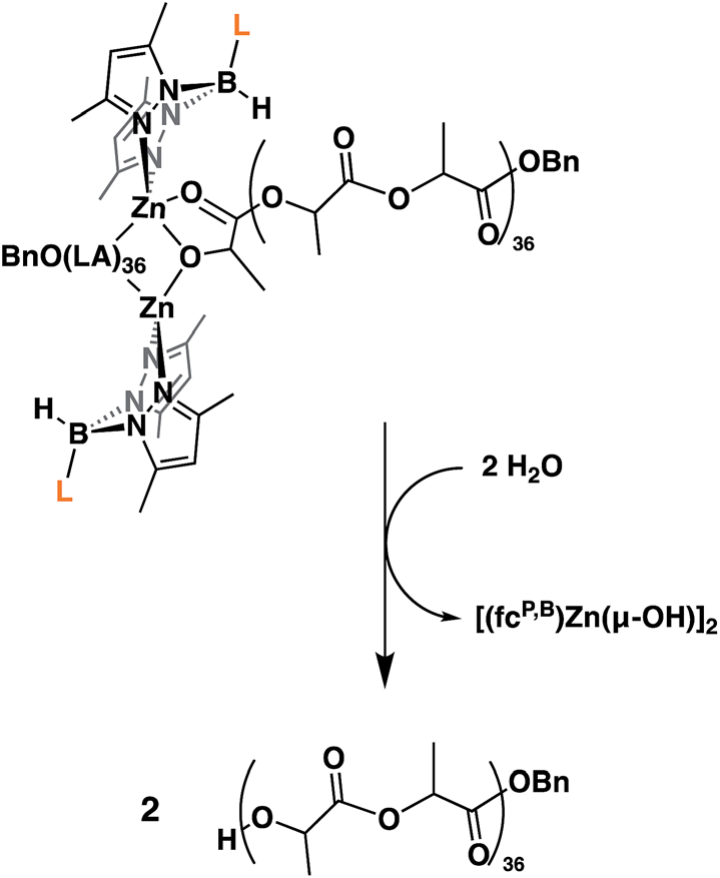
Illustration of l-lactide polymerization quenching undertaken for the DOSY NMR experiment.

Next, we looked at the identity of the catalytically active species in the case of each monomer. In order to evaluate if it remains a dimer during polymerizations, an attempt to characterize the product corresponding to the ring opening of a single equivalent of monomer was made. Due to its slow rate of polymerization at ambient temperature, l-lactide was chosen as the model substrate. On an NMR scale, addition of two equivalents of l-lactide to [(fc^P,B^)Zn(μ-OCH_2_Ph)]_2_ resulted in the formation of a single major species (Fig. S12†) after 2 hours at ambient temperature. Performing a DOSY NMR experiment on this product yielded a slower diffusion rate than for the parent complex (Fig. S17†), consistent with the retention of the dimeric state post incorporation of one equivalent of l-lactide per metal center. These results are reproduced during quenching experiments of l-lactide polymerizations (Fig. 2). A DOSY NMR experiment performed with [(fc^P,B^)Zn(PLA)_36_(OCH_2_Ph)]_2_ yielded a diffusion rate of 1.04 × 10^–6^ m s^–2^ (Fig. S20†). Water was then added to the same sample resulting in the hydrolysis of the polymer chains from the zinc catalyst and the formation of [(fc^P,B^)Zn(μ-OH)]_2_. The free polymers, PhCH_2_O(PLA)_36_H, displayed a diffusion rate of 2.00 × 10^–6^ m s^–2^ (Fig. S21†). Since the diffusion rate of a molecule is inversely proportional to its hydrodynamic radius, two polymer chains bound together by a catalyst will diffuse at half the rate of a single polymer chain. The doubling of the diffusion rate upon hydrolysis of the active polymerization species is consistent with the liberation of polymer chains from a dimeric species. Similar results were obtained in the case of TMC polymerization (Fig. S18 and S19†) suggesting that the catalytically active species is a dimer in both cases.

**Fig. 3:**
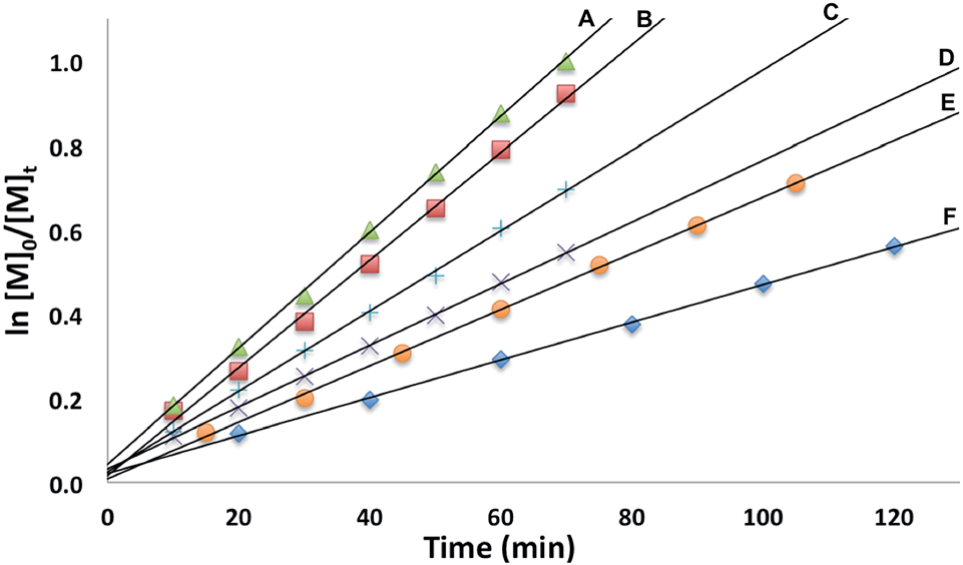
Semilogarithmic plots of l-lactide conversion with time in C_6_H_6_ at 70 °C with [(fc^P,B^)Zn(μ-OCH_2_Ph)]_2_ as a catalyst ([LA]_0_ = 0.313 M: (A) [Zn] = 4.69 mM, [LA]/[Zn] = 67; (B) [Zn] = 3.91 mM, [LA]/[Zn] = 80; (C) [Zn] = 3.13 mM, [LA]/[Zn] = 100; (D) [Zn] = 2.34 mM, [LA]/[Zn] = 133; (E) [Zn] = 1.88 mM, [LA]/[Zn] = 167; (F) [Zn] = 1.56 mM, [LA]/[Zn] = 200).

**Fig. 4:**
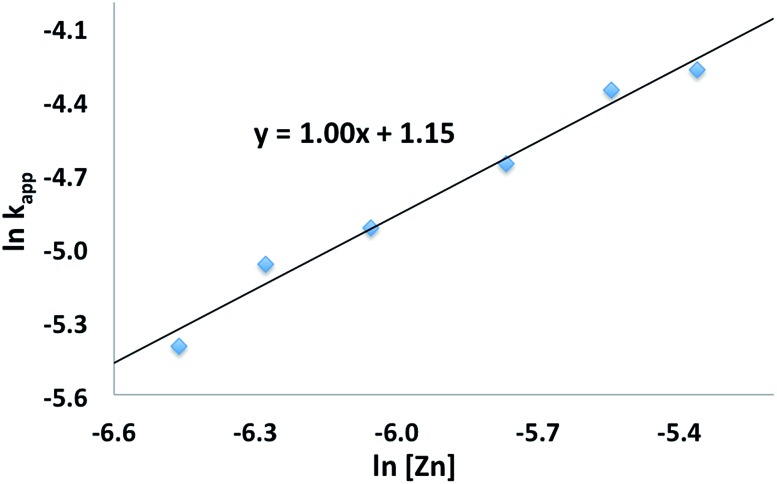
Plot of ln *k*_app_*vs.* ln[Zn] for the polymerization of l-lactide with [(fc^P,B^)Zn(μ-OCH_2_Ph)]_2_ as a catalyst (C_6_H_6_, 70 °C, [LA]_0_ = 0.313 M).

The conversion of l-lactide was monitored by ^1^H NMR spectroscopy for varying concentrations of monomer, in benzene at 70 °C. In all cases, first-order kinetics were observed *via* the semilogarithmic plots of several polymerizations (Fig. 3). The order in pre-catalyst was determined *via* the logarithmic plot of the metal complex concentration against *k*_app_ (Fig. 4) displaying first-order kinetics and yielding the following rate law (eqn (2)):

2 –d[LA]/d*t* = *k*[Zn_2_]^1^[LA]^1^

A first-order in both monomer and pre-catalyst is commonly observed for metal mediated ring-opening polymerizations. In particular, a clear order in catalyst is consistent with a well-behaved system in solution and the retention of the dimeric state by the catalyst throughout the polymerization process.^50^,^51^

Finally, we looked at the preparation of LA/TMC homopolymers as well as, in keeping with the *ca.* 20% by weight optimal composition, the preparation of a variety of multiblock copolymers. In all cases, the multiblock copolymers were prepared *via* the sequential addition of monomer to the growing polymer chain. Utilizing our system, the copolymerization of TMC and LA is not limited by the order of monomer addition. The percent by weight composition of TMC was kept within 15–20%, and the number average molar mass was kept at *ca.* 50 000 g mol^–1^. We reasoned that attempting to maintain these variable relatively constant would allow us to probe the influence that the copolymer microstructure has on the physical properties of the corresponding materials.

Polymerization of *ca.* 100 equivalents of TMC (Table 1, entry 2) reaches completion at room temperature within one hour. Polymerization of l-lactide at room temperature is much slower and requires up to 24 hours for the same number of equivalents to reach completion. Raising the temperature to 70 °C results in a complete conversion within an hour. In both cases, the polymerizations are well controlled. The molar masses increase with conversion while retaining low dispersity (*Đ*) values (Fig. S45 and S46 and Tables S1 and S2†).

**Table 1:** Addition copolymerization of l-lactide and 1,3-trimethylene carbonate^*a*^

Entry	Polymer	PTMC (wt%)	PLA (wt%)	*M*_n_ (TMC, NMR)	*M*_n_ (LA, NMR)	*M*_n_ (NMR)	*M*_n_ (SEC)	*Đ*
1	PLA	—	100	—	—	40.7	39.8	1.14
2	PTMC	100	—	—	—	10.4	9.0	1.01
3	PLA-*b*-PTMC	19	81	10.0	43.7	53.7	55.5	1.12
4	PTMC-*b*-PLA	17	83	8.0	39.5	47.5	47.0	1.60
5	PTMC-*b*-PLA-*b*-PTMC	18	82	8.7	40.8	49.5	43.2	1.67
6	PLA-*b*-PTMC-*b*-PLA	17	83	9.0	43.7	52.7	55.6	1.46
7	PLA-*b*-PTMC-*b*-PLA-*b*-PTMC	19	81	10.2	42.9	53.1	48.2	1.49
8	PTMC-*b*-PLA-*b*-PTMC-*b*-PLA-*b*-PTMC	18	82	9.8	45.2	55.0	58.9	1.49
9	PLA-*b*-PTMC-*b*-PLA-*b*-PTMC-*b*-PLA	19	81	10.0	42.3	52.3	53.2	1.69
10	PLA-*b*-PTMC-*b*-PLA	10	90	5.2	47.5	52.7	50.8	1.29
11	PLA-*b*-PTMC-*b*-PLA	30	70	15.9	36.8	52.7	48.9	1.42
12	PLA-*b*-PTMC-*b*-PLA	39	61	22.1	34.5	56.6	51.2	1.68

^*a*^Conditions: benzene as a solvent (1.5 mL) and hexamethylbenzene as an internal standard. All experiments were performed at 70 °C, except for those corresponding to entry 2 and the first blocks of entries 3, 5, 7, and 8, which were performed at ambient temperature. The order of block preparation is illustrated from right to left in the final copolymer. The respective monomer loading (Fig. S31–S40) is distributed evenly between the blocks of each type. *M*_n_ are reported in 10^3^ g mol^–1^; *Đ* = *M*_w_/*M*_n_. Values for *M*_n_ calculated using NMR spectroscopy are based on integration of polymer peaks *versus* the internal standard and take into account monomer conversion.

**Fig. 5:**
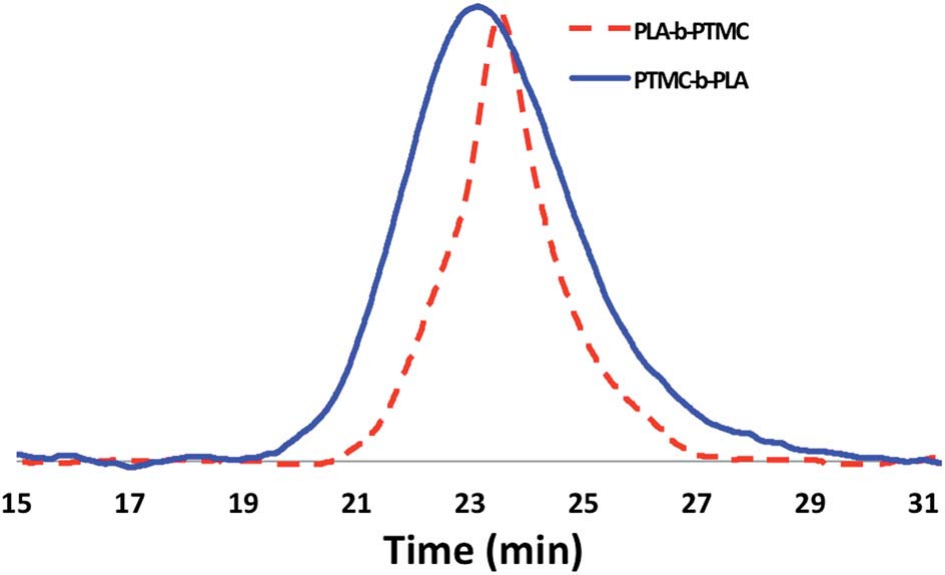
SEC traces of PLA-*b*-PTMC (Table 1, entry 3) and PTMC-*b*-PLA (Table 1, entry 4) copolymers.

Although the homopolymerization of TMC proceeds quickly at ambient temperature, elevated temperatures are required to polymerize it after l-lactide due to the nature of the intermediate formed after the ring opening of lactide that features a five-membered chelate.^32^,^52^–^57^ This difference in shifting the polymerization of TMC from room temperature, as in the case of PLA-*b*-PTMC (Table 1, entry 3), to elevated temperatures, as in the case of PTMC-*b*-PLA (Table 1, entry 4), manifests itself in the broadening of the molar mass distributions (Fig. 5). As a result, the dispersity values are larger for the copolymers subjected to TMC polymerization at elevated temperatures, ranging from 1.45 to 1.69 (Table 1, entries 4–9), then for the polymers that were not (Table 1, entries 2–3).

**Fig. 6:**
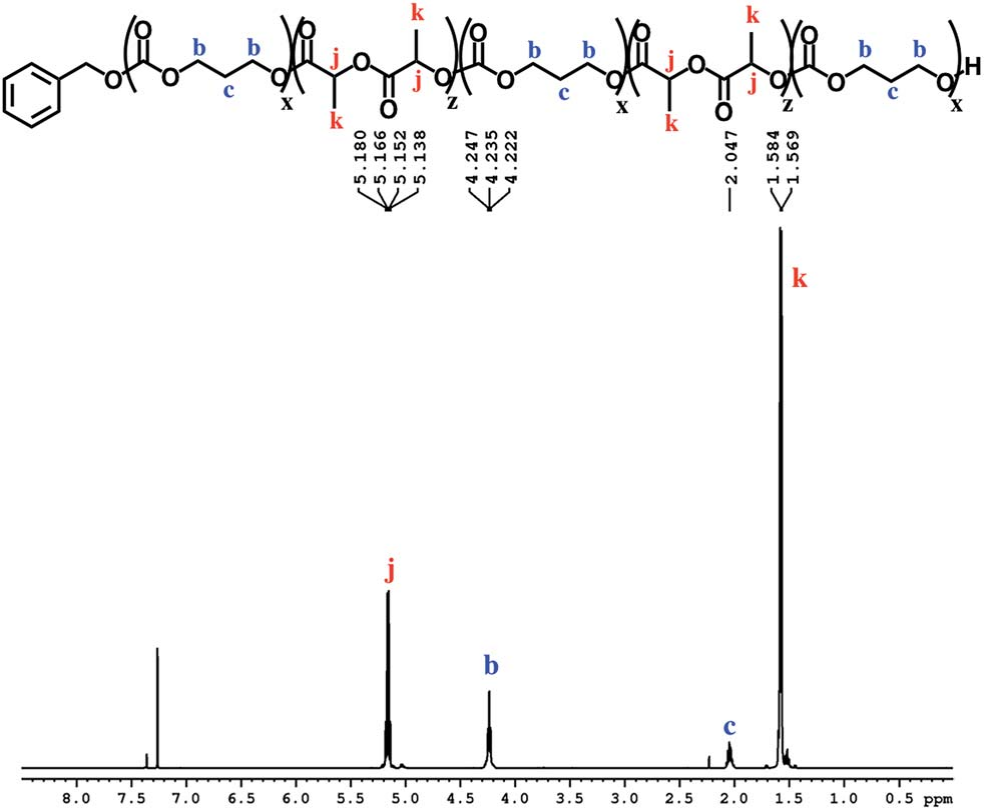
^1^H NMR spectrum (CDCl_3_, 500 MHz, 298 K) of PTMC-*b*-PLA-*b*-PTMC-*b*-PLA-*b*-PTMC (Table 1, entry 8); see Fig. S36† for integration values.

**Fig. 7:**
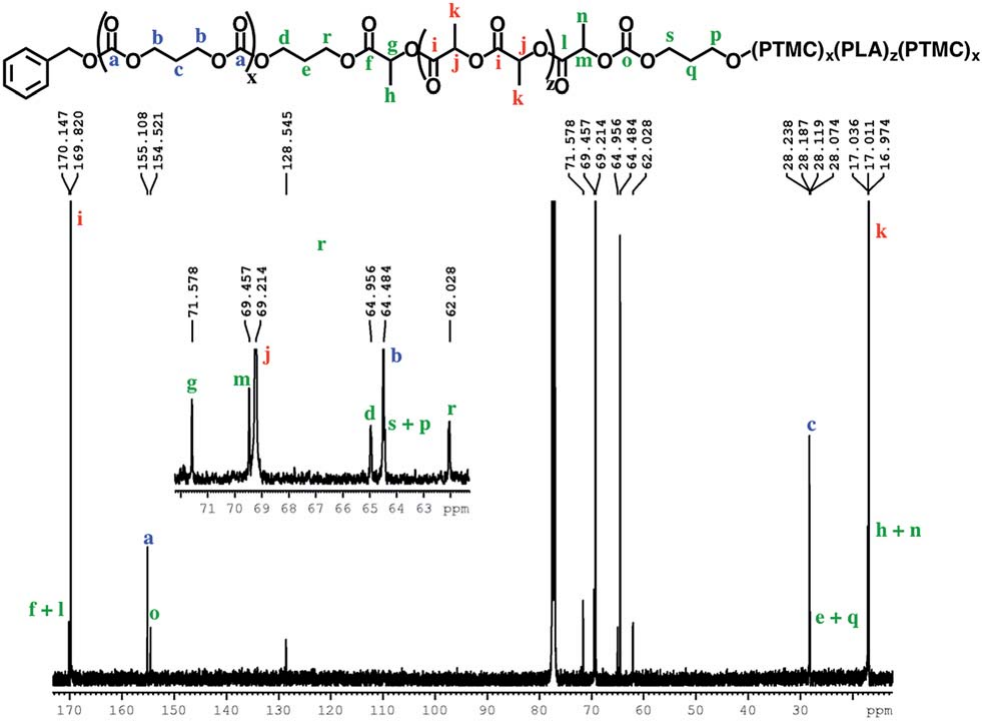
^13^C{H} NMR spectrum (CDCl_3_, 500 MHz, 298 K) of the PTMC-*b*-PLA-*b*-PTMC-*b*-PLA-*b*-PTMC copolymer.

**Fig. 8:**
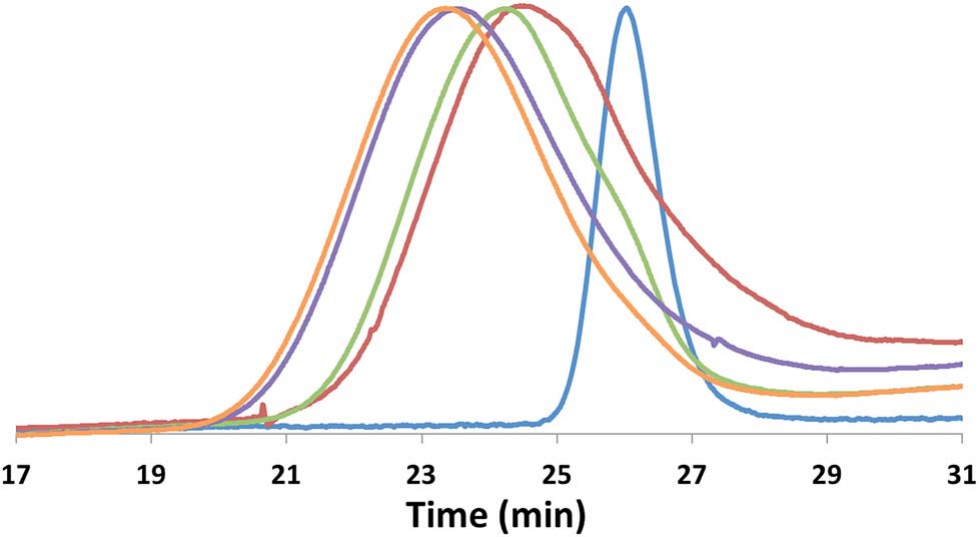
SEC traces corresponding to the stepwise preparation of PLA-*b*-PTMC-*b*-PLA-*b*-PTMC-*b*-PLA (*M*_n_ are reported in 10^3^ g mol^–1^; *Đ* = *M*_w_/*M*_n_): PLA (blue, *M*_n_ = 13.5, *Đ* = 1.09); PTMC-*b*-PLA (red, *M*_n_ = 20.0, *Đ* = 1.25); PLA-*b*-PTMC-*b*-PLA (green, *M*_n_ = 32.9, *Đ* = 1.29); PTMC-*b*-PLA-*b*-PTMC-*b*-PLA (purple, *M*_n_ = 40.0, *Đ* = 1.42); PLA-*b*-PTMC-*b*-PLA-*b*-PTMC-*b*-PLA (orange, *M*_n_ = 45.1, *Đ* = 1.43).

The block structures of the polymers are consistent with observations from the ^1^H NMR spectra. In all cases, the copolymer peaks appear as a superposition of the signals corresponding to individual blocks (Fig. 6 and S31–S40†), a defining characteristic of true block copolymers.^20^ Alternatively, both gradient and random block copolymers of TMC and LA yield broadened peaks for PTMC and a distribution of peaks in the methine region of PLA.^20^ The junctions of the copolymer^19^,^58^ can also be clearly observed in the ^13^C NMR spectrum of the pentablock copolymers (Fig. 7 and S41†).^19^,^20^ DOSY NMR experiments carried out with the triblock and pentablock copolymers (Fig. S22–S25†) show the same diffusion rate for both the PLA and the PTMC segments in all cases, further supporting a block copolymer formation. Additionally, ^1^H NMR spectra of aliquots collected during the preparation of the PLA-*b*-PTMC-*b*-PLA-*b*-PTMC-*b*-PLA copolymer show the stepwise growth of each block (Fig. S44†). Similarly, the corresponding SEC (size exclusion chromatography) traces of the same aliquots show an increase in molar mass with every additional block (Fig. 8). The benzoxide end group is clearly observed and diffuses at the same rate as the polymers in DOSY NMR spectra for both homopolymers, both in the case of the polymers still attached to the catalyst and in free polymers (Fig. S18–S21†). The downfield shift in ^1^H NMR spectra of the benzoxide methylene protons from 4.03 ppm in the parent complex to 4.72 ppm and 4.94 ppm in the ring-opening polymerization products of LA and TMC, respectively, is also indicative of the participation of the benzoxide group in the ring-opening process of the monomers.^19^ The experiments described above suggest that these polymerization processes proceed *via* a living mechanism.^59^

The differential scanning calorimetry (DSC) curves for the newly synthesized block copolymers display *T*_g_ and *T*_m_ values corresponding to isotactic PLA only (Table 2 and Fig. S64–S70†). Even at high sample loadings, the *T*_g_ corresponding to PTMC could not be detected (Fig. S63†), likely due to the relatively low content of PTMC in each copolymer. Only when we examined copolymers with a *ca.* 40% weight composition of TMC, could we detect the *T*_g_ corresponding to PTMC (Table 2, entry 11; Fig. S73†). In general, both the *T*_g_ and the *T*_m_ values are observed to decrease with the increasing number of blocks in the polymer. This depression of the *T*_g_ and *T*_m_ values is a known phenomenon in poly(l-lactide) chemistry;^60^ the inclusion of amorphous polymer segments influences the crystallization behavior of the semicrystalline PLA fragments and improves the polymer chain mobility.^61^–^63^

**Table 2:** Polymer thermal and mechanical properties

Entry	Polymer structure	PTMC (wt%)	*T*_g_ ^*a*^ (°C)	*T*_g_ ^*a*^ (°C)	*T*_m_ ^*a*^ (°C)	*E* ^*b*^ (MPa)	*σ* ^*c*^ (MPa)	*ε* ^*d*^ (%)
1	PLA	0	—	55	173	1733 ± 108	49 ± 3	11 ± 4
2	PLA-*b*-PTMC	19	—	42	173	865 ± 85	36 ± 5	18 ± 3
3	PTMC-*b*-PLA	17	—	37	164	763 ± 135	37 ± 5	23 ± 4
4	PTMC-*b*-PLA-*b*-PTMC	18	—	35	161	521 ± 30	24 ± 2	249 ± 32
5	PLA-*b*-PTMC-*b*-PLA	17	—	35	165	382 ± 61	12 ± 4	219 ± 44
6	PLA-*b*-PTMC-*b*-PLA-*b*-PTMC	19	—	34	165	471 ± 147	27 ± 0	208 ± 47
7	PTMC-*b*-PLA-*b*-PTMC-*b*-PLA-*b*-PTMC	18	—	34	160	334 ± 70	21 ± 2	176 ± 23
8	PLA-*b*-PTMC-*b*-PLA-*b*-PTMC-*b*-PLA	19	—	34	153	303 ± 44	20 ± 1	251 ± 32
9	PLA-*b*-PTMC-*b*-PLA	10	—	43	163	545 ± 145	41 ± 2	18 ± 3
10	PLA-*b*-PTMC-*b*-PLA	30	—	40	161	332 ± 48	22 ± 4	81 ± 11
11	PLA-*b*-PTMC-*b*-PLA	39	–13	9	157	364 ± 64	21 ± 4	257 ± 13

^*a*^Glass transition temperatures and melting points were determined using DSC.

^*b*^Young's modulus.

^*c*^Ultimate tensile strength.

^*d*^Elongation at break. Material properties corresponding to entries 2 and 3 are averages of two different batches of materials (Fig. S75 and S76). Average values for multiple runs are reported along with the standard error.

The mechanical properties of the polymers were determined *via* dynamic mechanical analysis (DMA, Table 2 and Fig. S74–S81†) on multiple samples of each copolymer prepared *via* a solvent casting method. The PLA homopolymer displayed a Young's modulus of 1733 MPa and an elongation at break value of 11% (Table 2, entry 1). Physical blends of PLA and PTMC show a higher Young's modulus and a lower increase in the elongation at break than the copolymer corresponding to the same weight percentage composition.^64^ The copolymers display lower Young's modulus values than PLA, consistent with the addition of a soft PTMC fragment,^65^ and, in most cases, display an order of magnitude improved elongation at break values. The diblock copolymers showed a lower Young's modulus and a minor improvement in the elongation at break of up to 23% (Table 2, entries 2 and 3). As the number of blocks increases to three or more, we observed a decrease in the Young's moduli while the elongation at break values were drastically improved up to 250% (Table 2, entries 4–8). Therefore, increasing the number of blocks while maintaining a consistent monomer composition results in copolymers with improved elasticity. Particularly in the case of the pentablock copolymers, the materials possess low Young's moduli and high elongation at break values while maintaining thermal properties similar to the rest of the block copolymers.

An inverse relationship between Young's modulus and elongation at break values was observed by Guerin *et al.* upon increasing the percent composition of TMC in their copolymers.^19^ We also prepared several triblock copolymers with different percent compositions of TMC (Table 1, entries 10–12; Table 2, entries 9–11) to study the effects of varying the TMC concentration in our copolymers. Lowering the TMC percent composition to 10% yielded a brittle material similar to PLA but with a lower Young's modulus than that of the homopolymer. On the other hand, when the TMC composition in the copolymer was increased to *ca.* 30% and 40% by weight we observed a similar inverse relationship between the Young's modulus and the elongation at break of the materials. Based on these results, a further increase in the PTMC composition would have a negative impact on the Young's modulus of the materials at the expense of an increased elongation at break. The copolymers with increased TMC loadings also show a drastic deviation in the glass transition temperature from the 20% weight PTMC multiblock copolymers. Therefore, multiblock copolymers derived from consistent monomer ratios yield materials with a unique combination of thermal and mechanical properties for various specialty applications.

Finally, to test the applicability of this system under industrially relevant conditions, we carried out some polymerizations under solvent-free conditions *via* monomer melts. The syntheses of PLA (Fig. S41 and S60†) and PTMC-*b*-PLA (Fig. S42 and S61†) were carried out at 140 °C in the absence of benzene. Although the isolated polymers displayed unimodal distributions in the SEC traces and narrow dispersities (Fig. S60 and S61†), the amount of TMC incorporated in the copolymer was very small (Fig. S42†). This is likely due to the viscous nature of PLA preventing a thorough mixing of TMC during its sequential addition. Further optimization of the reaction conditions could provide a viable method for the preparation of various multiblock copolymers under solvent-free conditions.

### DFT calculations

To gain a better understanding of the mechanism, we turned to density functional theory.^66^,^67^ All calculations were carried out with the Gaussian 09 program package^68^ on the Extreme Science and Engineering Discovery Environment (XSEDE).^69^ The methyl groups on the pyrazole substituents were replaced by hydrogen atoms and the phenyl groups on PPh_2_ were replaced by methyl groups to simplify the calculations (for more details about calculations, see the ESI†). First, possible monomeric and dimeric structures of the zinc benzoxide complexes were optimized and their energies compared (Fig. S86†). The energy of the dimer [(fc^P,B^)Zn(μ-OCH_2_Ph)]_2_ was lower by 3.3 kcal mol^–1^ than that of the corresponding monomer, (fc^P,B^)Zn(OCH_2_Ph), in agreement with the experimental observations.

Since the energy difference between the dimeric and the monomeric species was small, the free energy surfaces for the reaction with LA and TMC were thus computed for both the monomer and the dimer (Fig. S87 and S88†) to compare the initiation step. For LA, although the monomer shows a lower activation barrier than the dimer (by 2.7 kcal mol^–1^) for the alkoxide nucleophilic attack (TS_I–II_), the energy for the ring opening step (TS_II–III_) and the overall activation barrier are lower for the dimeric species than for the monomer by 4.2 and 4.4 kcal mol^–1^, respectively; furthermore, the two zinc centers participate in the process synergistically when the reaction occurs with the dimer. Similarly, for the initiation of TMC, both activation barriers were lower for the dimer (by 3.1 and 1.6 kcal mol^–1^). These results are again in agreement with the experimental observations discussed above that the dimeric zinc complex facilitates the polymerization.

**Fig. 9:**
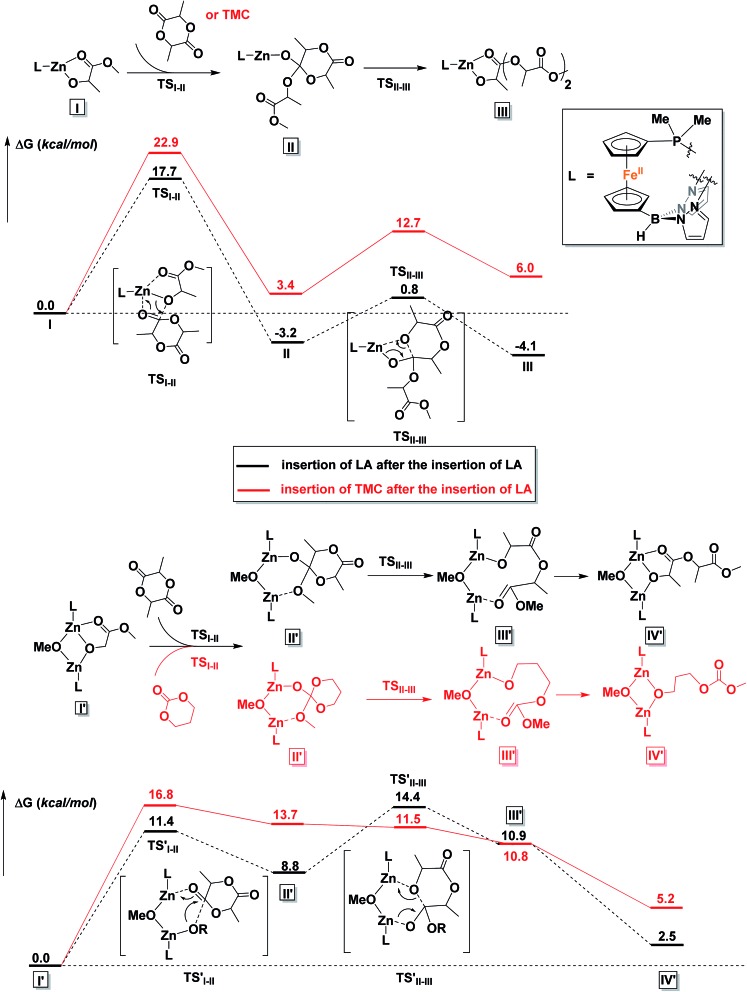
Comparison of reaction coordinates for propagation catalyzed by a monomeric (top) or dimeric (bottom) form of the zinc complex.

The copolymerization steps were then considered. Since the insertion of TMC leads to a product that has a similar structure as the step before, each following insertion should be similar to the initiation step, making the homopolymerization and copolymerization possible. However, after the insertion of LA, the resulting product contains a five-membered ring, in which the bond between the Zn center and the carbonyl group cannot be ignored. Thus, the insertions of a second LA or TMC molecule, respectively, after the insertion of the first LA were considered. As shown in Fig. 9, the dimeric species significantly lowers the overall activation barriers, thus making the propagations possible after the insertion of LA. We would like to note that we are treating the results shown in Fig. 9 from a qualitative point of view that allows us to compare the behavior of LA *versus* TMC. The large number of atoms involved and the simplifications necessary in order to get the respective transition states and intermediates to converge in a reasonable amount of time likely resulted in obtaining energies for the products that are positive with respect to the starting materials.

It is interesting to observe that after the insertion of LA, the insertion of another LA is easier than the insertion of TMC. Based on these results, we can envision that although the homopolymerization of TMC is much easier than that of LA, during the copolymerization of LA and TMC in one pot, LA would be consumed first (Fig. S47†).

## Conclusions

We report the ring opening copolymerization of LA and TMC to obtain pentablock copolymers, by the multiple step-wise addition of either monomer to the other, without the need for synthesizing tailored initiators or using any other additives. These reactions were possible with a dimeric zinc catalyst, [(fc^P,B^)Zn(μ-OCH_2_Ph)]_2_. The solution state behavior of [(fc^P,B^)Zn(μ-OCH_2_Ph)]_2_ in the presence and absence of LA and TMC was thoroughly investigated in order to understand why this catalyst does not have the limitations of previously reported compounds that cannot polymerize TMC after LA. Utilizing diffusion ordered NMR spectroscopy, as well as other spectroscopic techniques, the retention of the dimeric state of the zinc complex in solution was confirmed. It was also found that the zinc complex reacts as a dimer when catalyzing the ROP of l-lactide and 1,3-trimethylene carbonate. A combination of molar mass *versus* conversion, end group analysis, chain extension, and kinetics experiments, as well as great control over the polymer molar masses, suggests that these polymerization processes proceed *via* a living mechanism.

The preparation of various multiblock copolymers was achieved by a simple step-wise addition of the cyclic ester and carbonate in the presence of the catalyst. The physical, thermal, and mechanical properties of the isolated copolymers were determined using NMR spectroscopy, SEC, DSC, and DMA. In all cases, the block-like structures of the isolated polymers could be observed by NMR spectroscopy and the theoretical molar masses agreed well with the SEC results. Furthermore, a clear trend in the influence of the block structures on the thermal and mechanical properties was observed; with an increasing number of blocks, a decrease in the glass transition temperatures, melting point temperatures, and the Young's modulus was observed. This study shows that multiblock copolymers derived from consistent monomer ratios yield materials with a unique combination of thermal and mechanical properties that may be used for various specialty applications.

To gain further insight into the polymerization mechanism, density functional theory calculations were performed. The DFT calculations indicate that: (1) in solution, the dimeric zinc species is more favored than the monomeric species; (2) the dimeric zinc species has lower overall activation barriers compared to the monomer; (3) both the polymerization of LA and TMC are possible with the dimeric catalyst and the rate of the polymerization of TMC is faster than that of LA; (4) however, after the insertion of LA, the insertion of another LA is easier than the insertion of TMC. However, obtaining an accurate description of the polymerization processes during copolymerization was hindered by the large and complex nature of our system.

## Experimental section

### General considerations

All reactions were performed using standard Schlenk techniques or in an MBraun drybox (<1 ppm O_2_/H_2_O) unless noted otherwise. All glassware, cannulas, and Celite were stored in an oven at >425 K before being brought into the drybox. Solvents were purified using a two-column solid-state purification system by the method of Grubbs^70^ and transferred to the glovebox without exposure to air. NMR solvents were obtained from Cambridge Isotope Laboratories, degassed, and stored over activated molecular sieves prior to use. NMR spectra were recorded at ambient temperature on Bruker AV-300, AV-400, AV-500, and DRX-500 spectrometers unless otherwise noted. Proton and carbon chemical shifts are given relative to residual solvent peaks. Phosphorus, boron, and fluorine chemical shifts are given relative to external standards, H_3_PO_4_ and Et_2_O·BF_3_ in C_6_D_6_, respectively. Hexamethylbenzene was purchased from Sigma Aldrich and passed through activated alumina in toluene prior to use. l-Lactide was purchased from TCI and recrystallized from THF/diethyl ether layering prior to use. The 1,3-trimethylene carbonate^71^ and (fc^P,B^)ZnCl·(C_7_H_8_)^37^ were prepared using literature procedures and, unless otherwise noted, all reagents were acquired from commercial sources and used as received. Elemental analysis of compound [(fc^P,B^)Zn(μ-OCH_2_Ph)]_2_ was performed on an Exeter Analytical, Inc. CE-440 Elemental Analyzer. Molar masses of the polymers were determined by SEC MALS using a Shimazu Prominence-i LC 2030C 3D equipped with an autosampler, two MZ Analysentechnik MZ-Gel SDplus LS 5 μm, 300 × 8 mm linear columns, a Wyatt DAWN HELEOS-II, and a Wyatt Optilab T-rEX. The column temperature was set at 40 °C. A flow rate of 0.70 mL min^–1^ was used, and samples were dissolved in chloroform. The number average molar mass and dispersity were found using the known concentration of the sample in chloroform with the assumption of 100% mass recovery to calculate d*n*/d*c* from the RI signal. DSC was obtained using a PerkinElmer DSC model 8000 heat flow system with Intracooler II. The method used was to increase the temperature from –40 to 220 °C at 10 °C min^–1^, held 220 °C for 2 min, then decreased back to –40 °C at 10 °C min^–1^ for three cycles. Mechanical properties were measured on a TA Instruments RSA III dynamic mechanical analyzer (DMA). Modulus tests were conducted at 20 °C and a frequency of 1 Hz with samples of 6.0 mm wide and ∼40 μm thick loaded onto the DMA with a 3 mm gap between the thin film grips. The stress–strain curves of the films were obtained at 20 °C at a stretching rate of 1 mm s^–1^. The tested samples used were 6.0 mm wide and ∼40 μm thick with a 3 mm gap between the thin film grips of the DMA. A minimum of three samples was tested per polymer.

### [(fc^P,B^)Zn(μ-OCH_2_Ph)]_2_

To KCH_2_Ph (82.4 mg, 0.633 mmol) in 5 mL of THF at –78 °C was added HOCH_2_Ph (66.0 μL, 0.633 mmol) drop-wise *via* syringe until the solution became colorless. A THF solution of (fc^P,B^)ZnCl·(C_7_H_8_) (483.7 mg, 0.633 mmol) was then added drop-wise and the reaction mixture stirred for 1 h at –78 °C. The reaction vessel was brought to ambient temperature and volatile substances were removed under reduced pressure. The desired product was extracted with 5 mL of toluene and filtered through Celite. Toluene was removed under reduced pressure and the remaining oily orange solids were dissolved in 5 mL of diethyl ether. After several minutes, a copious amount of yellow solids precipitated from diethyl ether. The solids were collected and washed with diethyl ether until the washings became pale yellow. The final product was isolated as yellow crystals in two crops from a THF/diethyl ether (1 : 2) mixture at –35 °C (354.2 mg, 68.5%). Crystals of [(fc^P,B^)Zn(μ-OCH_2_Ph)]_2_ always contain two molecules of solvent per molecule of compound as a mixture of THF and diethyl ether as supported by NMR spectroscopic data. X-ray quality crystals were obtained from a THF/diethyl ether layering at –35 °C. ^1^H NMR (C_6_D_6_, 500 MHz, 298 K): *δ* (ppm) 2.00 (s, 6H, C*H*_3_), 2.47 (s, 6H, C*H*_3_), 3.74 (t, 2H, Cp-*H*), 3.91 (t, 2H, Cp-*H*), 4.03 (s, 2H, OC*H*_2_Ph), 4.13 (q, 2H, Cp-*H*), 4.32 (t, 2H, Cp-*H*), 4.88 (br s, 1H, B*H*), 5.76 (s, 2H, C*H*), 6.69 (m, 2H, *o*-Ph), 6.81 (m, 2H, *m*-Ph), 6.87 (m, 1H, *p*-Ph), 7.04 (m, 6H, *m*-Ph, *p*-Ph), 7.53 (m, 4H, *o*-Ph). ^13^C NMR (C_6_D_6_, 126 MHz, 298 K): *δ* (ppm) 13.5 (s, *C*H_3_), 14.0 (d, *C*H_3_), 69.9 (s, Cp-*C*), 70.5 (s, O*C*H_2_Ph), 72.7 (d, Cp-*C*), 74.1 (s, Cp-*C*), 74.3 (d, Cp-*C*), 75.7 (d, Cp-*C*), 106.0 (s, *C*H), 127.3 (s, aromatic), 129.3 (s, aromatic), 134.4 (d, aromatic), 141.0 (d, aromatic), 144.3 (s, aromatic), 147.7 (s, *C*CH_3_), 150.2 (s, *C*CH_3_). ^31^P{^1^H} NMR (C_6_D_6_, 121 MHz, 298 K): *δ* (ppm) –15.5 (s). ^11^B NMR (C_6_D_6_, 161 MHz, 298 K): *δ* (ppm) –7.2 (br s). Anal. calcd: [(fc^P,B^)Zn(μ-OCH_2_Ph)]_2_·(THF)_2_ (C_86_H_96_B_2_Fe_2_N_8_O_2_P_2_Zn_2_) C, 63.30; H, 5.93; N, 6.87. Found: C, 63.76; H, 5.87; N, 7.01.

### [(fc^P,B^)Zn(μ-OH)]_2_

To [(fc^P,B^)Zn(μ-OCH_2_Ph)]_2_ (101.5 mg, 62.1 mmol), outside of the glove-box, was added 5 mL of wet diethyl ether and the mixture was stirred for 30 min at ambient temperature. The solution volume was reduced to 2 mL and yellow solids were collected on a frit and washed with 2 × 1 mL of cold diethyl ether. After drying under a reduced pressure for several hours, the final product was isolated as a yellow powder (70.8 mg, 87.2%). ^1^H NMR (C_6_D_6_, 500 MHz, 298 K): *δ* (ppm) –0.76 (s, 1H, O*H*), 2.24 (s, 6H, C*H*_3_), 2.37 (s, 6H, C*H*_3_), 3.40 (t, 2H, Cp-*H*), 3.48 (t, 2H, Cp-*H*), 3.99 (q, 2H, Cp-*H*), 4.21 (t, 2H, Cp-*H*), 4.69 (br s, 1H, B*H*), 5.76 (s, 2H, C*H*), 7.04 (m, 6H, *m*-Ph, *p*-Ph), 7.45 (m, 4H, *o*-Ph). ^13^C NMR (C_6_D_6_, 126 MHz, 298 K): *δ* (ppm) 13.4 (s, *C*H_3_), 13.7 (d, *C*H_3_), 69.9 (s, Cp-*C*), 72.6 (d, Cp-*C*), 74.5 (s, Cp-*C*), 74.6 (d, Cp-*C*), 76.0 (d, Cp-*C*), 105.1 (s, *C*H), 134.3 (d, aromatic), 140.1 (d, aromatic), 146.6 (s, *C*CH_3_), 148.6 (s, *C*CH_3_). ^31^P{^1^H} NMR (C_6_D_6_, 203 MHz, 298 K): *δ* (ppm) –15.6 (s). ^11^B NMR (C_6_D_6_, 161 MHz, 298 K): *δ* (ppm) –6.6 (br s). Anal. calcd: [(fc^P,B^)Zn(μ-OH)]_2_ (C_64_H_68_B_2_Fe_2_N_8_O_2_P_2_Zn_2_) C, 58.80; H, 5.24; N, 8.57. Found: C, 59.28; H, 5.26; N, 8.59.

### *In situ* generation of [(fc^P,B^)Zn(LA)(OCH_2_Ph)]_2_ and other NMR scale reactions

To a small vial, [(fc^P,B^)Zn(μ-OCH_2_Ph)]_2_ (5 μmol), the appropriate amount of monomer, and 0.5 mL of C_6_D_6_ were added. The contents of the vial were stirred and the homogeneous solution was transferred to a J. Young NMR tube equipped with a Teflon valve. The NMR tube was sealed, taken out of the box and placed in an oil bath. Monomer consumption was monitored by ^1^H NMR spectroscopy until the desired product was formed.

### General polymerization procedures

To a Schlenk flask, sealed with a Teflon screw cap, [(fc^P,B^)Zn(μ-OCH_2_Ph)]_2_ (2.5 μmol), an external standard, hexamethylbenzene (25 μmol), the appropriate amount of monomer, and up to 1.5 mL of C_6_H_6_ in total were added. The Schlenk flask was taken out of the glovebox and placed in an oil bath at 70 °C. Upon completion of each block, the reaction was cooled to room temperature and brought back into the glovebox for the addition of monomer comprising the next block. Typical reaction times for the complete conversion of 100 equivalents of l-lactide are 3–9 h in 0.5–1.5 mL of C_6_H_6_; 50 equivalents of TMC, after lactide, are polymerized over a period of 12–24 h in 0.5–1.5 mL of C_6_H_6_. The l-lactide and 1,3-trimethylene carbonate used in each polymerization experiment were distributed evenly across each block. Upon completion of the final block, the contents of the Schlenk flask were diluted with 1 mL of dichloromethane and poured into 15 mL of methanol to yield white solids. The product was collected on a glass frit, washed with additional 10 mL of methanol and kept under reduced pressure at 70 °C until it reached a consistent weight.

Melt polymerizations were carried out in a Schlenk tube equipped with a Teflon screw cap and a stir bar, at 140 °C, with a 600 : 1 monomer to [(fc^P,B^)Zn(μ-OCH_2_Ph)]_2_ ratio.

### General kinetics procedure

In a glove box a Schlenk flask, sealed with a Teflon screw cap, equipped with a stir bar an appropriate amount of [(fc^P,B^)Zn(μ-OCH_2_Ph)]_2_ and l-lactide were added with 1.6 mL as the final volume of C_6_H_6_. The flask was then taken out of the glove box and placed in an oil bath at 70 °C. At the appropriate time intervals the flask was removed from the bath and cooled under a flowing stream of cold water prior to being brought back into the glove box. Inside the box, aliquots were poured into hexanes, dried to a constant weight under reduced pressure, and analyzed by ^1^H NMR spectroscopy.

### X-ray crystallography

X-ray quality crystals were obtained from various concentrated solutions placed in a –40 °C freezer in the glove box unless otherwise specified. Inside the glove box, the crystals were coated with oil (STP Oil Treatment) on a microscope slide, which was brought outside the glove box. The X-ray data collections were carried out on a Bruker SMART 1000 single crystal X-ray diffractometer using MoK_α_ radiation and a SMART APEX CCD detector. The data was reduced by SAINTPLUS and an empirical absorption correction was applied using the package SADABS. The structure was solved and refined using SHELXTL (Brucker 1998, SMART, SAINT, XPREP and SHELXTL, Bruker AXS Inc., Madison, Wisconsin, USA). Tables with atomic coordinates and equivalent isotropic displacement parameters, with all the distances and angles, and with anisotropic displacement parameters are listed in the cif (CCDC ; 1580591).

### DFT calculations

All calculations were carried out with the GAUSSIAN 09.^68^ The methyl groups on the pyrazole substituents were replaced by hydrogen atoms and the phenyl groups on PPh_2_ were replaced by methyl groups to simplify the calculations. Geometry optimizations were performed with B3LYP.^72^–^74^ The LANL2DZ basis set^75^–^77^ with ECP was used for Fe, and the 6-31G(d) basis set^78^–^80^ was used for other atoms. Frequency analysis was conducted at the same level of theory to verify that the stationary points are minima or saddle points. The single point energies and solvent effects in benzene were computed with PBE1PBE/81SDD-6-311+G(d,p) basis sets^82^ by using the PCM solvation model.^83^ The D3 version of Grimme's dispersion was applied for the dispersion correction.^84^ All enthalpies and the Gibbs free energies are given in Hartree.

## Conflicts of interest

There are no conflicts to declare.

## Supplementary Material

Supplementary information

Crystal structure data
