# Supplementary material for: Preparation of multiblock copolymers via step-wise addition of l-lactide and trimethylene carbonate
Source: Chem Sci. 2018 Jan 11;9(8):2168–78. doi: 10.1039/c7sc04507g (PMC5903370; doi:10.1039/c7sc04507g)
Supplement: Supplementary file 1 [file SC-009-C7SC04507G-s001.pdf]

Electronic Supplementary Material (ESI) for Chemical Science.
This journal is © The Royal Society of Chemistry 2018

Supporting information for

Preparation of Multiblock Copolymers via Step-wise Addition Polymerization of L-lactide and Trimethylene Carbonate

*Mark Abubekerov, Junnian Wei, Kevin R. Swartz, Zhixin Xie, Qibing Pei, and Paula L. Diaconescu**

Department of Chemistry and Biochemistry, University of California, Los Angeles, CA 90095

Table of Contents

1. NMR Spectra	S2
2. Conversion Studies	S28
3. Size Exclusion Chromatography	S30
4. Differential Scanning Calorimetry	S37
5. Dynamic Mechanical Analysis	S43
6. X-ray Crystallographic Data	S49
7. DFT Calculations	S50
8. References	S148

NMR Spectroscopy

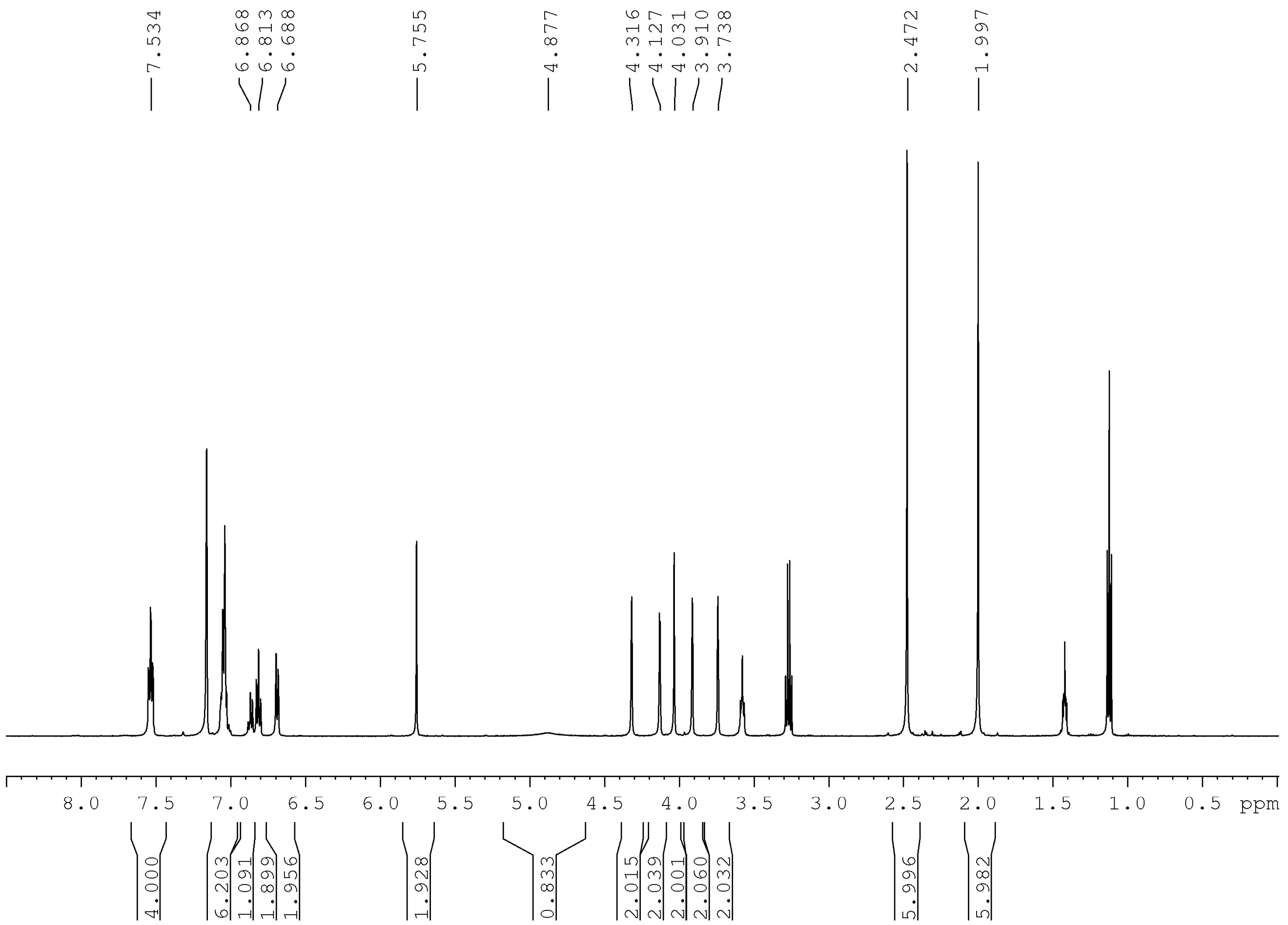

Figure S1. ^{1}H NMR spectrum (C_6D_6, 500 MHz, 298 K) of [(fc^{P,B})Zn(μ-OCH_2Ph)]$_2$: δ (ppm) 2.00 (s, 6H, CH_3), 2.47 (s, 6H, CH_3), 3.74 (t, 2H, Cp-*H*), 3.91 (t, 2H, Cp-*H*), 4.03 (s, 2H, OCH_2Ph), 4.13 (q, 2H, Cp-*H*), 4.32 (t, 2H, Cp-*H*), 4.88 (br s, 1H, B*H*), 5.76 (s, 2H, C*H*), 6.69 (m, 2H, *o*-Ph), 6.81 (m, 2H, *m*-Ph), 6.87 (m, 1H, *p*-Ph), 7.04 (m, 6H, *m*-Ph, *p*-Ph), 7.53 (m, 4H, *o*-Ph). Peaks at 1.12 ppm and 3.27 ppm are attributed to residual diethyl ether. Peaks at 1.42 ppm and 3.58 ppm are attributed to residual tetrahydrofuran.

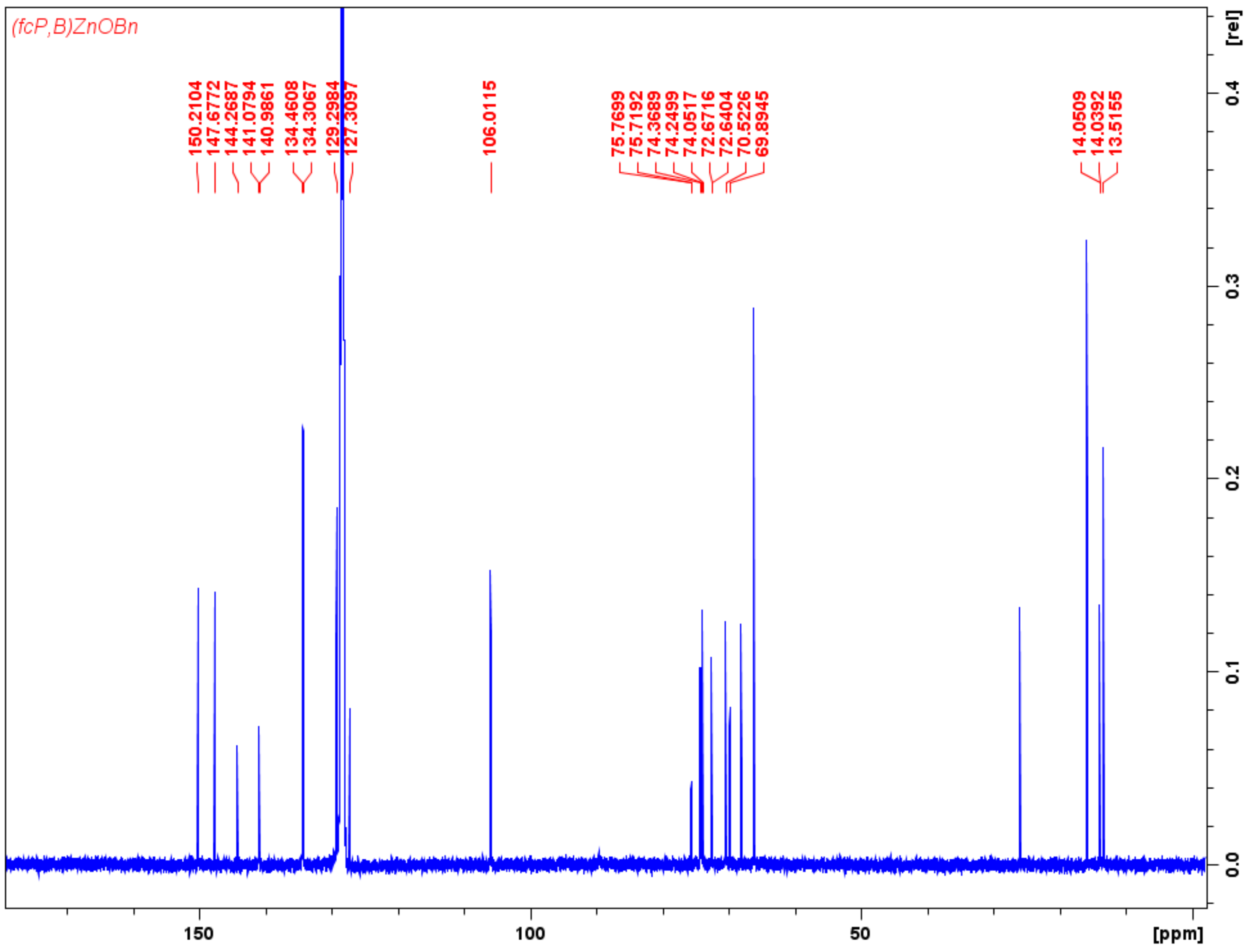

Figure S2. ^{13}C NMR spectrum (C_6D_6, 126 MHz, 298 K) of [(fc^{P,B})Zn(μ-OCH_2Ph)]$_2$: δ (ppm) 13.5 (s, CH_3), 14.0 (d, CH_3), 69.9 (s, Cp-*C*), 70.5 (s, O*C*H_2Ph), 72.7 (d, Cp-*C*), 74.1 (s, Cp-*C*), 74.3 (d, Cp-*C*), 75.7 (d, Cp-*C*), 106.0 (s, *C*H), 127.3 (s, aromatic), 129.3 (s, aromatic), 134.4 (d, aromatic), 141.0 (d, aromatic), 144.3 (s, aromatic), 147.7 (s, *C*CH_3), 150.2 (s, *C*CH_3). Peaks at 15.9 ppm and 66.3 ppm are attributed to residual diethyl ether. Peaks at 26.2 ppm and 68.2 ppm are attributed to residual tetrahydrofuran.

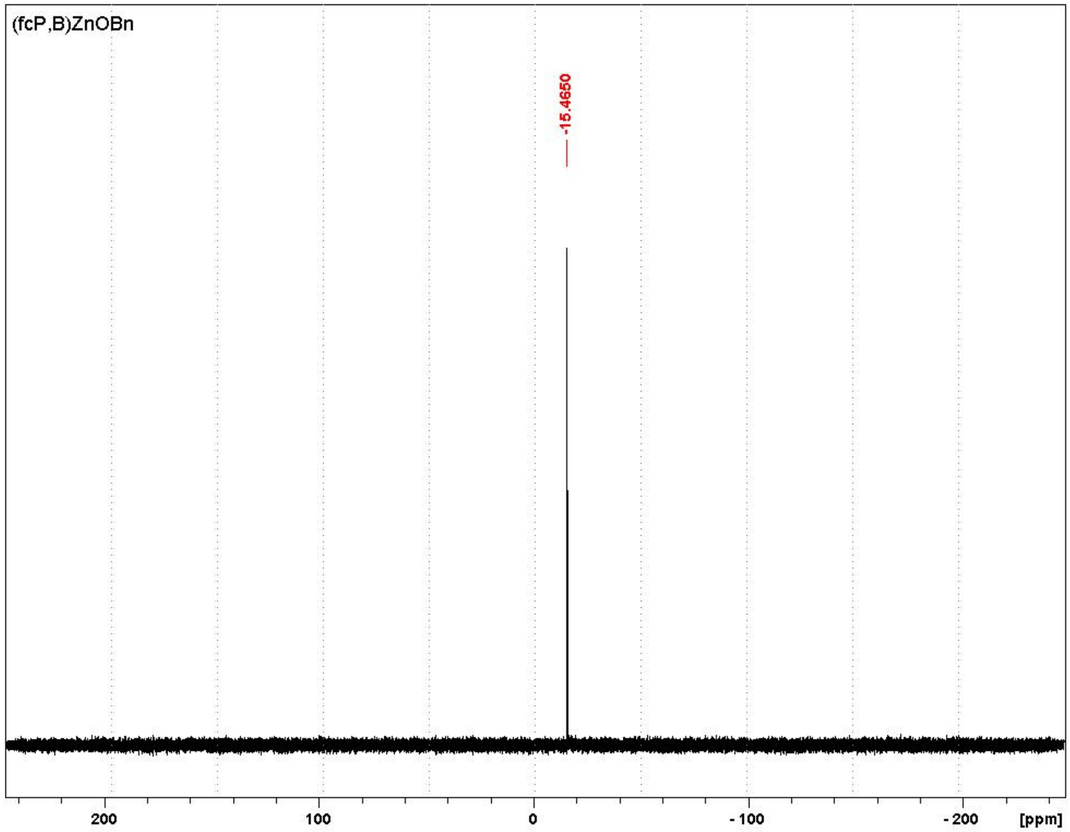

Figure S3. $^{31}P\{^1H\}$ NMR spectrum (C_6D_6, 203 MHz, 298 K) of $[(fc^{P,B})Zn(\mu\text{-}OCH_2Ph)]_2$: δ (ppm) -15.5 (s).

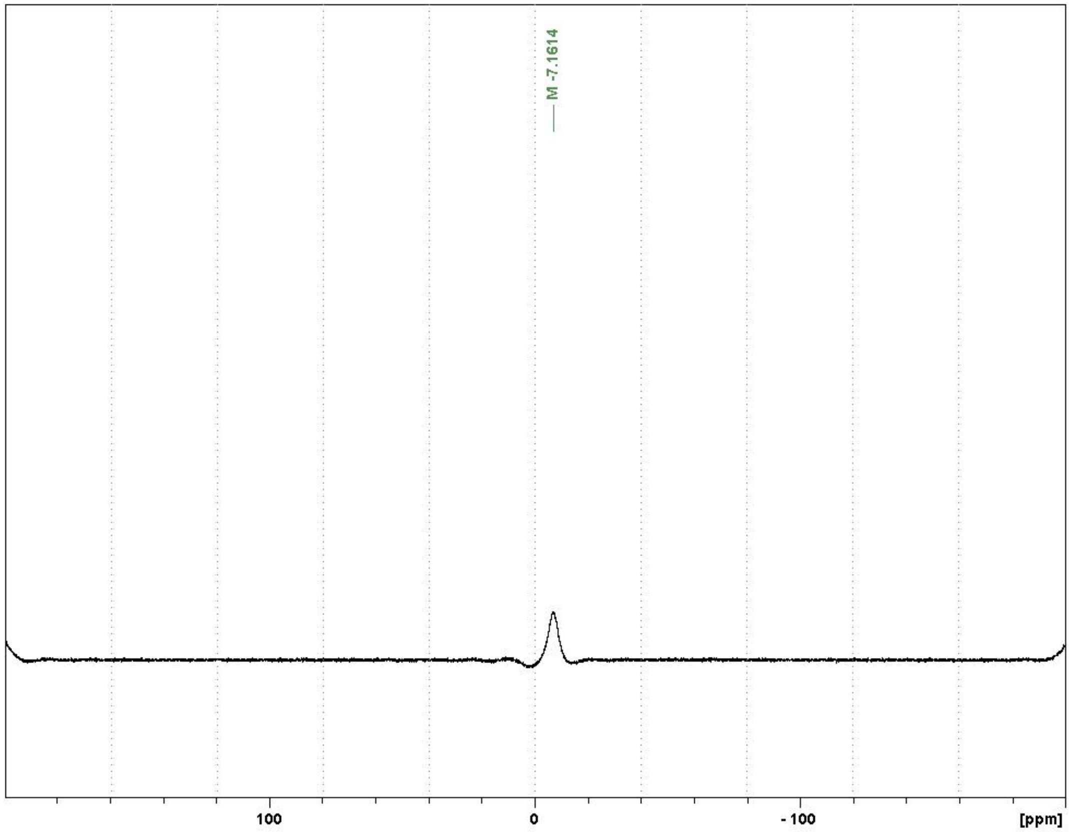

Figure S4. ^{11}B NMR spectrum (C_6D_6, 161 MHz, 298 K) of $[(fc^{P,B})Zn(\mu\text{-}OCH_2Ph)]_2$: δ (ppm) -7.2 (br s).

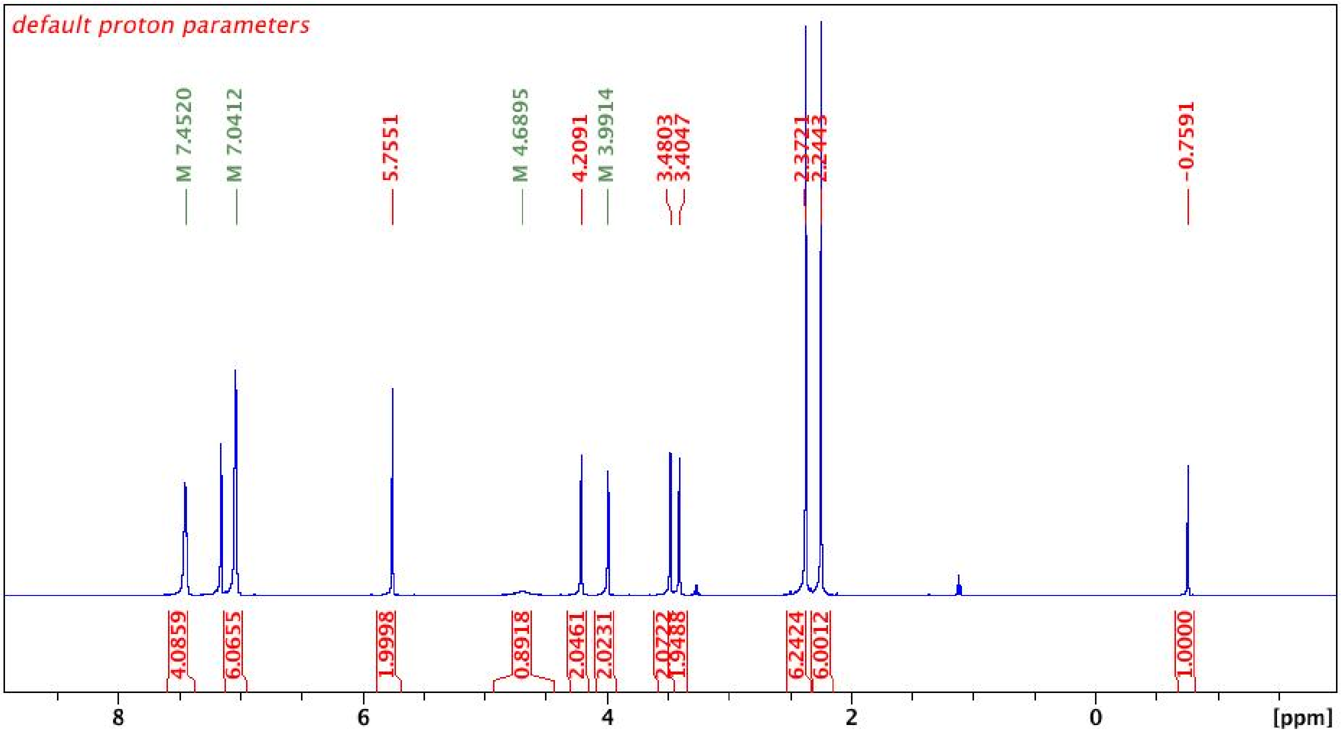

Figure S5. ^{1}H NMR spectrum (C_6D_6, 500 MHz, 298 K) of [(fc^{P,B})Zn(μ-OH)]$_2$: δ (ppm) -0.76 (s, 1H, O*H*), 2.24 (s, 6H, C*H*$_3$), 2.37 (s, 6H, C*H*$_3$), 3.40 (t, 2H, Cp-*H*), 3.48 (t, 2H, Cp-*H*), 3.99 (q, 2H, Cp-*H*), 4.21 (t, 2H, Cp-*H*), 4.69 (br s, 1H, B*H*), 5.76 (s, 2H, C*H*), 7.04 (m, 6H, *m*-Ph, *p*-Ph), 7.45 (m, 4H, *o*-Ph).

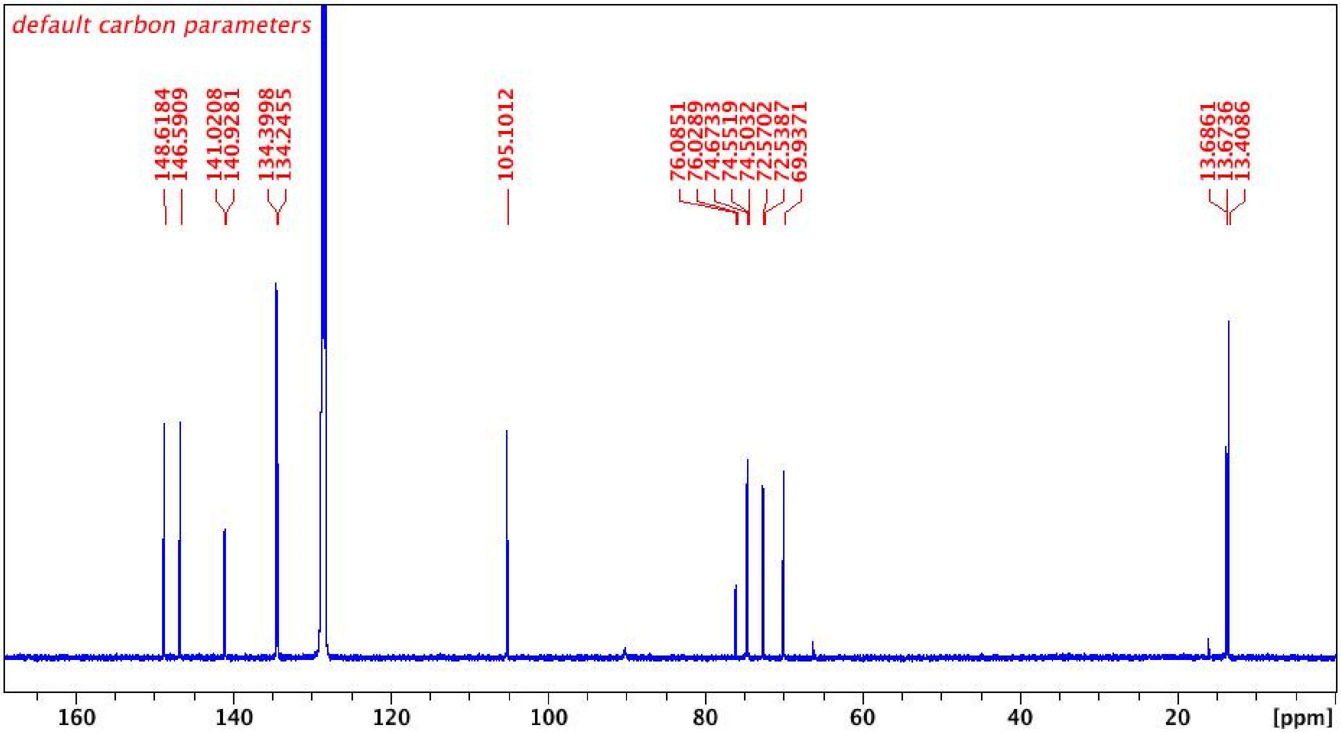

Figure S6: ^{13}C NMR spectrum (C_6D_6, 126 MHz, 298 K) of [(fc^{P,B})Zn(μ-OH)]$_2$: δ (ppm) 13.4 (s, *C*H$_3$), 13.7 (d, *C*H$_3$), 69.9 (s, Cp-*C*), 72.6 (d, Cp-*C*), 74.5 (s, Cp-*C*), 74.6 (d, Cp-*C*), 76.0 (d, Cp-*C*), 105.1 (s, *C*H), 134.3 (d, aromatic), 140.1 (d, aromatic), 146.6 (s, *C*CH$_3$), 148.6 (s, *C*CH$_3$).

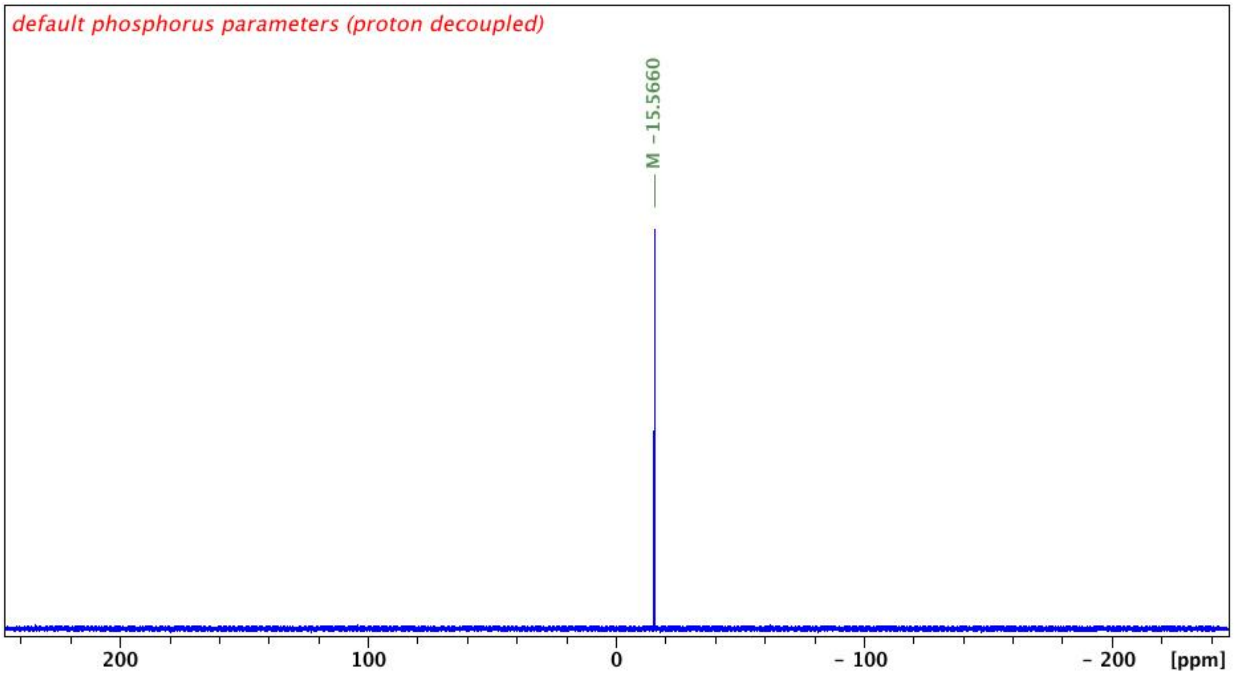

Figure S7. $^{31}P\{^{1}H\}$ NMR spectrum (C_6D_6, 203 MHz, 298 K) of $[(fc^{P,B})Zn(\mu\text{-}OH)]_2$: δ (ppm) -15.6 (s).

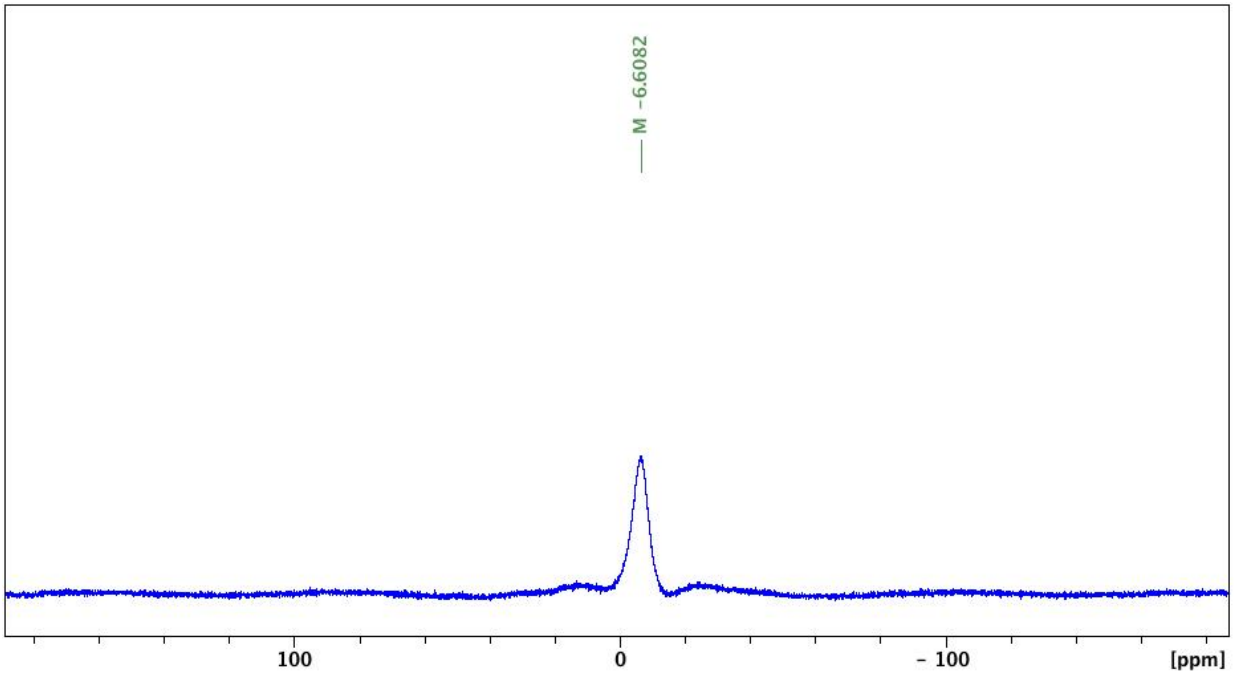

Figure S8. ^{11}B NMR spectrum (C_6D_6, 161 MHz, 298 K) of $[(fc^{P,B})Zn(\mu\text{-}OH)]_2$: δ (ppm) -6.6 (br s).

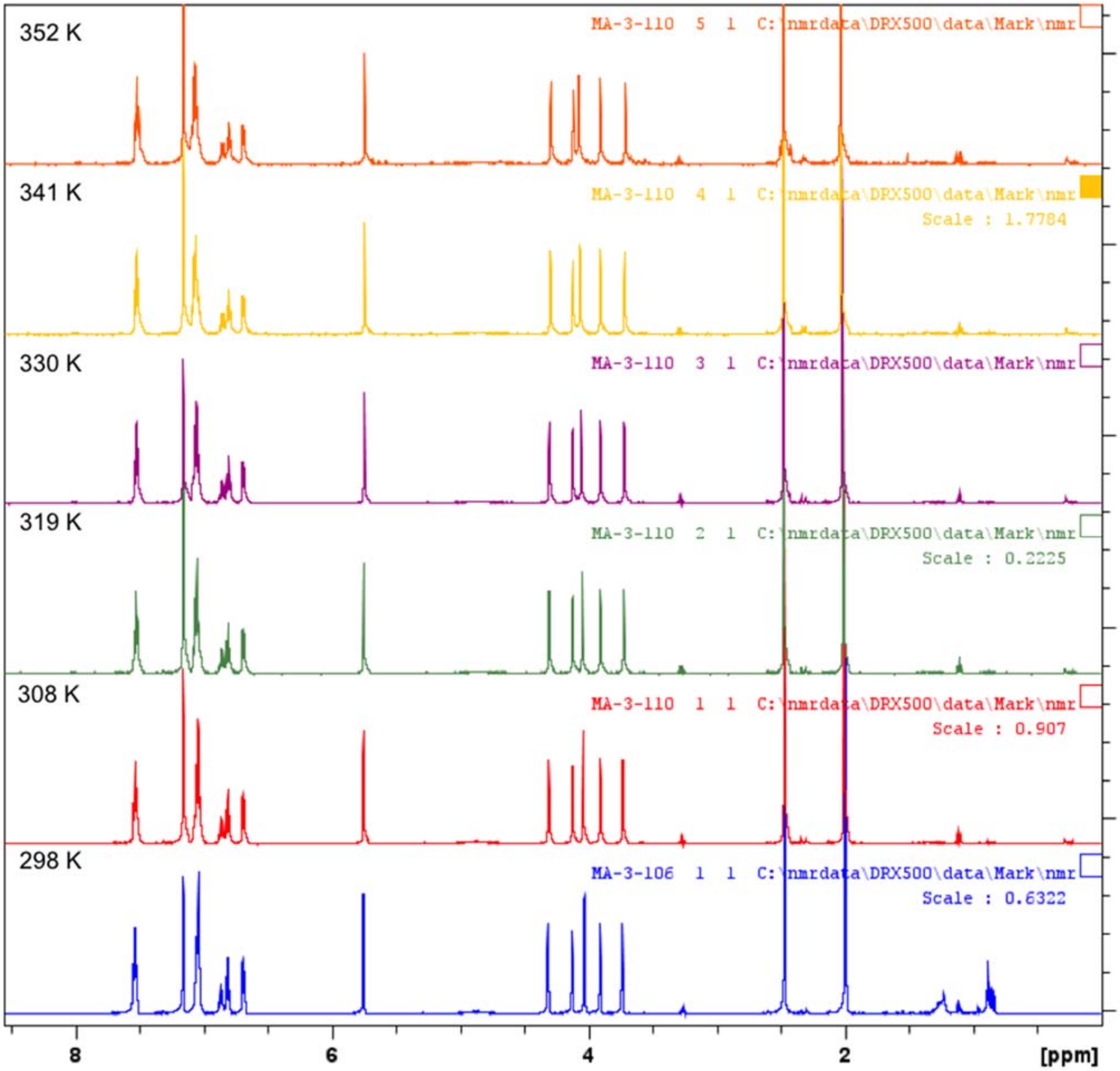

Figure S9. Variable temperature NMR (C_6D_6, 500 MHz) study of $[(fc^{P,B})Zn(\mu\text{-}OCH_2Ph)]_2$.

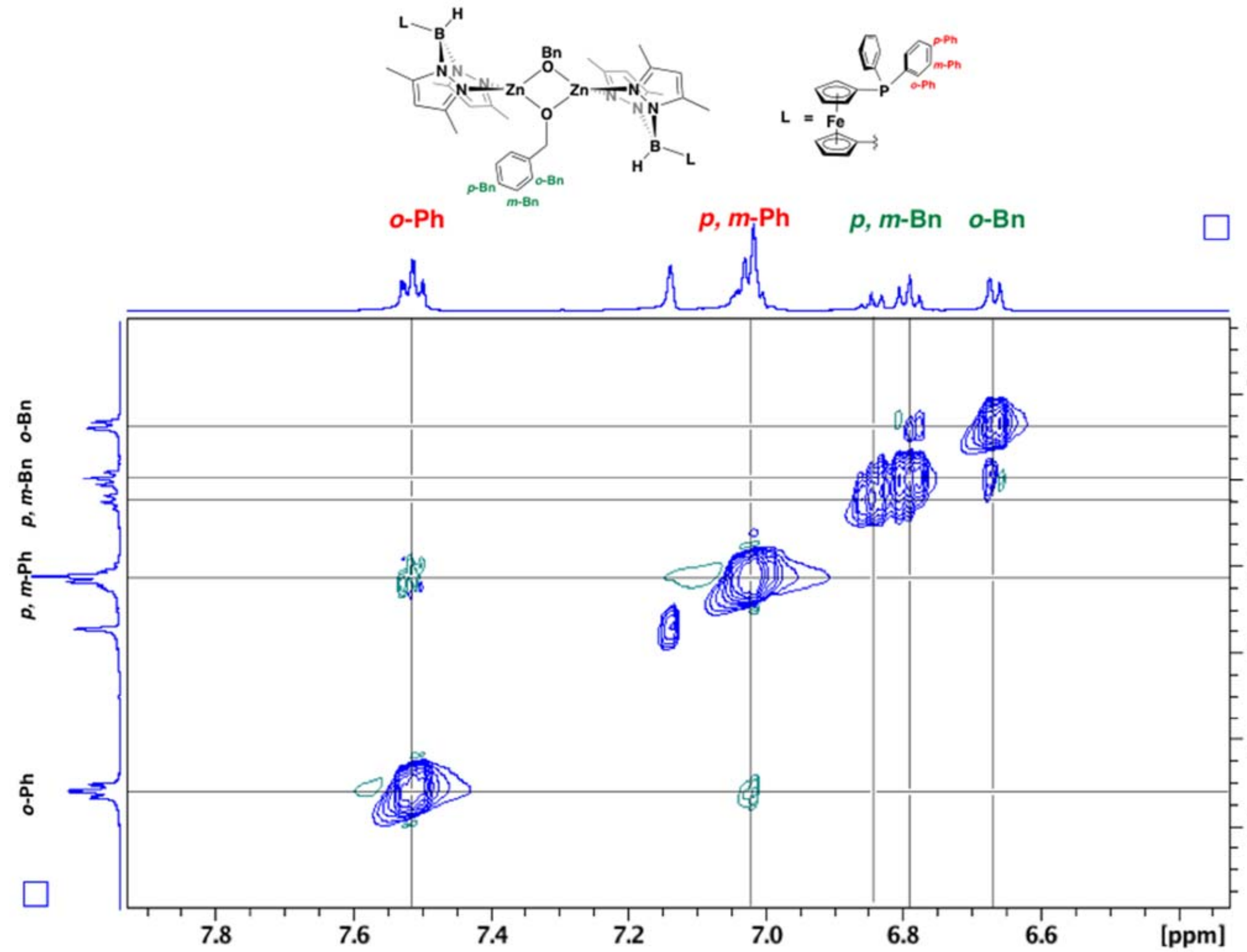

Figure S10. Selected region of ^{1}H NOESY NMR spectrum (C_6D_6, 500 MHz, 298 K) of $[(fc^{P,B})Zn(\mu\text{-}OCH_2Ph)]_2$.

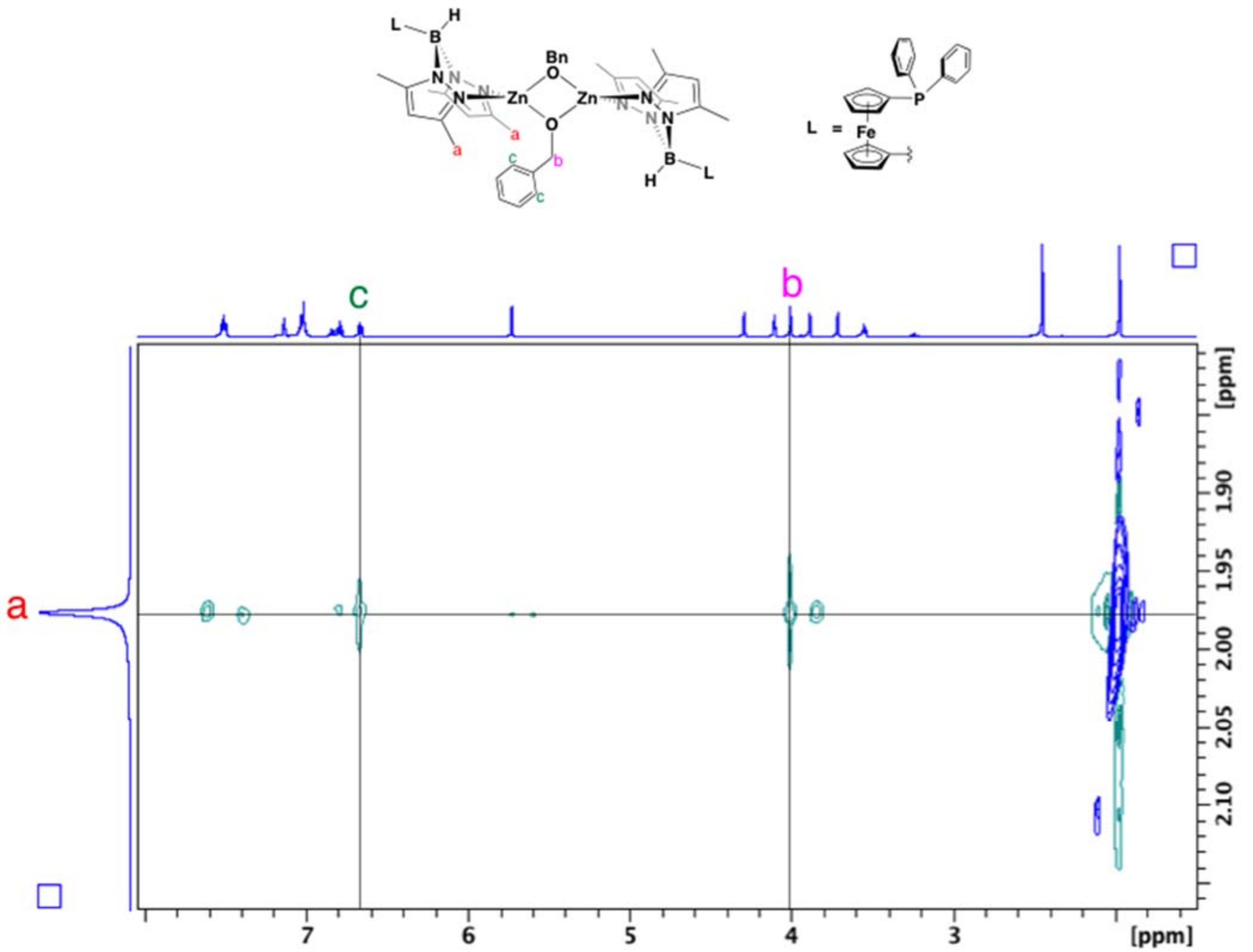

Figure S11. Selected region of ^{1}H NOESY NMR spectrum (C_6D_6, 500 MHz, 298 K) of $[(fc^{P,B})Zn(\mu\text{-}OCH_2Ph)]_2$.

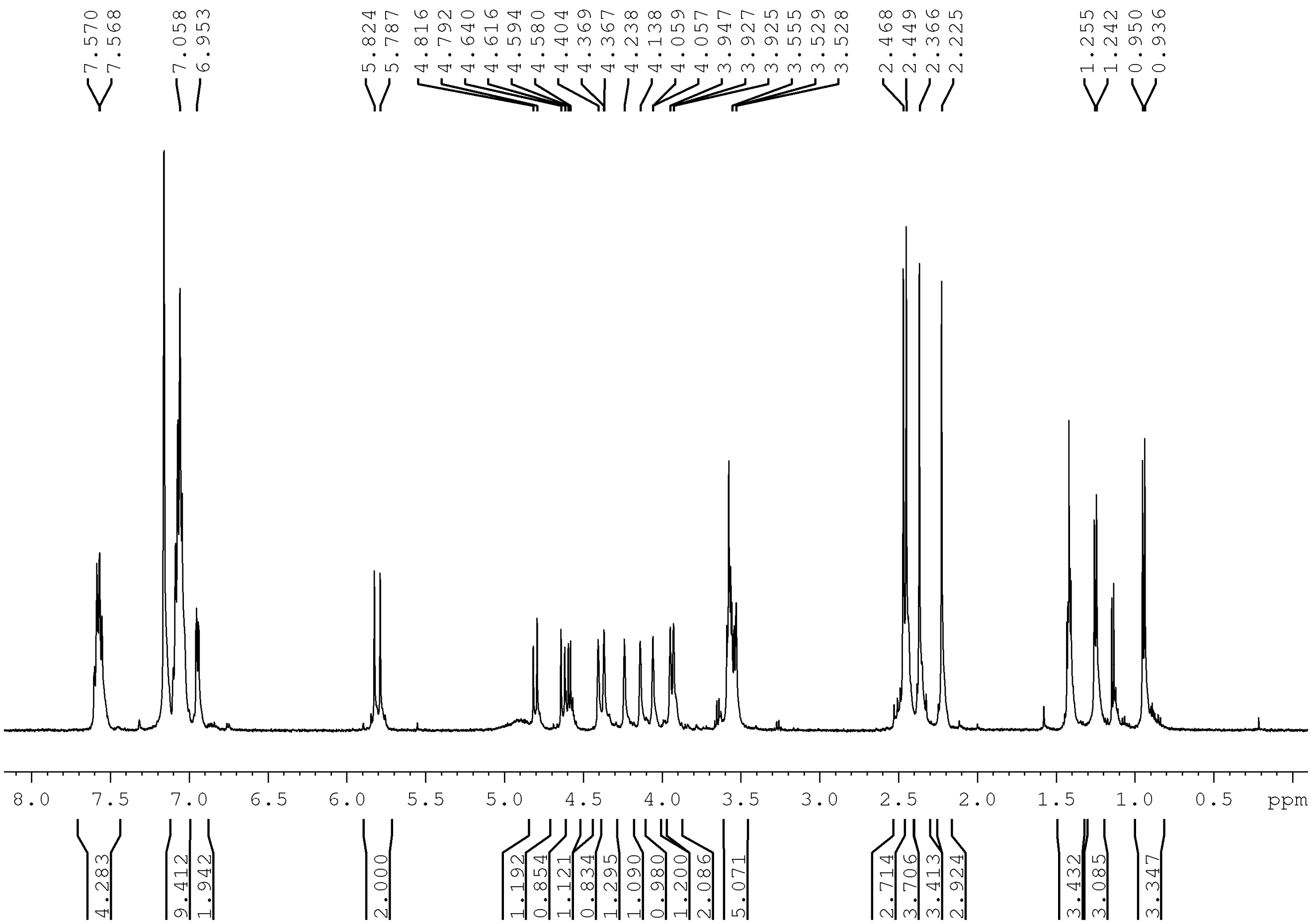

Figure S12. NMR scale reaction (C_6D_6, 500 MHz, 298 K) of [(fc^{P,B})Zn(μ-OCH_2Ph)]$_2$ and L-lactide: δ (ppm) 0.94 (d, 3H, C*H*3(LA)), 1.25 (d, 3H, C*H*3(LA)), 2.22 (s, 3H, C*H*3(pz)), 2.37 (s, 3H, C*H*3(pz)), 2.45 (s, 3H, C*H*3(pz)), 2.47 (s, 3H, C*H*3(pz)), 3.53 (q, 1H, C*H*(LA)), 3.93 (s, 1H, Cp-*H*), 3.95 (s, 1H, Cp-*H*), 4.06 (s, 1H, Cp-*H*), 4.14 (s, 1H, Cp-*H*), 4.24 (s, 1H, Cp-*H*), 4.37 (s, 1H, Cp-*H*), 4.40 (s, 1H, Cp-*H*), 4.60 (q, 1H, C*H*(LA)), 4.72 (q, 2H, OC*H*2Ph), 5.79 (s, 1H, C*H*(pz)), 5.82 (s, 1H, C*H*(pz)), 6.95 (m, 2H, *o*-Bn), 7.06 (m, 9H, *m*-Bn, *p*-Bn, *m*-Ph, *p*-Ph), 7.57 (m, 4H, *o*-Ph).

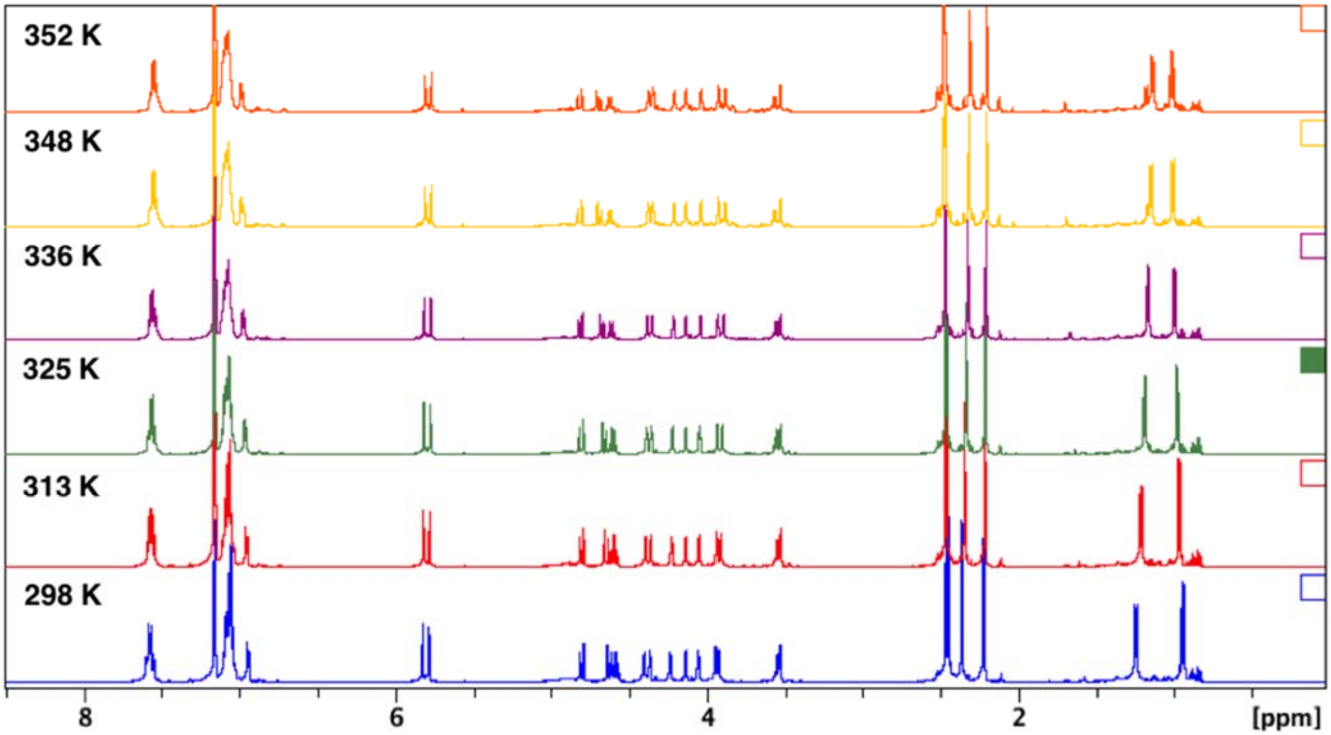

Figures S13. Variable temperature NMR (C_6D_6, 500 MHz) study of $[(fc^{P,B})Zn(LA)(OCH_2Ph)]_2$.

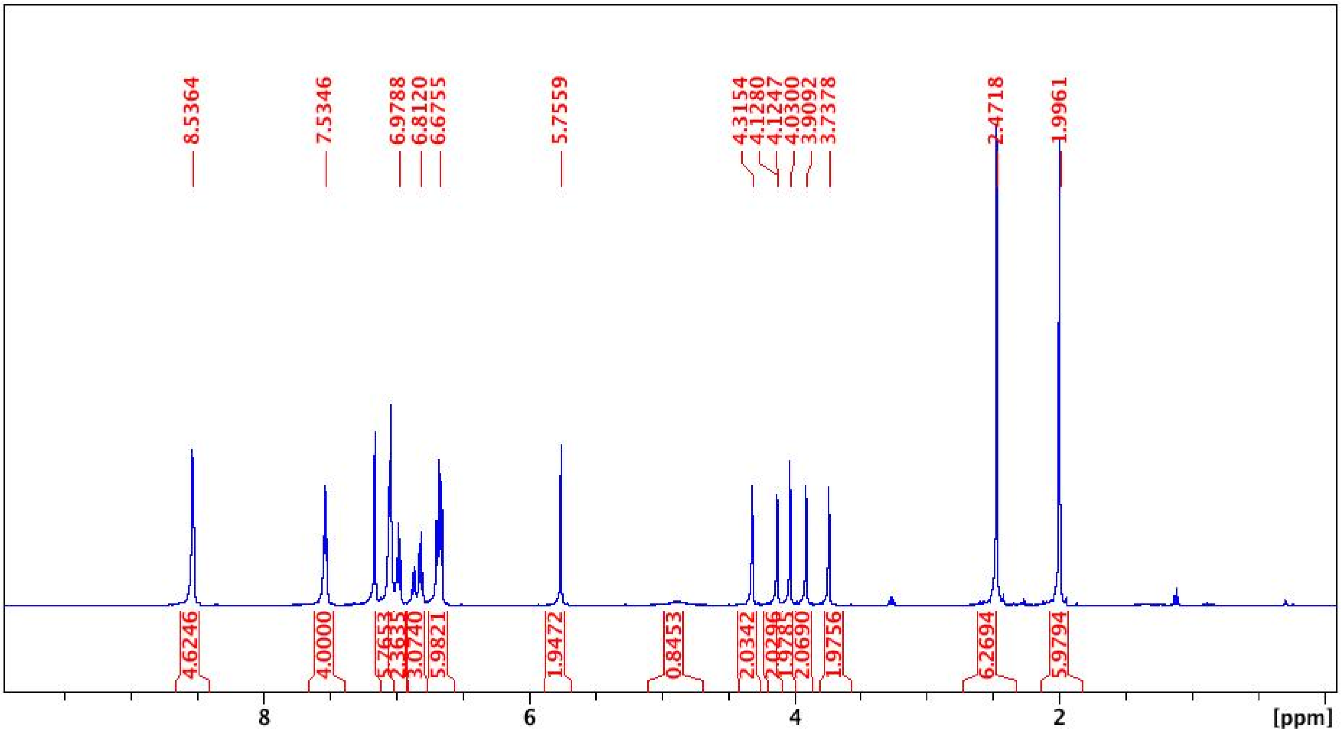

Figure S14. ^1H NMR spectrum (C_6D_6, 500 MHz, 298 K) of $[(fc^{P,B})Zn(\mu\text{-}OCH_2Ph)]_2$ in the presence of 5 equivalent of pyridine. No change in the signals corresponding to $[(fc^{P,B})Zn(\mu\text{-}OCH_2Ph)]_2$ is observed.

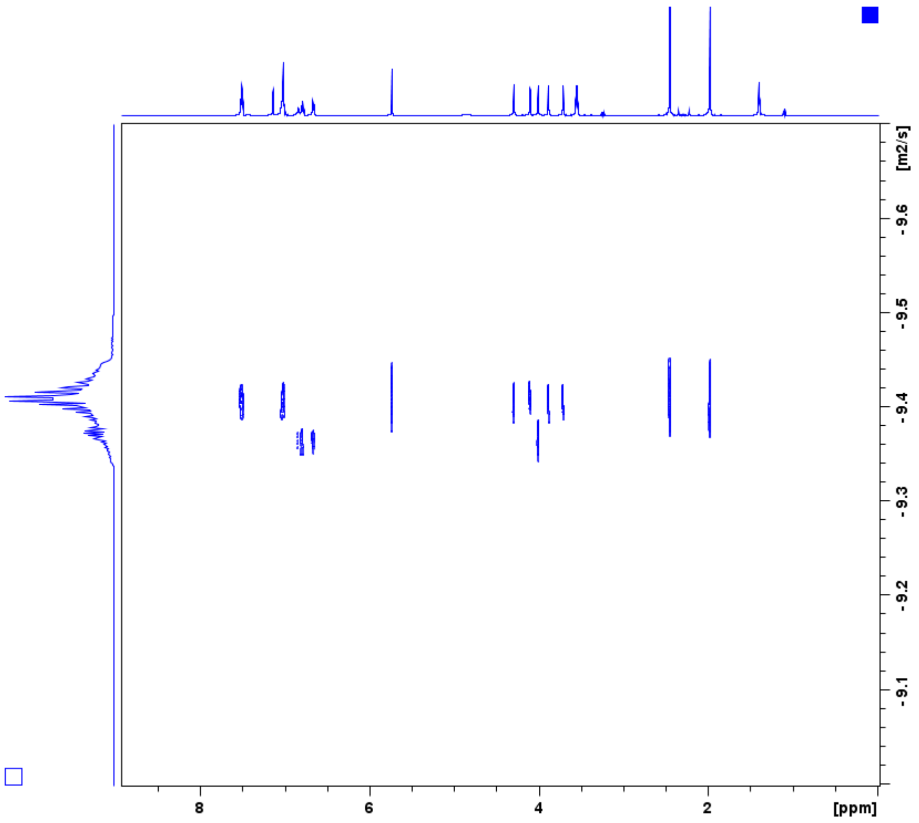

Figure S15. ^{1}H DOSY NMR spectrum (C_6D_6, 500 MHz, 298 K) of $[(fc^{P,B})Zn(\mu\text{-}OCH_2Ph)]_2$. A minor difference in the diffusion rates of the –OCH_2Ph component vs. the heteroscorpionate supporting ligand is observed. Such drifts in the signals corresponding to the same molecule can be found in various DOSY spectra in the literature.[1]

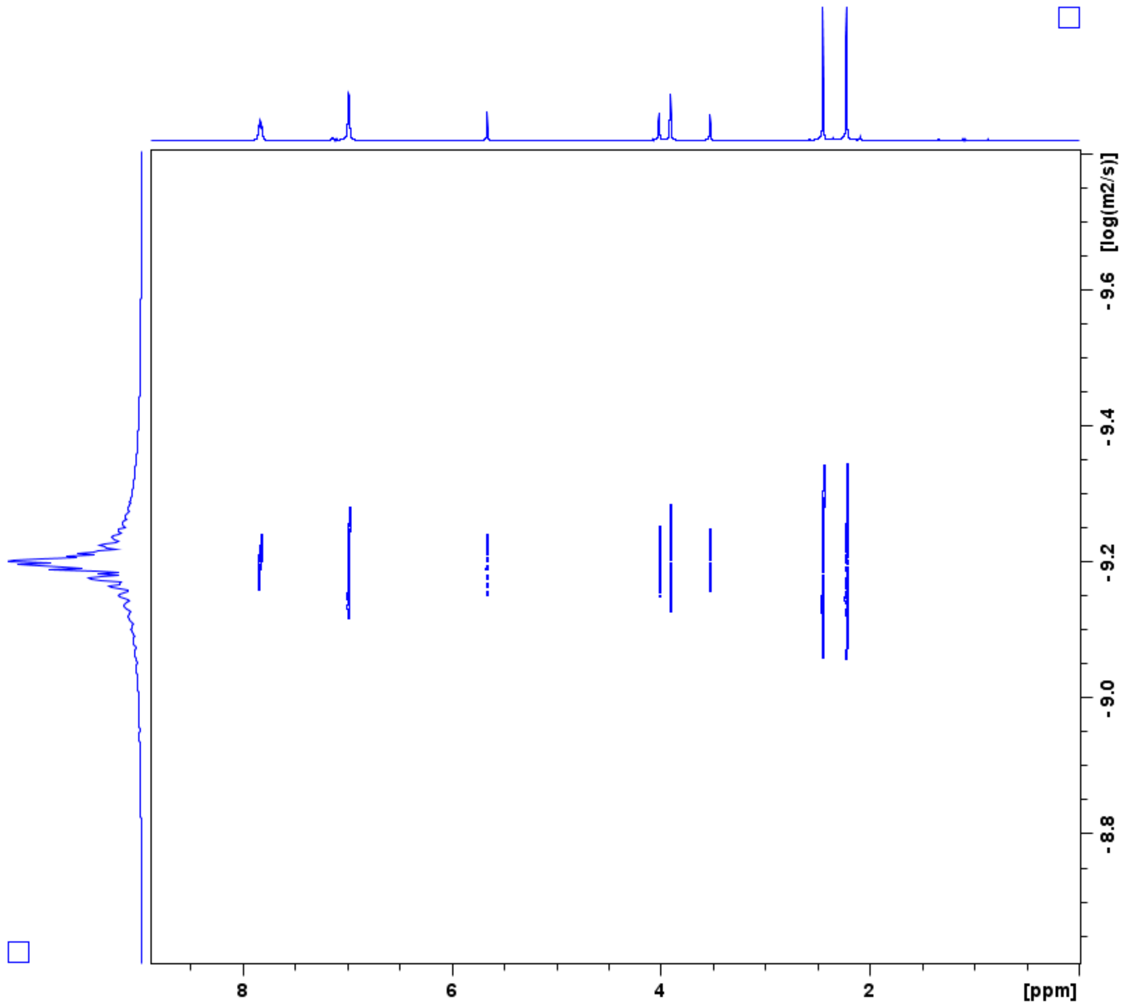

Figure S16. ^{1}H DOSY NMR spectrum (C_6D_6, 500 MHz, 298 K) of $(fc^{P,B})ZnCl$.

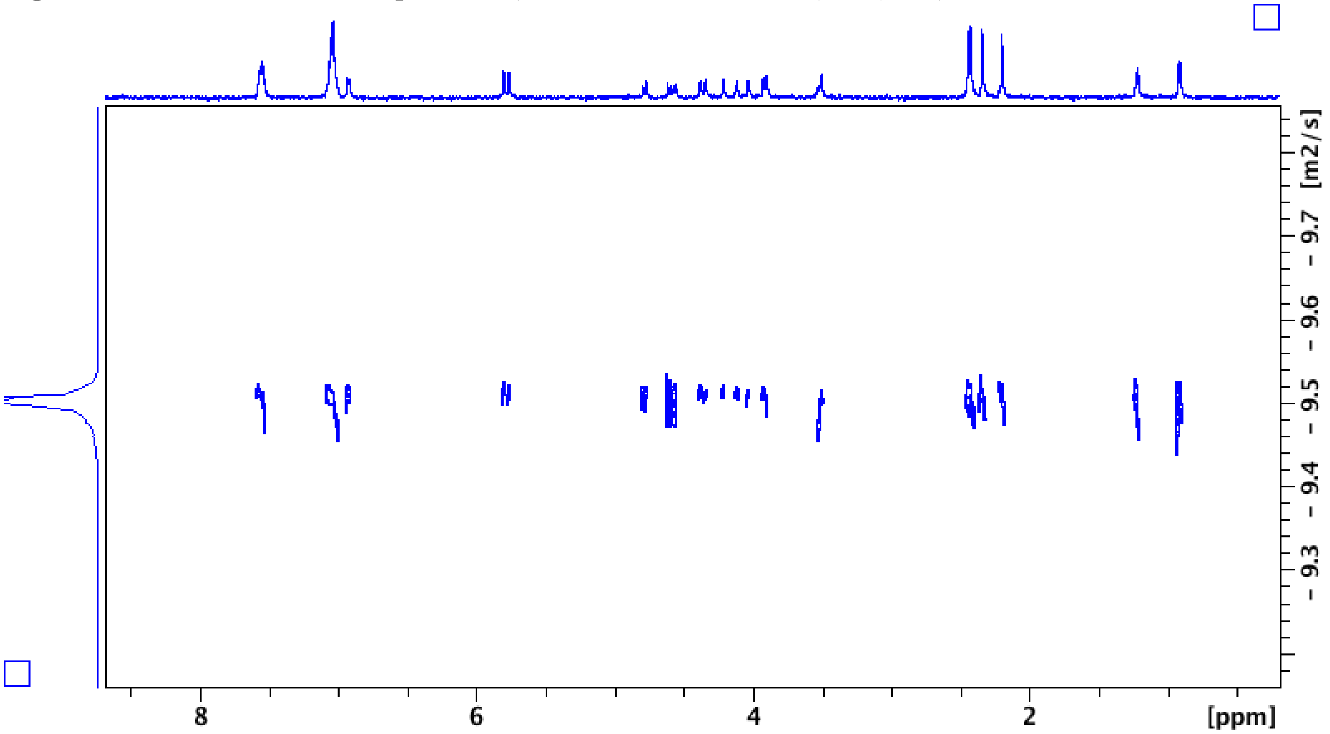

Figure S17. ^{1}H DOSY NMR spectrum (C_6D_6, 500 MHz, 298 K) of $[(fc^{P,B})Zn(LA)(OCH_2Ph)]_2$.

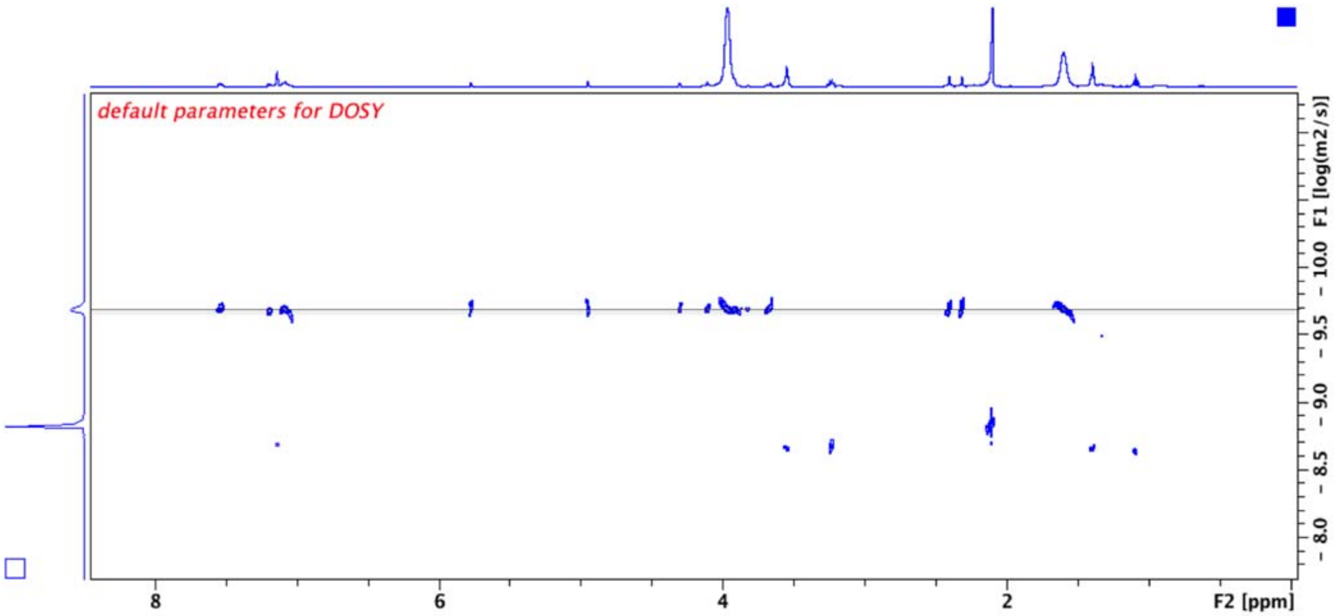

Figure S18. ^{1}H DOSY NMR spectrum (C_6D_6, 500 MHz, 298 K) of [(fc^{P,B})Zn(PTMC)$_{36}$(OCH$_2$Ph)]$_2$. Signals at 4.94 ppm, 7.07 ppm and 7.18 ppm correspond to the -OCH$_2$Ph end group. Signals not on the line are attributed to residual monomer, THF, hexamethylbenzene, and C_6D_6.

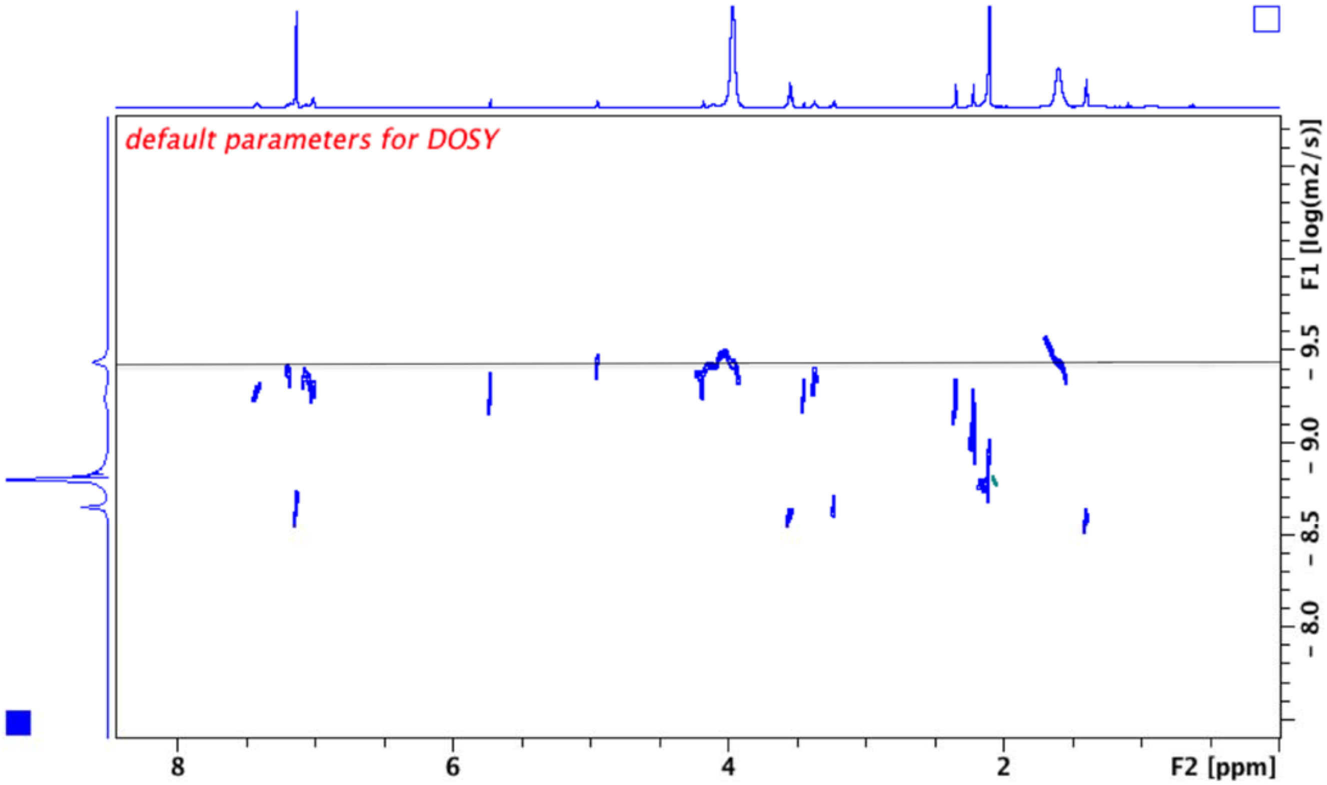

Figure S19. ^{1}H DOSY NMR spectrum (C_6D_6, 500 MHz, 298 K) of the PhCH$_2$O(PTMC)$_{36}$H polymer. Signals at 4.94 ppm, 7.07 ppm and 7.18 ppm correspond to the -OCH$_2$Ph end group. Signals not on the line are attributed to hydrolyzed catalyst, residual monomer, THF, hexamethylbenzene, and C_6D_6.

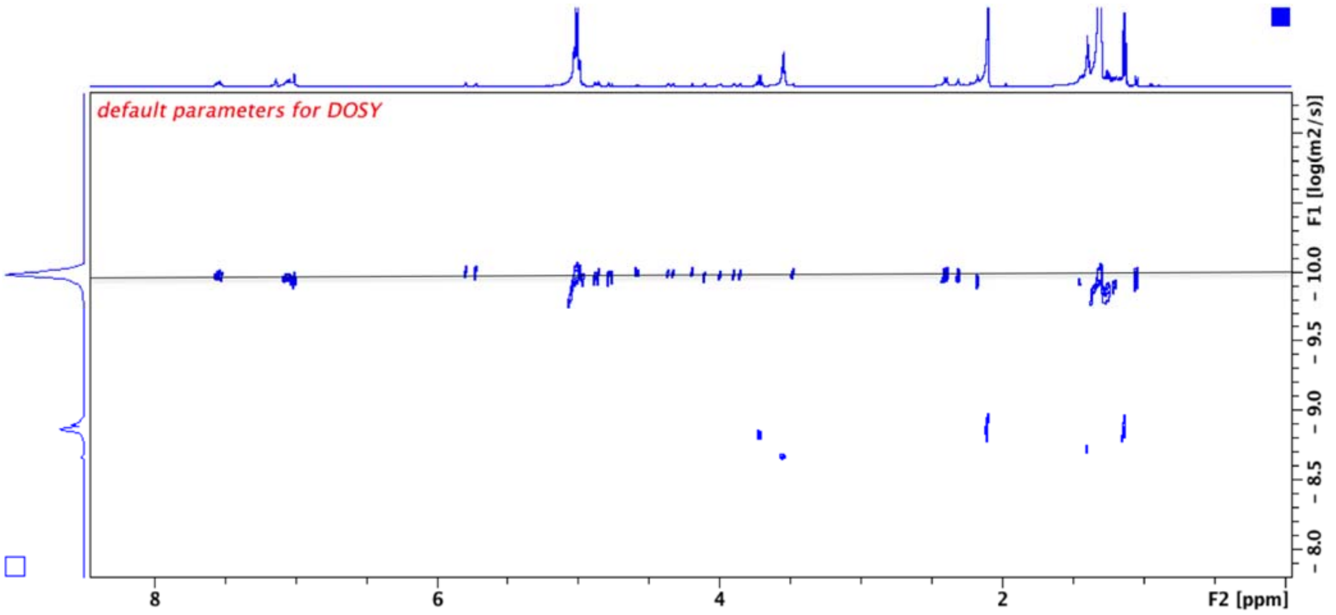

Figure S20. ^{1}H DOSY NMR spectrum (C_6D_6, 500 MHz, 298 K) of $[(fc^{P,B})Zn(PLA)_{36}(OCH_2Ph)]_2$. Signals at 4.72 ppm, 6.95 ppm and 7.06 ppm correspond to the - OCH_2Ph end group. Signals not on the line are attributed to residual monomer, THF, hexamethylbenzene, and C_6D_6.

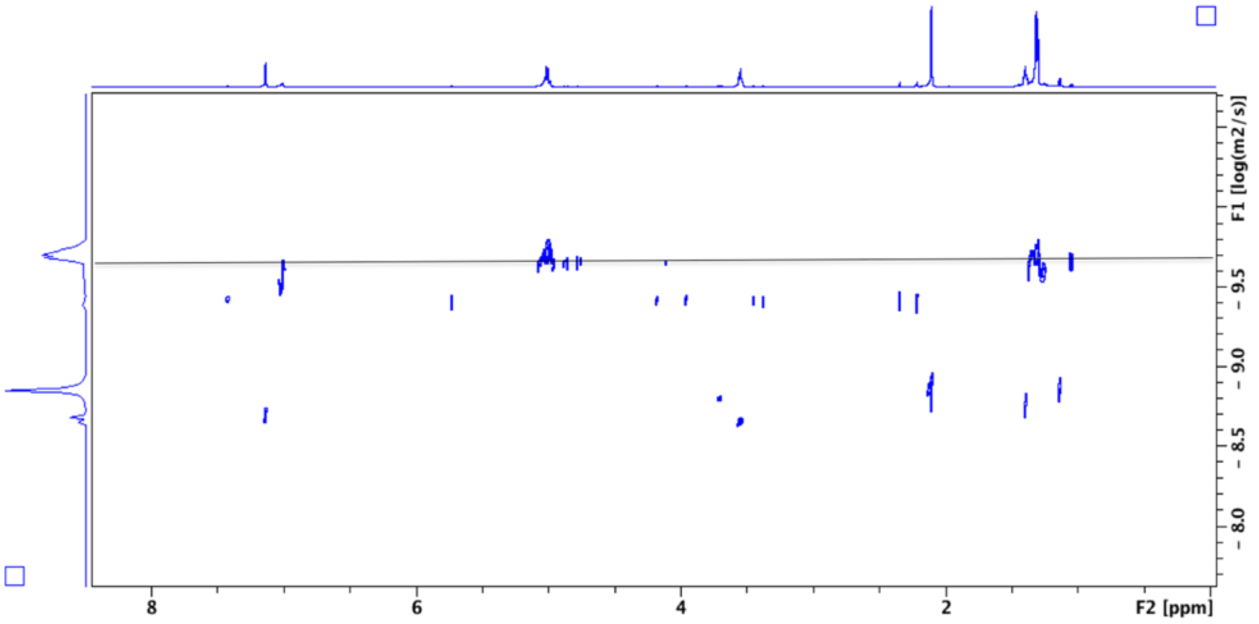

Figure S21. ^{1}H DOSY NMR spectrum (C_6D_6, 500 MHz, 298 K) of $PhCH_2O(PLA)_{36}H$. Signals at 4.72 ppm, 6.95 ppm and 7.06 ppm correspond to the $-OCH_2Ph$ end group. Signals not on the line are attributed to hydrolyzed catalyst, residual monomer, THF, hexamethylbenzene, and C_6D_6.

From the DOSY NMR spectra the following the diffusion coefficients were determined:

$D[(fc^{P,B})ZnCl] = 6.38 \times 10^{-10}$ m^2/s

$D = 3.92 \times 10^{-10}$ m^2/s

$D\{[(fc^{P,B})Zn(LA)(OCH_2Ph)]_2\} = 3.16 \times 10^{-10}$ m^2/s

$D\{[(fc^{P,B})Zn(PTMC)_{36}(OCH_2Ph)]_2\} = 2.07 \times 10^{-10}$ m^2/s

D[PhCH$_2$O(PTMC)$_{36}$H] = 3.80 × 10^{-10} m^2/s

D{[(fc^{P,B})Zn(PLA)$_{36}$(OCH$_2$Ph)]$_2$} = 1.04 × 10^{-10} m^2/s

D[PhCH$_2$O(PLA)$_{36}$H] = 2.00 × 10^{-10} m^2/s

The Stokes-Einstein equation shows the relationship between the hydrodynamic radius and the diffusion coefficient:

$D = (kT)/(6\pi\eta r_H)$

By comparing the diffusion coefficients of two compounds the ratios of the hydrodynamic radii can be determined.

$D_{(ZnCl)}/D_{(ZnOBn)2} = r_{(ZnOBn)2}/r_{(ZnCl)} = 1.63$

$D_{(ZnOBn)2}/D_{[Zn(LA)OBn]2} = r_{[Zn(LA)OBn]2}/r_{(ZnOBn)2} = 1.24$

$D_{(PhCH2O(PLA)36H)}/D_{[Zn(PLA)36(OCH2Ph)]2} = r_{[Zn(PLA)36(OCH2Ph)]2}/r_{(PhCH2O(PLA)36H)} = 1.92$

$D_{(PhCH2O(PTMC)36H)}/D_{[Zn(PTMC)36(OCH2Ph)]2} = r_{[Zn(PTMC)36(OCH2Ph)]2}/r_{(PhCH2O(PTMC)36H)} = 1.84$

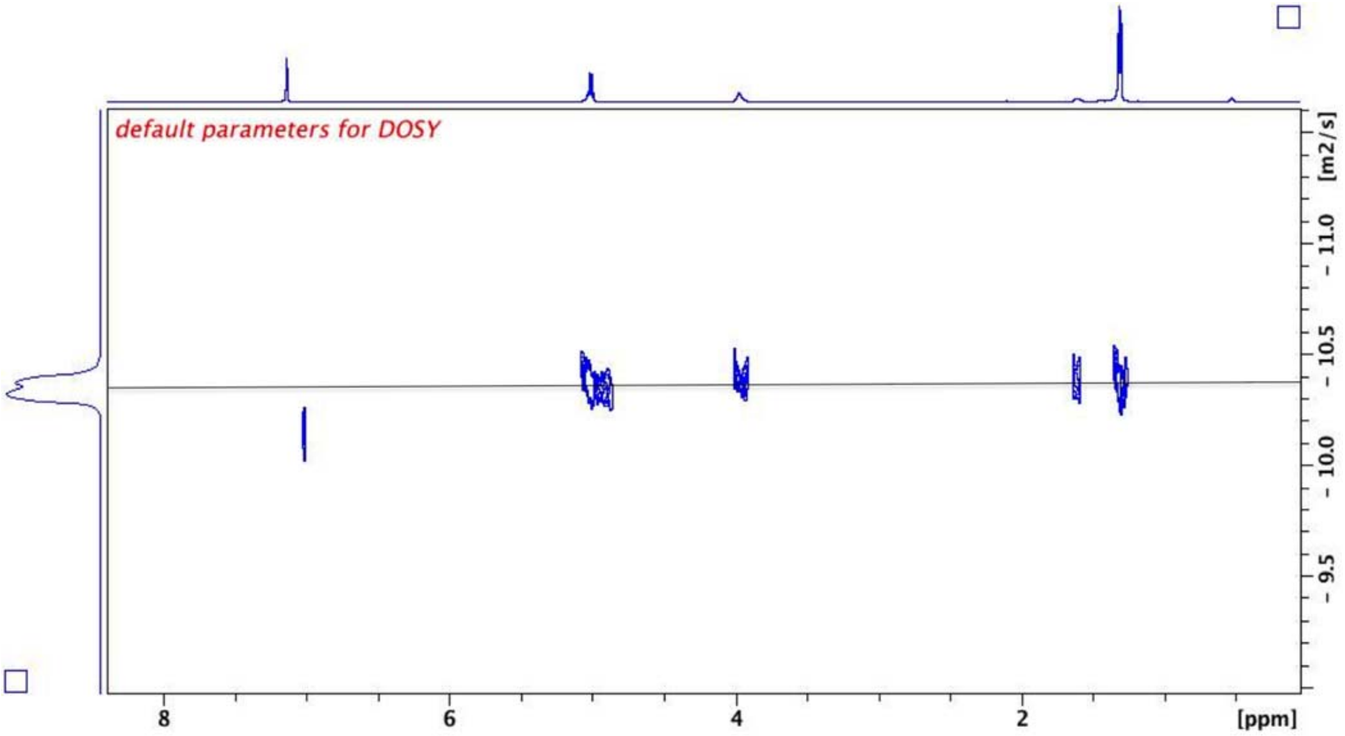

Figure S22. ^{1}H DOSY NMR spectrum (C_6D_6, 500 MHz, 298 K) of PLA-*b*-PTMC-*b*-PLA.

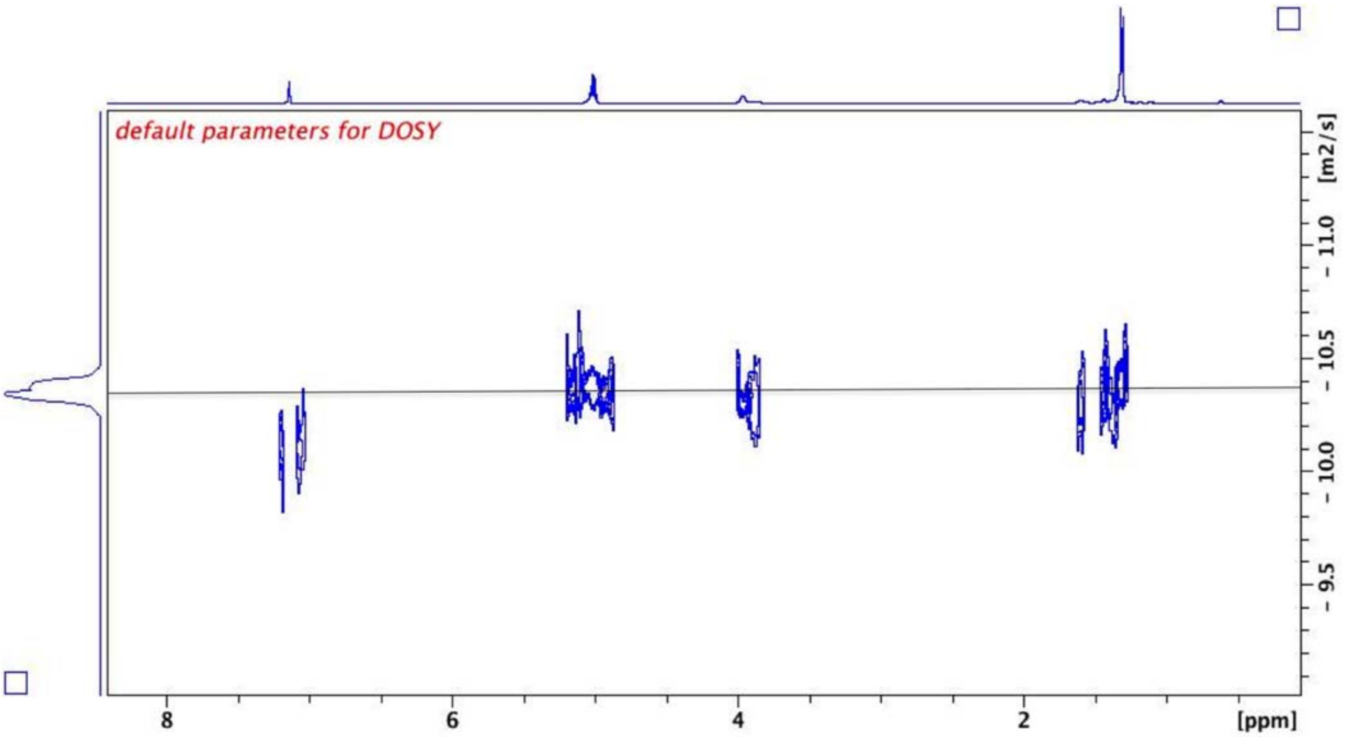

Figure S23. ^{1}H DOSY NMR spectrum (C_6D_6, 500 MHz, 298 K) of PTMC-*b*-PLA-*b*-PTMC.

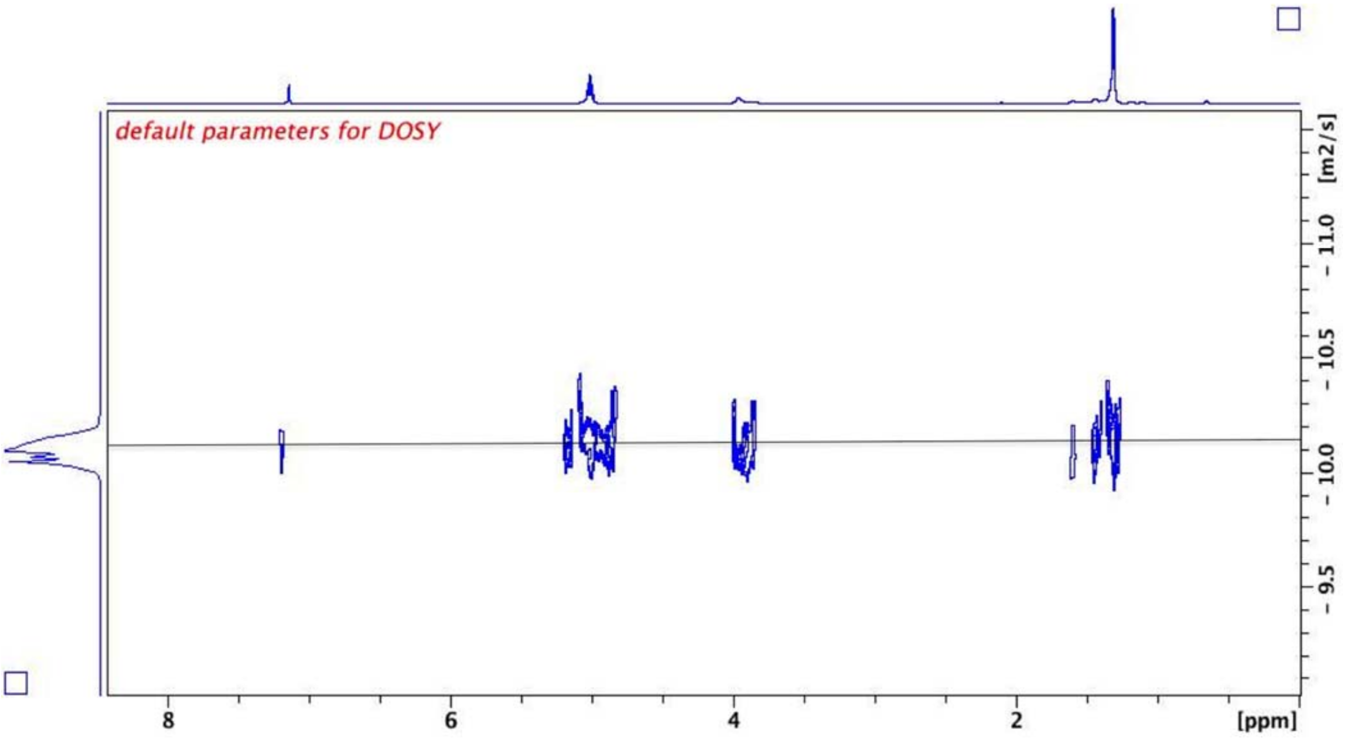

Figure S24. ^{1}H DOSY NMR spectrum (C_6D_6, 500 MHz, 298 K) of PTMC-*b*-PLA-*b*-PTMC-*b*-PLA-*b*-PTMC.

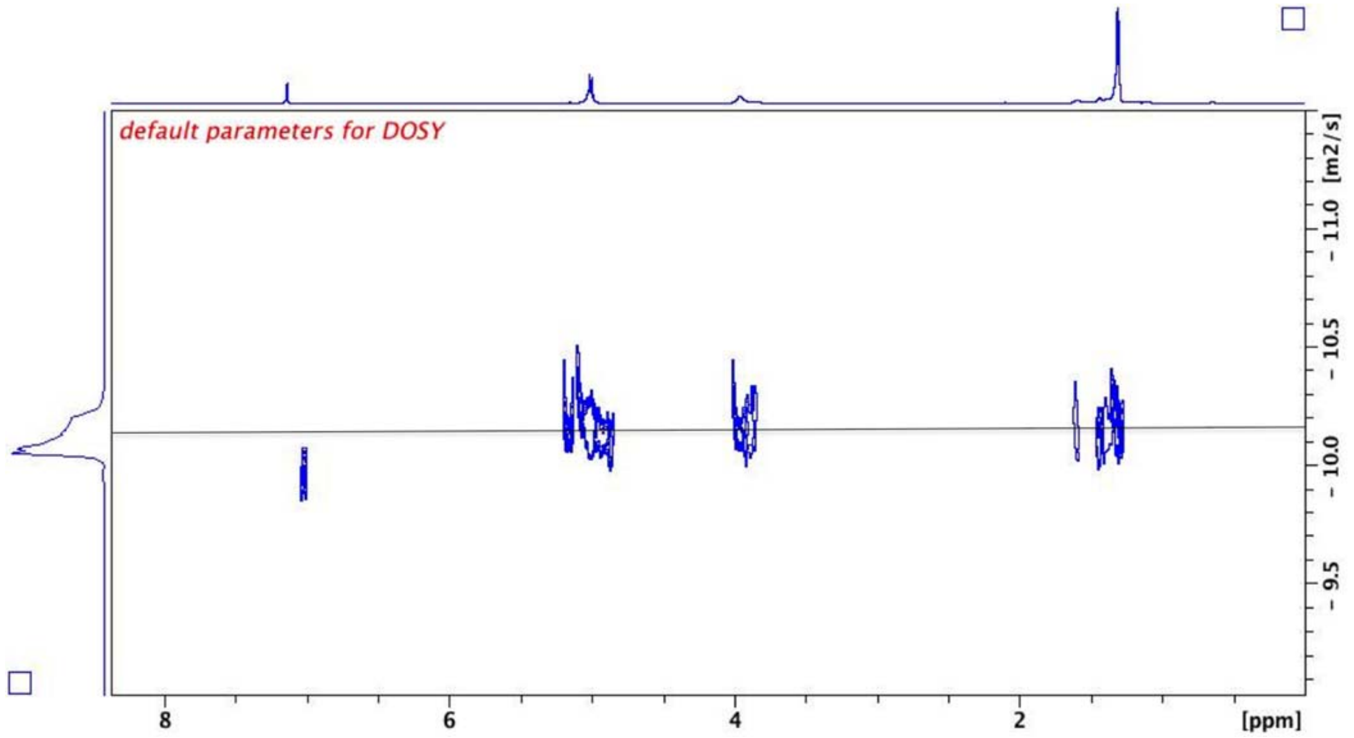

Figure S25. ^{1}H DOSY NMR spectrum (C_6D_6, 500 MHz, 298 K) of PLA-*b*-PTMC-*b*-PLA-*b*-PTMC-*b*-PLA.

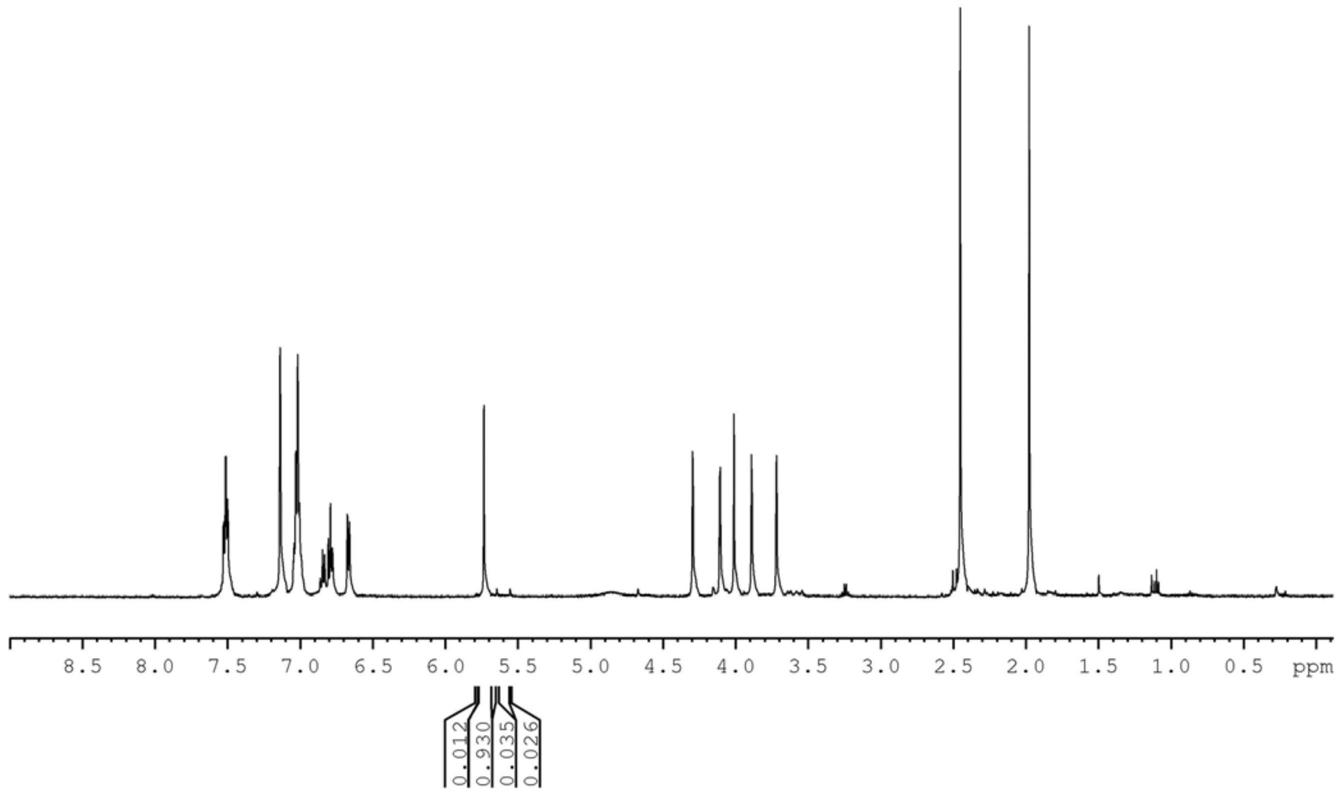

Figure S26. Thermal decomposition study (C_6D_6, 500 MHz, 298 K) of [(fc^{P,B})Zn(μ-OCH_2Ph)]$_2$, 24 h at ambient temperature. Percent decomposition is calculated based on newly formed pyrazole signals.

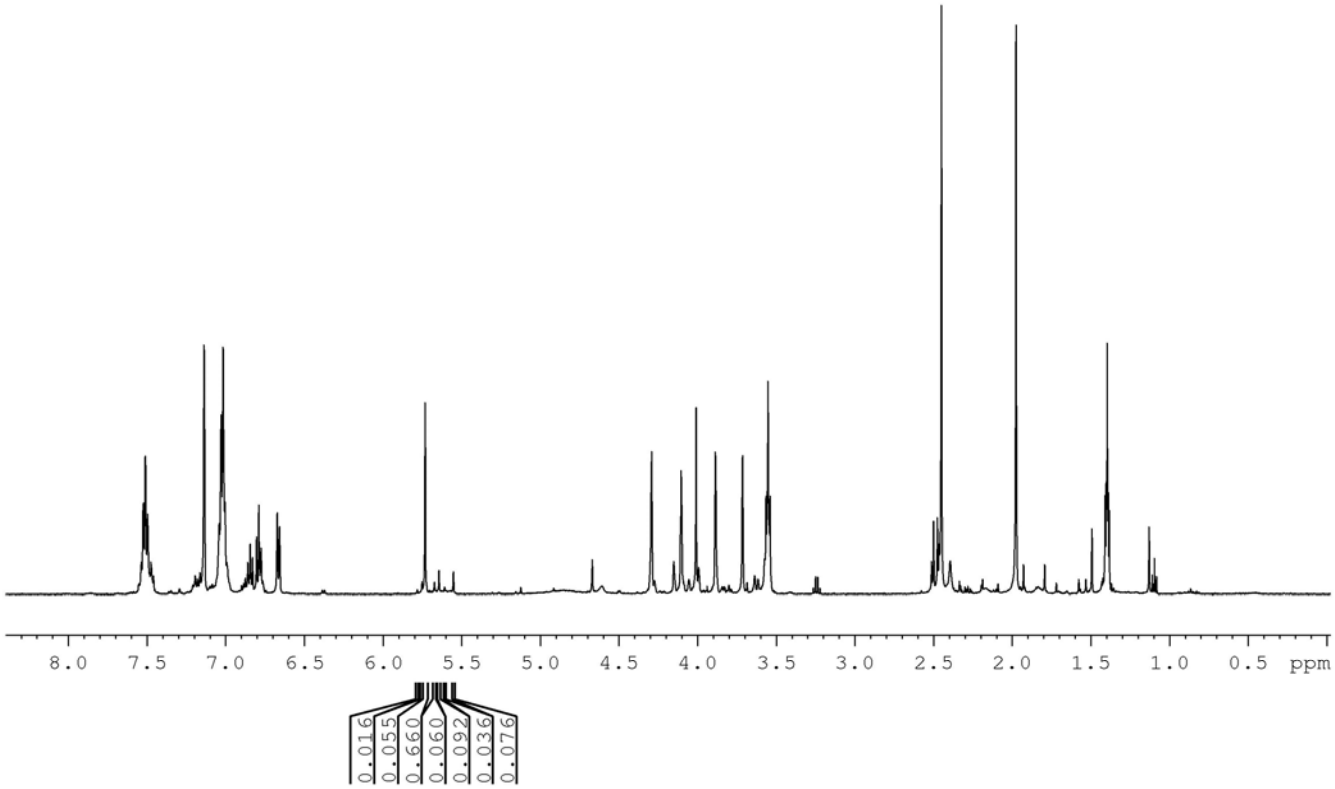

Figure S27. Thermal decomposition study (C_6D_6, 500 MHz, 298 K) of $[(fc^{P,B})Zn(\mu\text{-}OCH_2Ph)]_2$, 1.5 h at 70 °C. Percent decomposition is calculated based on newly formed pyrazole signals.

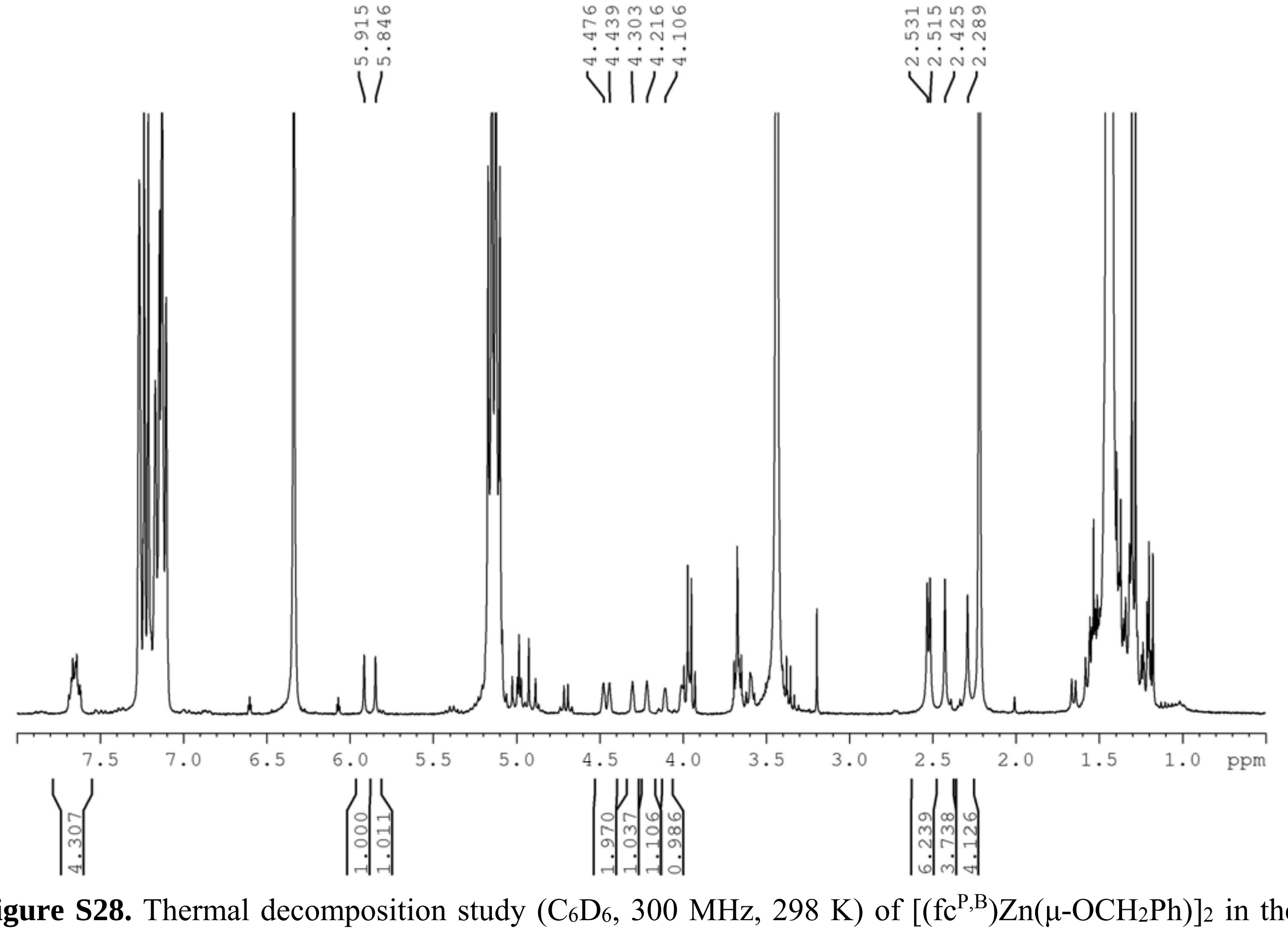

Figure S28. Thermal decomposition study (C_6D_6, 300 MHz, 298 K) of $[(fc^{P,B})Zn(\mu\text{-}OCH_2Ph)]_2$ in the presence of 100 equivalents of L-lactide, 3 h at 70 °C. No catalyst decomposition is observed.

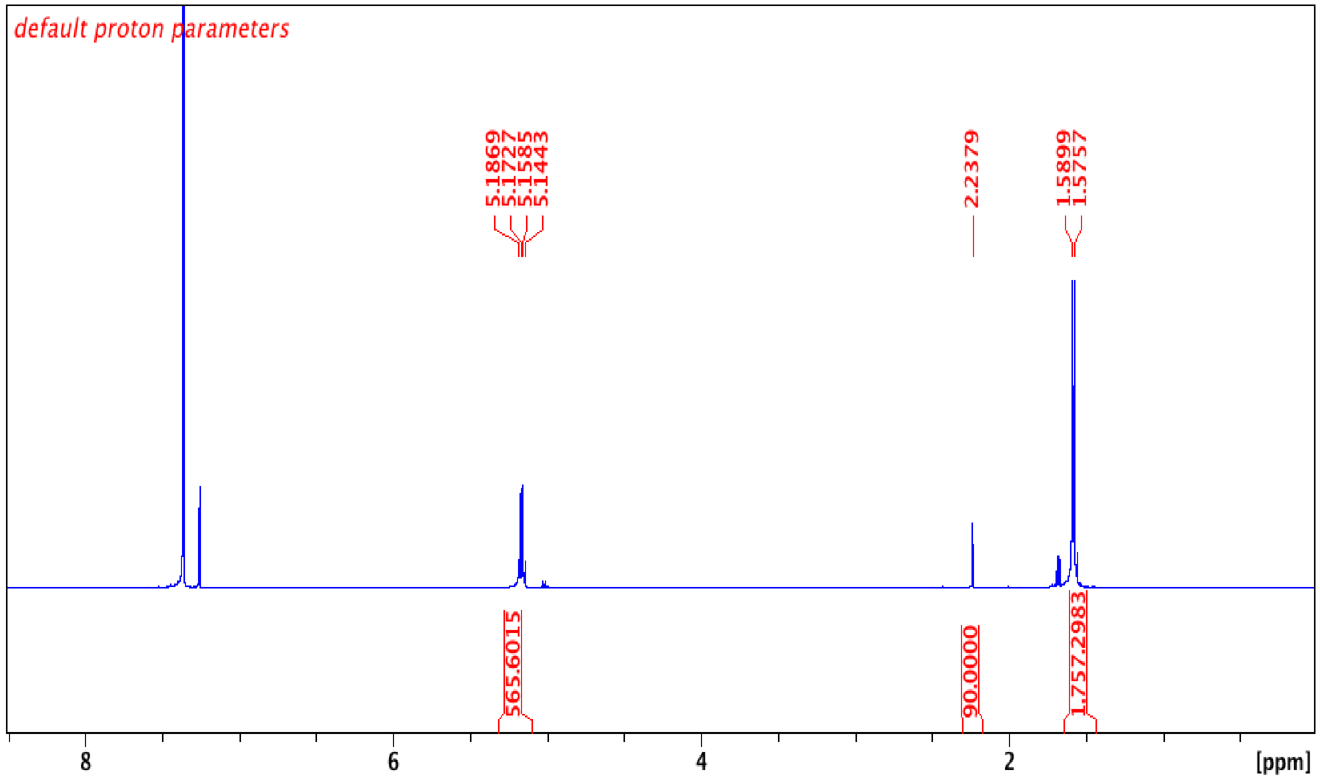

Figure S29. ^{1}H NMR spectrum ($CDCl_3$, 500 MHz, 298 K) of L-LA polymerization (Table 1, entry 1). Standard is hexamethylbenzene (HMB). [($fc^{P,B}$)Zn(μ-OCH_2Ph)]$_2$: HMB: TMC: LA ratio is 1:10:566. δ (ppm) 1.58 (d, 6H, CHC*H*$_3$ PLA), 2.23 (s, 18H, C*H*$_3$ HMB), 5.16 (q, 2H, C*H*CH_3 PLA).

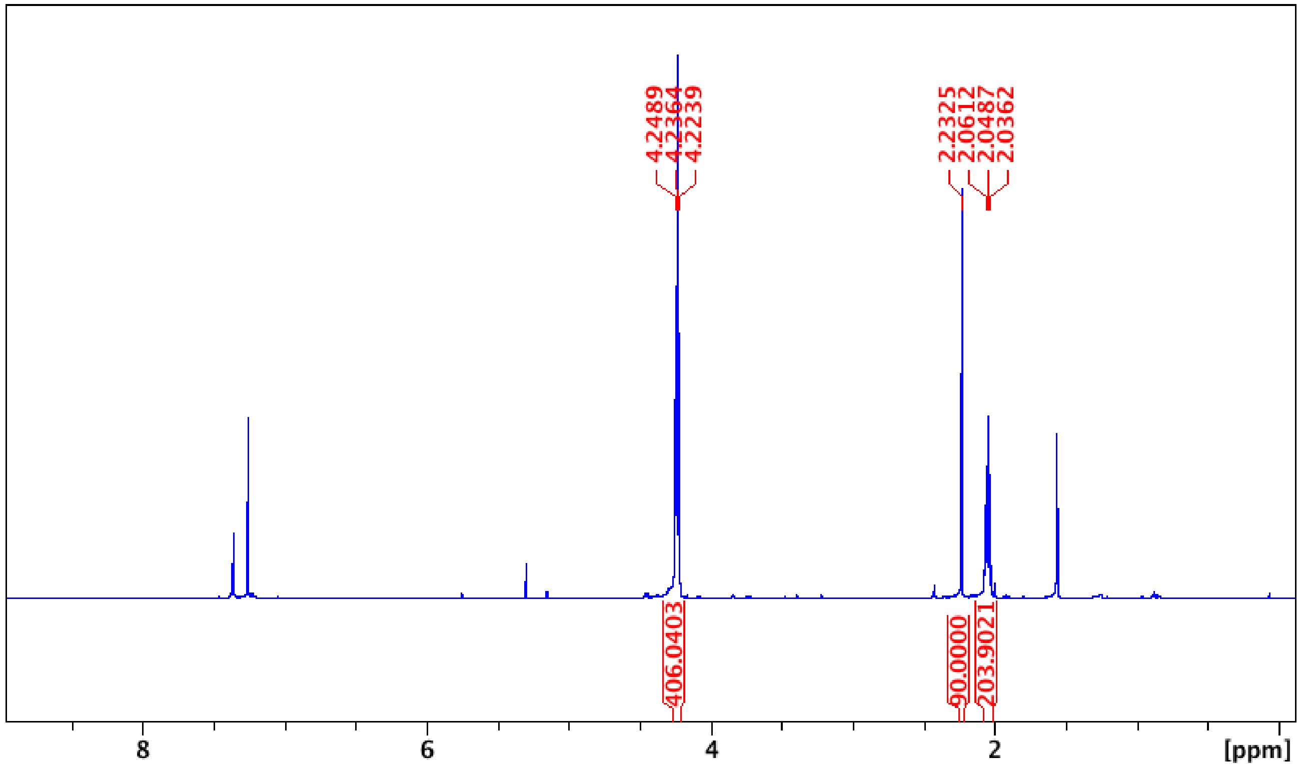

Figure S30. ^{1}H NMR spectrum ($CDCl_3$, 500 MHz, 298 K) of TMC polymerization (Table 1, entry 2). Standard is hexamethylbenzene (HMB). [($fc^{P,B}$)Zn(μ-OCH_2Ph)]$_2$: HMB: TMC ratio is 1:10:202. δ (ppm) 2.05 (t, 2H, C*H*$_2$ PTMC), 2.23 (s, 18H, C*H*$_3$ HMB), 4.23 (t, 4H, C*H*$_2$ PTMC).

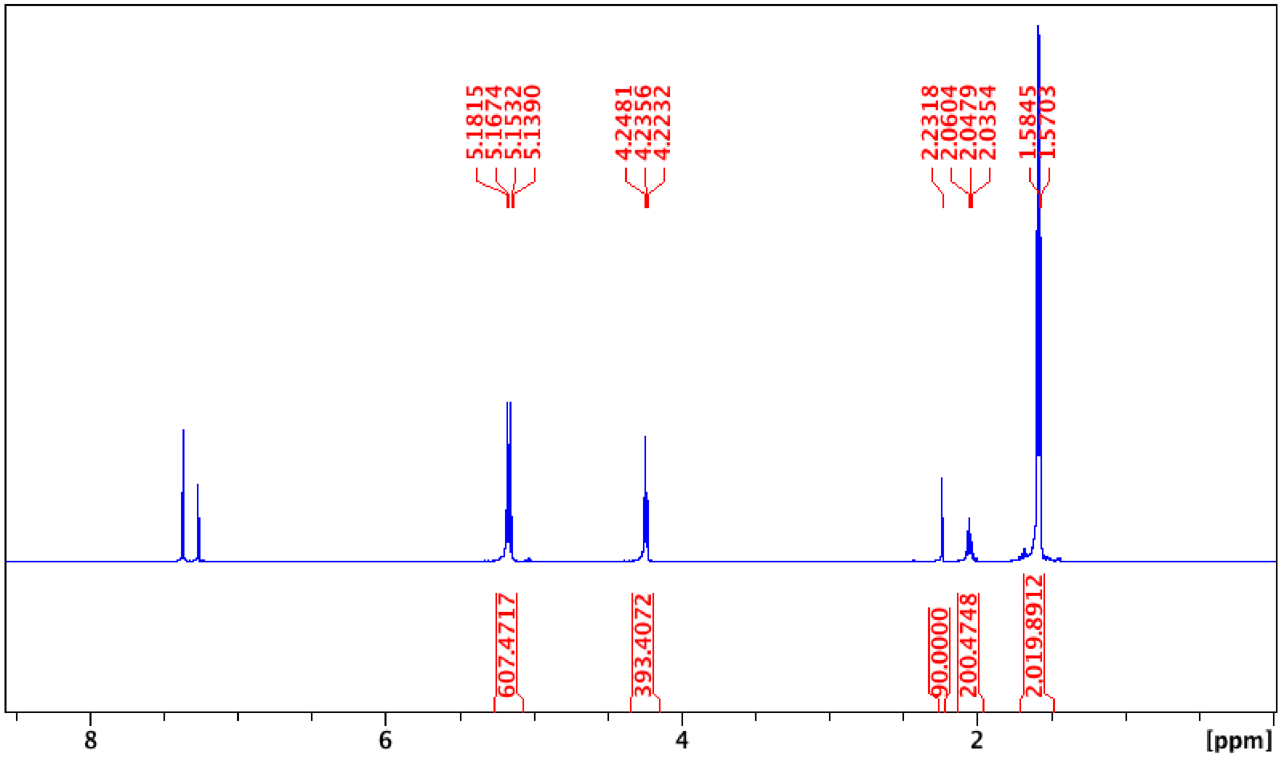

Figure S31. ^{1}H NMR spectrum ($CDCl_3$, 500 MHz, 298 K) of PLA-*b*-PTMC polymerization (Table 1, entry 3). Standard is hexamethylbenzene (HMB). $[(fc^{P,B})Zn(\mu\text{-}OCH_2Ph)]_2$: HMB: TMC: LA ratio is 1:10:196:608. δ (ppm) 1.58 (d, 6H, $CHCH_3$ PLA), 2.05 (t, 2H, CH_2 PTMC), 2.23 (s, 18H, CH_3 HMB), 4.23 (t, 4H, CH_2 PTMC), 5.16 (q, 2H, $CHCH_3$ PLA).

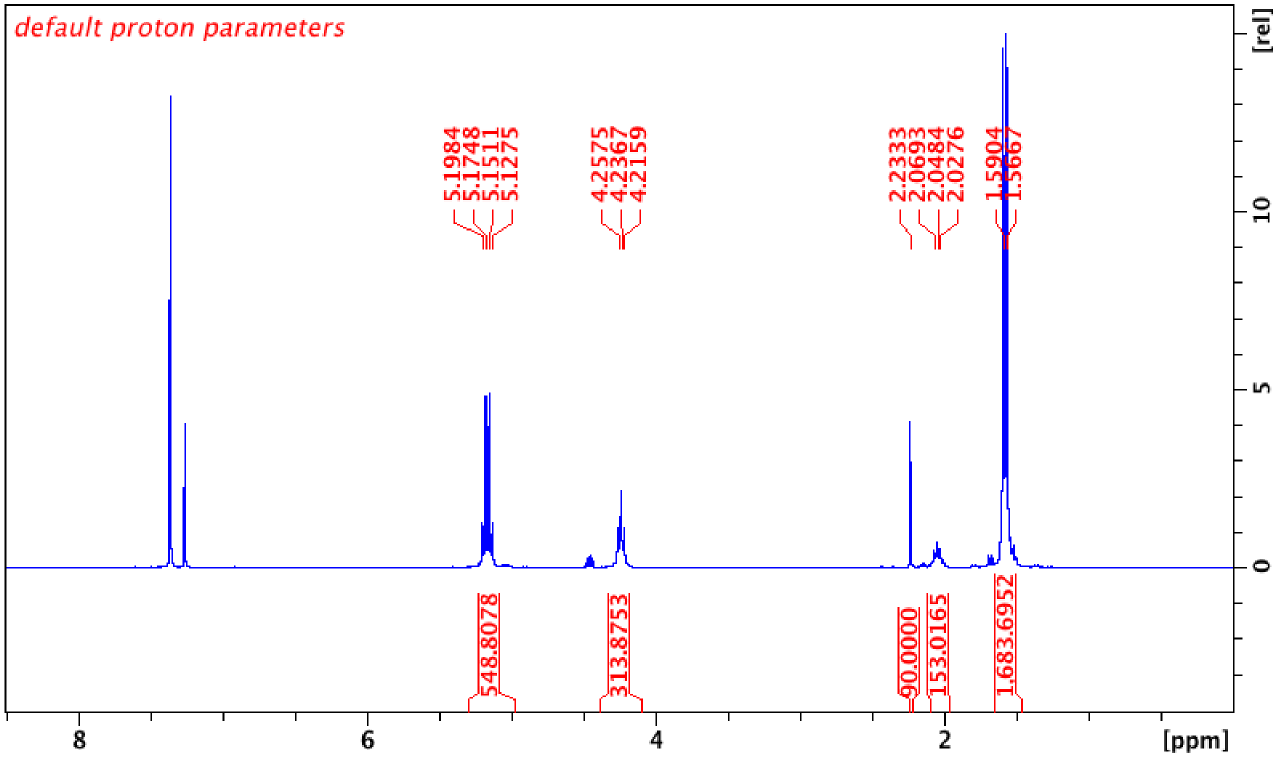

Figure S32. ^{1}H NMR spectrum ($CDCl_3$, 300 MHz, 298 K) of PTMC-*b*-PLA polymerization (Table 1, entry 4). Standard is hexamethylbenzene (HMB). $[(fc^{P,B})Zn(\mu\text{-}OCH_2Ph)]_2$: HMB: TMC: LA ratio is 1:10:158:550. δ (ppm) 1.58 (d, 6H, $CHCH_3$ PLA), 2.05 (t, 2H, CH_2 PTMC), 2.23 (s, 18H, CH_3 HMB), 4.23 (t, 4H, CH_2 PTMC), 5.16 (q, 2H, $CHCH_3$ PLA).

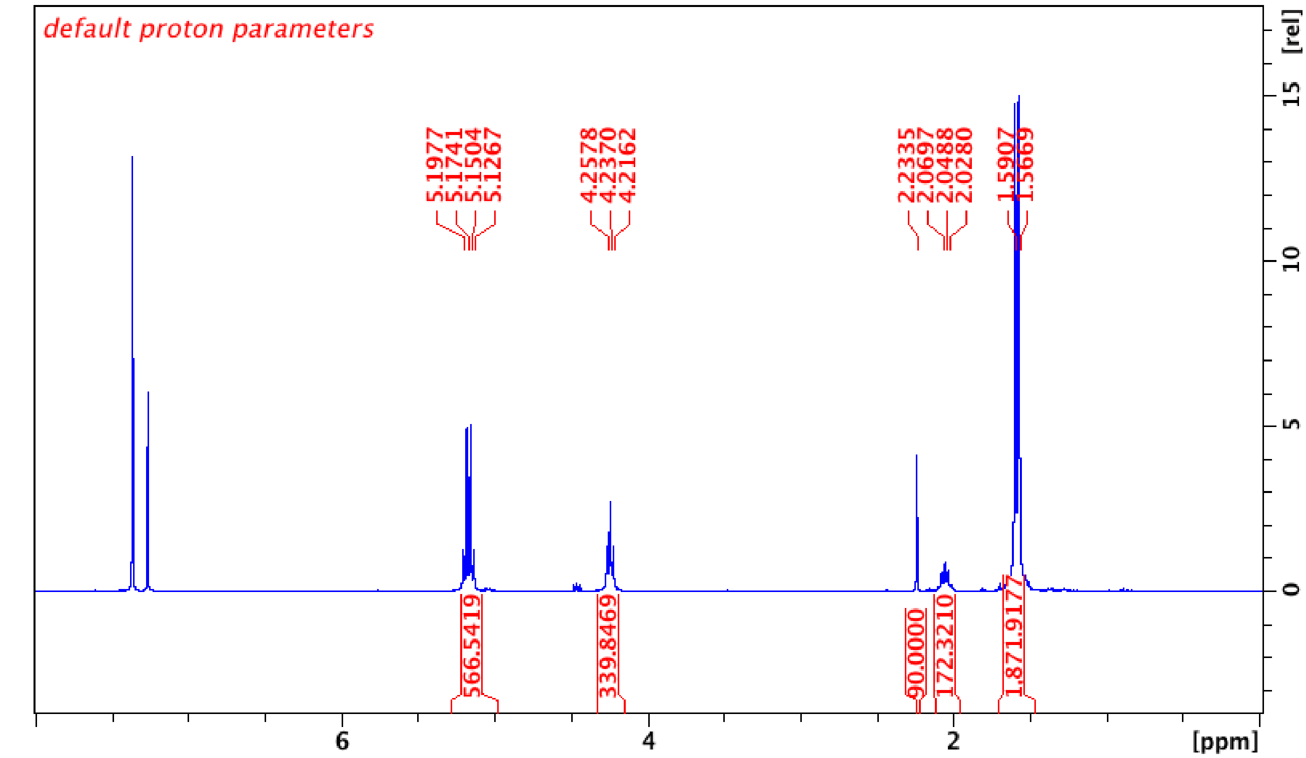

Figure S33. ^{1}H NMR spectrum ($CDCl_3$, 300 MHz, 298 K) of PTMC-*b*-PLA-*b*-PTMC polymerization (Table 1, entry 5). Standard is hexamethylbenzene (HMB). [(fc^{P,B})Zn(μ-OCH_2Ph)]$_2$: HMB: TMC: LA ratio is 1:10:170:566. δ (ppm) 1.58 (d, 6H, CHC*H*$_3$ PLA), 2.05 (t, 2H, C*H*$_2$ PTMC), 2.23 (s, 18H, C*H*$_3$ HMB), 4.23 (t, 4H, C*H*$_2$ PTMC), 5.16 (q, 2H, C*H*CH_3 PLA).

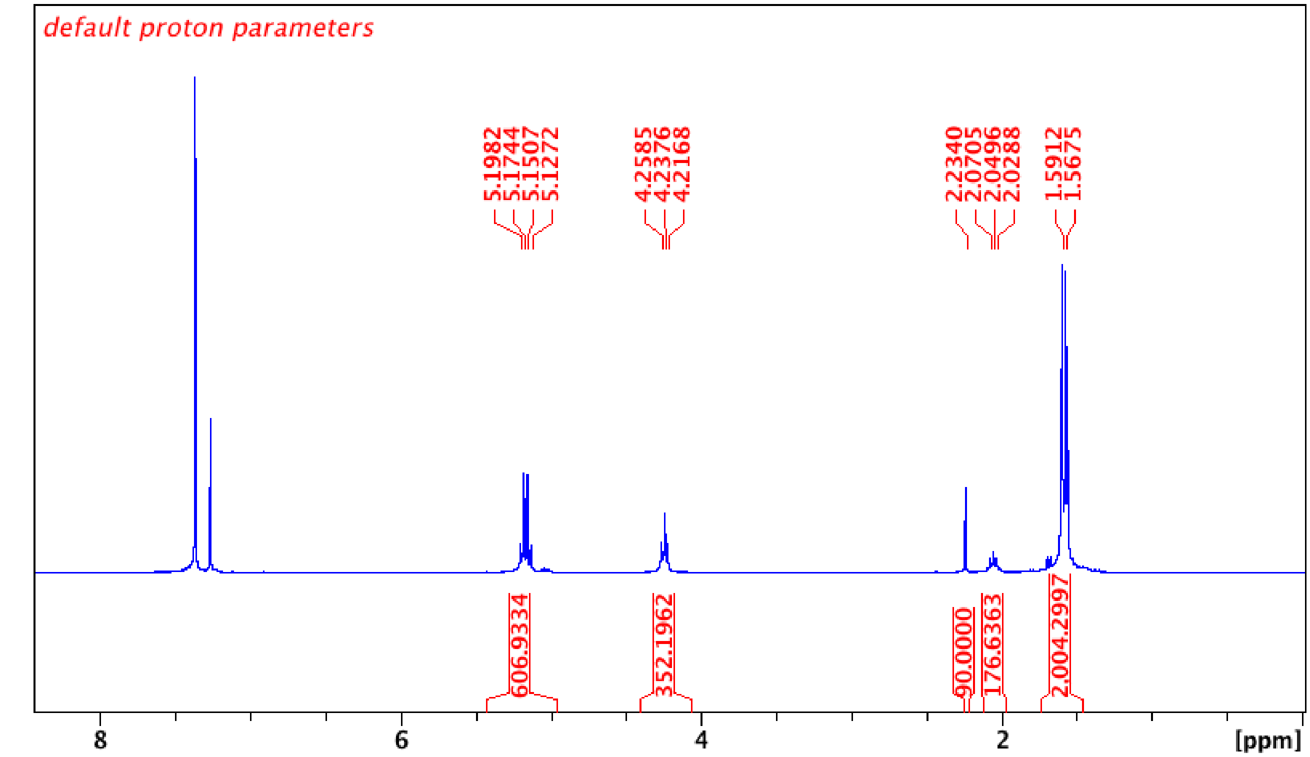

Figure S34. ^{1}H NMR spectrum ($CDCl_3$, 300 MHz, 298 K) of PLA-*b*-PTMC-*b*-PLA polymerization (Table 1, entry 6). Standard is hexamethylbenzene (HMB). [(fc^{P,B})Zn(μ-OCH_2Ph)]$_2$: HMB: TMC: LA ratio is 1:10:176:606. δ (ppm) 1.58 (d, 6H, CHC*H*$_3$ PLA), 2.05 (t, 2H, C*H*$_2$ PTMC), 2.23 (s, 18H, C*H*$_3$ HMB), 4.23 (t, 4H, C*H*$_2$ PTMC), 5.16 (q, 2H, C*H*CH_3 PLA).

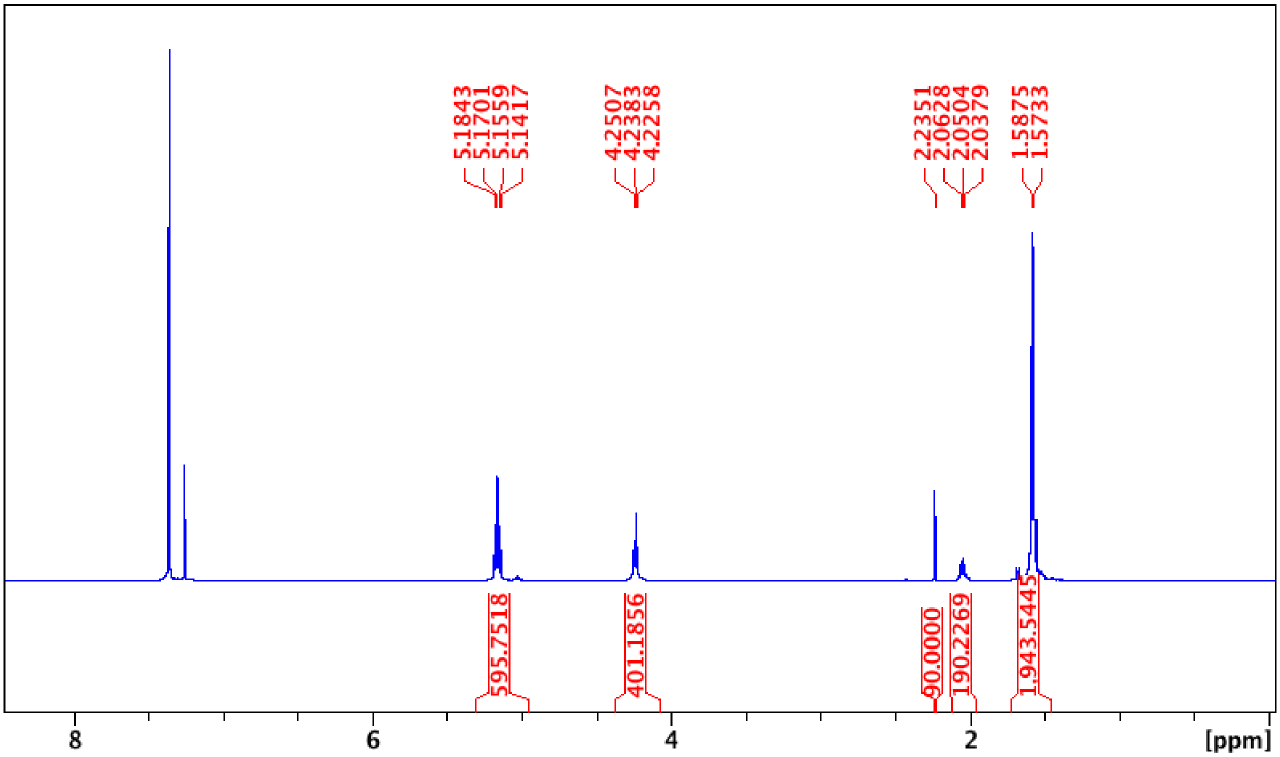

Figure S35. ^{1}H NMR spectrum ($CDCl_3$, 500 MHz, 298 K) of PLA-*b*-PTMC-*b*-PLA-*b*-PTMC polymerization (Table 1, entry 7). Standard is hexamethylbenzene (HMB). $[(fc^{P,B})Zn(\mu\text{-}OCH_2Ph)]_2$: HMB: TMC: LA ratio is 1:10:200:596. δ (ppm) 1.58 (d, 6H, CHCH_3 PLA), 2.05 (t, 2H, CH_2 PTMC), 2.23 (s, 18H, CH_3 HMB), 4.23 (t, 4H, CH_2 PTMC), 5.16 (q, 2H, C*H*CH_3 PLA).

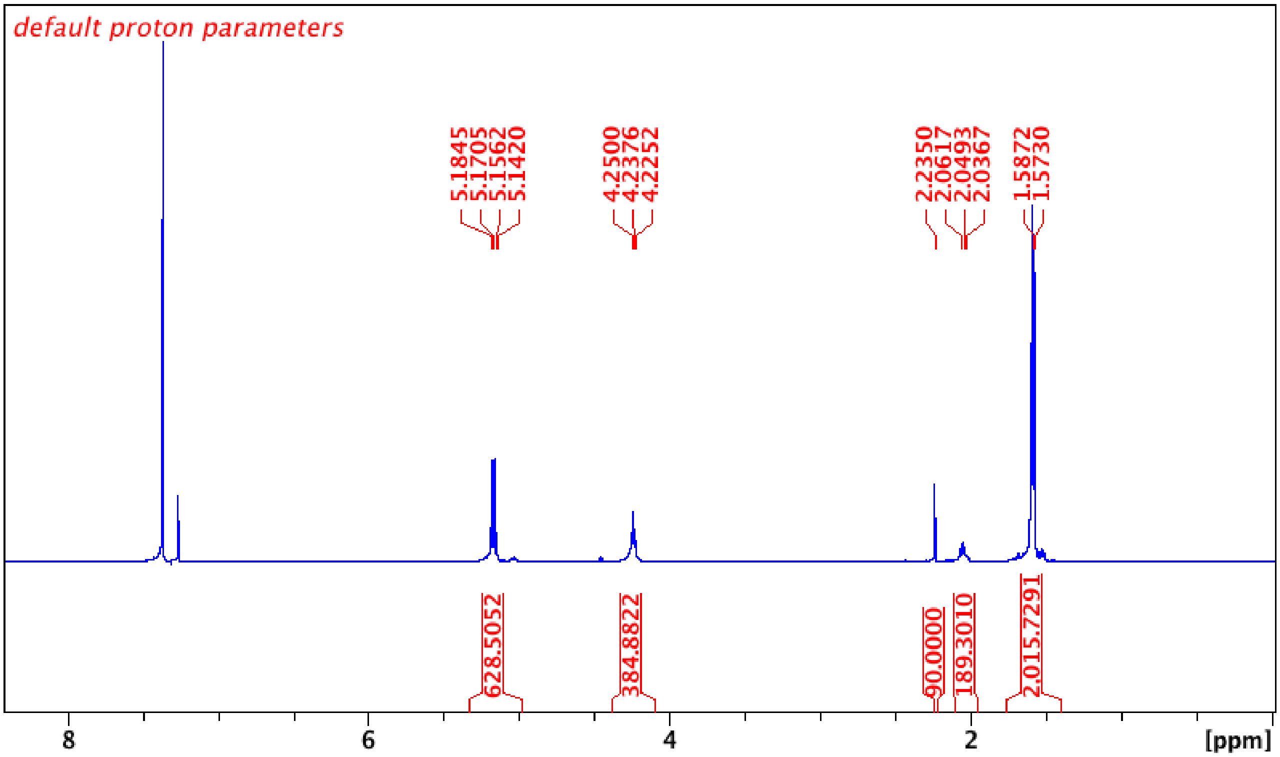

Figure S36. ^{1}H NMR spectrum ($CDCl_3$, 500 MHz, 298 K) of PTMC-*b*-PLA-*b*-PTMC-*b*-PLA-*b*-PTMC polymerization (Table 1, entry 8). Standard is hexamethylbenzene (HMB). $[(fc^{P,B})Zn(\mu\text{-}OCH_2Ph)]_2$: HMB: TMC: LA ratio is 1:10:192:628. δ (ppm) 1.58 (d, 6H, CHCH_3 PLA), 2.05 (t, 2H, CH_2 PTMC), 2.23 (s, 18H, CH_3 HMB), 4.23 (t, 4H, CH_2 PTMC), 5.16 (q, 2H, C*H*CH_3 PLA).

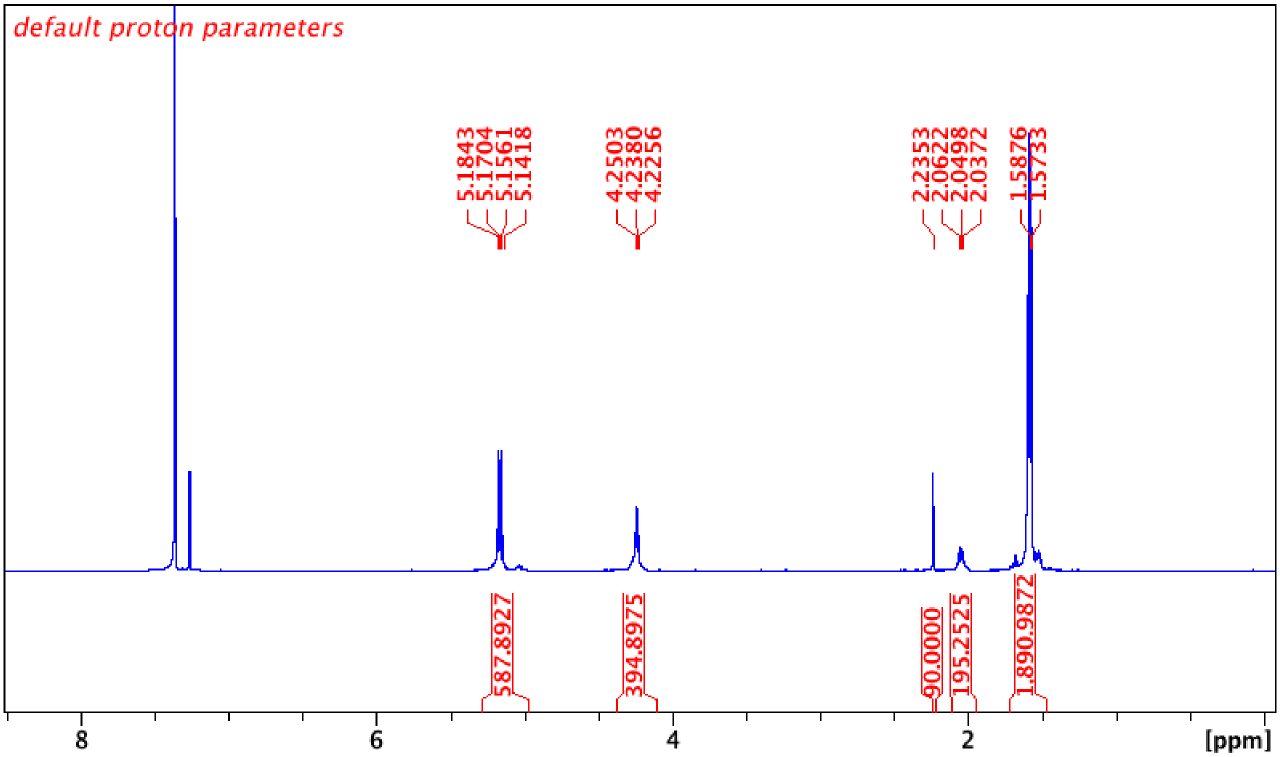

Figure S37. ^{1}H NMR spectrum ($CDCl_3$, 500 MHz, 298 K) of PLA-*b*-PTMC-*b*-PLA-*b*-PTMC-*b*-PLA polymerization (Table 1, entry 9). Standard is hexamethylbenzene (HMB). $[(fc^{P,B})Zn(\mu\text{-}OCH_2Ph)]_2$: HMB: TMC: LA ratio is 1:10:198:588. δ (ppm) 1.58 (d, 6H, CHCH_3 PLA), 2.05 (t, 2H, CH_2 PTMC), 2.23 (s, 18H, CH_3 HMB), 4.23 (t, 4H, CH_2 PTMC), 5.16 (q, 2H, C*H*CH_3 PLA).

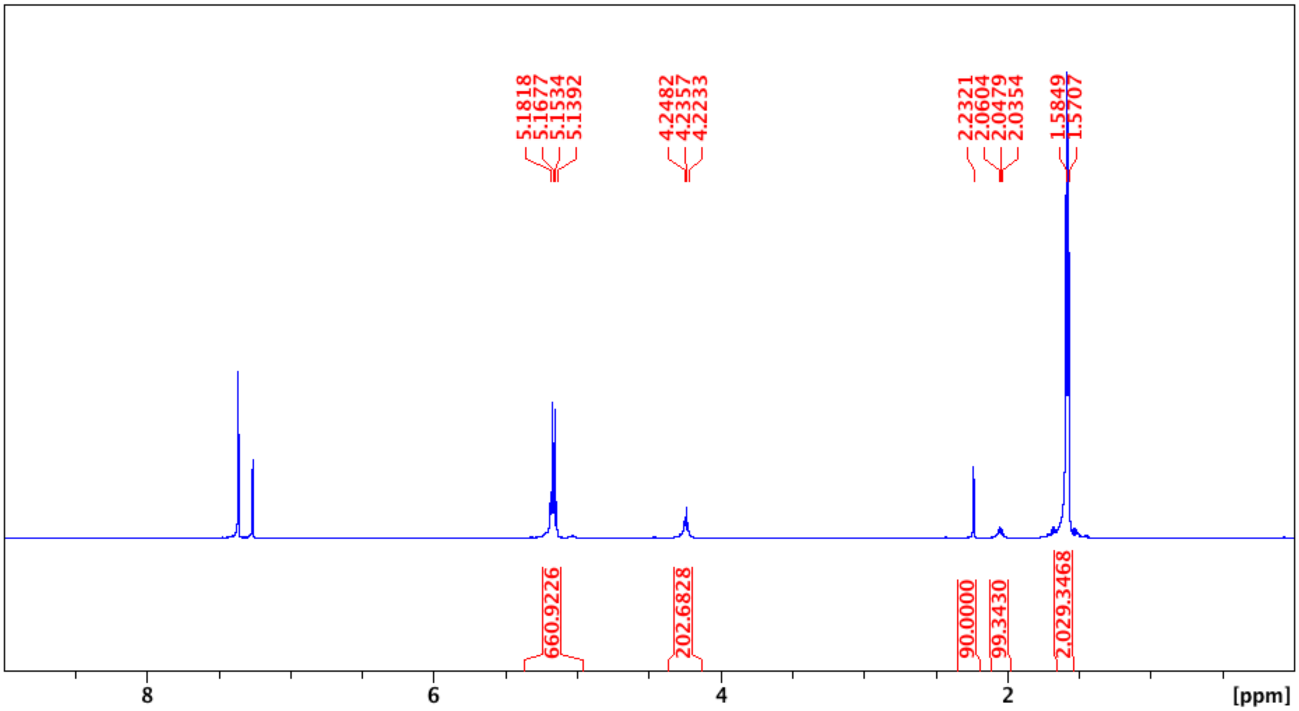

Figure S38. ^{1}H NMR spectrum ($CDCl_3$, 300 MHz, 298 K) of PLA-*b*-PTMC-*b*-PLA polymerization (Table 1, entry 10). Standard is hexamethylbenzene (HMB). $[(fc^{P,B})Zn(\mu\text{-}OCH_2Ph)]_2$: HMB: TMC: LA ratio is 1:10:102:660. δ (ppm) 1.58 (d, 6H, CHCH_3 PLA), 2.05 (t, 2H, CH_2 PTMC), 2.23 (s, 18H, CH_3 HMB), 4.23 (t, 4H, CH_2 PTMC), 5.16 (q, 2H, C*H*CH_3 PLA).

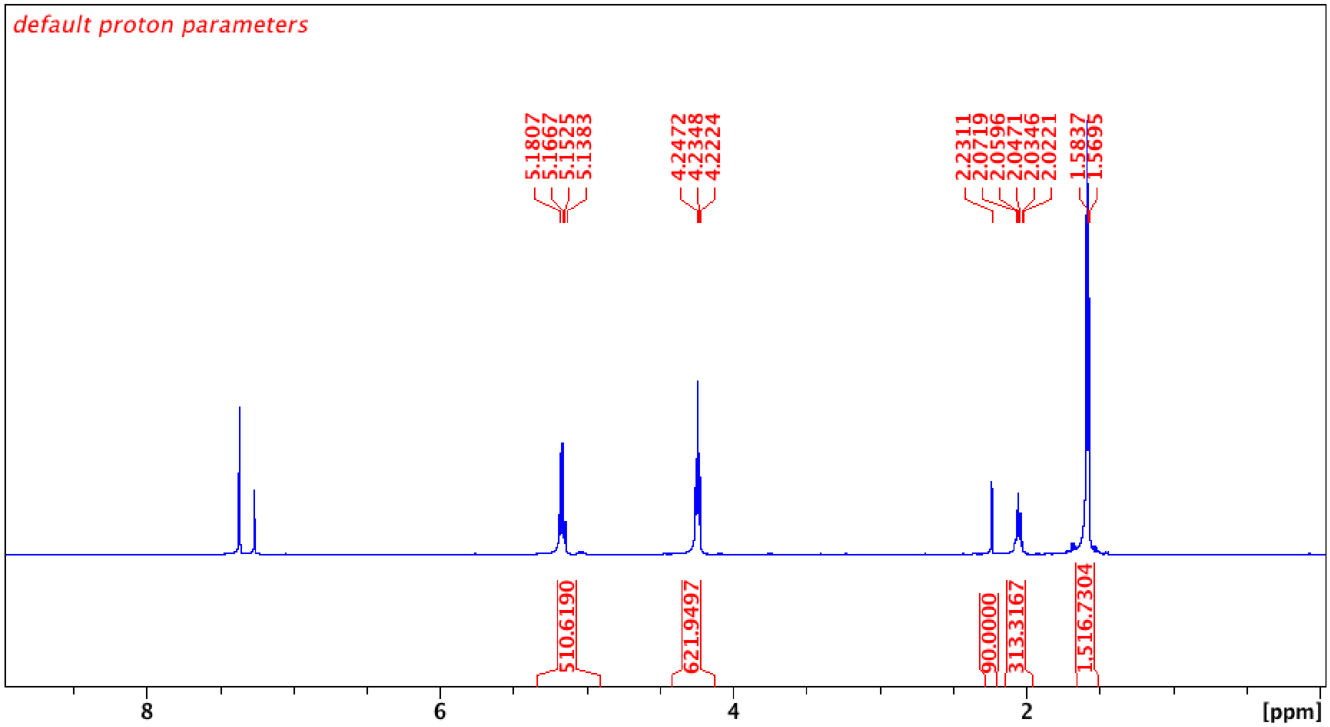

Figure S39. ^{1}H NMR spectrum ($CDCl_3$, 300 MHz, 298 K) of PLA-*b*-PTMC-*b*-PLA polymerization (Table 1, entry 11). Standard is hexamethylbenzene (HMB). $[(fc^{P,B})Zn(\mu\text{-}OCH_2Ph)]_2$: HMB: TMC: LA ratio is 1:10:312:510. δ (ppm) 1.58 (d, 6H, CHCH_3 PLA), 2.05 (t, 2H, CH_2 PTMC), 2.23 (s, 18H, CH_3 HMB), 4.23 (t, 4H, CH_2 PTMC), 5.16 (q, 2H, C*H*CH_3 PLA).

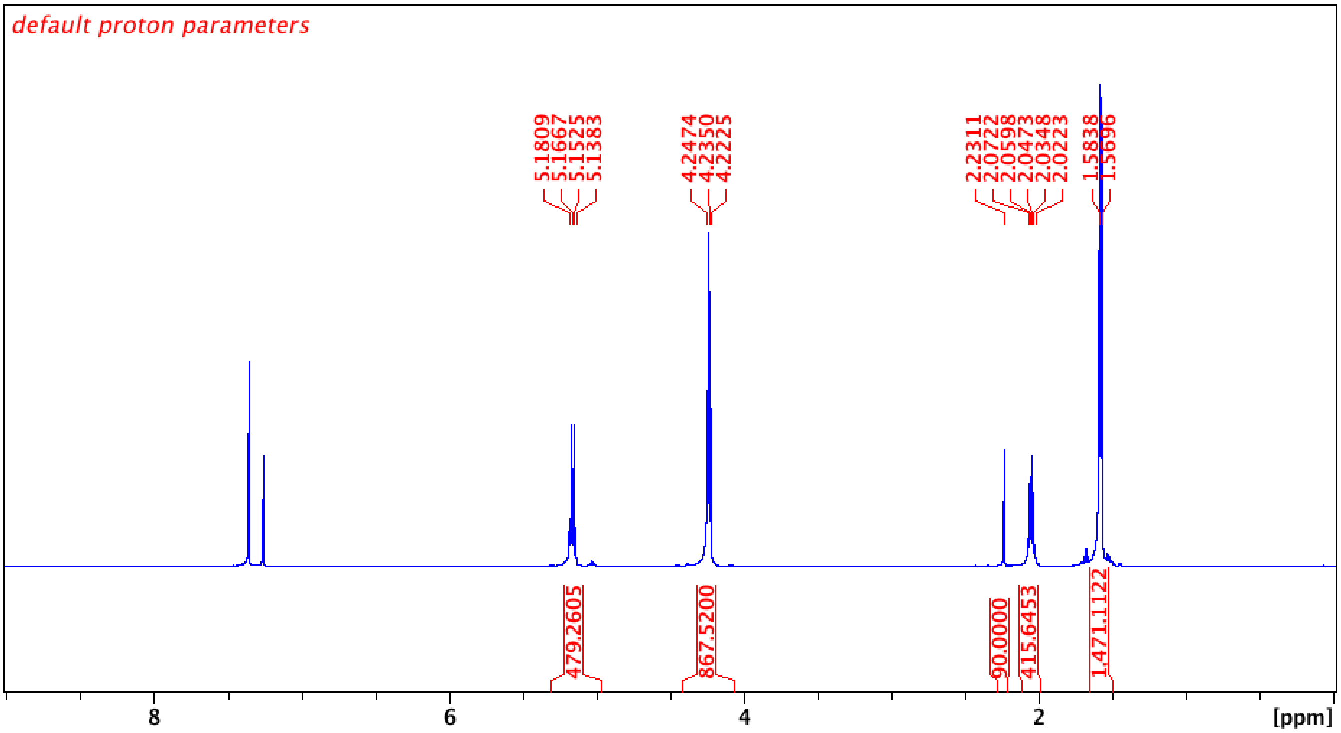

Figure S40. ^{1}H NMR spectrum ($CDCl_3$, 300 MHz, 298 K) of PLA-*b*-PTMC-*b*-PLA polymerization (Table 1, entry 12). Standard is hexamethylbenzene (HMB). $[(fc^{P,B})Zn(\mu\text{-}OCH_2Ph)]_2$: HMB: TMC: LA ratio is 1:10:434:480. δ (ppm) 1.58 (d, 6H, CHCH_3 PLA), 2.05 (t, 2H, CH_2 PTMC), 2.23 (s, 18H, CH_3 HMB), 4.23 (t, 4H, CH_2 PTMC), 5.16 (q, 2H, C*H*CH_3 PLA).

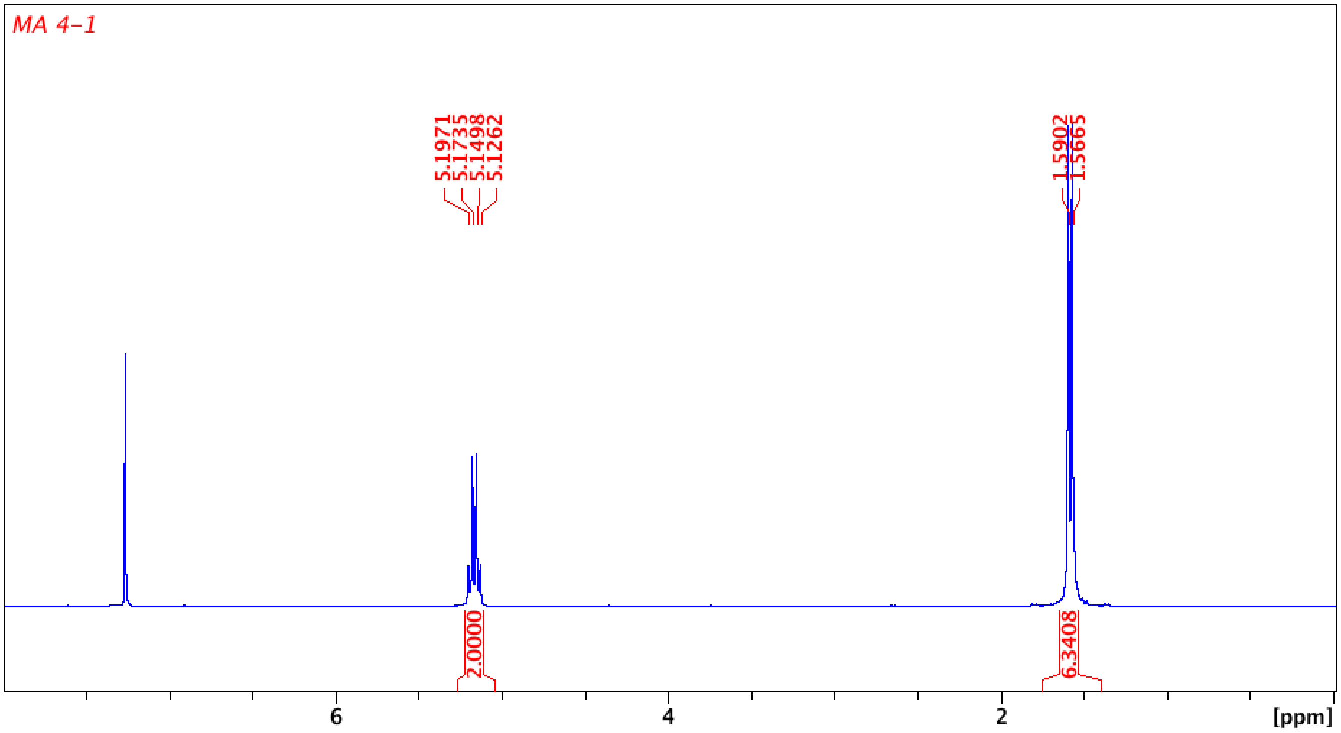

Figure S41. ^{1}H NMR spectrum ($CDCl_3$, 300 MHz, 298 K) of L-LA melt polymerization. [(fc^{P,B})Zn(μ-OCH_2Ph)]$_2$: LA loading ratio is 1:600. δ (ppm) 1.58 (d, 6H, CHCH_3 PLA), 5.16 (q, 2H, CHCH$_3$ PLA).

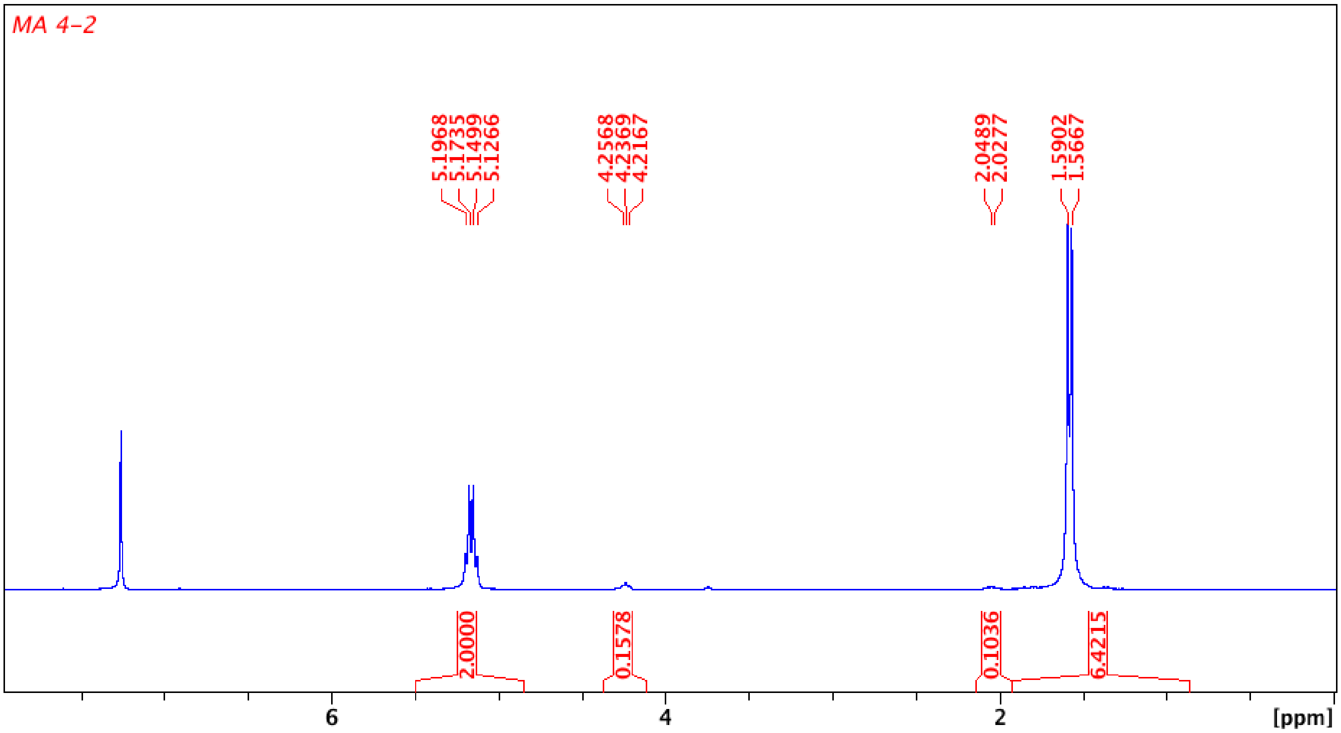

Figure S42. ^{1}H NMR spectrum ($CDCl_3$, 300 MHz, 298 K) of PTMC-*b*-PLA melt polymerization. [(fc^{P,B})Zn(μ-OCH_2Ph)]$_2$:TMC:LA loading ratio is 1:600:600. δ (ppm) 1.58 (d, 6H, CHCH_3 PLA), 2.05 (t, 2H, CH_2 PTMC), 4.23 (t, 4H, CH_2 PTMC), 5.16 (q, 2H, CHCH$_3$ PLA).

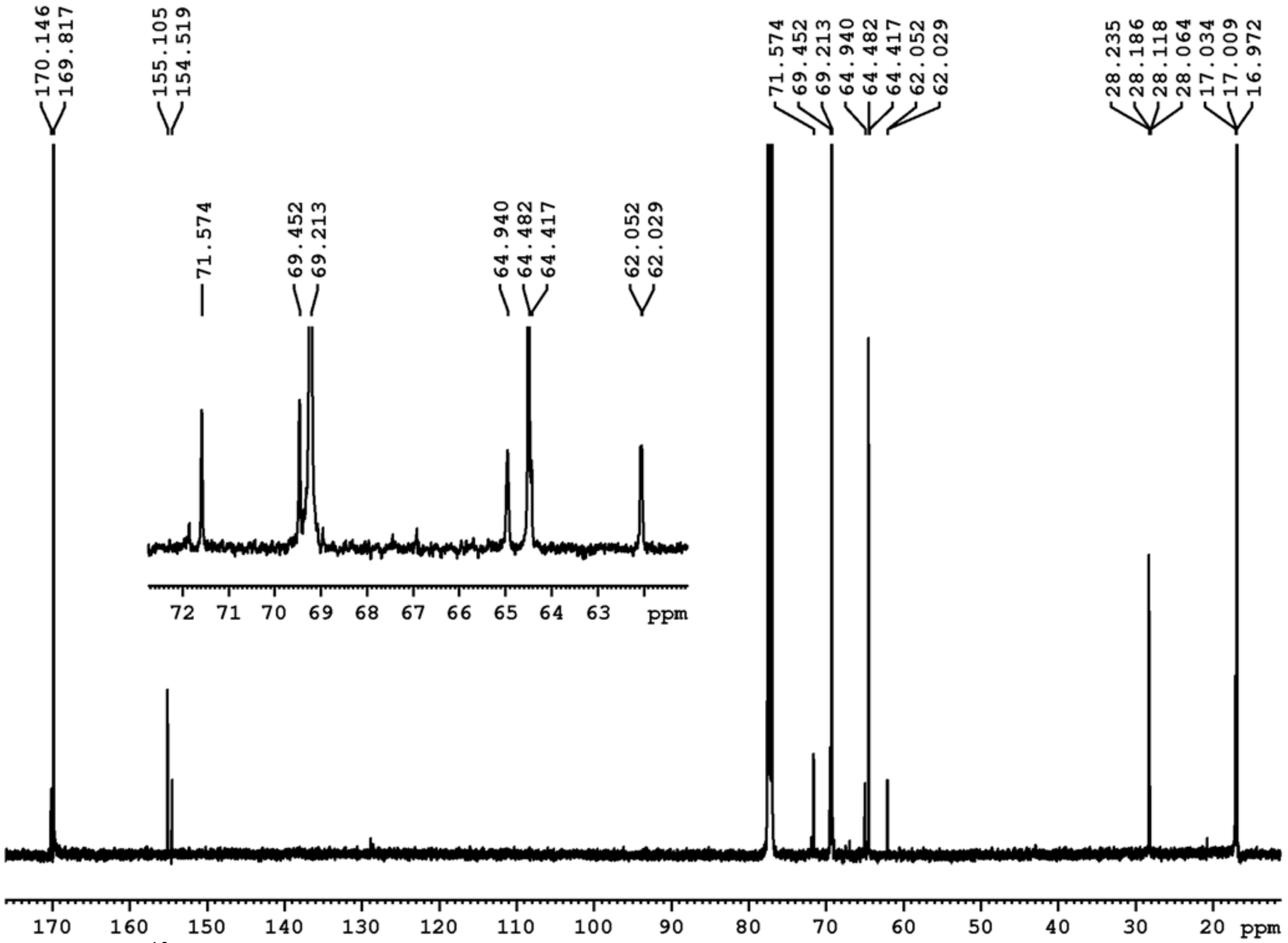

Figure S43. ^{13}C NMR spectrum ($CDCl_3$, 126 MHz, 298 K) of the PLA-*b*-PTMC-*b*-PLA-*b*-PTMC-*b*-PLA copolymer (Table 1, entry 9).

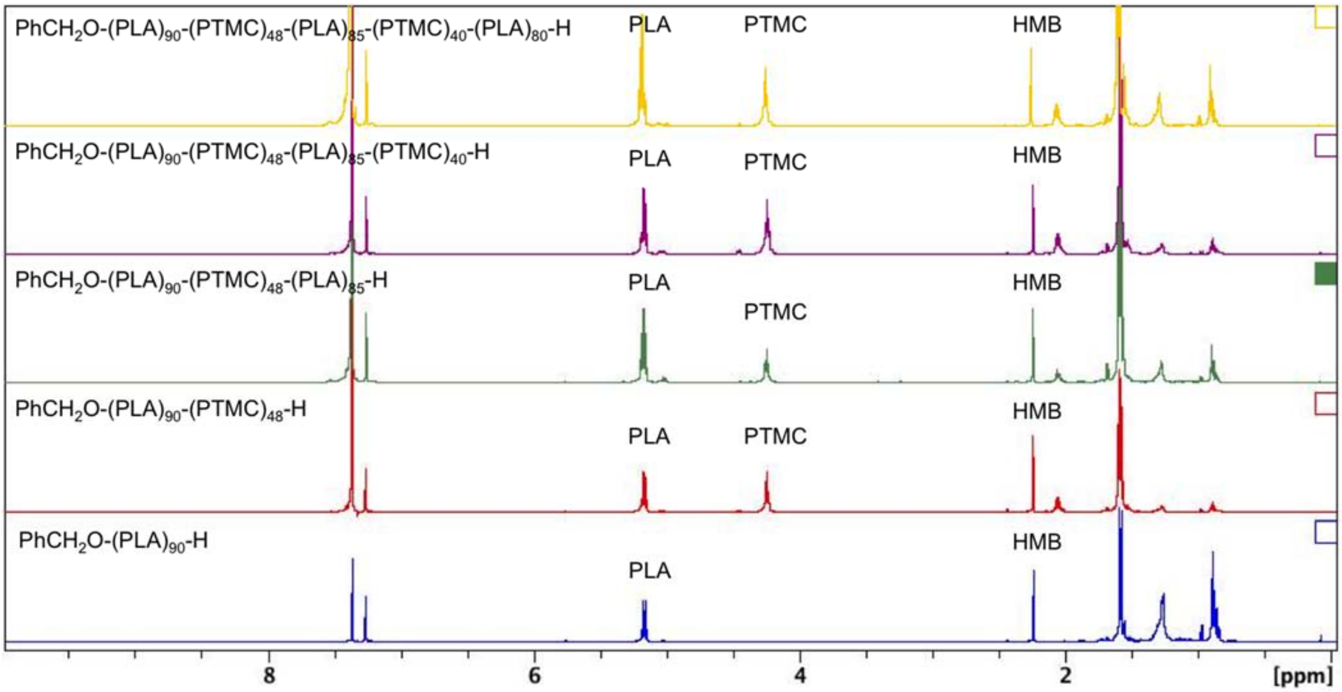

Figure S44. ^{1}H NMR spectra ($CDCl_3$, 500 MHz, 298 K) corresponding to the stepwise preparation of PLA-*b*-PTMC-*b*-PLA-*b*-PTMC-*b*-PLA in the presence of HMB as an internal standard.

Conversion Studies

Table S1. Molar mass versus conversion study of 1,3-trimethylene carbonate.

Time (min)	Conversion (%)	M_n (NMR)	M_n (SEC)	*Đ*
**30**	37	9,400	10,300	1.04
**50**	44	11,200	12,500	1.09
**70**	58	14,800	14,500	1.14
**90**	72	18,400	17,000	1.17

Conditions: benzene as a solvent (1.5 mL) and hexamethylbenzene as an internal standard. The experiment was performed at ambient temperature.

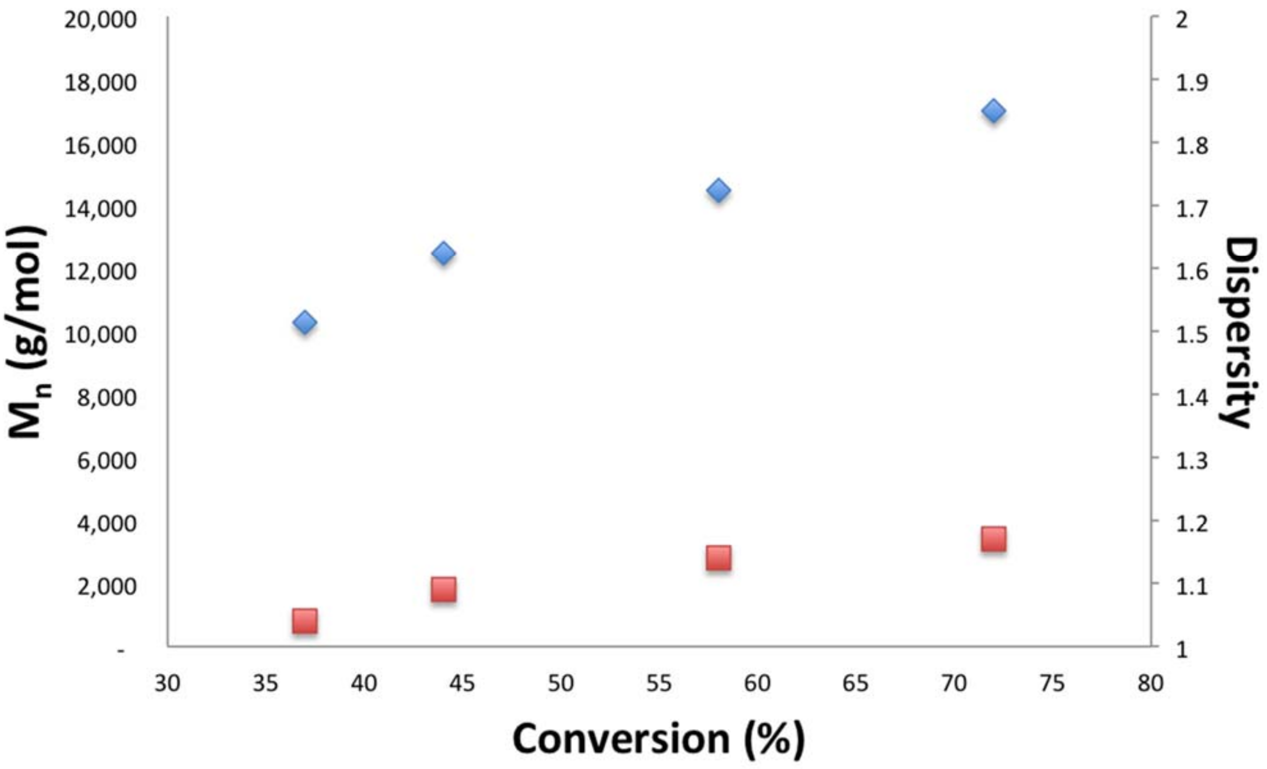

Figure S45. Conversion of 1,3-trimethylene carbonate versus M_n.

Table S2. Molar mass versus conversion study of L-lactide.

Time (min)	Conversion (%)	M_n (NMR)	M_n (SEC)	*Đ*
**10**	18	8,100	8,800	1.06
**20**	31	13,500	13,500	1.09
**30**	42	19,700	20,400	1.08
**40**	52	24,100	25,400	1.01
**50**	60	27,700	28,700	1.03
**60**	69	31,200	31,700	1.02

Conditions: benzene as a solvent (1.5 mL) and hexamethylbenzene as an internal standard. The experiment was performed at 70 °C.

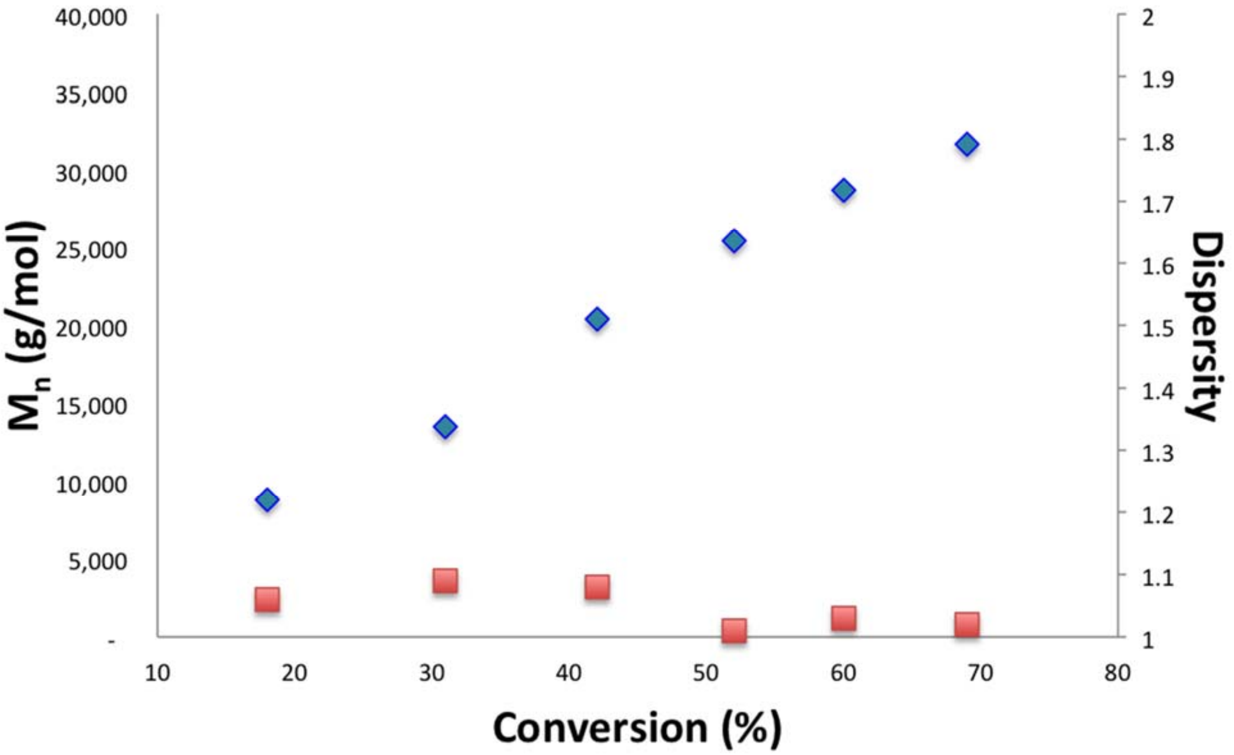

Figure S46. Conversion of L-lactide versus M_n.

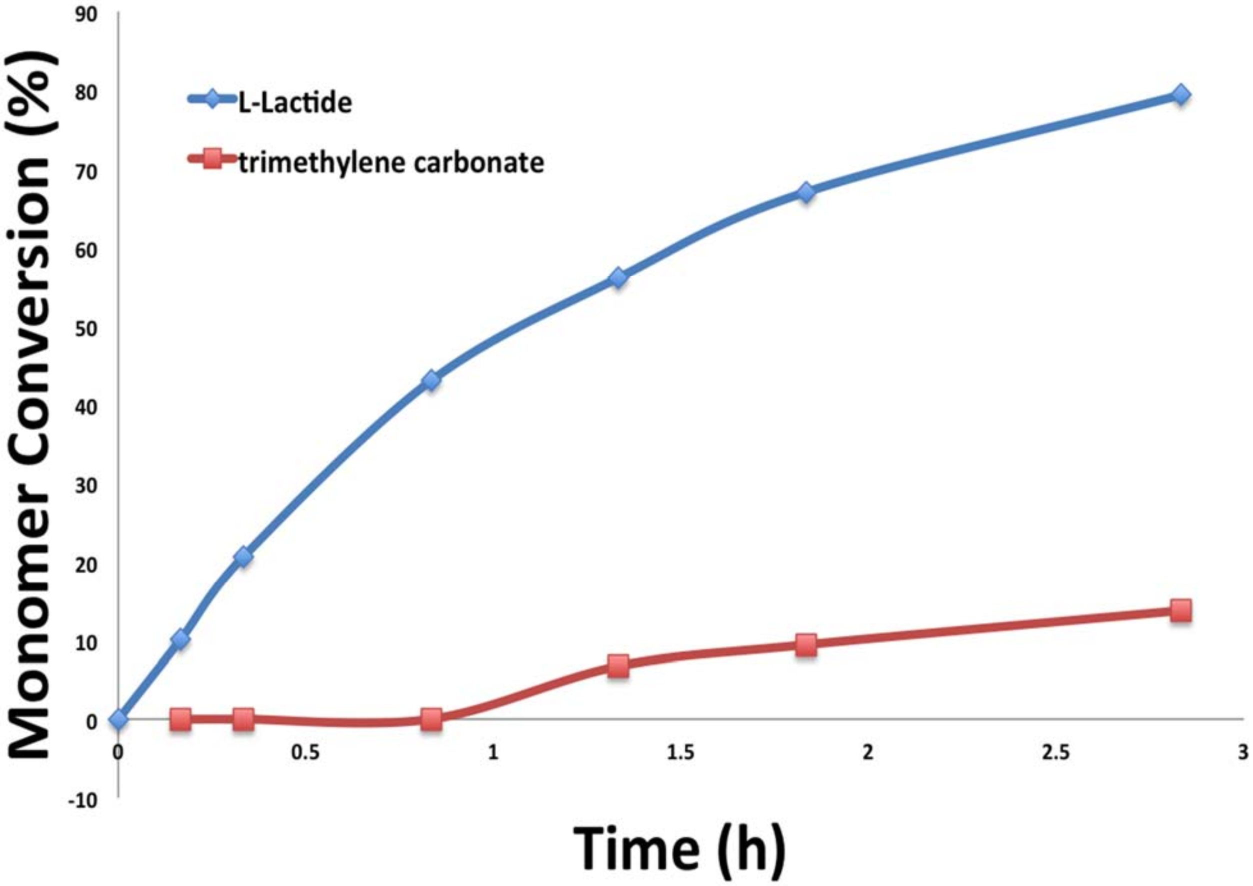

Figure S47. One pot polymerization of L-lactide (100 equivalents) and trimethylene carbonate (50 equivalents) in 0.5 mL of C_6D_6 at 50 °C.

Size Exclusion Chromatography

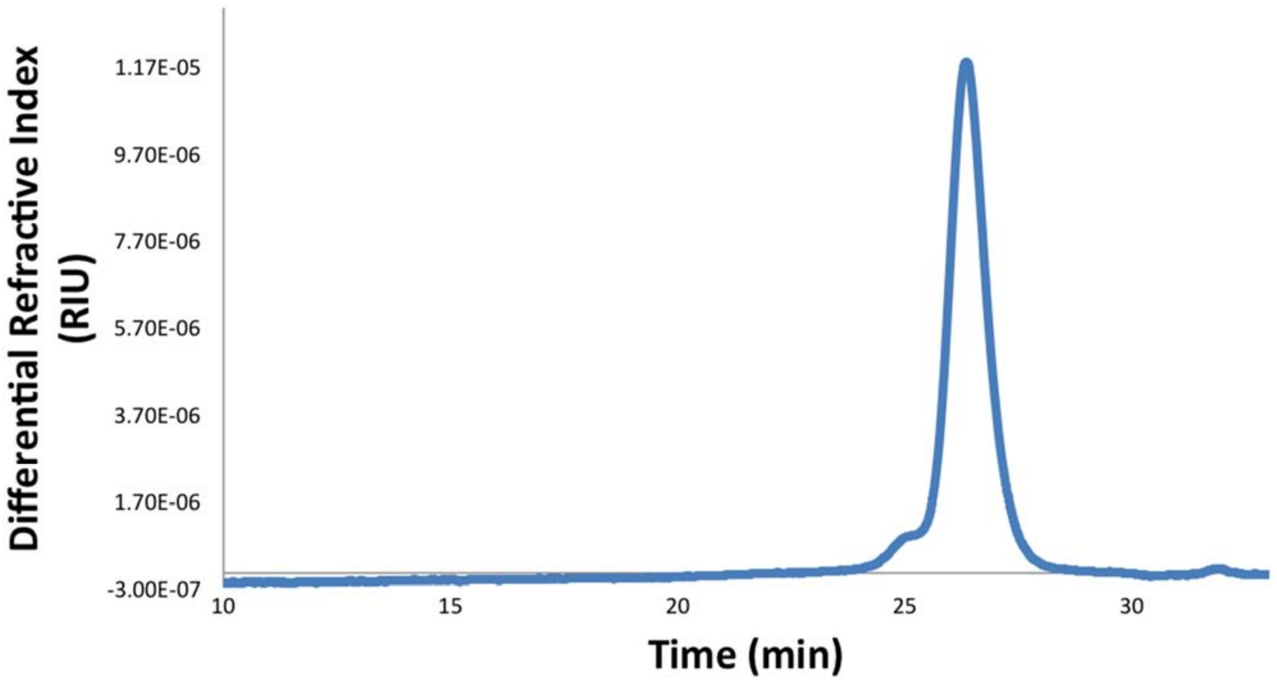

Figure S48. Polymerization of 101 equivalents of 1,3-trimethylene carbonate (Table 1, entry 2); M_n = 9,000; M_w =9,100; $Đ$ = 1.01.

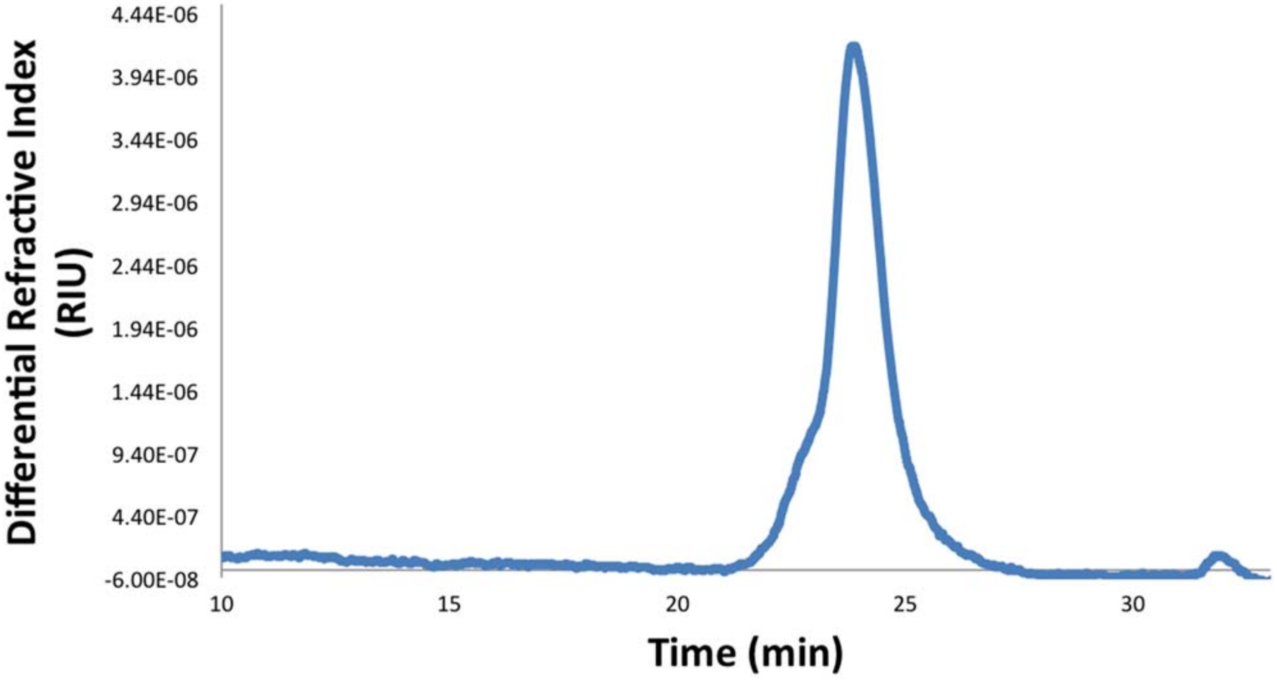

Figure S49. Polymerization of 283 equivalents of L-lactide (Table 1, entry 1); M_n = 39,800; M_w = 45,200; $Đ$ = 1.14.

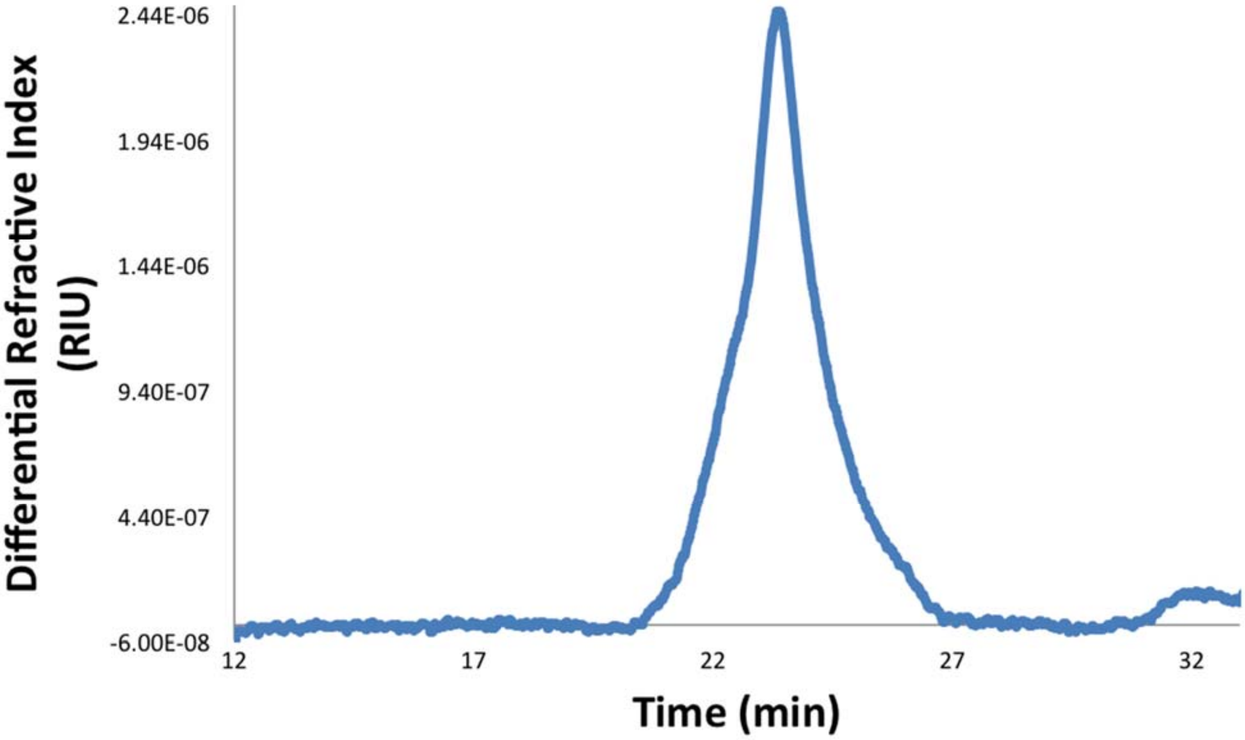

Figure S50. SEC trace of PLA-*b*-PTMC copolymer (Table 1, entry 3); M_n = 55,500; M_w = 61,800; *Đ* = 1.12.

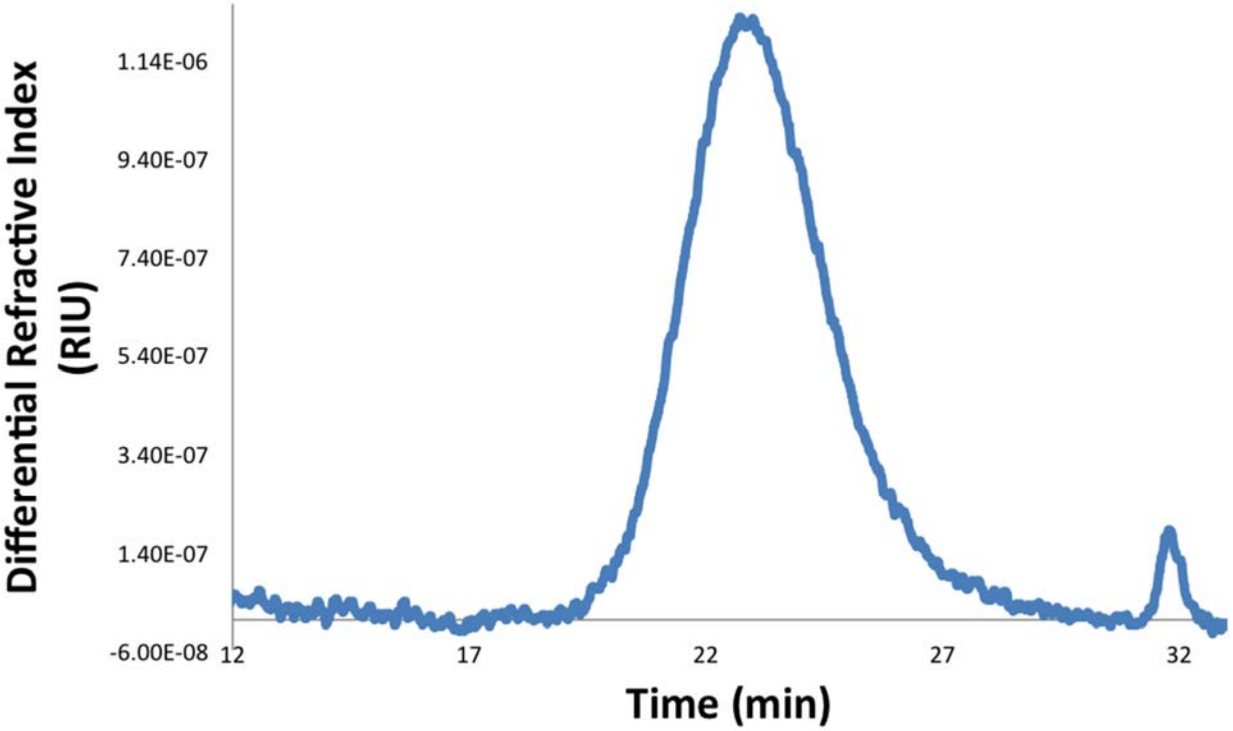

Figure S51. SEC trace of PTMC-*b*-PLA copolymer (Table 1, entry 4); M_n = 47,000; M_w = 75,000; *Đ* = 1.60.

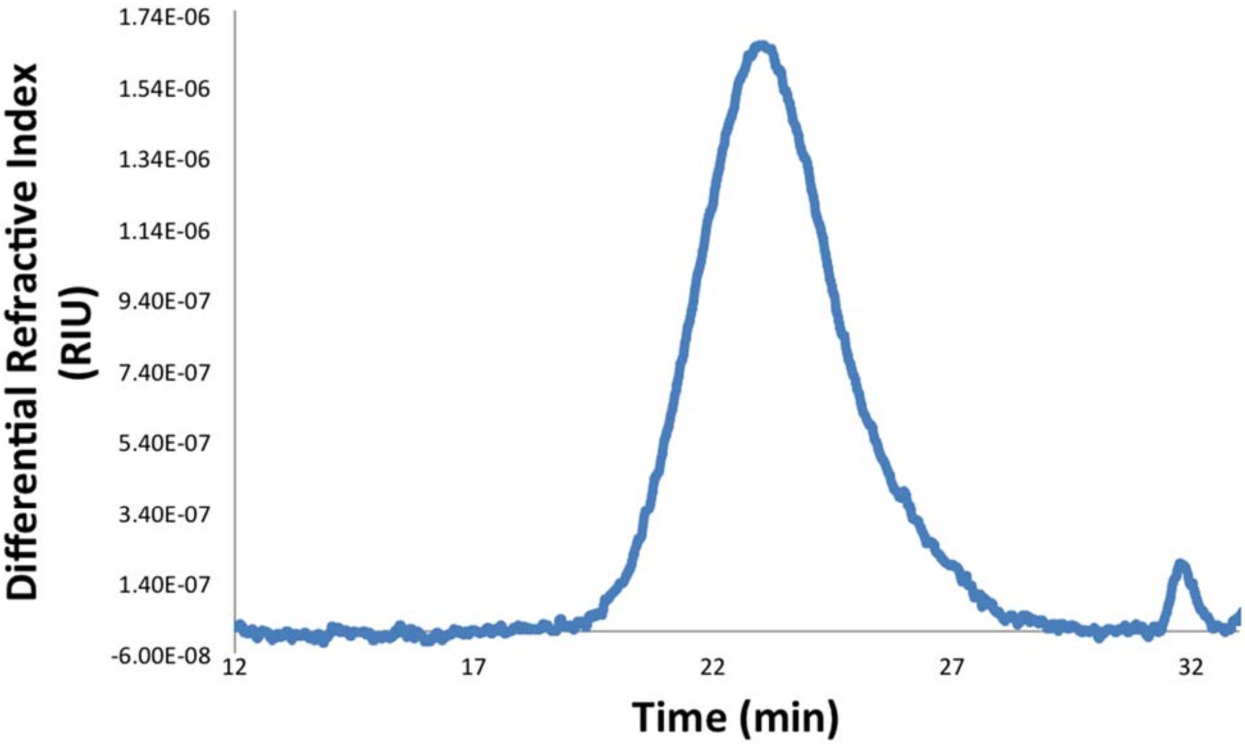

Figure S52. SEC trace of PTMC-*b*-PLA-*b*-PTMC copolymer (Table 1, entry 5); M_n = 43,200; M_w = 72,200; *Đ* = 1.67.

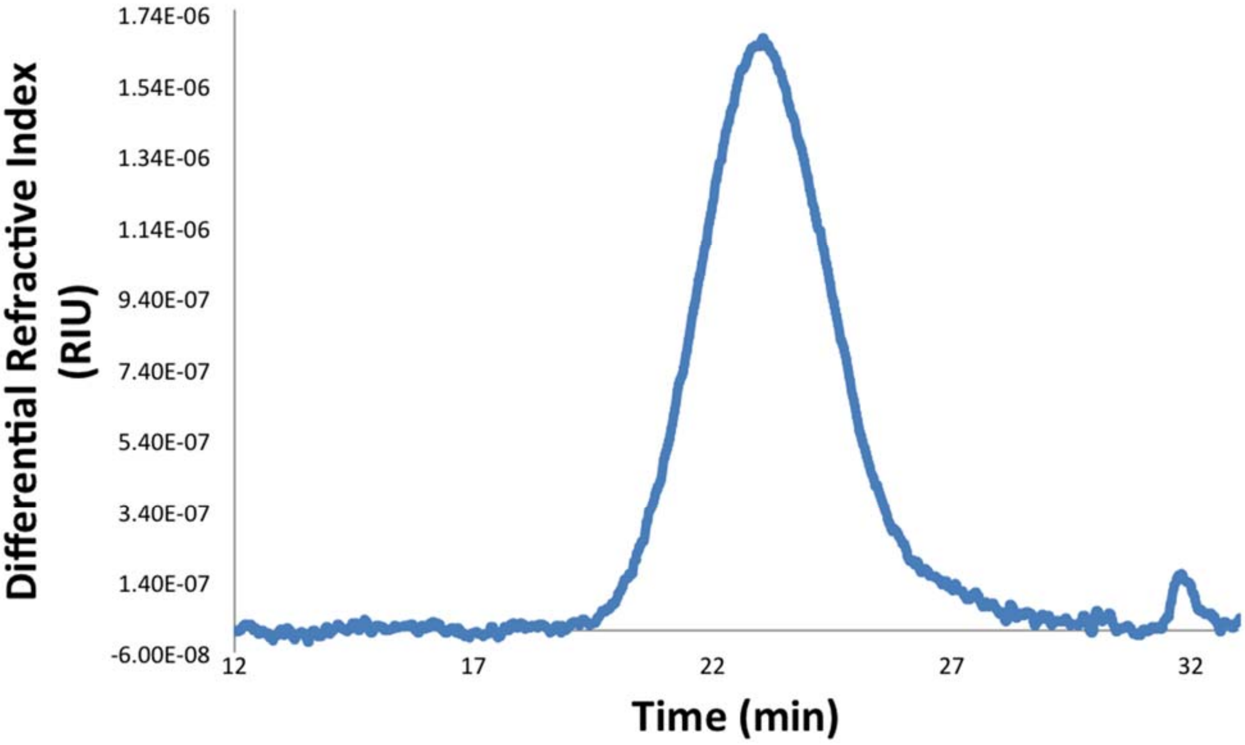

Figure S53. SEC trace of PLA-*b*-PTMC-*b*-PLA copolymer (Table 1, entry 6); M_n = 55,600; M_w = 81,400; *Đ* = 1.46.

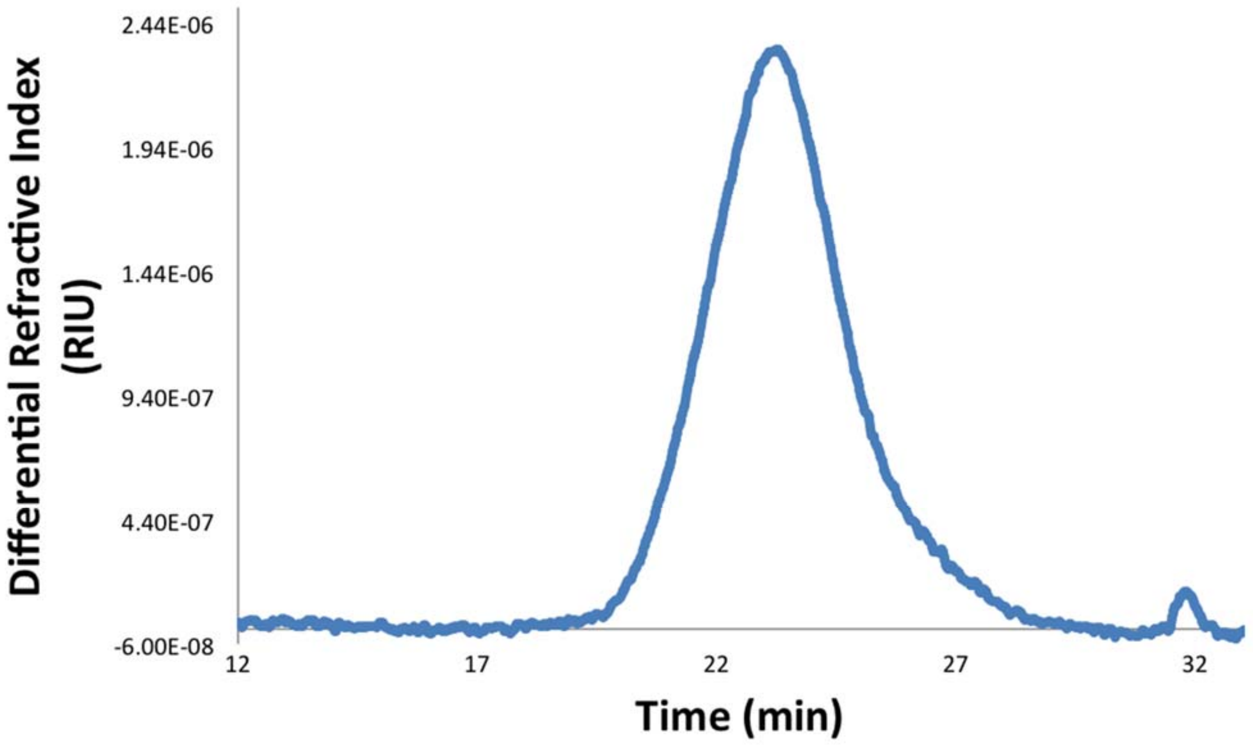

Figure S54. SEC trace of PLA-*b*-PTMC-*b*-PLA-*b*-PTMC copolymer (Table 1, entry 7); M_n = 48,200; M_w = 71,800; $Đ$ = 1.49.

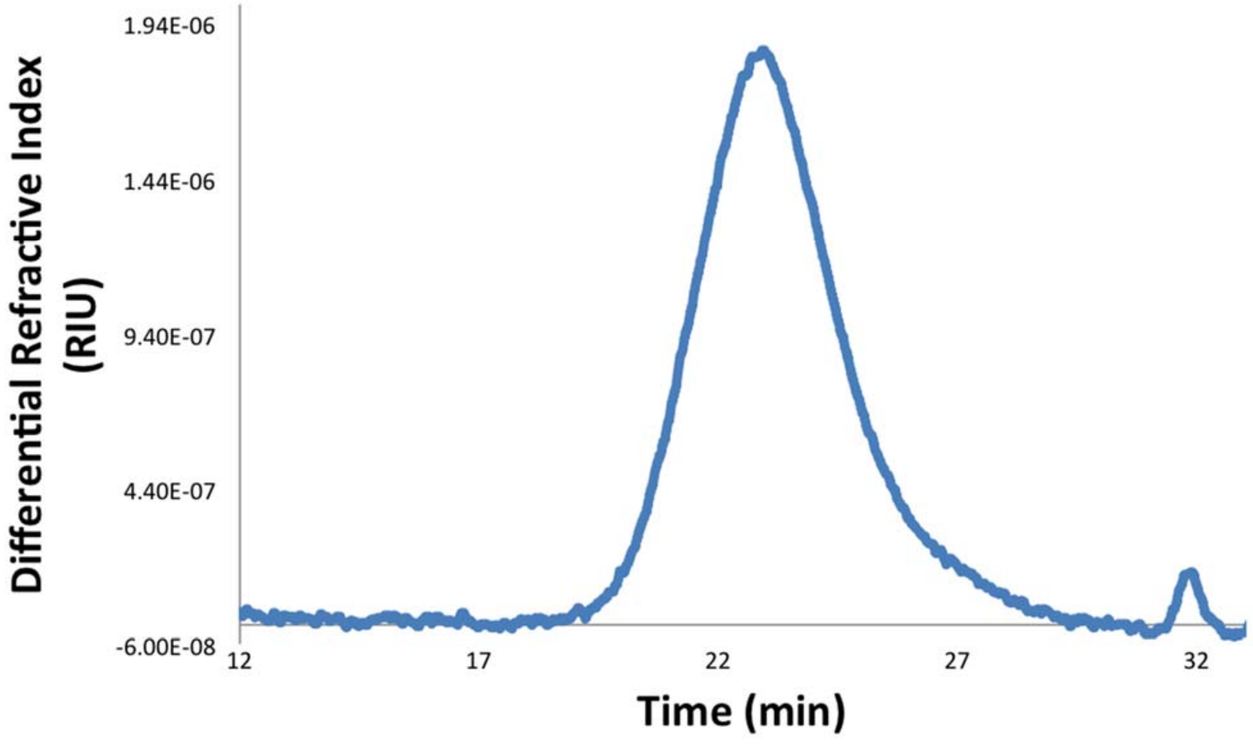

Figure S55. SEC trace of PTMC-*b*-PLA-*b*-PTMC-*b*-PLA-*b*-PTMC copolymer (Table 1, entry 8); M_n = 58,900; M_w = 87,900; $Đ$ = 1.49.

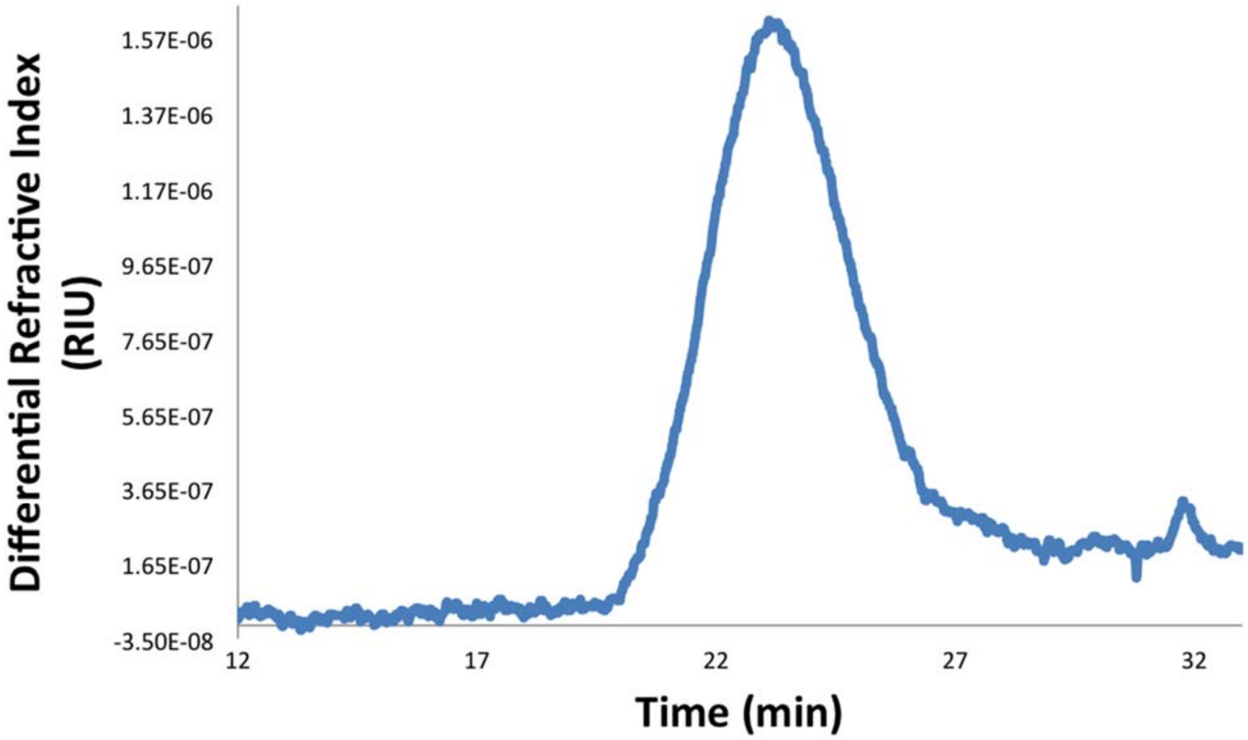

Figure S56. SEC trace of PLA-*b*-PTMC-*b*-PLA-*b*-PTMC-*b*-PLA copolymer (Table 1, entry 9); M_n = 53,200; M_w = 89,700; *Đ* = 1.69.

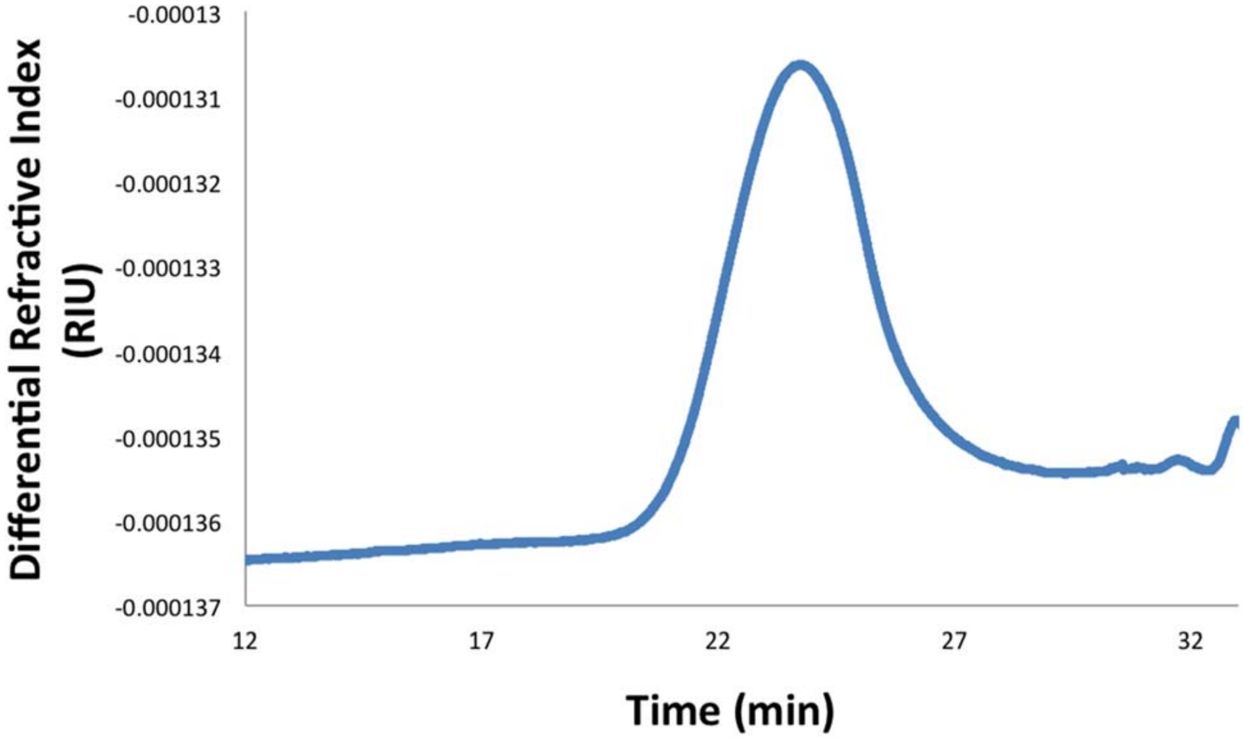

Figure S57. SEC trace of PLA-*b*-PTMC-*b*-PLA copolymer (Table 1, entry 10); M_n = 50,800; M_w = 65,800; *Đ* = 1.29.

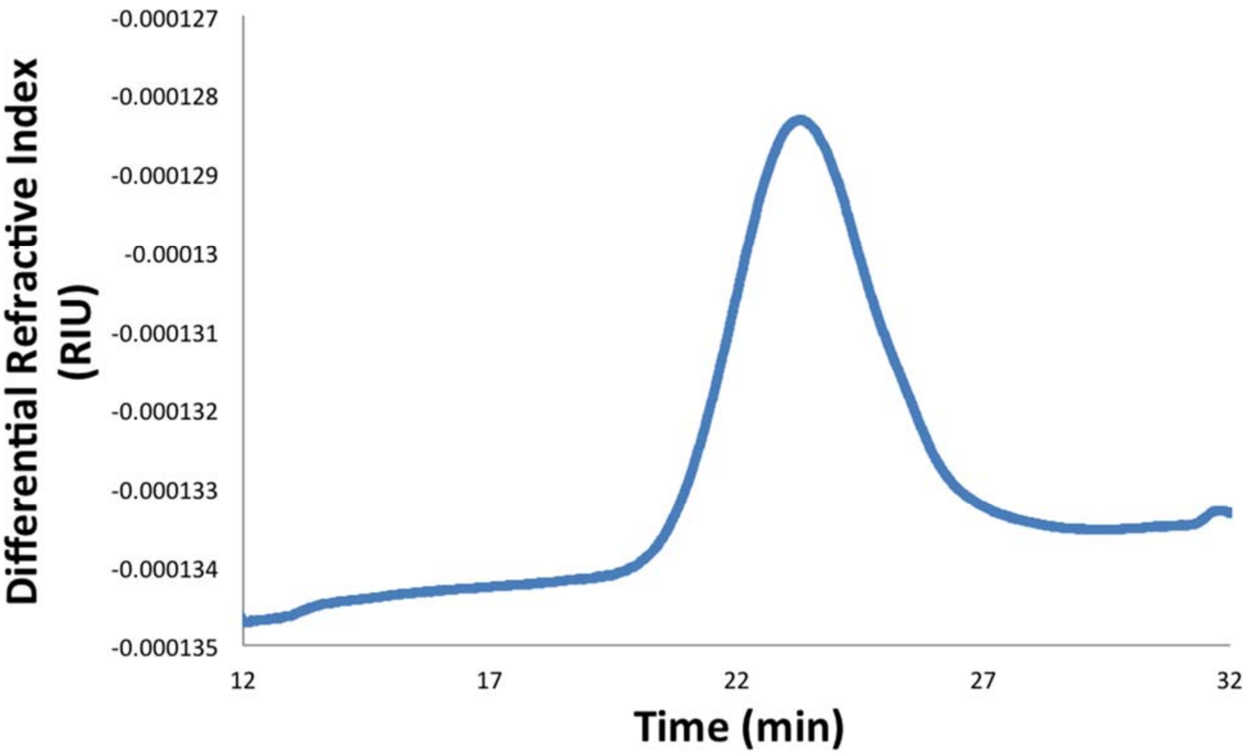

Figure S58. SEC trace of PLA-*b*-PTMC-*b*-PLA copolymer (Table 1, entry 11); M_n = 48,900; M_w = 69,400; $Đ$ = 1.42.

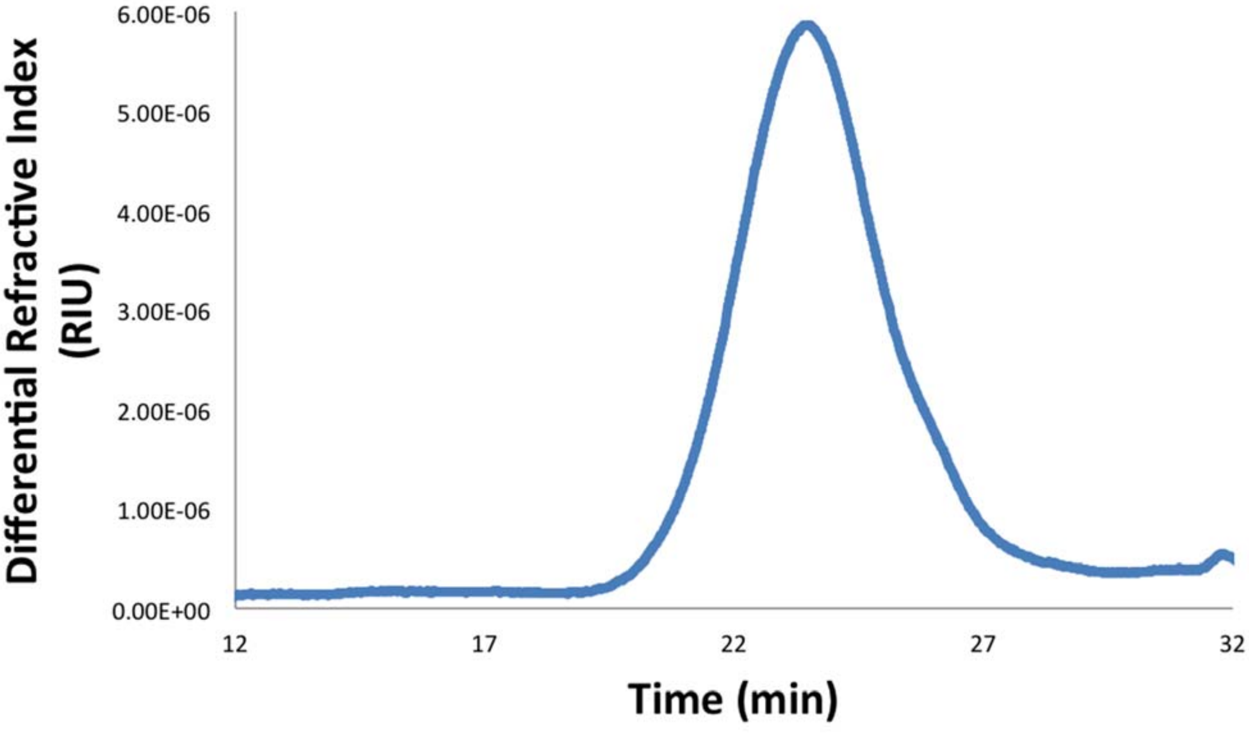

Figure S59. SEC trace of PLA-*b*-PTMC-*b*-PLA copolymer (Table 1, entry 12); M_n = 51,200; M_w = 86,000; $Đ$ = 1.68.

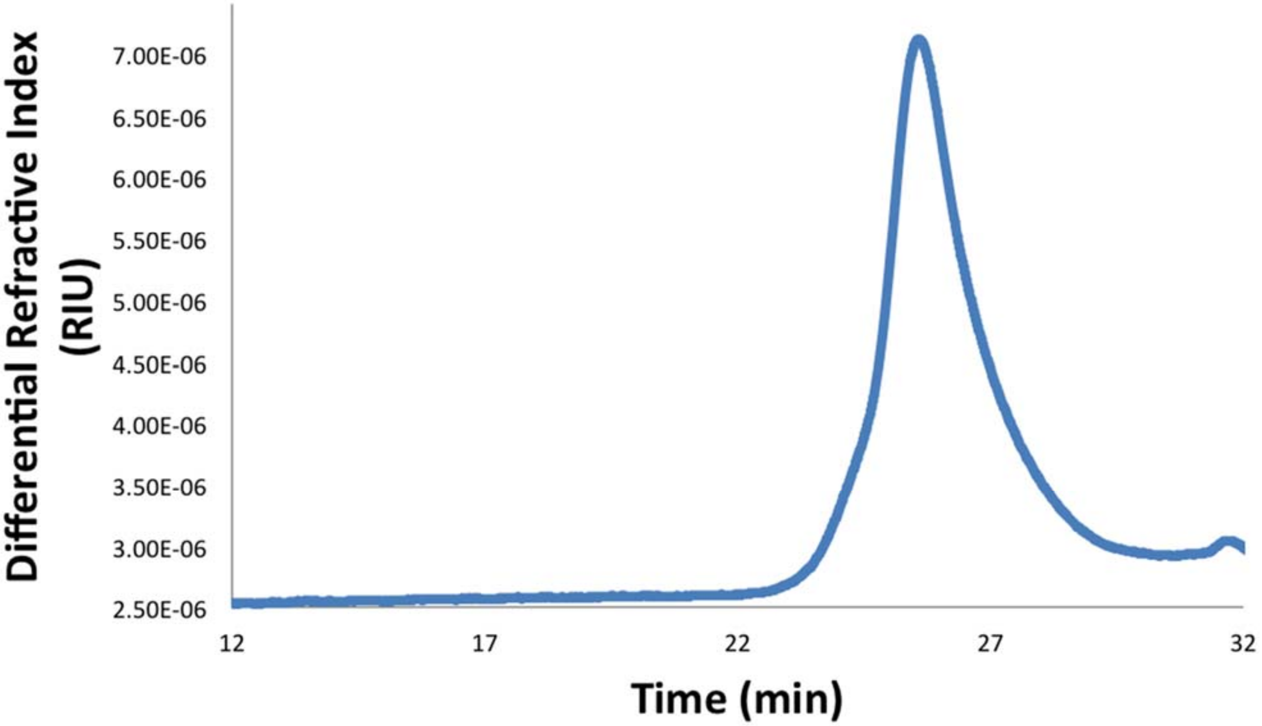

Figure S60. SEC trace of PLA polymer obtained by melt polymerization; M_n = 25,800; M_w = 29,800; *Đ* = 1.16.

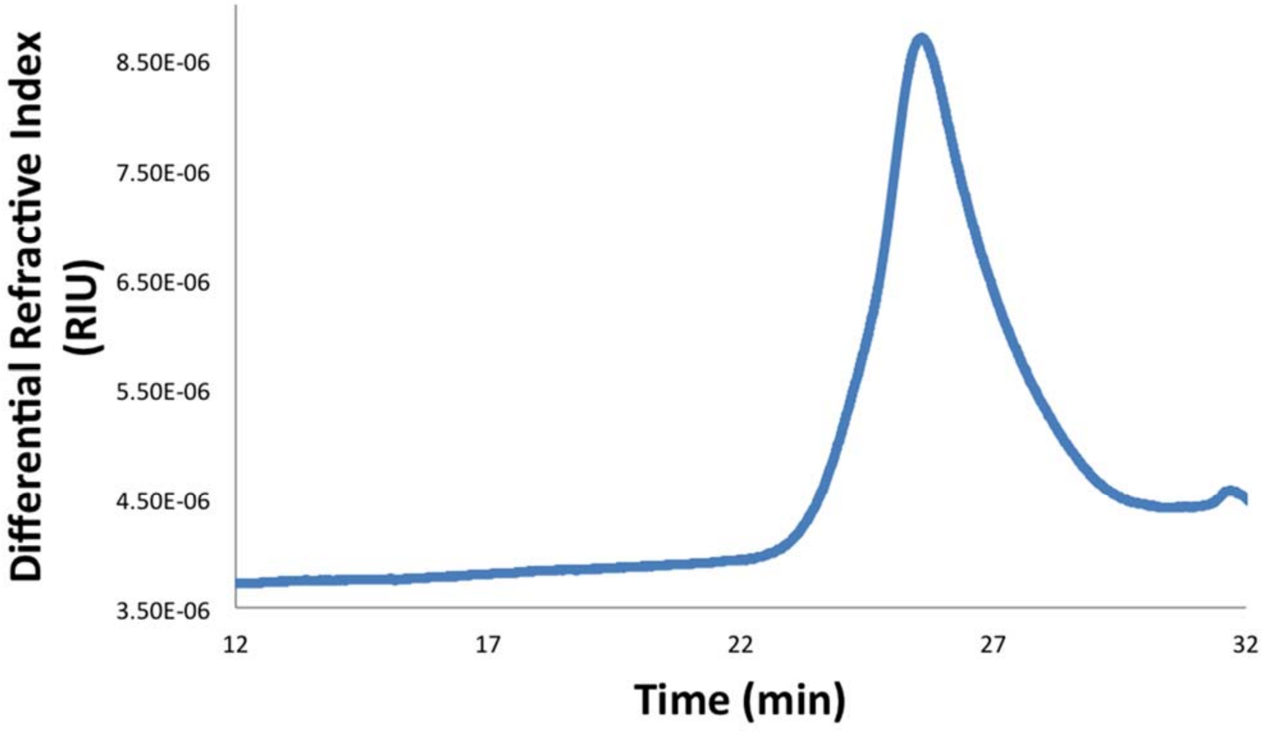

Figure S61. SEC trace of PTMC-*b*-PLA copolymer obtained by melt polymerization; M_n = 24,800; M_w = 29,000; *Đ* = 1.17.

Differential Scanning Calorimetry

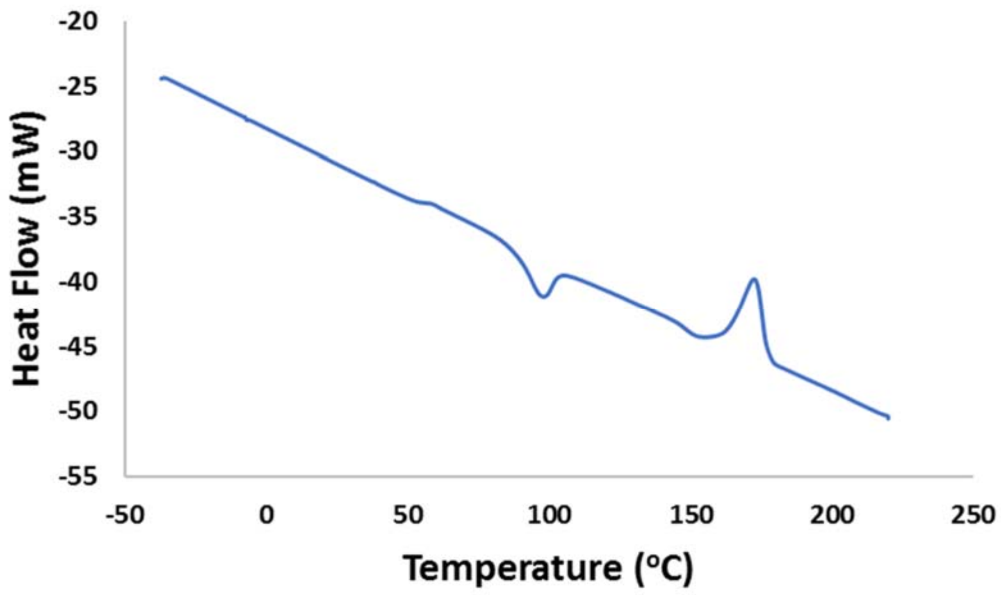

Figure S62. DSC curve (third heating run) of a PLA sample (M_n (SEC) = 39,800; Table 1, entry 1).

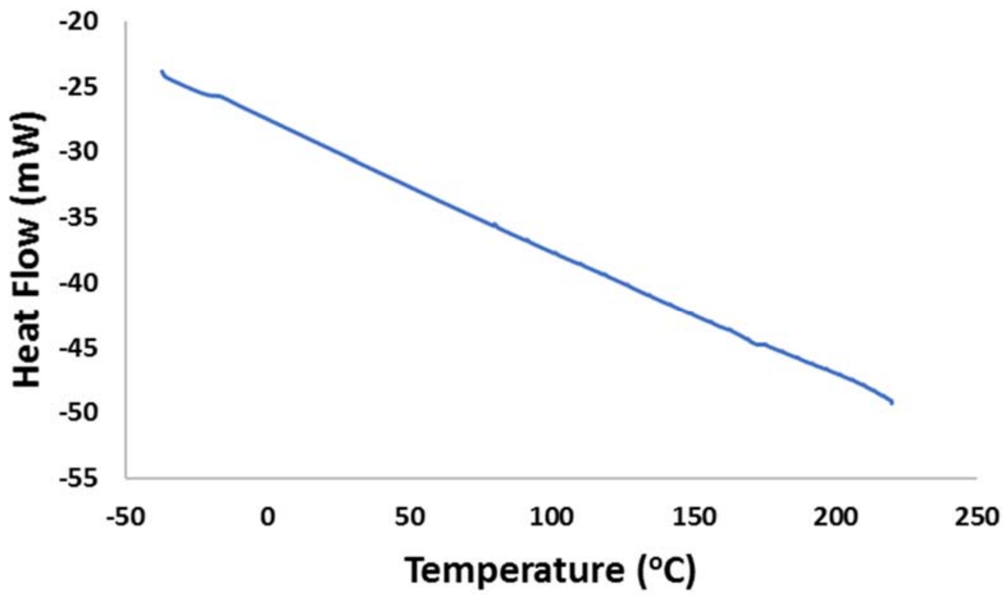

Figure S63. DSC curve (third heating run) of a PTMC sample (M_n (SEC) = 9,000; Table 1, entry 2).

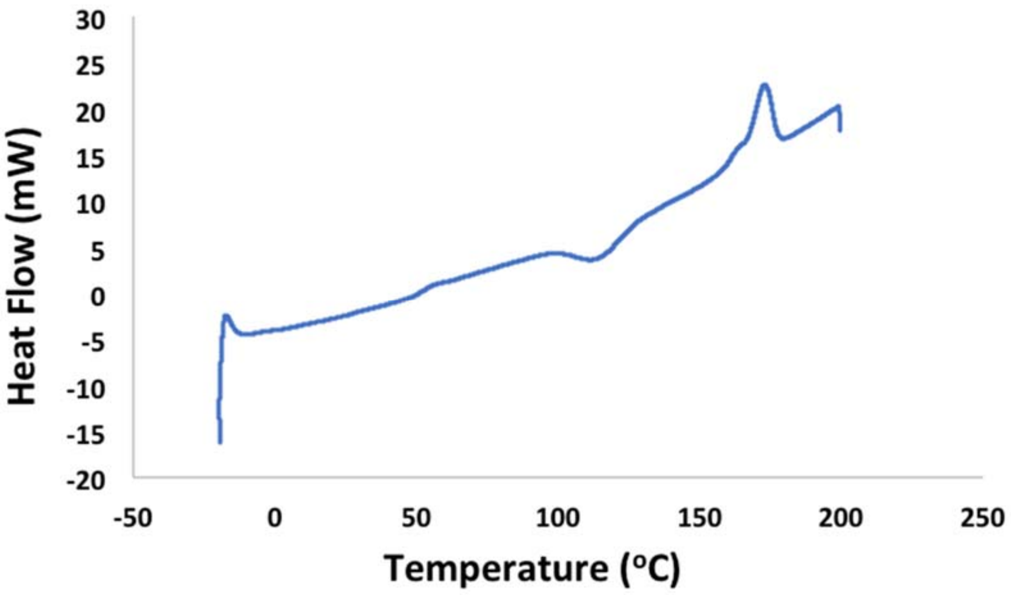

Figure S64. DSC curve (third heating run) of a PLA-*b*-PTMC sample (M_n (SEC) = 55,500; Table 1, entry 3).

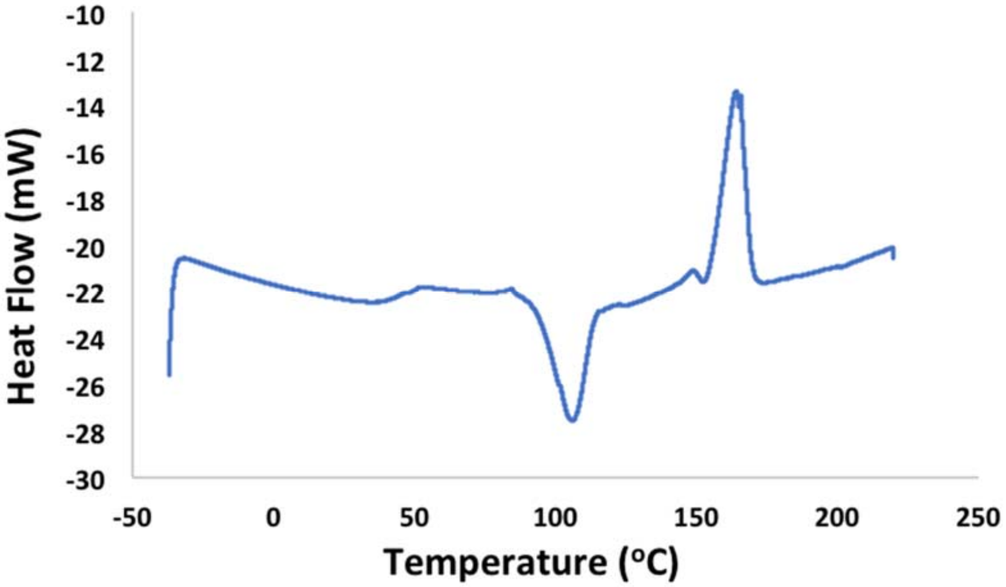

Figure S65. DSC curve (third heating run) of a PTMC-*b*-PLA sample (M_n (SEC) = 47,000; Table 1, entry 4).

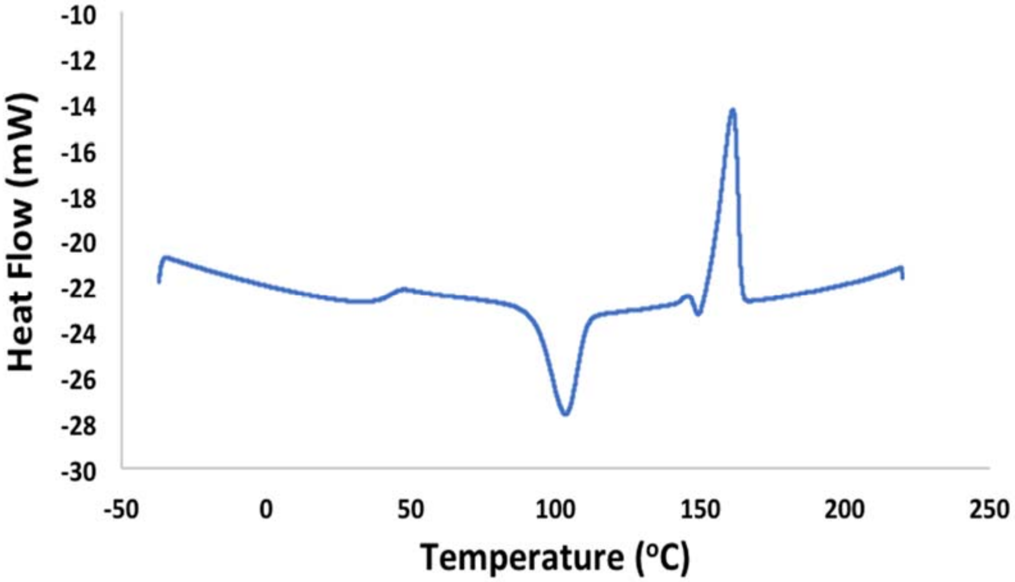

Figure S66. DSC curve (third heating run) of a PTMC-*b*-PLA-*b*-PTMC sample (M_n (SEC) = 43,200; Table 1, entry 5).

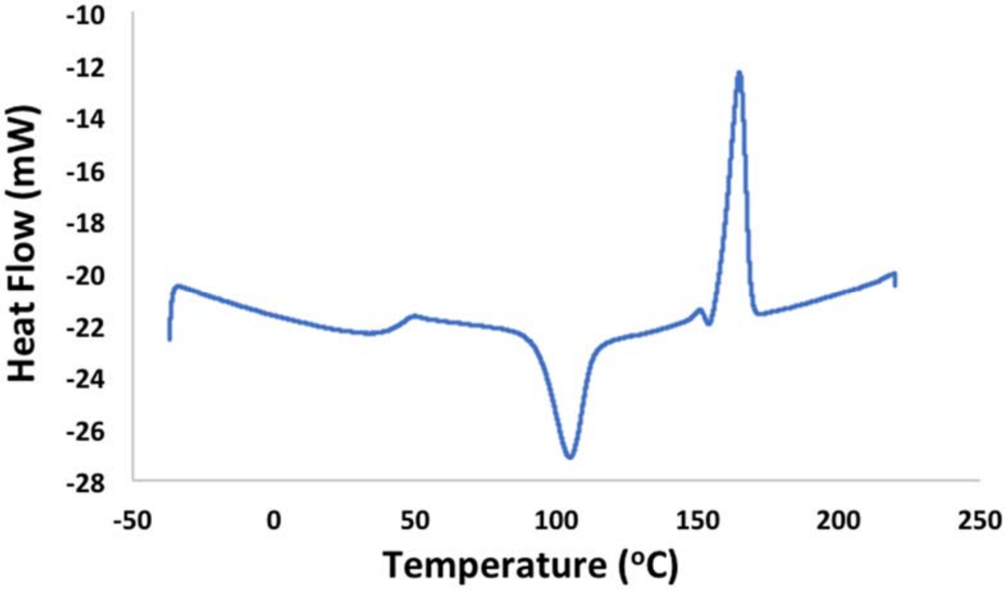

Figure S67. DSC curve (third heating run) of a PLA-*b*-PTMC-*b*-PLA sample (M_n (SEC) = 55,600; Table 1, entry 6).

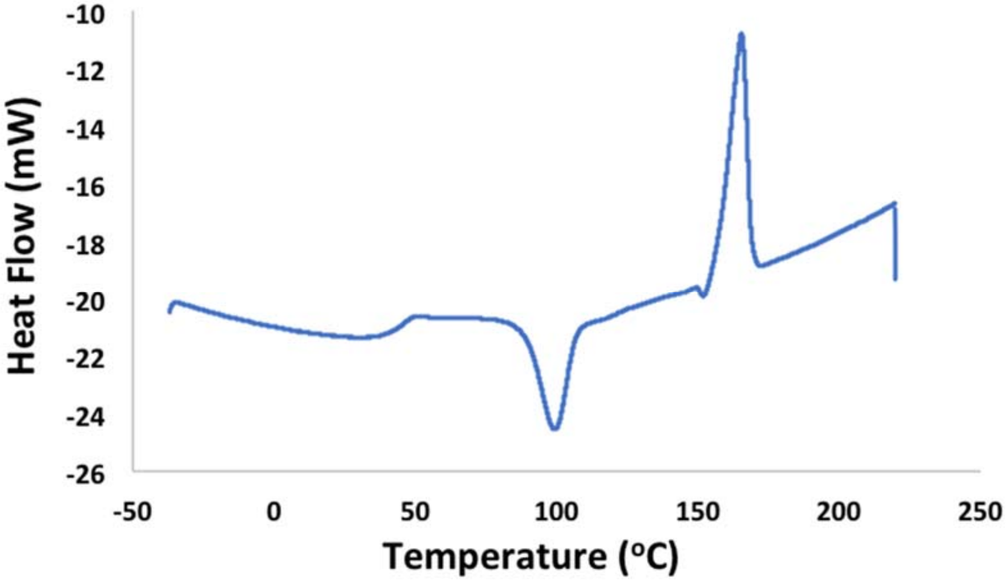

Figure S68. DSC curve (third heating run) of a PLA-*b*-PTMC-*b*-PLA-*b*-PTMC sample (M_n (SEC) = 48,200; Table 1, entry 7).

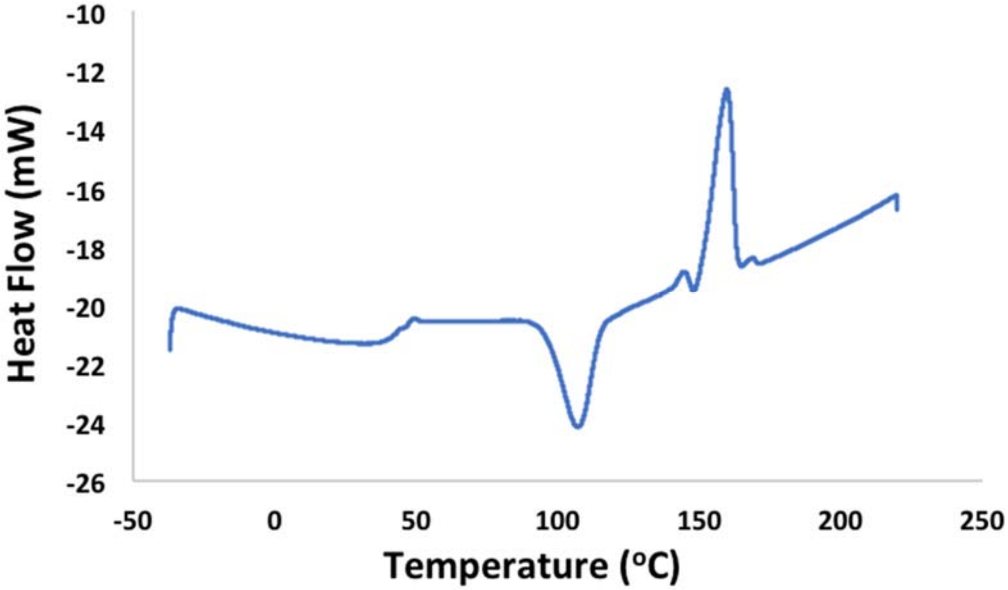

Figure S69. DSC curve (third heating run) of a PTMC-*b*-PLA-*b*-PTMC-*b*-PLA-*b*-PTMC sample (M_n (SEC) = 58,900; Table 1, entry 8).

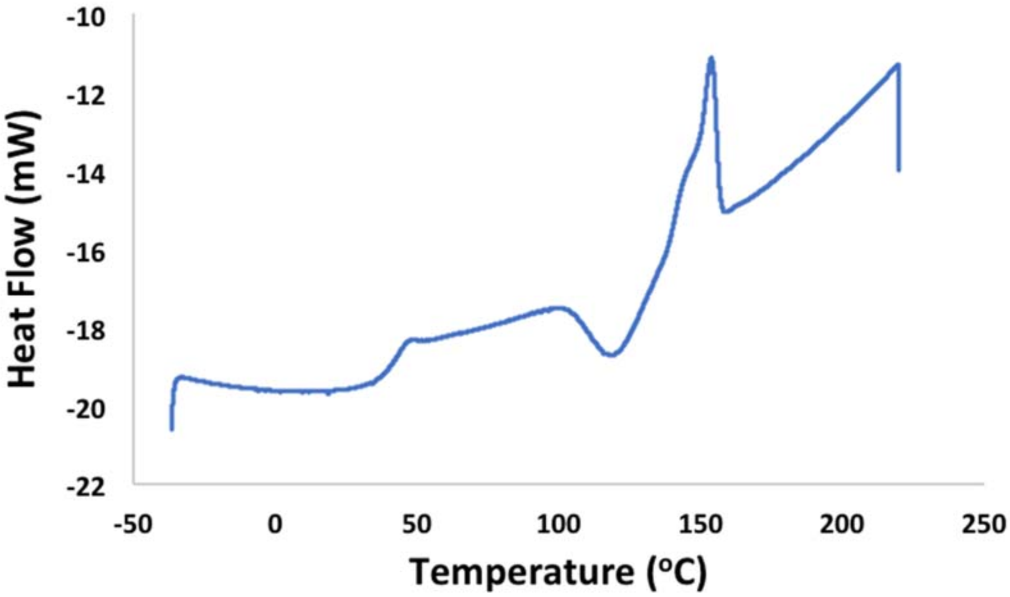

Figure S70. DSC curve (third heating run) of a PLA-*b*-PTMC-*b*-PLA-*b*-PTMC-*b*-PLA sample (M_n (SEC) = 53,200; Table 1, entry 9).

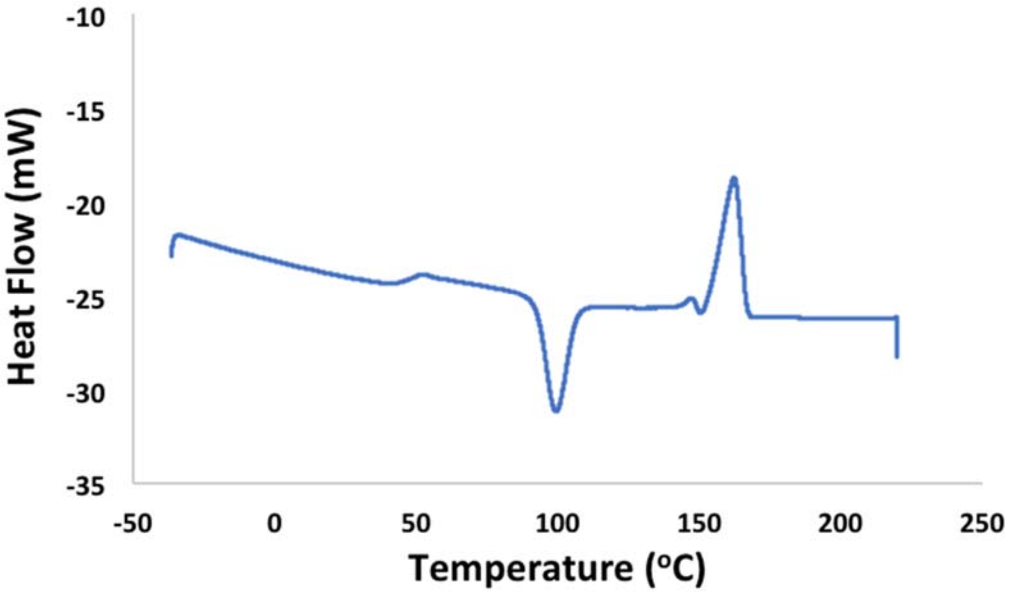

Figure S71. DSC curve (third heating run) of a PLA-*b*-PTMC-*b*-PLA sample (M_n (SEC) = 50,800; Table 1, entry 10).

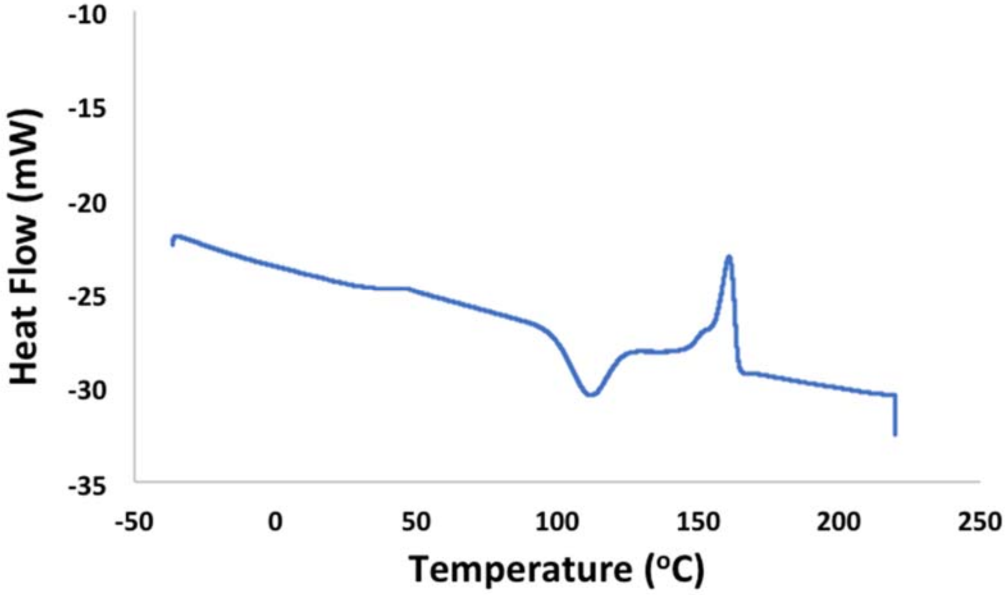

Figure S72. DSC curve (third heating run) of a PLA-*b*-PTMC-*b*-PLA sample (M_n (SEC) = 48,900; Table 1, entry 11).

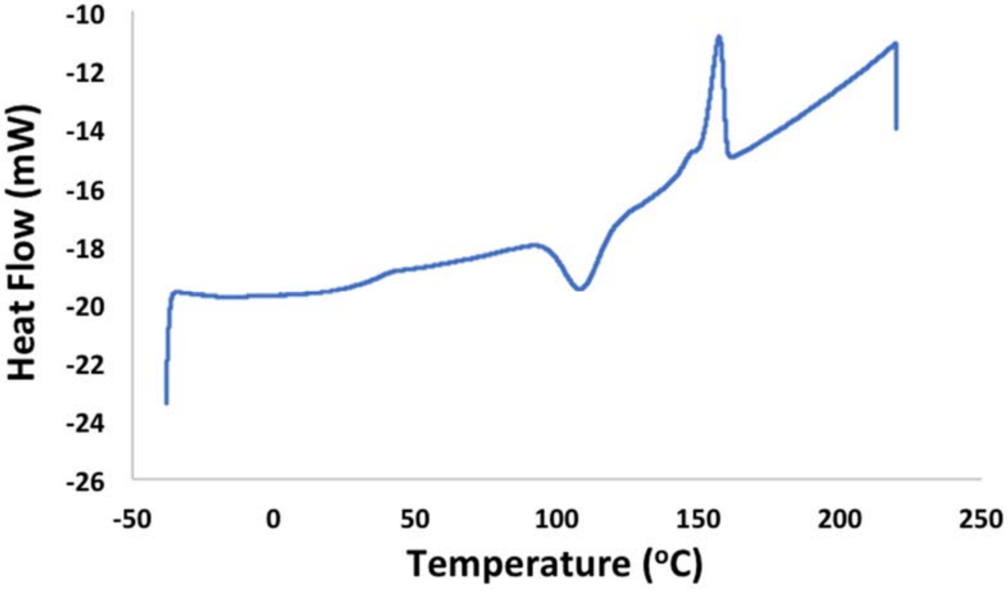

Figure S73. DSC curve (third heating run) of a PLA-*b*-PTMC-*b*-PLA sample (M_n (SEC) = 51,200; Table 1, entry 12).

Dynamic Mechanical Analysis

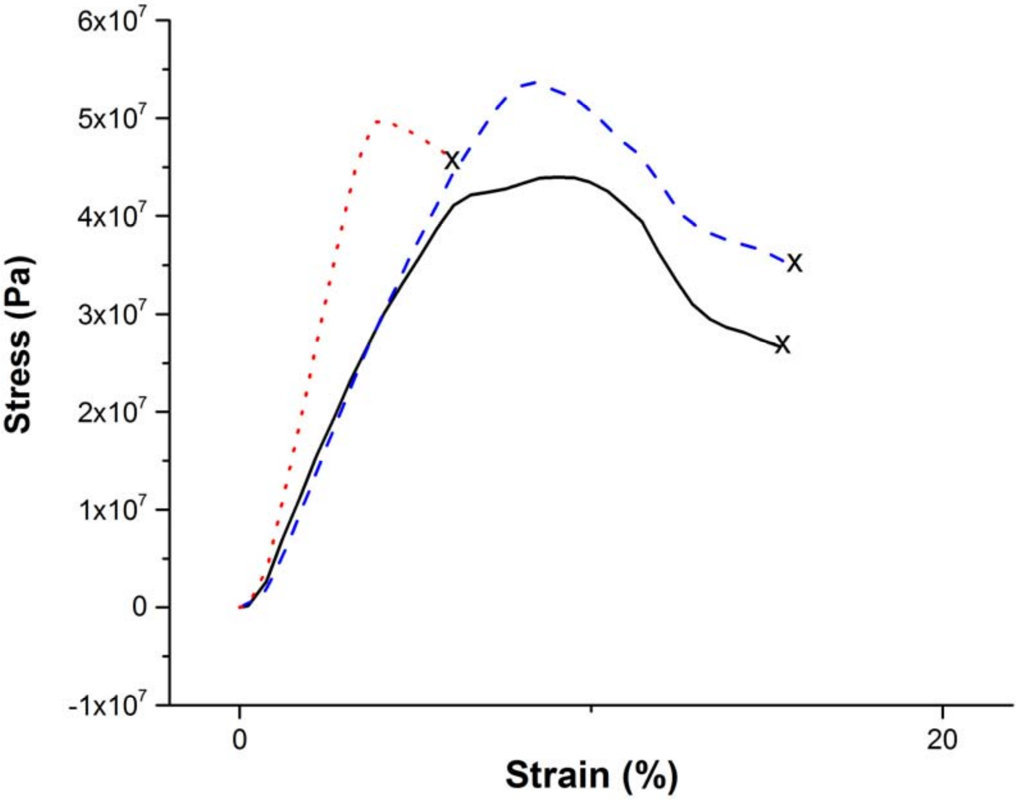

Figure S74. Stress vs. strain curve of a PLA sample (M_n (SEC) = 39,800; Table 1, entry 2).

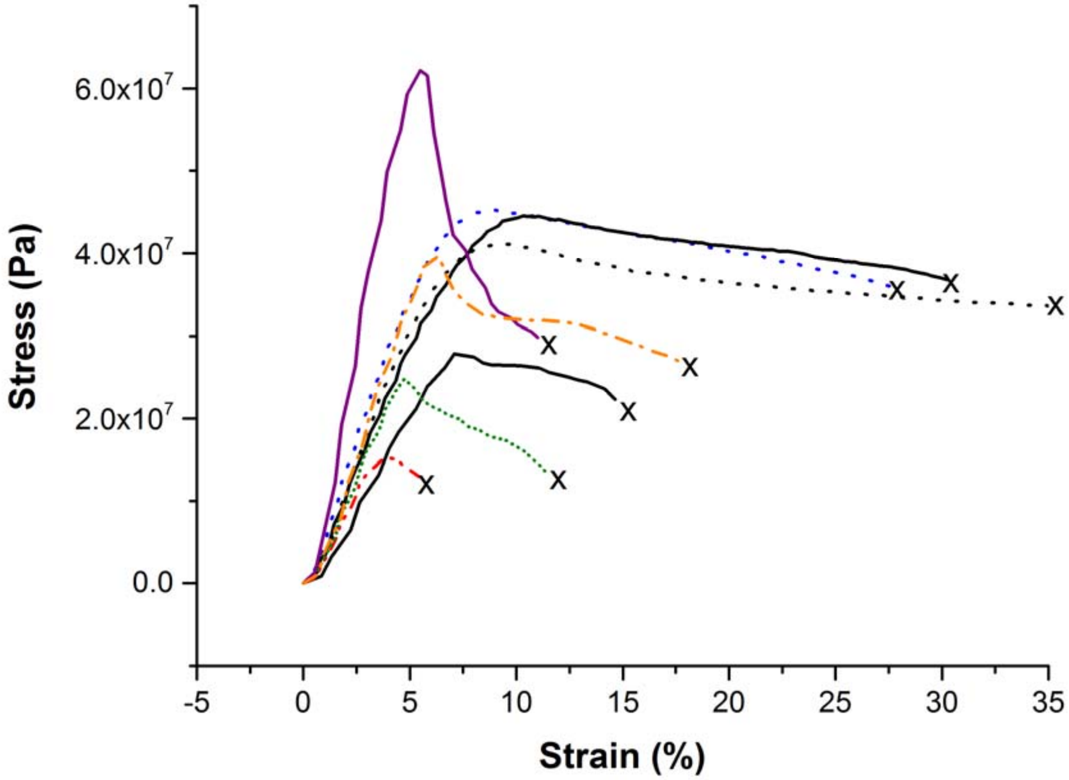

Figure S75. Stress vs. strain curve of a PLA-*b*-PTMC sample (M_n (SEC) = 55,500; Table 1, entry 3) and PLA-*b*-PTMC sample (M_n (SEC) = 53,400).

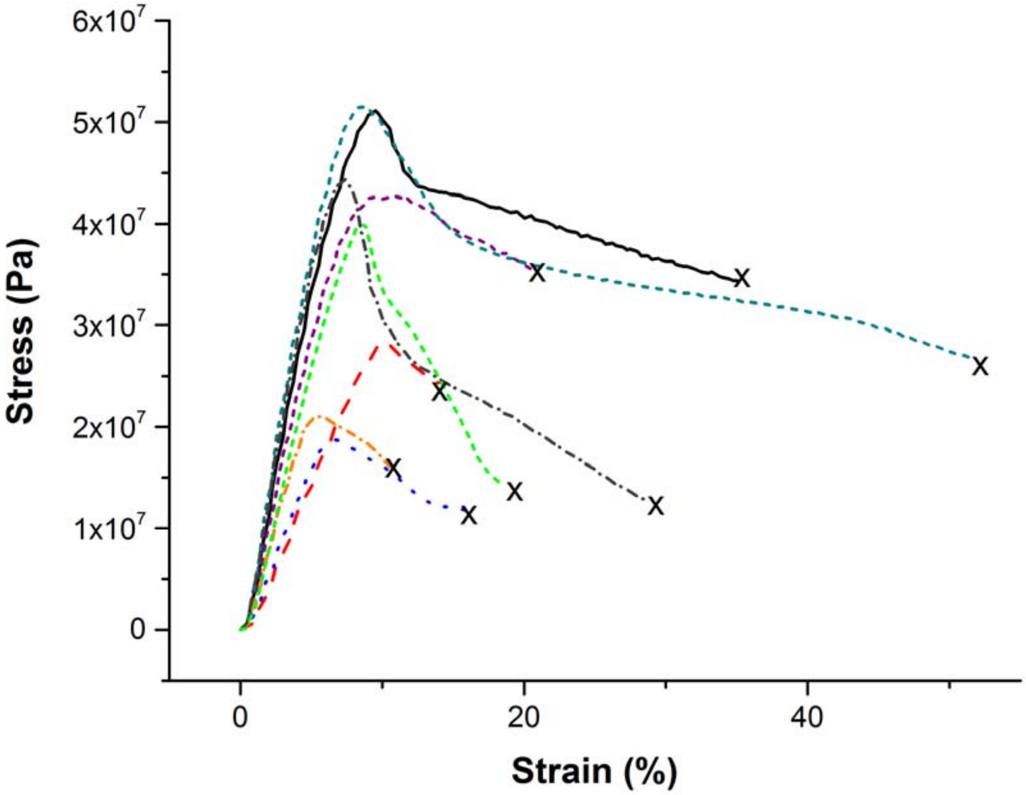

Figure S76. Stress vs. strain curve of a PTMC-*b*-PLA sample (M_n (SEC) = 47,000; Table 1, entry 4) and PTMC-*b*-PLA sample (M_n (SEC) = 59,000).

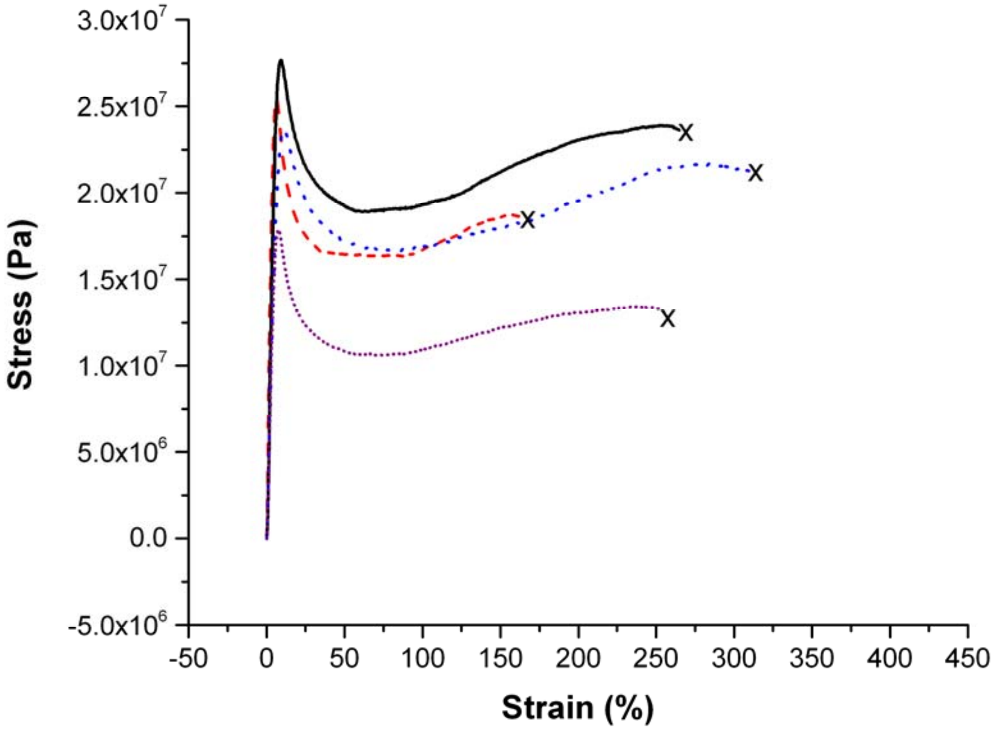

Figure S77. Stress vs. strain curve of a PTMC-*b*-PLA-*b*-PTMC sample (M_n (SEC) = 43,200; Table 1, entry 5).

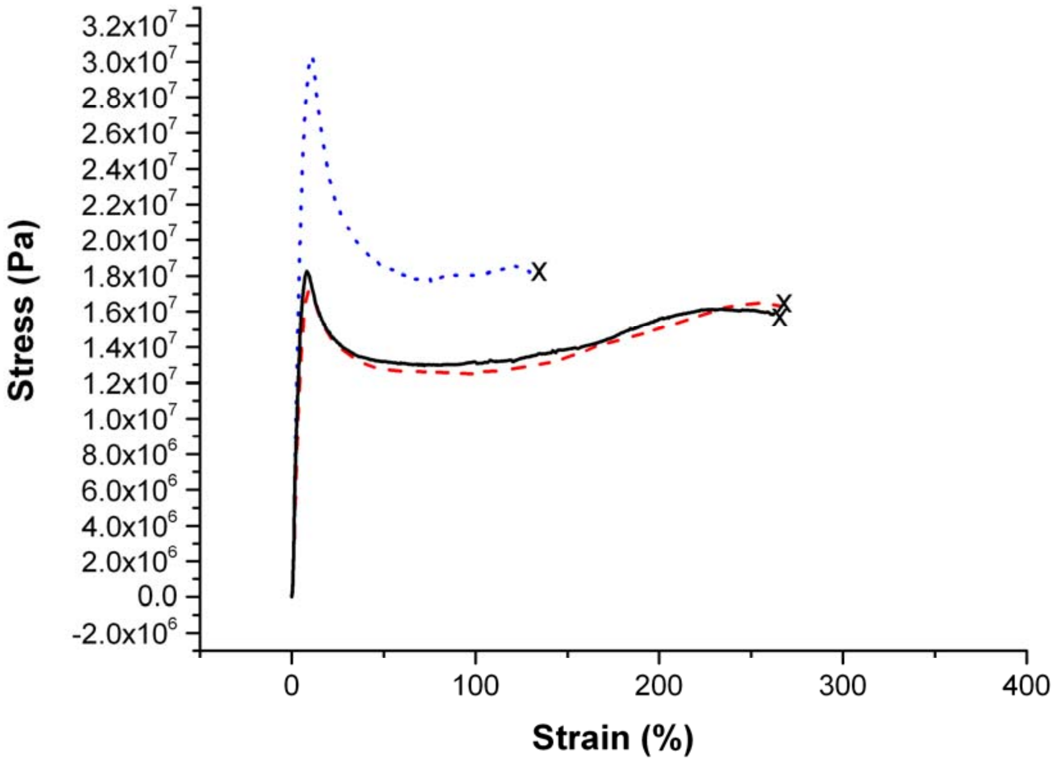

Figure S78. Stress vs. strain curve of a PLA-*b*-PTMC-*b*-PLA sample (M_n (SEC) = 55,600; Table 1, entry 6).

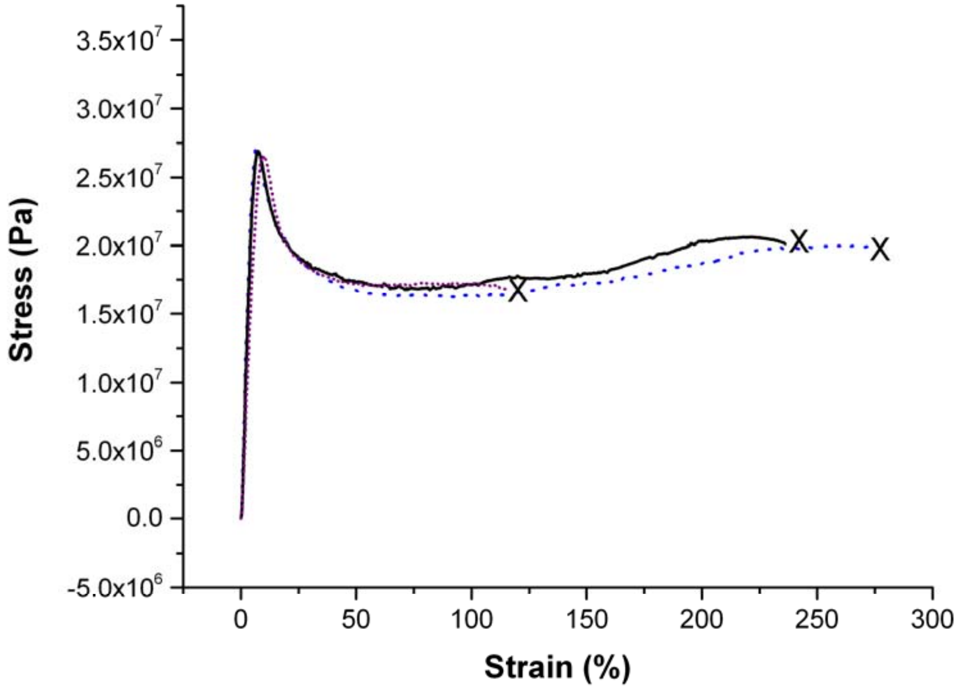

Figure S79. Stress vs. strain curve of a PTMC-*b*-PLA-*b*-PTMC-*b*-PLA sample (M_n (SEC) = 48,200; Table 1, entry 7).

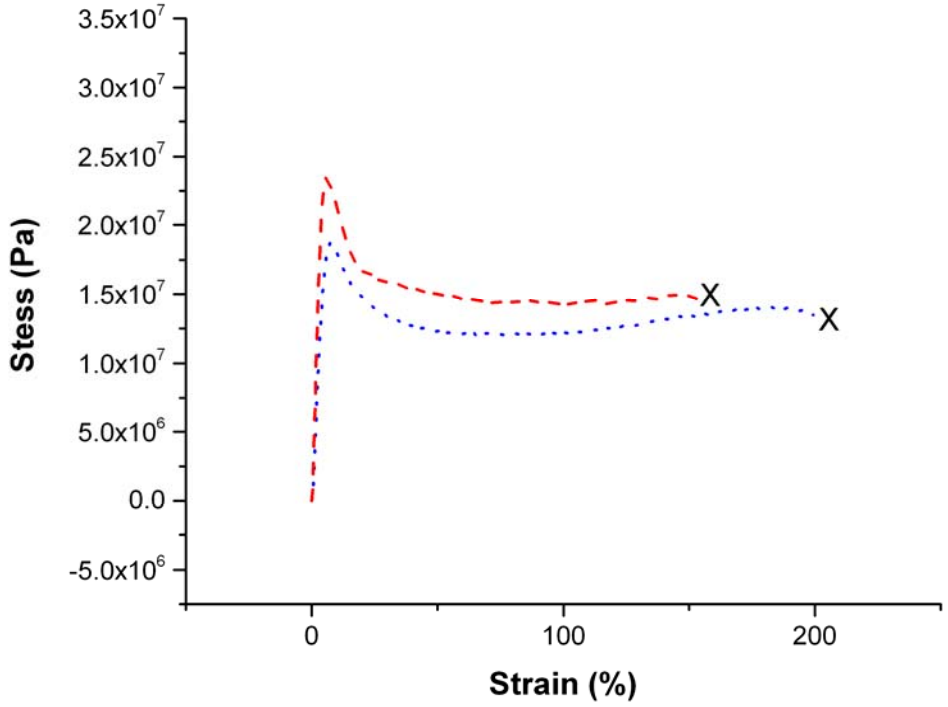

Figure S80. Stress vs. strain curve of a PTMC-*b*-PLA-*b*-PTMC-*b*-PLA-*b*-PTMC sample (M_n (SEC) = 58,900; Table 1, entry 8).

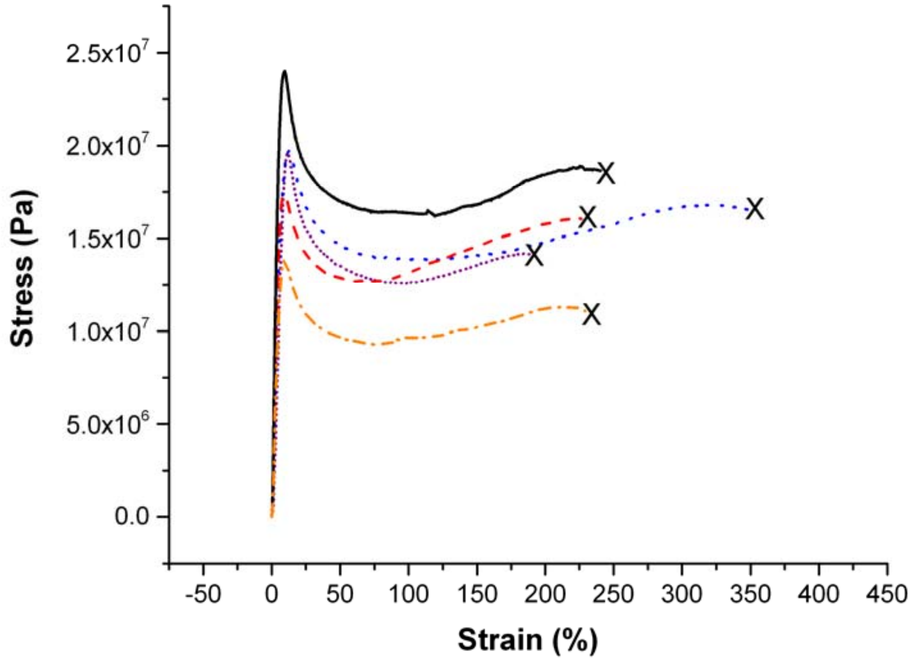

Figure S81. Stress vs. strain curve of a PLA-*b*-PTMC-*b*-PLA-*b*-PTMC-*b*-PLA sample (M_n (SEC) = 53,200; Table 1, entry 9).

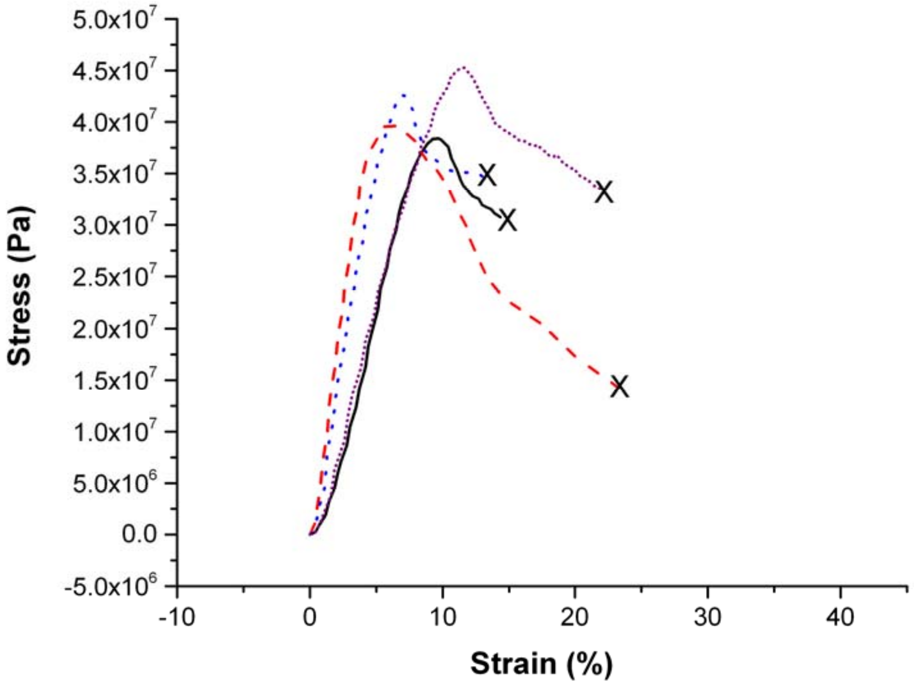

Figure S82. Stress vs. strain curve of a PLA-*b*-PTMC-*b*-PLA sample (M_n (SEC) = 50,800; Table 1, entry 10).

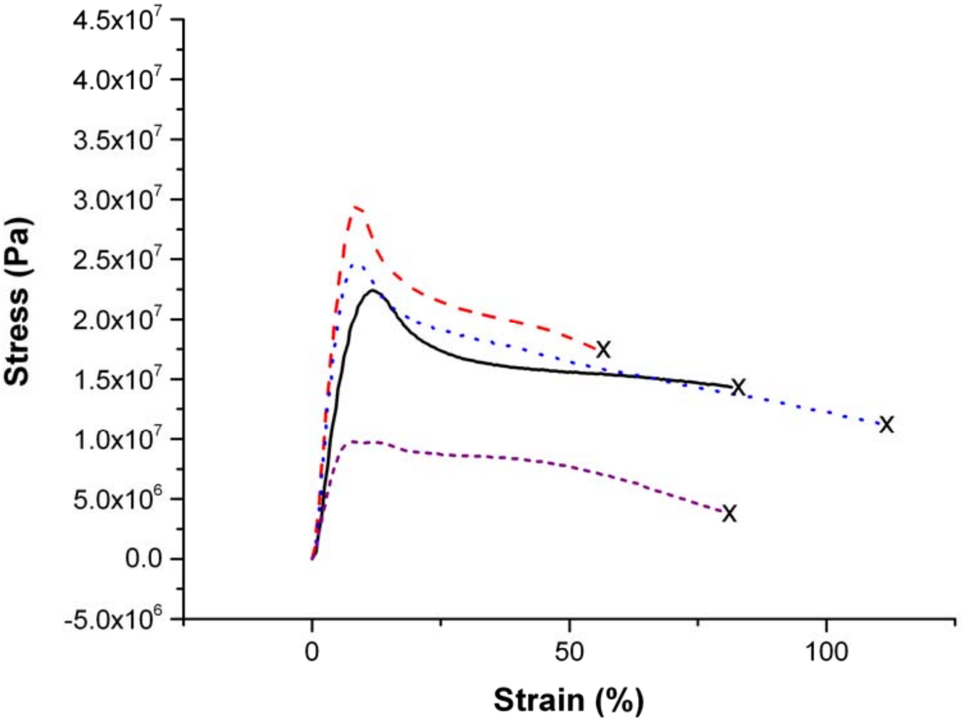

Figure S83. Stress vs. strain curve of a PLA-*b*-PTMC-*b*-PLA sample (M_n (SEC) = 48,900; Table 1, entry 11).

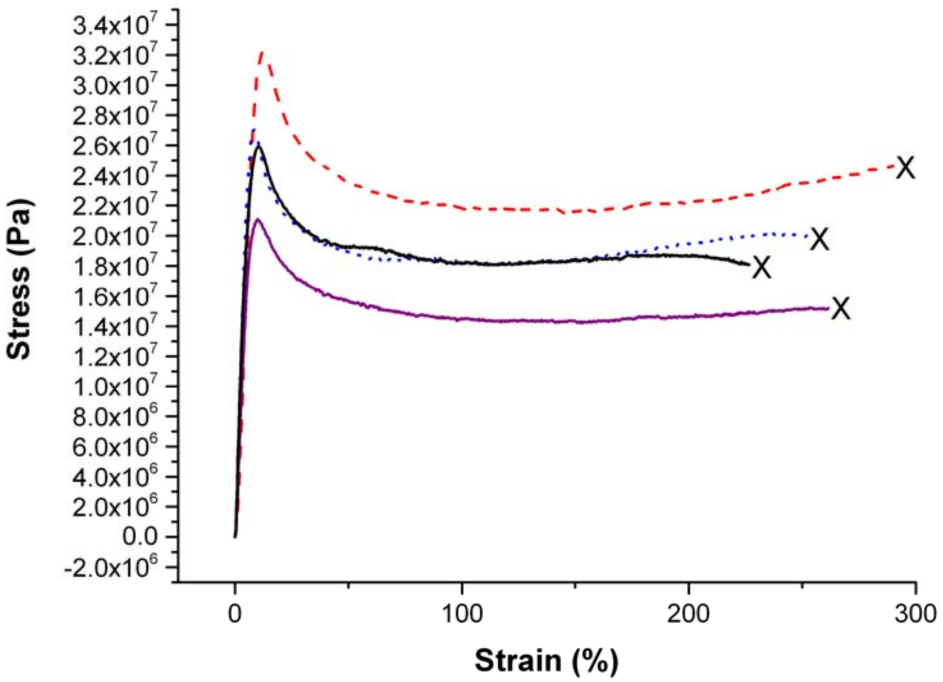

Figure S84. Stress vs. strain curve of a PLA-*b*-PTMC-*b*-PLA sample (M_n (SEC) = 51,200; Table 1, entry 12).

X-ray Crystallographic Data

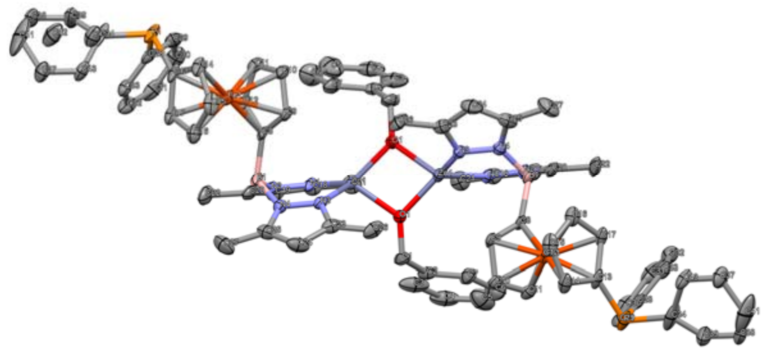

Figure S85. Molecular structure drawing of $[(fc^{P,B})Zn(\mu\text{-}OCH_2Ph)]_2$ with thermal ellipsoids at 50% probability; hydrogen atoms are omitted for clarity. Selected distances (Å) and angles (°): N(1)-Zn(1), 1.970(4); N(3)-Zn(1), 2.019(4); O(1)-Zn(1), 1.946(3), 1.981(4); N(1)-Zn(1)-N(3), 99.44(17); O(1)-Zn(1)-O(1) 80.26(16); N(3)-Zn(1)-O(1), 104.43(16), 131.00(17); N(1)-Zn(1)-O(1), 118.28(17), 124.18(16).

Crystal data for $C_{78}H_{78}B_2Fe_2N_8O_2P_2Zn_2$; M_r = 1485.48; Monoclinic; space group P2/c; a = 13.855(4) Å; b = 9.378(2) Å; c = 30.742(8) Å; α = 90°; β = 101.648(3)°; γ = 90°; V = 3912.1(17) $Å^3$; Z = 2; T = 100(2) K; λ = 0.71073 Å; μ = 1.058 mm^{-1}; d_{calc} = 1.261$g\cdot cm^{-3}$; 39551 reflections collected; 9416 unique (R_{int} = 0.0667); giving R_1 = 0.0797, wR_2 = 0.2105 for 7205 data with [I>2σ(I)] and R_1 = 0.0983, wR_2 = 0.2187 for all 9416 data. Residual electron density ($e^{-}\cdot Å^{-3}$) max/min: 1.873/-1.188.

DFT Calculations

The full reference for the GAUSSIAN 09[2] program package can be found at the end of the document.

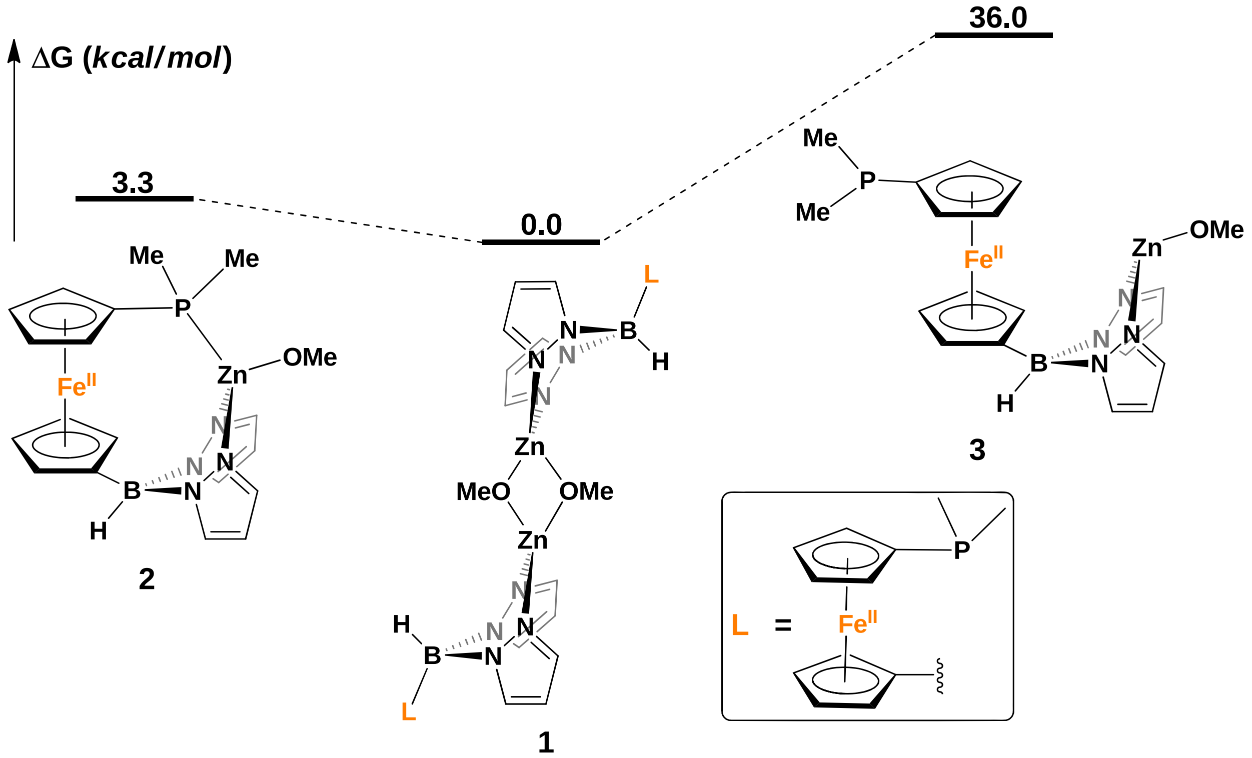

Figure S86. Comparison between the energies of the dimeric zinc complex and two monomeric forms.

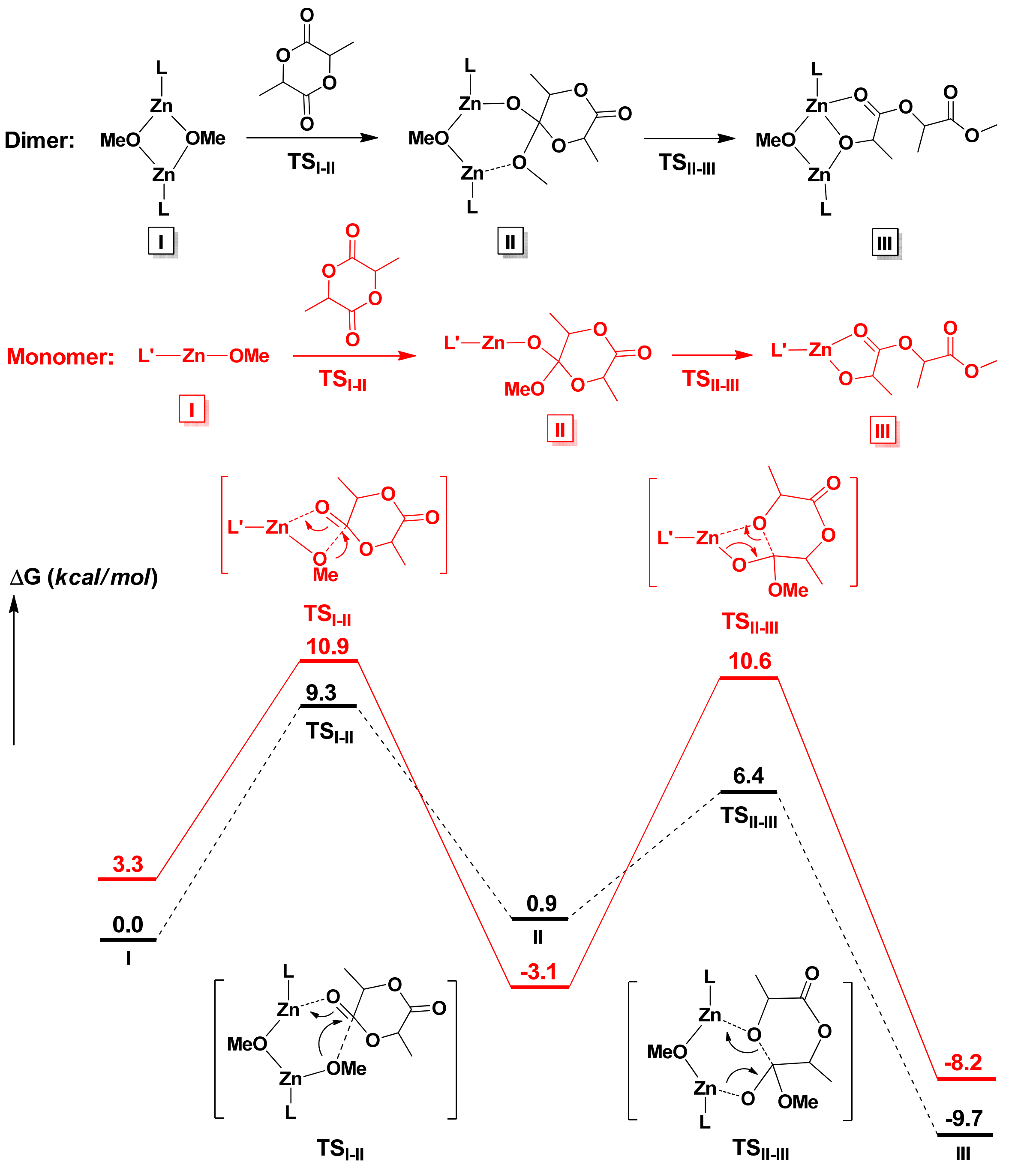

Figure S87. Comparison of the initiation steps of LA polymerization catalyzed by a monomeric (red) or dimeric (black) form of the zinc complex.

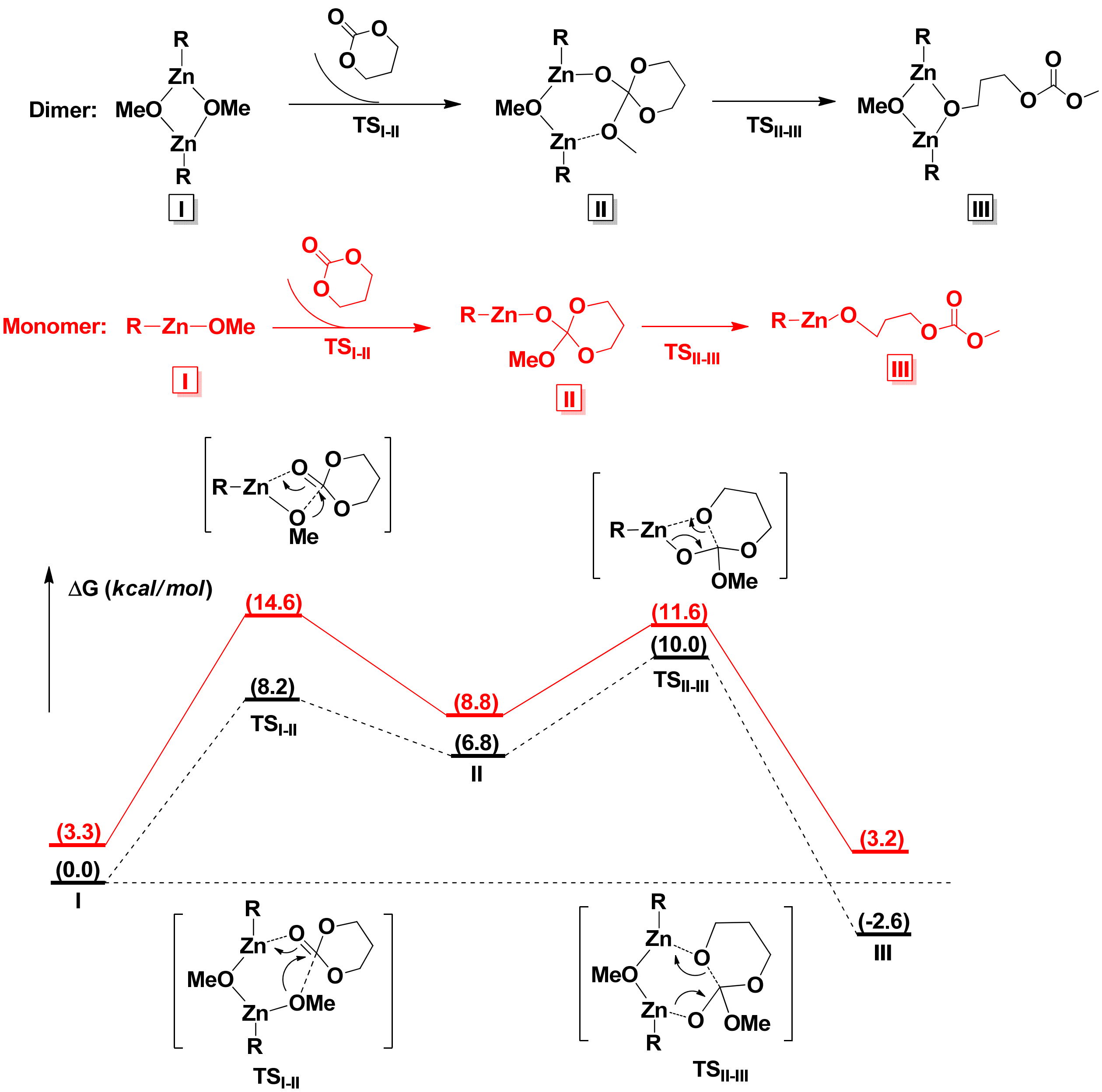

Figure S88. Comparison of the initiation steps of TMC polymerization catalyzed by a monomeric (red) or dimeric (black) form of the zinc complex.

Table S3. Energies, enthalpies, and free energies of the structures calculated at the PBE1PBE/SDD, 6-311+G(d,p) (PCM, GD3, benzene)//B3LYP/LANL2DZ, 6-31G(d) level.

**Structures**	correction of H	correction of G	G	E with corrections	new G with corrections
**lactide**	0.151554	0.105907	-534.250539	-533.935059	-533.829152
**trimethylene carbonate**	0.111273	0.074079	-381.634182	-381.4101659	-381.3360869
**1**	0.875728	0.70951	-3175.387086	-3497.86323	-3497.15372
**2**	0.436398	0.345477	-1587.679245	-1748.919683	-1748.574206
**3**	0.436644	0.344288	-1587.660442	-1748.892456	-1748.548168
**TSI-II^{monomer}-LA**	0.589366	0.478011	-2121.90727	-2282.86919	-2282.391179
**II^{monomer}-LA**	0.591557	0.481052	-2121.921651	-2282.894573	-2282.413521
**TSII-III^{monomer}-LA**	0.58906	0.479528	-2121.905148	-2282.871218	-2282.39169
**III^{monomer}-LA**	0.591123	0.476715	-2121.941604	-2282.898472	-2282.421757
**TSI-II^{dimer}-LA**	1.028291	0.842812	-3709.600812	-4031.810914	-4030.968102
**II^{dimer}-LA**	1.030228	0.846358	-3709.610079	-4031.827784	-4030.981426
**TSII-III^{dimer}-LA**	1.029558	0.838501	-3709.602191	-4031.811084	-4030.972583
**III^{dimer}-LA**	1.030265	0.841752	-3709.641343	-4031.840047	-4030.998295
**TSI-II^{monomer}-TMC**	0.548666	0.446324	-1969.292661	-2130.338613	-2129.892289
**II^{monomer}-TMC**	0.550803	0.447984	-1969.298916	-2130.349591	-2129.901607
**TSII-III^{monomer}-TMC**	0.549183	0.448359	-1969.298147	-2130.34546	-2129.897101
**III^{monomer}-TMC**	0.550702	0.445793	-1969.306127	-2130.356265	-2129.910472
**TSI-II^{dimer}-TMC**	0.988222	0.811889	-3556.991913	-3879.288584	-3878.476695
**II^{dimer}-TMC**	0.989541	0.813922	-3556.994991	-3879.292875	-3878.478953
**TSII-III^{dimer}-TMC**	0.988158	0.811071	-3556.991928	-3879.28486	-3878.473789
**III^{dimer}-TMC**	0.989914	0.804174	-3557.015624	-3879.298082	-3878.493908
**Cat^{monomer}-propagation**	0.514005	0.413133	-1854.818974	-2015.915846	-2015.502713
**TSI-II^{monomer}-propagation-LA**	0.666528	0.54449	-2389.028178	-2549.84817	-2549.30368
**II^{monomer}-propagation-LA**	0.668844	0.546174	-2389.049481	-2549.88321	-2549.337036
**TSII-III^{monomer}-propagation-LA**	0.666657	0.545564	-2389.031229	-2549.876085	-2549.330521
**III^{monomer}-propagation-LA**	0.667879	0.537682	-2389.06437	-2549.876085	-2549.338403

**TSI-II^{monomer}-propagation-TMC**	0.625781	0.511976	-2236.41156	-2397.314263	-2396.802287
**II^{monomer}-propagation-TMC**	0.628223	0.51387	-2236.433189	-2397.347309	-2396.833439
**TSII-III^{monomer}-propagation-TMC**	0.626479	0.512416	-2236.420035	-2397.330991	-2396.818575
**III^{monomer}-propagation-TMC**	0.62831	0.511968	-2236.4261	-2397.341203	-2396.829235
**Cat^{dimer}-propagation**	0.923049	0.750067	-3403.230614	-3725.575668	-3724.825601
**TSI-II^{dimer}-propagation-LA**	1.076096	0.88247	-3937.437068	-4259.51908	-4258.63661
**II^{dimer}-propagation-LA**	1.077715	0.885663	-3937.440182	-4259.526353	-4258.64069
**TSII-III^{dimer}-propagation-LA**	1.075994	0.882083	-3937.428402	-4259.513895	-4258.631812
**III^{dimer}-propagation-LA**	1.077382	0.883962	-3937.432711	-4259.521379	-4258.637417
**IV^{dimer}-propagation-LA**	1.077412	0.87887	-3937.459297	-4259.52969	-4258.65082
**TSI-II^{dimer}-propagation-TMC**	1.035662	0.849595	-3784.822131	-4106.984541	-4106.134946
**II^{dimer}-propagation-TMC**	1.037218	0.850853	-3784.826982	-4106.991026	-4106.140173
**TSII-III^{dimer}-propagation-TMC**	1.035811	0.848165	-3784.826779	-4106.991833	-4106.143668
**III^{dimer}-propagation-TMC**	1.036798	0.847828	-3784.829799	-4106.99265	-4106.144822
**IV^{dimer}-propagation-TMC**	1.037851	0.844254	-3784.843767	-4106.998055	-4106.153801

Cartesian coordinates for optimized structures

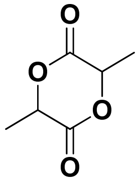

lactide

Standard orientation:

```
 ---------------------------------------------------------------------
 Center     Atomic      Atomic             Coordinates (Angstroms)
 Number      Number       Type             X           Y           Z
 ---------------------------------------------------------------------
      1          8           0       -0.251339   -1.378080   -0.499932
      2          6           0       -1.320763   -0.564515   -0.401020
```

3	6	0	-1.082296	0.923033	-0.165678
4	8	0	0.251335	1.378074	-0.499947
5	6	0	1.320759	0.564510	-0.401039
6	6	0	1.082295	-0.923036	-0.165680
7	8	0	-2.440962	-1.003075	-0.511489
8	6	0	-1.448013	1.348328	1.258052
9	8	0	2.440958	1.003075	-0.511486
10	6	0	1.448023	-1.348318	1.258050
11	1	0	1.727506	-1.445788	-0.875621
12	1	0	1.312576	-2.429144	1.358689
13	1	0	2.494593	-1.098641	1.456161
14	1	0	0.821493	-0.848560	2.004123
15	1	0	-2.494581	1.098654	1.456174
16	1	0	-0.821477	0.848576	2.004124
17	1	0	-1.312563	2.429154	1.358681
18	1	0	-1.727512	1.445780	-0.875618

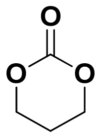

trimethylene carbonate

Standard orientation:

Center Number	Atomic Number	Atomic Type	Coordinates (Angstroms) X	Y	Z
1	6	0	-1.681521	0.000052	-0.395248
2	6	0	-1.012713	-1.221142	0.206605
3	8	0	0.415311	-1.167911	0.027101
4	6	0	1.103441	-0.000006	-0.010043
5	8	0	0.415323	1.167920	0.026896
6	6	0	-1.012638	1.221111	0.206784
7	8	0	2.299282	-0.000024	-0.106276
8	1	0	-1.224172	-1.294153	1.281504
9	1	0	-1.327389	-2.152966	-0.269310
10	1	0	-2.754012	0.000070	-0.170748
11	1	0	-1.561868	0.000156	-1.484744
12	1	0	-1.223874	1.293853	1.281752
13	1	0	-1.327419	2.153062	-0.268807

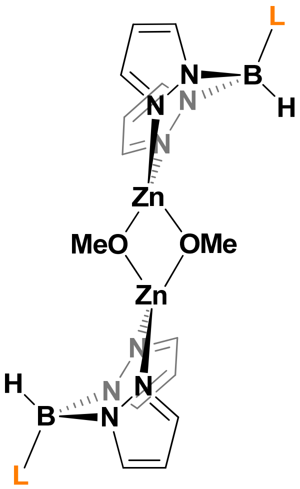

1

Standard orientation:

Center Number	Atomic Number	Atomic Type	Coordinates (Angstroms) X	Y	Z
1	6	0	-8.106275	-0.513037	0.699931
2	6	0	-8.177410	-0.416148	-0.731992
3	6	0	-8.487520	0.953686	-1.038573
4	6	0	-8.608985	1.677603	0.184229
5	6	0	-8.378305	0.768158	1.260640
6	26	0	-6.704047	0.854177	0.025945
7	6	0	-5.082480	1.414357	1.199610
8	6	0	-4.768410	0.168174	0.557627
9	6	0	-4.863273	0.424837	-0.853241
10	6	0	-5.217636	1.792586	-1.070879
11	6	0	-5.352440	2.409650	0.210073
12	5	0	-4.435514	-1.242652	1.263082
13	7	0	-3.581368	-1.021757	2.567421
14	7	0	-2.347024	-0.447460	2.555770
15	6	0	-1.947762	-0.335524	3.833828
16	6	0	-2.933399	-0.826884	4.693800
17	6	0	-3.948594	-1.252909	3.841950
18	30	0	-1.319383	-0.322474	0.751435
19	8	0	0.653754	-0.694750	0.820235
20	6	0	1.335878	-1.715803	1.520349
21	15	0	-7.921542	-1.851451	-1.841289
22	6	0	-7.506812	-1.009194	-3.454119
23	30	0	1.267842	1.029411	0.061856
24	8	0	-0.705452	1.372374	-0.071522
25	6	0	-1.386364	2.551916	-0.448086
26	7	0	2.142062	1.398832	-1.787589
27	7	0	3.384576	1.951384	-1.852667

28	6	0	3.620943	2.358857	-3.114032
29	6	0	2.509259	2.072025	-3.901151
30	6	0	1.605430	1.476366	-3.016735
31	5	0	4.347338	1.995255	-0.614350
32	7	0	3.630472	2.788551	0.546946
33	7	0	2.450456	2.390468	1.097161
34	6	0	2.180923	3.230669	2.111157
35	6	0	3.197927	4.180036	2.236894
36	6	0	4.093068	3.858161	1.220469
37	6	0	4.724450	0.509935	-0.121874
38	6	0	5.484448	0.183908	1.052678
39	6	0	5.613699	-1.233745	1.162435
40	6	0	4.951534	-1.817024	0.040546
41	6	0	4.408123	-0.749407	-0.740817
42	26	0	6.473860	-0.573957	-0.620766
43	6	0	8.327336	0.350978	-0.832485
44	6	0	7.513072	0.598450	-1.977130
45	6	0	7.189070	-0.659769	-2.565288
46	6	0	7.805878	-1.680418	-1.781326
47	6	0	8.525593	-1.065652	-0.701600
48	15	0	9.720843	-1.892562	0.415605
49	6	0	9.434915	-0.975019	2.014892
50	6	0	8.833711	-3.489450	0.793031
51	7	0	-2.389391	-1.834567	-0.178840
52	7	0	-3.630751	-2.161582	0.276381
53	6	0	-4.044278	-3.287662	-0.334797
54	6	0	-3.049609	-3.721577	-1.207214
55	6	0	-2.029354	-2.777503	-1.065889
56	6	0	-9.703327	-2.300827	-2.206233
57	1	0	-2.913841	-0.872067	5.772444
58	1	0	-5.134706	1.569115	2.270589
59	1	0	-5.643052	3.435576	0.397864
60	1	0	-8.363953	1.018752	2.313407
61	1	0	-7.840281	-1.405805	1.250930
62	1	0	-8.581376	1.376906	-2.029857
63	1	0	-8.799711	2.739179	0.277583
64	1	0	-5.385708	2.267359	-2.029317
65	1	0	-4.713383	-0.316173	-1.628628
66	1	0	-3.067211	-4.592247	-1.845500
67	1	0	-5.399022	-1.880367	1.610355
68	1	0	-9.730451	-3.095781	-2.960535
69	1	0	-10.173767	-2.685535	-1.295455
70	1	0	-10.287175	-1.446432	-2.568436
71	1	0	-7.444169	-1.769219	-4.240662
72	1	0	-8.252340	-0.263273	-3.753683
73	1	0	-6.531287	-0.520695	-3.374058

74	1	0	-1.059770	-2.727474	-1.543672
75	1	0	-5.023015	-3.691528	-0.121039
76	1	0	-0.976793	0.083186	4.063650
77	1	0	-4.907669	-1.702313	4.054554
78	1	0	-1.397321	3.291456	0.367709
79	1	0	-0.898726	3.018961	-1.316922
80	1	0	-2.426450	2.335271	-0.719133
81	1	0	2.384201	-1.772729	1.204378
82	1	0	1.317524	-1.544883	2.608430
83	1	0	0.871336	-2.694297	1.327642
84	1	0	1.272709	3.111891	2.687401
85	1	0	3.273787	4.983425	2.954304
86	1	0	5.028963	4.314189	0.931984
87	1	0	0.602769	1.109194	-3.190913
88	1	0	2.376913	2.272162	-4.953937
89	1	0	4.562658	2.825037	-3.364909
90	1	0	5.292021	2.677740	-0.931800
91	1	0	5.906622	0.905245	1.741983
92	1	0	6.133756	-1.769653	1.945602
93	1	0	4.894998	-2.872712	-0.192547
94	1	0	3.875972	-0.870139	-1.675903
95	1	0	7.727919	-2.744636	-1.962216
96	1	0	7.153488	1.566336	-2.299321
97	1	0	6.551721	-0.817745	-3.425639
98	1	0	8.716516	1.107835	-0.163858
99	1	0	9.385094	-4.020724	1.576726
100	1	0	7.801240	-3.328871	1.119236
101	1	0	8.828913	-4.128363	-0.096075
102	1	0	10.008554	-1.466237	2.808763
103	1	0	9.810321	0.049411	1.923762
104	1	0	8.377870	-0.939862	2.297110

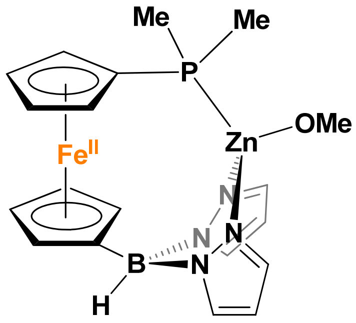

2

Standard orientation:

Center Number	Atomic Number	Atomic Type	Coordinates (Angstroms) X	Y	Z
1	6	0	2.827229	-1.274958	-1.833238
2	6	0	1.418888	-1.250191	-1.597444
3	6	0	1.121767	-1.876085	-0.340243
4	6	0	2.394767	-2.261055	0.201480
5	6	0	3.436857	-1.910144	-0.710477
6	26	0	2.446190	-0.206244	-0.099199
7	6	0	3.027092	1.767541	-0.395520
8	6	0	1.704067	1.706773	0.163534
9	6	0	1.805808	1.031935	1.431733
10	6	0	3.170019	0.690196	1.644059
11	6	0	3.921934	1.137148	0.516256
12	15	0	0.132646	2.258759	-0.533615
13	6	0	-0.138152	3.973569	0.107956
14	5	0	-0.322757	-2.341558	0.220645
15	7	0	-0.782039	-1.692158	1.576467
16	7	0	-1.290874	-0.436578	1.702284
17	6	0	-1.539208	-0.237798	3.008496
18	6	0	-1.176998	-1.366285	3.749525
19	6	0	-0.709138	-2.263674	2.795081
20	30	0	-1.826235	0.742897	0.034561
21	8	0	-3.193589	2.047055	0.066220
22	6	0	-4.540956	1.818891	0.354689
23	7	0	-1.962660	-0.940686	-1.219998
24	7	0	-1.448660	-2.148095	-0.853022
25	6	0	-1.977254	-3.109820	-1.635558
26	6	0	-2.864888	-2.529408	-2.535140
27	6	0	-2.824313	-1.166880	-2.226428
28	6	0	0.467555	2.545286	-2.326893
29	1	0	-1.972536	0.697342	3.338852
30	1	0	-3.456745	-3.023903	-3.290854
31	1	0	-3.372858	-0.341778	-2.661568
32	1	0	-1.682416	-4.139949	-1.499073
33	1	0	2.542436	-2.743657	1.160278
34	1	0	4.498564	-2.058649	-0.558428
35	1	0	3.560204	0.141478	2.491064
36	1	0	0.977708	0.787336	2.081219
37	1	0	3.294346	2.182641	-1.358130
38	1	0	4.982190	0.986814	0.358858
39	1	0	3.342028	-0.859754	-2.690598
40	1	0	0.677297	-0.836300	-2.269399
41	1	0	-1.257736	-1.517905	4.815592
42	1	0	-5.018825	1.078321	-0.317278

43	1	0	-4.714430	1.463047	1.389782
44	1	0	-0.288859	-3.531367	0.442109
45	1	0	-5.121431	2.751394	0.247560
46	1	0	-0.338183	-3.272999	2.897683
47	1	0	0.642004	4.664469	-0.229151
48	1	0	-1.120907	4.318480	-0.227377
49	1	0	-0.144608	3.950007	1.201703
50	1	0	1.286506	3.256783	-2.476792
51	1	0	0.721439	1.598478	-2.810176
52	1	0	-0.437112	2.947420	-2.793605

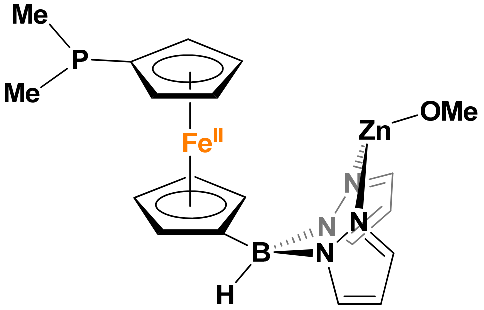

3

Standard orientation:

Center Number	Atomic Number	Atomic Type	Coordinates (Angstroms) X	Y	Z
1	7	0	-1.208043	1.246388	-1.476769
2	7	0	-1.138347	2.119977	-0.437135
3	6	0	-0.848073	3.345818	-0.909783
4	6	0	-0.722276	3.280255	-2.294877
5	6	0	-0.964154	1.938711	-2.602379
6	5	0	-1.395077	1.664359	1.044100
7	7	0	-2.839783	1.057191	1.119183
8	7	0	-3.163167	-0.075802	0.436433
9	6	0	-4.434387	-0.381858	0.747589
10	6	0	-4.944880	0.554064	1.650841
11	6	0	-3.895831	1.446789	1.855454
12	30	0	-1.783937	-0.666259	-0.996676
13	8	0	-1.915490	-2.123587	-2.151113
14	6	0	-2.498201	-3.363843	-1.857214
15	6	0	-0.318138	0.597739	1.607725
16	6	0	1.002549	0.933673	2.052784
17	6	0	1.564515	-0.166131	2.769376
18	6	0	0.612292	-1.226971	2.749693
19	6	0	-0.529997	-0.762671	2.034451

20	26	0	1.206011	-0.740328	0.822281
21	6	0	1.097956	-1.829247	-0.996792
22	6	0	1.915135	-2.504734	-0.041820
23	6	0	2.995043	-1.642423	0.306968
24	6	0	2.855395	-0.410849	-0.420691
25	6	0	1.669807	-0.537936	-1.215456
26	15	0	3.923562	1.081727	-0.454043
27	6	0	5.193702	0.522558	-1.712366
28	6	0	4.944805	0.854032	1.090938
29	1	0	1.491786	1.883557	1.875776
30	1	0	-3.499470	-3.285656	-1.392782
31	1	0	-0.977457	1.437744	-3.561324
32	1	0	-5.930036	0.582005	2.091808
33	1	0	-4.907390	-1.250117	0.308490
34	1	0	-3.827991	2.327810	2.476916
35	1	0	2.550357	-0.205991	3.214250
36	1	0	1.276167	0.231365	-1.865558
37	1	0	1.732819	-3.491312	0.364655
38	1	0	3.765325	-1.864450	1.033750
39	1	0	0.742854	-2.214146	3.174348
40	1	0	-1.436822	-1.335114	1.887322
41	1	0	-0.490053	4.085969	-2.974960
42	1	0	-1.875713	-3.978028	-1.179266
43	1	0	-1.405000	2.672155	1.709406
44	1	0	-2.626655	-3.943634	-2.784249
45	1	0	-0.751846	4.179852	-0.230050
46	1	0	5.741894	1.605626	1.089759
47	1	0	4.326458	1.023573	1.977232
48	1	0	5.407119	-0.137444	1.162065
49	1	0	5.989671	1.273215	-1.777967
50	1	0	5.638341	-0.447287	-1.460627
51	1	0	4.721014	0.446874	-2.696934
52	1	0	0.226146	-2.235383	-1.500688

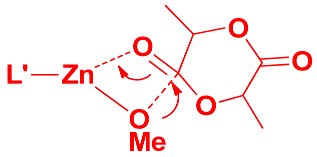

TSI-II^{monomer}-LA

Standard orientation:

Center Number	Atomic Number	Atomic Type	Coordinates (Angstroms) X	Y	Z

1	6	0	-3.124565	-2.910447	-0.442393
2	6	0	-4.237317	-2.829829	0.443867
3	6	0	-3.838038	-2.085691	1.594094
4	6	0	-2.484895	-1.684174	1.418287
5	6	0	-2.027023	-2.194312	0.151654
6	26	0	-3.630640	-0.923294	-0.134036
7	6	0	-3.213002	0.519402	-1.588307
8	6	0	-3.331082	1.182478	-0.320291
9	6	0	-4.648110	0.862212	0.153725
10	6	0	-5.325172	0.047524	-0.804265
11	6	0	-4.426957	-0.169766	-1.891455
12	5	0	-2.340618	2.280852	0.336810
13	7	0	-1.299786	2.808582	-0.700351
14	7	0	-0.213282	2.102316	-1.118147
15	6	0	0.405937	2.849099	-2.048709
16	6	0	-0.289415	4.043773	-2.257375
17	6	0	-1.361729	3.973672	-1.375317
18	30	0	0.618030	0.443078	-0.060979
19	15	0	-0.377017	-1.893158	-0.522031
20	6	0	0.678279	-3.235074	0.196590
21	8	0	2.205776	-0.309414	1.396445
22	6	0	3.265518	-0.270894	0.752455
23	6	0	4.201858	0.915083	0.919565
24	8	0	5.244787	0.991040	-0.073967
25	6	0	5.692480	-0.111928	-0.691030
26	6	0	5.056625	-1.464942	-0.375450
27	8	0	3.798810	-1.437510	0.335866
28	6	0	6.033251	-2.360404	0.388905
29	8	0	6.604670	-0.052412	-1.481766
30	6	0	4.826786	0.923527	2.317747
31	8	0	2.385583	0.467405	-1.024659
32	6	0	2.620860	-0.069693	-2.293553
33	7	0	-0.446612	1.083836	1.669735
34	7	0	-1.579565	1.837807	1.638823
35	6	0	-1.966032	2.108816	2.901070
36	6	0	-1.073218	1.516962	3.788474
37	6	0	-0.132939	0.892622	2.963507
38	6	0	-0.503466	-2.391022	-2.298777
39	1	0	0.753850	0.329257	3.214303
40	1	0	-0.043700	4.847472	-2.935509
41	1	0	1.325546	2.495306	-2.491609
42	1	0	-2.163848	4.670037	-1.178718
43	1	0	-5.063448	1.187265	1.100237
44	1	0	-6.321234	-0.365588	-0.705749
45	1	0	-4.470641	-1.821698	2.431337
46	1	0	-1.909497	-1.073707	2.099963
47	1	0	-3.123098	-3.392462	-1.410623
48	1	0	-5.223427	-3.235831	0.259254
49	1	0	-4.621923	-0.771590	-2.770270
50	1	0	-2.333766	0.547512	-2.220084
51	1	0	-1.093449	1.553005	4.867700
52	1	0	2.693918	-1.175670	-2.282892
53	1	0	1.818122	0.184862	-3.007984

54	1	0	-2.995972	3.243325	0.669707
55	1	0	-2.842502	2.714664	3.079546
56	1	0	0.294122	-4.227656	-0.061332
57	1	0	1.705374	-3.121787	-0.161640
58	1	0	0.695482	-3.126033	1.284502
59	1	0	-0.854577	-3.422699	-2.406784
60	1	0	-1.192499	-1.722316	-2.821666
61	1	0	0.484219	-2.304813	-2.761255
62	1	0	3.606397	1.813871	0.767536
63	1	0	3.560293	0.312827	-2.726309
64	1	0	4.838302	-1.916184	-1.346797
65	1	0	5.487582	1.789613	2.415911
66	1	0	4.039243	0.987049	3.073555
67	1	0	5.413023	0.016705	2.501517
68	1	0	6.954118	-2.469960	-0.189568
69	1	0	6.279476	-1.935028	1.367242
70	1	0	5.584515	-3.345967	0.543335

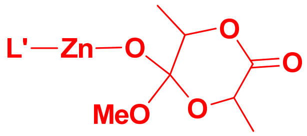

II^{monomer}-LA

Standard orientation:

Center Number	Atomic Number	Atomic Type	Coordinates (Angstroms) X	Y	Z
1	6	0	-1.975214	-2.156024	0.514375
2	6	0	-2.641967	-1.457642	1.583197
3	6	0	-3.993928	-1.896628	1.623385
4	6	0	-4.181941	-2.857186	0.585096
5	6	0	-2.943623	-3.024957	-0.098130
6	26	0	-3.551997	-1.041553	-0.232050
7	6	0	-4.608402	0.739323	-0.393906
8	6	0	-3.258968	1.002727	-0.808038
9	6	0	-3.016456	0.105850	-1.902327
10	6	0	-4.188384	-0.665140	-2.168683
11	6	0	-5.184818	-0.265880	-1.228867
12	5	0	-2.357994	2.257680	-0.332946
13	7	0	-1.895770	2.242632	1.168912
14	7	0	-0.865802	1.499864	1.657732
15	6	0	-0.788970	1.744237	2.977309
16	6	0	-1.787955	2.642414	3.362965
17	6	0	-2.458400	2.934711	2.179628
18	30	0	0.492373	0.515243	0.396250
19	7	0	0.023971	1.720633	-1.239252
20	7	0	-1.113977	2.472073	-1.265344
21	6	0	-1.036373	3.348545	-2.286405
22	6	0	0.177080	3.178352	-2.944203

23	6	0	0.810445	2.149976	-2.241261
24	15	0	-0.246504	-1.881468	0.075057
25	6	0	0.730995	-3.075877	1.093846
26	8	0	2.250531	0.530330	1.228445
27	6	0	3.330602	0.311648	0.488851
28	8	0	3.187835	-0.901510	-0.274291
29	6	0	4.281555	-1.175720	-1.155871
30	6	0	5.660174	-0.752817	-0.629066
31	8	0	5.760643	-0.116814	0.554022
32	6	0	4.588470	0.152249	1.368200
33	8	0	6.655635	-0.974670	-1.281135
34	6	0	4.280750	-2.669350	-1.476208
35	8	0	3.544778	1.308768	-0.535245
36	6	0	3.778322	2.632651	-0.073694
37	6	0	4.453727	-0.925547	2.435617
38	6	0	-0.056035	-2.548078	-1.634625
39	1	0	-0.012214	1.275071	3.566780
40	1	0	0.548612	3.728936	-3.795566
41	1	0	1.793429	1.716978	-2.366896
42	1	0	-1.851502	4.032133	-2.473544
43	1	0	-5.113660	1.228507	0.430408
44	1	0	-6.180341	-0.681709	-1.137395
45	1	0	-4.758982	-1.526816	2.293196
46	1	0	-2.199315	-0.700508	2.214912
47	1	0	-2.774829	-3.669520	-0.950285
48	1	0	-5.114458	-3.344531	0.331321
49	1	0	-4.293559	-1.433436	-2.924364
50	1	0	-2.084370	0.037629	-2.449097
51	1	0	-1.985258	3.036803	4.348581
52	1	0	-3.016829	3.267367	-0.443780
53	1	0	-3.290279	3.595657	1.984856
54	1	0	0.443133	-4.111698	0.885931
55	1	0	1.791988	-2.926747	0.874381
56	1	0	0.563397	-2.866744	2.154601
57	1	0	-0.347039	-3.602217	-1.689362
58	1	0	-0.668182	-1.969253	-2.330534
59	1	0	0.993864	-2.454496	-1.925998
60	1	0	4.150003	-0.604995	-2.083714
61	1	0	4.810227	1.109886	1.846459
62	1	0	5.102291	-2.897102	-2.159112
63	1	0	4.405011	-3.264193	-0.564813
64	1	0	3.335025	-2.953249	-1.949344
65	1	0	5.393051	-1.019511	2.989633
66	1	0	3.652556	-0.660016	3.129565
67	1	0	4.215494	-1.892917	1.982837
68	1	0	3.555537	3.302205	-0.908764
69	1	0	3.120327	2.881915	0.766779
70	1	0	4.826700	2.776986	0.221164

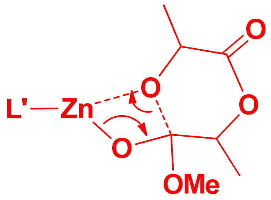

TSII-III^{monomer}-LA

Standard orientation:

Center Number	Atomic Number	Atomic Type	Coordinates (Angstroms) X	Y	Z
1	6	0	-0.682735	3.477945	-2.073251
2	7	0	-0.749861	2.520221	-1.125766
3	7	0	0.250783	1.614725	-1.325493
4	6	0	0.941509	2.030643	-2.403634
5	6	0	0.386890	3.205684	-2.920763
6	5	0	-1.908123	2.370032	-0.084429
7	7	0	-1.314519	2.073520	1.336971
8	7	0	-0.302291	1.203788	1.614574
9	6	0	-0.150857	1.188466	2.952421
10	6	0	-1.076076	2.046006	3.556640
11	6	0	-1.785574	2.590991	2.489766
12	30	0	0.874385	0.179543	0.145912
13	8	0	2.376532	-0.068846	2.002512
14	6	0	3.488614	0.053225	1.460740
15	8	0	4.122509	1.236193	1.348786
16	6	0	3.356589	2.428716	1.576262
17	6	0	-3.042784	1.337664	-0.597347
18	6	0	-4.382193	1.245738	-0.084520
19	6	0	-5.171706	0.413455	-0.937726
20	6	0	-4.328005	-0.037377	-1.998164
21	6	0	-3.032686	0.526582	-1.783518
22	26	0	-3.591774	-0.673490	-0.161993
23	6	0	-2.127854	-2.042753	0.345760
24	6	0	-3.260083	-2.720206	-0.234958
25	6	0	-4.397141	-2.460473	0.584268
26	6	0	-3.988787	-1.619755	1.663690
27	6	0	-2.595705	-1.360656	1.525208
28	15	0	-0.409494	-2.044281	-0.216683
29	6	0	0.291673	-3.569512	0.571456
30	8	0	2.756434	-0.226691	-0.463859
31	6	0	3.672414	-0.170912	-1.504253
32	6	0	5.117455	-0.489040	-1.055225
33	8	0	6.037210	-0.388356	-1.839561
34	6	0	3.308705	-1.121953	-2.662221
35	6	0	-0.493754	-2.542684	-1.994624
36	6	0	4.490466	-1.087510	1.302805
37	6	0	3.866257	-2.472415	1.256794
38	8	0	5.412589	-0.884836	0.210687
39	1	0	0.628011	0.582865	3.391786
40	1	0	0.718404	3.778600	-3.774575

41	1	0	1.804594	1.476436	-2.741909
42	1	0	-1.403551	4.283056	-2.080931
43	1	0	-4.738704	1.722412	0.821288
44	1	0	-6.209066	0.141423	-0.786513
45	1	0	-4.633946	-1.211011	2.430570
46	1	0	-1.997868	-0.737467	2.175364
47	1	0	-3.257896	-3.307680	-1.143618
48	1	0	-5.407301	-2.796236	0.386881
49	1	0	-4.612120	-0.708563	-2.799315
50	1	0	-2.172271	0.374974	-2.423774
51	1	0	-1.199332	2.256379	4.609303
52	1	0	-2.405478	3.470694	0.006310
53	1	0	-2.583682	3.319617	2.470551
54	1	0	-0.352752	-4.433176	0.373732
55	1	0	1.293719	-3.769892	0.181273
56	1	0	0.365876	-3.423298	1.653287
57	1	0	-1.002040	-3.505001	-2.118859
58	1	0	-1.026199	-1.780783	-2.569679
59	1	0	0.523326	-2.630081	-2.389060
60	1	0	4.085772	3.239476	1.594990
61	1	0	2.648816	2.587065	0.755967
62	1	0	2.817738	2.369835	2.524583
63	1	0	3.760773	0.850281	-1.917414
64	1	0	4.048308	-1.063220	-3.466246
65	1	0	3.259954	-2.157389	-2.303533
66	1	0	2.325100	-0.851949	-3.063664
67	1	0	5.151465	-1.025324	2.177875
68	1	0	3.258632	-2.640108	2.151432
69	1	0	3.230850	-2.572343	0.375307
70	1	0	4.659672	-3.225793	1.221270

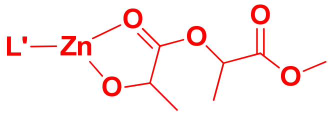

III^{monomer}-LA

Standard orientation:

Center Number	Atomic Number	Atomic Type	Coordinates (Angstroms) X	Y	Z
1	6	0	2.196687	0.999243	-1.896885
2	6	0	3.136149	1.533222	-0.945330
3	6	0	4.448960	1.288233	-1.435147
4	6	0	4.340462	0.600040	-2.681014
5	6	0	2.957688	0.423036	-2.973130
6	26	0	3.412932	-0.507791	-1.173034
7	6	0	4.303107	-1.544234	0.390483
8	6	0	2.886952	-1.542546	0.627082
9	6	0	2.310063	-2.169212	-0.527421
10	6	0	3.340264	-2.562490	-1.435461
11	6	0	4.587115	-2.175411	-0.859246

12	5	0	2.168041	-1.164057	2.026771
13	7	0	0.701486	-1.718648	2.099379
14	6	0	0.327996	-2.847805	2.733971
15	6	0	-1.021942	-3.079194	2.498231
16	6	0	-1.418295	-2.012016	1.686945
17	7	0	-0.375212	-1.200116	1.440112
18	30	0	-0.430733	0.799391	0.765299
19	8	0	-2.567599	0.362285	-0.404730
20	6	0	-3.355088	1.162482	0.091727
21	8	0	-4.668534	1.139851	-0.193125
22	6	0	-5.127242	0.125420	-1.099158
23	6	0	-6.590470	0.435882	-1.406442
24	15	0	0.405686	1.024122	-1.664435
25	6	0	-0.149346	2.589640	-2.493154
26	6	0	-0.241129	-0.265463	-2.818824
27	7	0	2.170157	0.353266	2.414137
28	7	0	1.294559	1.268243	1.923300
29	6	0	1.586432	2.446512	2.496545
30	6	0	2.680629	2.308996	3.358418
31	6	0	3.009037	0.959888	3.279201
32	8	0	-1.614185	2.196265	1.386435
33	6	0	-2.943223	2.267227	1.073089
34	6	0	-3.329988	3.642765	0.479548
35	6	0	-4.972833	-1.257759	-0.461702
36	8	0	-4.827398	-1.475605	0.719136
37	8	0	-5.066591	-2.206752	-1.410295
38	6	0	-4.972986	-3.565084	-0.940242
39	1	0	2.553663	-0.088426	-3.836538
40	1	0	-6.989076	-0.301253	-2.109030
41	1	0	0.975403	3.311215	2.275408
42	1	0	-1.628602	-3.889875	2.873717
43	1	0	-2.396179	-1.776956	1.289547
44	1	0	1.051954	-3.407700	3.307633
45	1	0	5.045191	-1.124721	1.059392
46	1	0	5.564920	-2.299090	-1.307900
47	1	0	5.369986	1.534014	-0.923077
48	1	0	2.882444	1.997322	-0.002814
49	1	0	5.164878	0.237729	-3.281569
50	1	0	3.201612	-3.038853	-2.398025
51	1	0	1.248841	-2.326569	-0.675333
52	1	0	3.149349	3.067533	3.967781
53	1	0	2.763805	-1.720448	2.923076
54	1	0	3.770657	0.386533	3.787492
55	1	0	0.193540	2.634908	-3.532351
56	1	0	-1.242534	2.647883	-2.469761
57	1	0	0.250518	3.450740	-1.949582
58	1	0	0.013739	-0.046908	-3.861426
59	1	0	0.173975	-1.238604	-2.545235
60	1	0	-1.328417	-0.307668	-2.711905
61	1	0	-3.593374	2.103733	1.958020
62	1	0	-4.403232	3.723569	0.276215
63	1	0	-3.043475	4.413945	1.200085
64	1	0	-2.773052	3.825781	-0.447421
65	1	0	-4.522179	0.149951	-2.009941

66	1	0	-7.185670	0.413093	-0.488489
67	1	0	-6.673440	1.431575	-1.851414
68	1	0	-5.106416	-4.188663	-1.824134
69	1	0	-3.993286	-3.741592	-0.489055
70	1	0	-5.751729	-3.768670	-0.201103

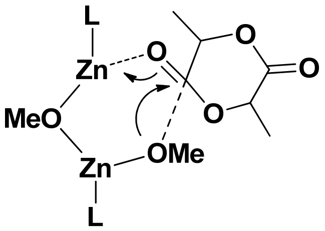

TSI-II^{dimer}-LA

Standard orientation:

Center Number	Atomic Number	Atomic Type	Coordinates (Angstroms) X	Y	Z
1	6	0	1.323833	4.177595	-1.181677
2	6	0	0.537592	3.374464	-0.165993
3	8	0	0.999589	3.314073	1.083857
4	6	0	2.429641	3.337239	1.333824
5	6	0	3.308044	3.810396	0.179660
6	8	0	2.749703	4.137162	-1.000033
7	8	0	-0.680353	3.220890	-0.356524
8	30	0	-1.846708	1.546551	-0.138297
9	7	0	-3.685295	2.318624	0.427225
10	6	0	-4.016996	3.303907	1.279041
11	6	0	-5.366325	3.634133	1.134981
12	6	0	-5.821952	2.787998	0.127455
13	7	0	-4.803295	2.012299	-0.289230
14	5	0	-4.874243	0.821313	-1.312347
15	6	0	-4.745322	-0.588667	-0.541103
16	6	0	-4.298734	-1.840581	-1.084831
17	6	0	-4.365294	-2.852877	-0.077300
18	6	0	-4.867575	-2.243990	1.112519
19	6	0	-5.101583	-0.864104	0.822627
20	26	0	-6.254776	-2.074508	-0.415400
21	6	0	-7.501222	-3.659108	-0.935031
22	6	0	-8.030555	-2.966915	0.194164
23	6	0	-8.271940	-1.599349	-0.177900
24	6	0	-7.875638	-1.469172	-1.553256
25	6	0	-7.410940	-2.733168	-2.018309
26	15	0	-8.955092	-0.206813	0.796619
27	6	0	-10.785183	-0.584030	0.663538
28	6	0	2.661928	4.177826	2.585200
29	8	0	4.505564	3.870636	0.323010
30	6	0	0.858974	5.638337	-1.164753
31	8	0	-0.902029	0.313007	1.054265
32	6	0	-1.607130	-0.503285	1.983554

33	7	0	-2.405273	1.021553	-2.062529
34	7	0	-3.728476	0.972939	-2.380887
35	6	0	-3.856322	0.921003	-3.720595
36	6	0	-2.591398	0.940519	-4.301263
37	6	0	-1.715325	1.011780	-3.215096
38	30	0	1.012361	-0.046757	0.581499
39	8	0	1.272533	1.378683	-0.744198
40	6	0	1.998537	1.105360	-1.924657
41	7	0	2.033046	-0.239162	2.406121
42	7	0	2.923425	-1.255358	2.572781
43	6	0	3.215213	-1.378833	3.881203
44	6	0	2.499361	-0.425427	4.600216
45	6	0	1.765817	0.258153	3.626835
46	5	0	3.529150	-2.063763	1.365939
47	7	0	2.347414	-2.738035	0.572142
48	7	0	1.332434	-2.029251	0.005050
49	6	0	0.552653	-2.905920	-0.650348
50	6	0	1.068025	-4.199054	-0.526397
51	6	0	2.204878	-4.040423	0.260618
52	6	0	4.377657	-1.092629	0.403030
53	6	0	4.761961	-1.355506	-0.955587
54	6	0	5.500197	-0.243857	-1.466631
55	6	0	5.603640	0.726268	-0.425169
56	6	0	4.920160	0.201668	0.714668
57	26	0	6.487606	-1.054044	0.176250
58	6	0	8.450823	-1.629914	-0.343555
59	6	0	7.686481	-2.755988	0.115181
60	6	0	7.249146	-2.503345	1.449347
61	6	0	7.729084	-1.216075	1.831450
62	6	0	8.461432	-0.677675	0.731119
63	15	0	9.480012	-1.560048	-1.858656
64	6	0	9.302834	0.229948	-2.353194
65	6	0	-8.671532	-0.795449	2.544589
66	6	0	8.356709	-2.363153	-3.113439
67	1	0	-0.335566	-2.563130	-1.165059
68	1	0	3.912065	-2.135828	4.210402
69	1	0	1.141839	3.740341	-2.163493
70	1	0	-5.065379	-2.744426	2.052020
71	1	0	2.506959	-0.260679	5.667381
72	1	0	-7.599126	-0.805303	2.760501
73	1	0	-4.838604	0.869468	-4.167109
74	1	0	-0.635332	1.061125	-3.195925
75	1	0	-2.347348	0.912333	-5.352741
76	1	0	-3.987771	-1.999269	-2.110386
77	1	0	-4.117492	-3.899230	-0.203988
78	1	0	-7.014874	-2.942776	-3.003406
79	1	0	-7.883045	-0.546351	-2.118621
80	1	0	-8.187706	-3.394913	1.175349
81	1	0	-7.185010	-4.694347	-0.954715
82	1	0	-5.505698	-0.136955	1.515971
83	1	0	-5.931695	4.377796	1.676375
84	1	0	-5.903164	0.972206	-1.924323
85	1	0	-11.346819	0.111837	1.297525
86	1	0	-11.112120	-0.435351	-0.370792

87	1	0	-11.024652	-1.611122	0.962846
88	1	0	-9.151439	-0.091308	3.233165
89	1	0	-9.079500	-1.795219	2.734719
90	1	0	-3.268342	3.724191	1.937754
91	1	0	-6.804278	2.670952	-0.306310
92	1	0	-2.447500	0.048226	2.425062
93	1	0	-2.011644	-1.404041	1.504720
94	1	0	-0.938710	-0.810540	2.799168
95	1	0	3.066389	1.340233	-1.822980
96	1	0	1.922377	0.045431	-2.212258
97	1	0	1.605554	1.694231	-2.772294
98	1	0	0.674039	-5.115152	-0.940517
99	1	0	2.923283	-4.763129	0.619411
100	1	0	1.055331	1.067135	3.735546
101	1	0	4.149182	-2.977391	1.855439
102	1	0	4.535663	-2.262239	-1.503689
103	1	0	5.915630	-0.157401	-2.461767
104	1	0	6.109849	1.680612	-0.477368
105	1	0	4.855540	0.690692	1.678863
106	1	0	8.930513	0.297399	0.705955
107	1	0	6.613270	-3.149240	2.039945
108	1	0	7.535643	-0.714350	2.770738
109	1	0	7.462532	-3.643642	-0.462079
110	1	0	9.769380	0.370148	-3.334706
111	1	0	8.259094	0.556643	-2.395896
112	1	0	9.841212	0.863308	-1.640613
113	1	0	8.808471	-2.258243	-4.106179
114	1	0	8.274814	-3.434207	-2.901342
115	1	0	7.352707	-1.927388	-3.122159
116	1	0	1.443894	6.208625	-1.891673
117	1	0	-0.200377	5.698021	-1.427365
118	1	0	1.002988	6.084010	-0.174562
119	1	0	2.719450	2.298888	1.517723
120	1	0	2.088522	3.766012	3.420458
121	1	0	3.724592	4.161943	2.836030
122	1	0	2.351070	5.215588	2.425524

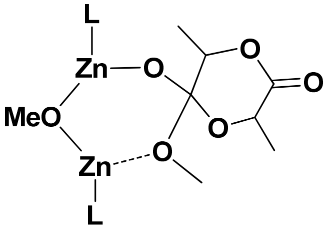

II^{dimer}-LA

Standard orientation:

Center Number	Atomic Number	Atomic Type	Coordinates (Angstroms) X	Y	Z
1	26	0	6.649038	-0.641174	0.553489

2	6	0	6.040091	0.473905	-1.099749
3	6	0	5.581397	1.085155	0.104922
4	6	0	4.738698	0.141900	0.768500
5	6	0	4.652946	-1.063924	-0.008646
6	6	0	5.483442	-0.841259	-1.160873
7	6	0	8.159909	-2.075531	0.572036
8	6	0	7.296624	-2.246791	1.694636
9	6	0	7.337995	-1.048297	2.466651
10	6	0	8.227468	-0.140677	1.817649
11	6	0	8.754812	-0.770050	0.639651
12	15	0	10.147841	-0.160447	-0.383840
13	1	0	3.663160	-3.550329	2.914096
14	7	0	2.966697	-2.805598	-0.969277
15	7	0	1.998471	-2.003769	-1.493818
16	6	0	1.547947	-2.603502	-2.610753
17	6	0	2.237928	-3.797365	-2.830878
18	6	0	3.125021	-3.882340	-1.760726
19	7	0	2.838001	-2.213936	1.513057
20	6	0	1.868494	-2.333355	3.520639
21	7	0	1.794909	-1.336192	1.505241
22	30	0	1.134241	-0.532318	-0.294006
23	8	0	-0.820524	-0.578158	-0.543101
24	6	0	-5.076597	-1.012602	0.668280
25	6	0	-5.120275	-0.433833	-0.645355
26	6	0	-5.196166	-1.543307	-1.554619
27	6	0	-5.185300	-2.769635	-0.817961
28	1	0	-5.116367	-3.138677	1.394258
29	6	0	-5.112390	-2.438023	0.568875
30	1	0	-8.548184	-1.631701	-2.519811
31	6	0	-8.520286	-2.805220	-0.609589
32	6	0	-8.553089	-1.642431	-1.437554
33	6	0	-8.527677	-0.499096	-0.587746
34	6	0	-8.491074	-0.939845	0.779700
35	6	0	-8.483760	-2.377376	0.750488
36	1	0	-8.424995	-3.029534	1.611641
37	15	0	-8.478217	0.216961	2.199916
38	5	0	-5.144627	1.137555	-1.006507
39	7	0	-4.482723	1.971896	0.148802
40	7	0	-3.154233	1.921548	0.447581
41	6	0	-2.967586	2.657333	1.556272
42	6	0	-5.116415	2.716076	1.075372
43	6	0	-4.184959	3.178171	2.001338
44	6	0	-4.945019	1.780820	-3.532230
45	7	0	-4.392009	1.422081	-2.357466
46	6	0	-3.953446	1.829639	-4.507482
47	1	0	-6.005866	1.975445	-3.592126
48	30	0	-1.779730	1.133538	-0.891800
49	8	0	-0.226285	2.307007	-1.315043
50	6	0	0.819146	2.416627	-0.551517
51	6	0	1.493794	3.794705	-0.646215
52	8	0	2.687053	3.863584	0.170920
53	6	0	2.749997	3.197141	1.338200
54	6	0	1.599439	2.279267	1.764318
55	1	0	0.230977	2.047130	3.419397

56	6	0	0.541848	4.930425	-0.287279
57	8	0	1.862544	1.335153	-0.864000
58	6	0	2.531321	1.444828	-2.133562
59	8	0	0.529008	2.109911	0.809199
60	6	0	-2.784639	1.482578	-3.824851
61	7	0	-3.056152	1.228165	-2.534086
62	6	0	2.895573	-2.816940	2.714566
63	6	0	1.201477	-1.412574	2.710777
64	6	0	0.992668	2.757645	3.082852
65	8	0	3.722630	3.305639	2.049414
66	26	0	-6.828667	-1.639466	-0.270463
67	6	0	-10.293960	0.188009	2.659560
68	6	0	-7.822787	-0.873954	3.564918
69	6	0	-1.504147	-1.811251	-0.320865
70	5	0	3.819354	-2.403507	0.296323
71	6	0	9.744152	1.655289	-0.525167
72	6	0	9.645277	-0.743396	-2.083891
73	1	0	0.751333	-2.149826	-3.185732
74	1	0	1.856162	3.938453	-1.665762
75	1	0	1.639613	-2.613922	4.537785
76	1	0	-6.775371	-1.120991	3.368110
77	1	0	-1.766547	1.416487	-4.184912
78	1	0	-4.064814	2.087233	-5.550170
79	1	0	-5.278089	-1.462726	-2.631935
80	1	0	-5.256941	-3.766692	-1.233973
81	1	0	-8.489024	0.532617	-0.911448
82	1	0	-8.485050	-3.830937	-0.954110
83	1	0	-5.044393	-0.447412	1.591464
84	1	0	-4.367773	3.802506	2.863164
85	1	0	-6.250408	1.597405	-1.156212
86	1	0	-10.442983	0.751257	3.588113
87	1	0	-10.875132	0.678542	1.871869
88	1	0	-10.676542	-0.830340	2.795232
89	1	0	-7.871243	-0.320868	4.509378
90	1	0	-8.389982	-1.804985	3.681150
91	1	0	-1.969782	2.776771	1.955126
92	1	0	-6.187534	2.847134	1.024262
93	1	0	-2.449280	-1.831872	-0.872534
94	1	0	-0.892379	-2.657806	-0.662588
95	1	0	-1.730399	-1.957719	0.744197
96	1	0	3.298831	2.221096	-2.079832
97	1	0	3.016244	0.486642	-2.322217
98	1	0	1.804974	1.665887	-2.922768
99	1	0	2.111450	-4.499566	-3.641295
100	1	0	3.860502	-4.632983	-1.510262
101	1	0	0.328489	-0.809405	2.923095
102	1	0	4.487506	-3.375733	0.553945
103	1	0	5.670599	-1.565501	-1.944933
104	1	0	6.708305	0.917489	-1.826245
105	1	0	5.829587	2.073385	0.468472
106	1	0	4.272683	0.304227	1.731498
107	1	0	8.449400	0.864942	2.150303
108	1	0	6.680458	-3.114008	1.891069
109	1	0	6.761827	-0.841285	3.359099

110	1	0	8.326613	-2.805642	-0.209515
111	1	0	10.435998	2.113439	-1.240764
112	1	0	8.713881	1.835611	-0.847908
113	1	0	9.901044	2.144013	0.441872
114	1	0	10.339835	-0.321359	-2.818781
115	1	0	9.730565	-1.833577	-2.138997
116	1	0	8.621442	-0.455644	-2.342973
117	1	0	1.076560	5.883886	-0.331883
118	1	0	-0.291035	4.951657	-0.994037
119	1	0	0.134754	4.802996	0.720435
120	1	0	2.070359	1.303751	1.923657
121	1	0	1.775976	2.826069	3.841546
122	1	0	0.524506	3.741012	2.969223

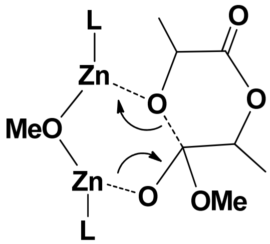

TSII-III^{dimer}-LA

Standard orientation:

Center Number	Atomic Number	Atomic Type	Coordinates (Angstroms) X	Y	Z
1	6	0	-1.248474	-1.784240	-3.281377
2	7	0	-1.785186	-1.457371	-2.092121
3	7	0	-2.676934	-2.435784	-1.772091
4	6	0	-2.700676	-3.352099	-2.758860
5	6	0	-1.801830	-2.977102	-3.753075
6	30	0	-1.151312	-0.090033	-0.638005
7	8	0	0.836714	-0.191452	-0.411055
8	30	0	1.912889	1.329646	0.220184
9	7	0	2.767658	1.351567	2.112825
10	7	0	4.122559	1.412350	2.238515
11	6	0	4.432872	1.700153	3.516716
12	6	0	3.259472	1.838091	4.252926
13	6	0	2.242424	1.618464	3.320984
14	5	0	5.129177	1.075078	1.073172
15	6	0	5.038656	-0.480605	0.662865
16	6	0	4.827963	-1.603429	1.532914
17	6	0	4.859756	-2.813415	0.772403
18	6	0	5.103530	-2.460700	-0.589480
19	6	0	5.215425	-1.036353	-0.650640
20	26	0	6.674757	-1.833171	0.604460
21	6	0	8.114333	-2.053561	2.092278
22	6	0	8.362095	-0.875673	1.329647

23	6	0	8.571228	-1.248226	-0.042581
24	6	0	8.436725	-2.678211	-0.107310
25	6	0	8.155719	-3.169078	1.201934
26	15	0	8.950518	-0.029929	-1.356893
27	6	0	8.509575	-0.985605	-2.897523
28	5	0	-3.623887	-2.380620	-0.511417
29	6	0	-4.657770	-1.152596	-0.633695
30	6	0	-5.396722	-0.768658	-1.805105
31	6	0	-6.208111	0.370027	-1.510274
32	6	0	-5.993719	0.707991	-0.140949
33	6	0	-5.053721	-0.228312	0.393761
34	26	0	-6.751607	-1.224244	-0.294574
35	6	0	-7.908915	-2.793979	-0.996732
36	6	0	-8.748279	-1.689448	-0.659138
37	6	0	-8.587661	-1.394322	0.736961
38	6	0	-7.623634	-2.329931	1.244150
39	6	0	-7.212381	-3.189902	0.182464
40	15	0	-9.637310	-0.271086	1.735486
41	6	0	-9.841051	1.177265	0.578319
42	7	0	-1.876655	-1.215838	0.981949
43	6	0	-1.357505	-1.368959	2.211730
44	6	0	-1.904611	-2.494839	2.833712
45	6	0	-2.777694	-3.018208	1.884601
46	7	0	-2.746213	-2.243925	0.782791
47	8	0	-1.650812	1.802754	-0.906094
48	6	0	-2.825660	2.185938	-1.584895
49	6	0	-3.417784	3.495097	-1.056018
50	8	0	-4.524102	3.866497	-1.363817
51	6	0	-2.646321	2.248975	-3.110776
52	6	0	-8.394627	0.432687	2.937349
53	6	0	1.402087	-1.504335	-0.372292
54	8	0	0.584850	2.842882	-0.058245
55	6	0	-0.593030	2.987480	0.336848
56	6	0	-1.302998	4.292355	-0.043047
57	8	0	-2.734247	4.221221	-0.113960
58	7	0	3.588975	2.038937	-0.759968
59	6	0	3.706030	2.822986	-1.844906
60	6	0	5.021930	3.272527	-1.976034
61	6	0	5.686846	2.715378	-0.886881
62	7	0	4.814124	1.985969	-0.166001
63	8	0	-0.895458	2.353431	1.493809
64	6	0	-2.210869	2.420755	2.075581
65	6	0	10.813940	-0.202560	-1.435617
66	6	0	-0.702693	4.930762	-1.291974
67	1	0	-5.360543	-1.278832	-2.760261
68	1	0	9.006190	-1.961180	-2.957162
69	1	0	5.214420	-3.149338	-1.417398
70	1	0	3.162819	2.071520	5.302768
71	1	0	-1.138752	4.970514	0.806284
72	1	0	-2.393570	1.251920	-3.485129
73	1	0	-0.612906	-0.671848	2.572583
74	1	0	-3.358168	-4.206219	-2.686090
75	1	0	-1.581071	-3.495954	-4.673952
76	1	0	7.427075	-1.137581	-2.940183

77	1	0	5.467841	1.786822	3.813778
78	1	0	1.168313	1.657932	3.443542
79	1	0	4.693857	-1.544138	2.606333
80	1	0	4.755625	-3.817723	1.163301
81	1	0	7.885741	-2.091224	3.149481
82	1	0	8.345450	0.139732	1.703734
83	1	0	8.500189	-3.282380	-1.002542
84	1	0	7.963860	-4.201489	1.465342
85	1	0	5.424045	-0.461287	-1.544375
86	1	0	5.434436	3.909735	-2.743872
87	1	0	6.210504	1.424488	1.478391
88	1	0	11.198060	0.393028	-2.271909
89	1	0	11.253271	0.190992	-0.513156
90	1	0	11.136364	-1.242751	-1.561405
91	1	0	8.804962	-0.394746	-3.771578
92	1	0	2.838921	3.025971	-2.459237
93	1	0	6.723579	2.769440	-0.588438
94	1	0	2.465319	-1.476426	-0.630980
95	1	0	1.302619	-1.955550	0.624538
96	1	0	0.894393	-2.156396	-1.095098
97	1	0	-1.693071	-2.880205	3.819987
98	1	0	-3.419367	-3.886603	1.917607
99	1	0	-0.491119	-1.151711	-3.725910
100	1	0	-4.136246	-3.473044	-0.455246
101	1	0	-4.712445	-0.257823	1.421159
102	1	0	-6.460355	1.525003	0.391815
103	1	0	-6.880367	0.872230	-2.193492
104	1	0	-9.387107	-1.151574	-1.347550
105	1	0	-6.461667	-3.966110	0.247432
106	1	0	-7.788828	-3.222217	-1.983406
107	1	0	-7.256739	-2.367497	2.261818
108	1	0	-10.326437	1.995287	1.122191
109	1	0	-8.889760	1.528618	0.166659
110	1	0	-10.501517	0.896235	-0.248632
111	1	0	-8.878354	1.225951	3.518356
112	1	0	-8.082777	-0.346393	3.640889
113	1	0	-7.506323	0.835420	2.440411
114	1	0	-1.287346	5.818211	-1.552922
115	1	0	0.331936	5.227080	-1.104494
116	1	0	-0.706740	4.233058	-2.132801
117	1	0	-3.576167	2.575075	-3.586654
118	1	0	-1.842990	2.936735	-3.394527
119	1	0	-3.629581	1.462535	-1.387210
120	1	0	-2.129377	1.881954	3.020433
121	1	0	-2.514835	3.453450	2.259836
122	1	0	-2.936125	1.927089	1.426689

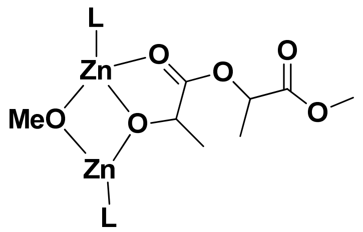

III^{dimer}-LA

Standard orientation:

Center Number	Atomic Number	Atomic Type	Coordinates (Angstroms) X	Y	Z
1	26	0	6.570533	-0.914018	0.833703
2	6	0	7.732491	-1.914410	-0.585517
3	6	0	8.061101	-0.515586	-0.550070
4	6	0	8.518576	-0.185586	0.758286
5	6	0	8.468988	-1.371297	1.552787
6	1	0	8.710963	-1.442701	2.605650
7	6	0	7.987876	-2.432933	0.731162
8	15	0	7.148261	-2.762536	-2.099202
9	6	0	5.375316	0.604603	1.597268
10	6	0	5.386473	-0.519986	2.480086
11	6	0	4.864923	-1.637647	1.759727
12	6	0	4.537185	-1.183381	0.443927
13	1	0	5.739432	1.594990	1.842884
14	5	0	4.663312	1.139515	-0.987409
15	7	0	4.126564	2.566828	-0.612964
16	7	0	2.880035	2.813502	-0.123201
17	6	0	2.870614	-0.840273	-3.624019
18	7	0	2.345877	0.322470	-1.771480
19	7	0	3.680263	0.460425	-2.003757
20	6	0	4.013341	-0.234412	-3.109060
21	6	0	2.819401	4.126416	0.158335
22	6	0	4.038944	4.743882	-0.133803
23	6	0	4.834089	3.713779	-0.624627
24	6	0	1.850817	-0.455914	-2.748780
25	1	0	-4.544949	-1.427597	-2.883041
26	6	0	-4.880631	-0.251896	0.240262
27	6	0	-3.868110	-2.009848	-0.822686
28	6	0	-5.137643	-0.116555	-1.159602
29	6	0	-8.001694	-2.012246	-0.791812
30	1	0	-7.330916	-3.223022	-2.560944
31	6	0	-7.686932	-2.174806	0.600041
32	6	0	-6.618697	-3.869478	-0.542122
33	1	0	-5.988717	-4.718598	-0.773469
34	15	0	-9.271857	-0.891044	-1.491054
35	1	0	-8.555082	-1.333123	-3.803168
36	1	0	-7.877609	0.976443	-0.586156
37	6	0	-8.932987	0.687644	-0.556009
38	6	0	-8.520993	-0.459869	-3.143659
39	5	0	-3.554302	-1.965525	1.895635
40	7	0	-3.187849	-0.757015	2.832787

41	7	0	-2.222789	0.143012	2.505682
42	6	0	-2.177556	1.055224	3.490497
43	1	0	-3.351312	1.303643	5.374186
44	6	0	-3.137756	0.763029	4.464060
45	6	0	-3.750402	-0.399169	4.002487
46	7	0	-2.273941	-2.860916	1.708449
47	6	0	-0.262477	-3.425476	1.088109
48	7	0	-1.106396	-2.385337	1.190953
49	6	0	-0.883419	-4.597220	1.529716
50	6	0	-2.156049	-4.186595	1.914829
51	30	0	-0.823422	-0.333780	1.036656
52	30	0	1.330179	1.378835	-0.269730
53	8	0	-0.614141	0.547359	-0.745987
54	6	0	-1.556020	1.391312	-1.332931
55	6	0	-1.130507	2.837009	-1.089501
56	8	0	-0.026584	3.141779	-0.641576
57	1	0	-0.728187	1.283785	-3.347356
58	6	0	-1.692787	1.144051	-2.847150
59	1	0	-2.430613	1.816354	-3.297026
60	6	0	-2.102992	4.990790	2.482326
61	8	0	-2.212480	4.698278	1.076088
62	6	0	-1.472899	5.473017	0.267353
63	6	0	-1.678992	5.137454	-1.210886
64	8	0	-2.043055	3.749660	-1.415404
65	6	0	-2.802455	5.976863	-1.809877
66	8	0	0.890412	0.542827	1.493971
67	6	0	4.839285	0.214850	0.324128
68	6	0	-4.512129	-1.216436	-1.821774
69	6	0	-4.078933	-1.422005	0.471228
70	6	0	-7.323595	-3.068079	-1.489785
71	6	0	-6.844478	-3.315635	0.752068
72	6	0	6.264039	-4.243403	-1.386609
73	6	0	8.733576	-3.609061	-2.629998
74	26	0	-5.916273	-1.919453	-0.468637
75	8	0	-0.766519	6.384840	0.634547
76	1	0	8.528264	-4.261952	-3.486333
77	1	0	-3.324847	-2.928072	-1.008765
78	1	0	5.359102	-3.914681	-0.867594
79	1	0	6.883214	-4.818347	-0.688145
80	1	0	4.301934	5.784831	-0.017836
81	1	0	5.758182	-0.532050	3.497052
82	1	0	8.804644	0.799984	1.102319
83	1	0	7.927749	0.177753	-1.370060
84	1	0	7.815150	-3.449711	1.058187
85	1	0	4.767873	-2.649462	2.133140
86	1	0	4.142993	-1.800239	-0.354177
87	1	0	2.795901	-1.464129	-4.502306
88	1	0	5.692043	1.333271	-1.590519
89	1	0	9.458571	-2.853343	-2.949677
90	1	0	9.180962	-4.206010	-1.826475
91	1	0	5.966455	-4.903830	-2.208650
92	1	0	0.795153	-0.687030	-2.757353
93	1	0	5.041780	-0.272918	-3.437193
94	1	0	1.900482	4.560644	0.527271

95	1	0	5.852640	3.715208	-0.984530
96	1	0	-2.553578	1.276344	-0.881303
97	1	0	-1.448798	1.853594	3.448856
98	1	0	-4.545836	-0.995485	4.425407
99	1	0	0.739819	-3.273546	0.709604
100	1	0	-0.470841	-5.594207	1.569757
101	1	0	-2.986694	-4.746157	2.319836
102	1	0	-4.342567	-2.636735	2.517302
103	1	0	-5.246992	0.414883	1.011429
104	1	0	-5.718005	0.666316	-1.629645
105	1	0	-6.406666	-3.660115	1.679201
106	1	0	-8.020321	-1.529277	1.402402
107	1	0	-9.125792	0.326587	-3.608680
108	1	0	-7.483754	-0.119994	-3.060188
109	1	0	-9.543514	1.488694	-0.987553
110	1	0	-9.237178	0.569511	0.489094
111	1	0	-2.019498	0.111950	-2.998064
112	1	0	-0.727423	5.331504	-1.710518
113	1	0	-2.567407	7.038689	-1.692501
114	1	0	-2.909151	5.754435	-2.875440
115	1	0	-3.751096	5.763584	-1.308152
116	1	0	-2.730698	4.252123	2.980246
117	1	0	-1.063620	4.902532	2.808477
118	1	0	-2.457632	6.004124	2.687250
119	6	0	1.702140	0.431390	2.647140
120	1	0	1.079246	0.340533	3.550144
121	1	0	2.366107	-0.441038	2.595434
122	1	0	2.334372	1.322001	2.759243

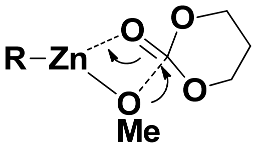

TSI-II^{monomer}-TMC

Standard orientation:

Center Number	Atomic Number	Atomic Type	Coordinates (Angstroms) X	Y	Z
1	8	0	4.114057	0.800200	-0.556480
2	6	0	3.604932	-0.483333	-0.571586
3	8	0	4.526644	-1.502732	-0.573369
4	6	0	5.736956	-1.345572	0.186456
5	6	0	6.331800	0.045350	0.011584
6	6	0	5.232469	1.058749	0.299349
7	8	0	2.607327	-0.643371	-1.334416
8	30	0	1.098428	0.049861	0.016515
9	15	0	-0.284820	-1.981883	-0.658759

10	6	0	-0.479007	-2.016744	-2.493418
11	7	0	0.529626	1.678909	-1.179463
12	6	0	1.191216	2.154394	-2.247165
13	6	0	0.679196	3.403110	-2.614051
14	6	0	-0.325091	3.650065	-1.684500
15	7	0	-0.400710	2.609360	-0.829851
16	5	0	-1.471678	2.417252	0.293707
17	6	0	-2.714418	1.505980	-0.196441
18	6	0	-2.915080	0.925299	-1.493165
19	6	0	-4.263115	0.472512	-1.621458
20	6	0	-4.926903	0.754460	-0.390084
21	6	0	-3.974078	1.370033	0.478572
22	26	0	-3.356864	-0.539816	-0.056808
23	6	0	-2.273476	-1.605348	1.354463
24	6	0	-1.951526	-2.063598	0.027479
25	6	0	-3.168566	-2.548166	-0.564113
26	6	0	-4.217022	-2.388755	0.386930
27	6	0	-3.664005	-1.812834	1.570298
28	8	0	2.867153	-0.502078	1.058026
29	6	0	2.872939	-1.732302	1.753474
30	7	0	0.157218	0.968353	1.715892
31	7	0	-0.770537	1.959494	1.619831
32	6	0	-1.005475	2.470127	2.845542
33	6	0	-0.219843	1.800016	3.776420
34	6	0	0.499229	0.876246	3.012148
35	6	0	0.456392	-3.649980	-0.335414
36	1	0	-3.278587	-2.933981	-1.568815
37	1	0	1.258086	0.170586	3.319173
38	1	0	0.998475	4.041473	-3.424518
39	1	0	2.004642	1.575501	-2.661345
40	1	0	-0.993296	4.491382	-1.571150
41	1	0	-4.172332	1.684573	1.496457
42	1	0	-5.951176	0.503847	-0.143708
43	1	0	-4.215852	-1.532927	2.458060
44	1	0	-1.581320	-1.144807	2.046105
45	1	0	-5.261064	-2.622406	0.222288
46	1	0	-4.694704	-0.023750	-2.481736
47	1	0	-2.154501	0.853812	-2.260349
48	1	0	-0.163922	1.972929	4.840965
49	1	0	-1.893352	3.528787	0.530434
50	1	0	-1.706626	3.282888	2.967131
51	1	0	-0.144162	-4.445322	-0.789723
52	1	0	1.467720	-3.670839	-0.753486
53	1	0	0.518904	-3.826374	0.742126
54	1	0	-0.997771	-2.920431	-2.830924
55	1	0	-1.036891	-1.137488	-2.822746

56	1	0	0.520329	-1.985637	-2.937516
57	1	0	5.539707	2.083266	0.075853
58	1	0	4.903150	1.003507	1.344110
59	1	0	7.178332	0.180136	0.695720
60	1	0	6.695368	0.175773	-1.014126
61	1	0	6.402933	-2.131910	-0.178234
62	1	0	5.525155	-1.537068	1.246141
63	1	0	3.645671	-1.741216	2.538281
64	1	0	1.904361	-1.901259	2.247390
65	1	0	3.063255	-2.578879	1.079681

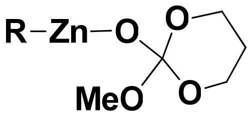

II^{monomer}-TMC

Standard orientation:

Center Number	Atomic Number	Atomic Type	Coordinates (Angstroms) X	Y	Z
1	6	0	-1.199588	3.955288	-1.203369
2	7	0	-1.006441	2.793818	-0.545561
3	7	0	0.004251	2.104978	-1.142851
4	6	0	0.446846	2.854790	-2.165171
5	6	0	-0.293134	4.038201	-2.254619
6	5	0	-1.860213	2.249124	0.646750
7	7	0	-0.909694	1.797258	1.813306
8	7	0	0.204313	1.027248	1.668318
9	6	0	0.736548	0.861036	2.891118
10	6	0	-0.042696	1.511865	3.851116
11	6	0	-1.069650	2.096674	3.117753
12	30	0	1.006558	0.505566	-0.194386
13	15	0	0.010698	-1.630623	-1.152781
14	6	0	-0.461924	-1.452777	-2.928409
15	8	0	2.997009	-0.503599	0.983636
16	6	0	3.672248	-0.018841	-0.219825
17	8	0	4.219745	-1.141767	-0.904489
18	6	0	5.266784	-1.822153	-0.197247
19	6	0	4.985313	-1.882121	1.314128
20	6	0	3.483661	-1.734943	1.535755
21	8	0	2.799611	0.607988	-0.972683
22	8	0	4.721959	0.794838	0.279318
23	6	0	5.333172	1.610418	-0.713710

24	6	0	1.130710	-3.105906	-1.192969
25	6	0	-1.479173	-2.165845	-0.287562
26	6	0	-2.656594	-2.820161	-0.789070
27	6	0	-3.557878	-3.002294	0.298769
28	6	0	-2.951088	-2.472075	1.476923
29	6	0	-1.676136	-1.950752	1.123219
30	26	0	-3.148445	-0.972978	0.036477
31	6	0	-4.977000	-0.020078	0.093157
32	6	0	-4.051627	0.663365	0.940531
33	6	0	-2.953551	1.161952	0.161858
34	6	0	-3.217292	0.732980	-1.181778
35	6	0	-4.452871	0.019576	-1.233218
36	1	0	-3.404397	-2.425273	2.458312
37	1	0	-2.843308	-3.098661	-1.817733
38	1	0	1.662063	0.313192	2.994382
39	1	0	-0.179545	4.843037	-2.965588
40	1	0	1.283651	2.509362	-2.757386
41	1	0	-1.968415	4.640334	-0.877007
42	1	0	-4.161256	0.783171	2.011898
43	1	0	-5.885236	-0.519243	0.407166
44	1	0	-0.989010	-1.439172	1.783627
45	1	0	-4.550286	-3.429338	0.232636
46	1	0	-4.894190	-0.438283	-2.109746
47	1	0	-2.578686	0.941956	-2.030416
48	1	0	0.124204	1.570768	4.916355
49	1	0	-2.453293	3.210819	1.084117
50	1	0	-1.896727	2.716726	3.431565
51	1	0	0.727655	-3.899438	-1.831258
52	1	0	2.116140	-2.793877	-1.553789
53	1	0	1.242660	-3.496742	-0.177353
54	1	0	-0.904922	-2.372935	-3.323795
55	1	0	-1.174348	-0.632824	-3.041093
56	1	0	0.438071	-1.218608	-3.505914
57	1	0	5.332179	-2.832656	1.737616
58	1	0	5.500256	-1.068731	1.831134
59	1	0	3.236827	-1.716538	2.602570
60	1	0	2.950165	-2.580597	1.079748
61	1	0	5.285051	-2.826741	-0.633171
62	1	0	6.234590	-1.345379	-0.396713
63	1	0	6.141913	2.147205	-0.210487
64	1	0	5.750298	1.005583	-1.530282
65	1	0	4.617259	2.325085	-1.131308

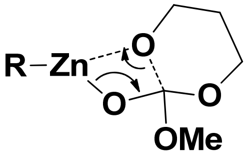

TSII-III^{monomer}-TMC

Standard orientation:

Center Number	Atomic Number	Atomic Type	Coordinates (Angstroms) X	Y	Z
1	7	0	0.563560	1.901949	-1.172259
2	7	0	-0.481521	2.735011	-0.911911
3	6	0	-0.384629	3.829764	-1.691881
4	6	0	0.753236	3.723043	-2.484012
5	6	0	1.316275	2.498725	-2.112068
6	5	0	-1.657946	2.389583	0.058555
7	7	0	-1.057445	2.012433	1.459371
8	7	0	0.010682	1.192902	1.649985
9	6	0	0.224860	1.119659	2.974677
10	6	0	-0.721710	1.887396	3.661882
11	6	0	-1.507654	2.440525	2.656145
12	30	0	1.165606	0.285927	0.090335
13	8	0	2.679995	-0.168656	1.560552
14	6	0	3.697623	-0.458940	0.889100
15	8	0	4.828728	0.282993	0.925615
16	6	0	4.641720	1.675661	1.194688
17	6	0	-2.667556	1.313902	-0.606975
18	6	0	-2.458753	0.483667	-1.760074
19	6	0	-3.686915	-0.140774	-2.138110
20	6	0	-4.686722	0.287468	-1.214259
21	6	0	-4.057986	1.166038	-0.280149
22	26	0	-3.183880	-0.714466	-0.212670
23	6	0	-3.671945	-1.601634	1.616285
24	6	0	-2.274569	-1.344876	1.540497
25	6	0	-1.747739	-2.061117	0.408124
26	6	0	-2.847572	-2.759510	-0.203635
27	6	0	-4.027175	-2.468499	0.540100
28	15	0	-0.019706	-2.000187	-0.126182
29	6	0	0.822834	-3.307507	0.879532
30	8	0	2.972412	0.053958	-0.847544
31	6	0	3.661224	-0.409881	-1.972397
32	6	0	4.132250	-1.862550	-1.787665
33	6	0	4.858148	-2.058134	-0.459967
34	8	0	3.972633	-1.765426	0.644616
35	6	0	-0.048300	-2.740614	-1.820263

36	1	0	1.055730	0.535420	3.344462
37	1	0	1.121433	4.433381	-3.209400
38	1	0	2.226135	2.021114	-2.444428
39	1	0	-1.132934	4.606305	-1.628436
40	1	0	-4.552658	1.643641	0.557230
41	1	0	-5.721899	-0.029779	-1.200273
42	1	0	-4.353862	-1.172309	2.338428
43	1	0	-1.711889	-0.699632	2.199815
44	1	0	-2.799154	-3.370480	-1.094810
45	1	0	-5.025398	-2.812096	0.301641
46	1	0	-3.828493	-0.836651	-2.955776
47	1	0	-1.509382	0.357887	-2.265899
48	1	0	-0.810869	2.038777	4.727558
49	1	0	-2.255525	3.427436	0.239490
50	1	0	-2.346053	3.119985	2.707574
51	1	0	0.385417	-4.295188	0.698596
52	1	0	1.891783	-3.313156	0.650132
53	1	0	0.713578	-3.057147	1.938814
54	1	0	-0.468423	-3.751913	-1.814243
55	1	0	-0.643120	-2.109875	-2.486582
56	1	0	0.974120	-2.789159	-2.206544
57	1	0	5.643962	2.105893	1.230403
58	1	0	4.061036	2.132792	0.388092
59	1	0	4.130246	1.819700	2.149498
60	1	0	4.550359	0.221393	-2.157791
61	1	0	3.033844	-0.341024	-2.877567
62	1	0	5.752052	-1.431005	-0.391359
63	1	0	5.149499	-3.100945	-0.310029
64	1	0	3.272220	-2.544278	-1.820335
65	1	0	4.805504	-2.145752	-2.608613

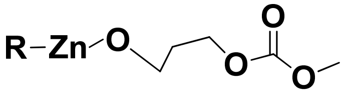

III^{monomer}-TMC

Standard orientation:

Center Number	Atomic Number	Atomic Type	Coordinates (Angstroms) X	Y	Z
1	7	0	0.000231	2.090315	-1.234561
2	7	0	-0.946637	2.811143	-0.570541
3	6	0	-1.132172	3.988467	-1.200268
4	6	0	-0.285784	4.054885	-2.301255

5	6	0	0.408415	2.842401	-2.271444
6	5	0	-1.812705	2.258499	0.611191
7	7	0	-0.885529	1.755060	1.774151
8	7	0	0.193612	0.940703	1.618699
9	6	0	0.660339	0.651723	2.846199
10	6	0	-0.127413	1.275865	3.818418
11	6	0	-1.089325	1.967644	3.089625
12	30	0	1.067192	0.545281	-0.239338
13	15	0	0.102809	-1.709881	-1.004362
14	6	0	1.171554	-3.198757	-0.761283
15	6	0	-2.908268	1.195520	0.079235
16	6	0	-3.084180	0.712200	-1.261505
17	6	0	-4.323502	0.011006	-1.366790
18	6	0	-4.939009	0.035738	-0.079788
19	6	0	-4.066640	0.747805	0.799080
20	26	0	-3.118325	-0.932611	0.030209
21	6	0	-2.991579	-2.367044	1.543836
22	6	0	-1.699270	-1.864985	1.227214
23	6	0	-1.440862	-2.135319	-0.162683
24	6	0	-2.597604	-2.810024	-0.688735
25	6	0	-3.547899	-2.945201	0.363802
26	8	0	2.953471	0.826223	-0.368685
27	6	0	3.794005	1.051132	-1.450050
28	6	0	4.285672	-0.244002	-2.126211
29	6	0	5.041886	-1.195312	-1.201711
30	8	0	4.158291	-1.793613	-0.220070
31	6	0	3.912668	-1.169761	0.940220
32	8	0	2.913938	-1.407720	1.592999
33	8	0	4.934749	-0.400302	1.342236
34	6	0	4.664779	0.438714	2.472874
35	6	0	-0.306536	-1.788113	-2.806444
36	1	0	1.527323	0.012157	2.944083
37	1	0	-0.183622	4.864475	-3.008458
38	1	0	1.187132	2.479265	-2.928876
39	1	0	-1.859372	4.693245	-0.824598
40	1	0	-4.249027	0.917960	1.853567
41	1	0	-5.874879	-0.437155	0.190567
42	1	0	-3.485246	-2.280702	2.502853
43	1	0	-1.036908	-1.339641	1.899959
44	1	0	-2.740672	-3.129120	-1.712327
45	1	0	-4.537571	-3.373484	0.270370
46	1	0	-4.711261	-0.479045	-2.251109
47	1	0	-2.377847	0.861226	-2.068256
48	1	0	-0.010734	1.242936	4.891537
49	1	0	-2.389664	3.221654	1.065784
50	1	0	-1.901928	2.601032	3.414515

51	1	0	0.645213	-4.109613	-1.065102
52	1	0	2.096232	-3.088441	-1.333697
53	1	0	1.452005	-3.259652	0.292922
54	1	0	-0.722254	-2.761455	-3.087115
55	1	0	-1.027091	-1.005017	-3.056556
56	1	0	0.607532	-1.620907	-3.385473
57	1	0	5.600711	0.960349	2.677790
58	1	0	3.878107	1.150600	2.213123
59	1	0	4.366345	-0.161637	3.336175
60	1	0	4.688051	1.610872	-1.114678
61	1	0	3.331523	1.681789	-2.236813
62	1	0	5.869753	-0.692682	-0.697096
63	1	0	5.436573	-2.050120	-1.758346
64	1	0	3.432722	-0.783094	-2.563585
65	1	0	4.959964	0.014647	-2.957082

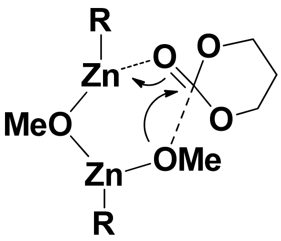

TSI-II^{dimer}-TMC

Standard orientation:

Center Number	Atomic Number	Atomic Type	Coordinates (Angstroms) X	Y	Z
1	6	0	-2.565372	1.478202	2.667863
2	7	0	-2.877315	1.484887	1.360321
3	7	0	-4.232080	1.590289	1.269432
4	6	0	-4.757163	1.628277	2.508964
5	6	0	-3.725766	1.557360	3.441527
6	30	0	-1.687940	1.652613	-0.336775
7	8	0	-0.251367	3.058993	-0.084637
8	6	0	0.785782	2.954158	0.601533
9	8	0	1.567884	4.038987	0.683830
10	6	0	2.828216	3.928205	1.379906
11	6	0	2.628950	3.216053	2.707563
12	6	0	1.971715	1.876245	2.422870
13	8	0	0.767946	2.062838	1.628033
14	5	0	-5.021666	1.534759	-0.090003
15	6	0	-4.961834	0.050892	-0.717549
16	6	0	-5.107874	-0.318686	-2.097914

17	6	0	-5.035981	-1.741466	-2.224144
18	6	0	-4.852489	-2.281178	-0.915283
19	6	0	-4.812560	-1.184229	0.000203
20	26	0	-6.631870	-1.225898	-1.012972
21	6	0	-8.351220	-0.203683	-0.478757
22	6	0	-8.445802	-0.621014	-1.837902
23	6	0	-8.344513	-2.044928	-1.867444
24	6	0	-8.196643	-2.504896	-0.525631
25	6	0	-8.201745	-1.366378	0.352874
26	15	0	-8.071465	-1.276230	2.177609
27	6	0	-7.291885	-2.923361	2.580756
28	8	0	-0.660181	0.108602	-1.009424
29	6	0	-1.346575	-1.077865	-1.408192
30	30	0	1.300888	-0.026490	-0.591961
31	8	0	2.031657	1.793116	-0.549352
32	6	0	2.342402	2.431615	-1.765512
33	7	0	2.306635	-1.348305	-1.884102
34	7	0	3.127368	-2.304466	-1.365779
35	6	0	3.508450	-3.141465	-2.350286
36	6	0	2.927483	-2.733606	-3.546631
37	6	0	2.178639	-1.606294	-3.196566
38	5	0	3.601069	-2.317099	0.133561
39	7	0	2.330895	-2.322732	1.062725
40	7	0	1.411144	-1.320835	1.054439
41	6	0	0.506370	-1.604695	2.006602
42	6	0	0.846321	-2.793512	2.659004
43	6	0	2.010039	-3.213130	2.021056
44	6	0	4.527277	-1.042873	0.472442
45	6	0	5.005373	-0.680359	1.779032
46	6	0	5.787762	0.512858	1.690581
47	6	0	5.813981	0.906325	0.317987
48	6	0	5.051003	-0.049133	-0.421038
49	26	0	6.645094	-0.987733	0.543569
50	6	0	7.718247	-2.560598	1.365293
51	6	0	7.294519	-2.892249	0.045299
52	6	0	7.866170	-1.940416	-0.851125
53	6	0	8.659037	-1.010824	-0.096761
54	6	0	8.550556	-1.404147	1.279951
55	15	0	9.828727	0.223571	-0.781764
56	6	0	8.844450	0.925865	-2.202316
57	6	0	9.666359	1.615343	0.450320
58	7	0	-3.122453	2.602209	-1.488029
59	7	0	-4.424817	2.592093	-1.088495
60	6	0	-5.100857	3.536693	-1.770324
61	6	0	-4.227367	4.189372	-2.635078
62	6	0	-2.995326	3.569698	-2.411161

63	6	0	-9.838932	-1.677871	2.651379
64	1	0	4.824178	-1.242723	2.687481
65	1	0	8.754808	0.175676	-2.994741
66	1	0	7.428211	-3.069378	2.275424
67	1	0	-1.621512	-1.697111	-0.543391
68	1	0	1.627770	1.371991	3.327721
69	1	0	1.553049	-0.974706	-3.813850
70	1	0	2.631775	-4.081886	2.181886
71	1	0	-4.786748	-3.331699	-0.661550
72	1	0	0.322910	-3.283021	3.466805
73	1	0	-6.261552	-2.944185	2.213780
74	1	0	-6.157315	3.676964	-1.593954
75	1	0	-2.027239	3.774777	-2.848383
76	1	0	-4.452321	4.996861	-3.315677
77	1	0	-5.274082	0.373553	-2.914545
78	1	0	-5.138911	-2.308388	-3.140824
79	1	0	-8.527875	0.028834	-2.699464
80	1	0	-8.340455	0.819368	-0.126381
81	1	0	-8.065575	-3.536820	-0.227703
82	1	0	-8.337015	-2.664112	-2.755600
83	1	0	-4.708790	-1.266423	1.075003
84	1	0	-3.809543	1.570586	4.517969
85	1	0	-6.135052	1.925549	0.163060
86	1	0	-9.910047	-1.776258	3.740820
87	1	0	-10.492761	-0.854996	2.344515
88	1	0	-10.197378	-2.603326	2.185749
89	1	0	-7.268588	-3.042512	3.669607
90	1	0	-7.837188	-3.772711	2.152561
91	1	0	-1.527458	1.441081	2.970034
92	1	0	-5.827859	1.685450	2.641179
93	1	0	-2.265150	-0.832283	-1.952360
94	1	0	-0.709333	-1.679883	-2.070060
95	1	0	2.845964	1.740538	-2.459220
96	1	0	1.443273	2.826513	-2.266706
97	1	0	3.023663	3.279596	-1.596895
98	1	0	3.028699	-3.190232	-4.519856
99	1	0	4.166804	-3.969480	-2.131369
100	1	0	-0.340246	-0.949015	2.161541
101	1	0	4.133657	-3.387304	0.311116
102	1	0	4.903726	-0.034895	-1.493264
103	1	0	6.334712	1.761380	-0.091791
104	1	0	6.302870	1.002170	2.508119
105	1	0	9.011348	-0.898632	2.119001
106	1	0	6.610812	-3.686690	-0.221119
107	1	0	7.713504	-1.915437	-1.922223
108	1	0	10.222712	2.482144	0.076503

109	1	0	8.624469	1.900010	0.628677
110	1	0	10.119991	1.319372	1.401885
111	1	0	9.391303	1.779284	-2.618548
112	1	0	7.840949	1.246608	-1.905253
113	1	0	3.522469	3.376903	0.739366
114	1	0	3.169173	4.958253	1.499694
115	1	0	3.594925	3.054642	3.199047
116	1	0	2.001740	3.821978	3.371900
117	1	0	2.643988	1.214206	1.873875

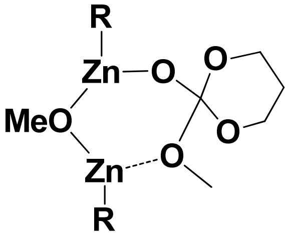

II^{dimer}-TMC

Standard orientation:

Center Number	Atomic Number	Atomic Type	Coordinates (Angstroms) X	Y	Z
1	6	0	-0.711342	-2.163021	-1.870140
2	6	0	-1.184750	-3.412456	-2.278430
3	6	0	-2.333294	-3.609310	-1.516823
4	7	0	-2.521711	-2.541407	-0.718671
5	7	0	-1.526861	-1.638420	-0.938700
6	5	0	-3.731042	-2.264741	0.250039
7	7	0	-3.169262	-2.006222	1.694669
8	7	0	-2.269177	-1.023633	1.977670
9	6	0	-2.070809	-1.040280	3.307280
10	6	0	-2.857196	-2.029645	3.903838
11	6	0	-3.533041	-2.616869	2.838661
12	30	0	-1.179339	-0.110095	0.427666
13	8	0	-1.955423	1.812147	0.280387
14	6	0	-2.396844	2.537230	1.435532
15	8	0	0.723404	0.043800	0.935631
16	6	0	1.398020	-1.102841	1.447104
17	30	0	1.687216	1.665294	0.280927
18	7	0	3.075008	2.596754	1.518009
19	7	0	4.394146	2.612501	1.179820
20	6	0	5.026151	3.547882	1.914801
21	6	0	4.105640	4.168253	2.754052
22	6	0	2.893573	3.539299	2.457371

23	5	0	5.049529	1.599128	0.171517
24	7	0	4.312260	1.689217	-1.212550
25	7	0	2.963910	1.561064	-1.353930
26	6	0	2.694893	1.602683	-2.669163
27	6	0	3.878323	1.736991	-3.399916
28	6	0	4.877002	1.789151	-2.431312
29	6	0	4.994413	0.093243	0.746089
30	6	0	4.844486	-1.116155	-0.013551
31	6	0	4.889240	-2.244812	0.862852
32	6	0	5.075303	-1.750633	2.189492
33	6	0	5.145401	-0.324056	2.112169
34	26	0	6.666979	-1.193304	0.993908
35	6	0	8.234144	-2.463984	0.492285
36	6	0	8.383701	-2.014461	1.837454
37	6	0	8.481115	-0.589971	1.819144
38	6	0	8.382767	-0.162071	0.463669
39	6	0	8.234677	-1.318442	-0.376908
40	15	0	8.104942	-1.213794	-2.200798
41	6	0	7.300794	-2.846349	-2.615418
42	6	0	9.866041	-1.639342	-2.677842
43	8	0	0.128098	2.874096	0.088574
44	6	0	-0.928700	2.583839	-0.582800
45	8	0	-1.562892	3.718426	-1.062184
46	6	0	-2.672501	3.521937	-1.948698
47	6	0	-2.238452	2.659040	-3.124829
48	6	0	-1.705991	1.345346	-2.572594
49	8	0	-0.674717	1.582321	-1.581934
50	6	0	-4.590620	-1.003855	-0.268104
51	6	0	-4.968197	-0.724994	-1.626683
52	6	0	-5.732181	0.482752	-1.674924
53	6	0	-5.848273	0.970142	-0.338109
54	6	0	-5.160310	0.054746	0.516960
55	26	0	-6.700025	-0.922954	-0.495218
56	6	0	-8.744412	-0.880591	0.042001
57	6	0	-8.012455	-1.801130	0.865756
58	6	0	-7.419354	-2.795124	0.031995
59	6	0	-7.768034	-2.499421	-1.317985
60	6	0	-8.575436	-1.322360	-1.313642
61	15	0	-9.918460	0.398667	0.630388
62	6	0	-8.969500	1.148593	2.050639
63	6	0	-9.696767	1.731976	-0.655621
64	1	0	-3.030241	-4.434294	-1.488721
65	1	0	4.825370	-3.286110	0.572848
66	1	0	8.563123	0.053264	2.685635
67	1	0	8.105076	-3.493778	0.186099
68	1	0	6.087360	3.706263	1.789557

69	1	0	-1.371342	-0.349108	3.759186
70	1	0	-0.756195	-4.079489	-3.011446
71	1	0	6.270445	-2.853561	-2.248082
72	1	0	1.903293	3.722668	2.852496
73	1	0	4.288442	4.961890	3.463051
74	1	0	5.313771	0.339650	2.951731
75	1	0	5.181998	-2.348975	3.085595
76	1	0	8.368125	0.863652	0.119469
77	1	0	8.379695	-2.640807	2.720612
78	1	0	4.739300	-1.159422	-1.090426
79	1	0	3.997389	1.796977	-4.471469
80	1	0	6.165848	2.016888	-0.020115
81	1	0	9.935325	-1.731521	-3.767963
82	1	0	10.532371	-0.828486	-2.365864
83	1	0	10.210858	-2.573178	-2.218610
84	1	0	7.275524	-2.957546	-3.705103
85	1	0	7.832985	-3.706993	-2.193218
86	1	0	1.667017	1.555780	-3.001720
87	1	0	5.949818	1.873603	-2.525618
88	1	0	2.318179	-0.808889	1.962670
89	1	0	0.759542	-1.636133	2.165592
90	1	0	1.671588	-1.802797	0.645638
91	1	0	-2.986486	3.403411	1.123122
92	1	0	-3.018246	1.857292	2.020716
93	1	0	-1.536612	2.874757	2.021921
94	1	0	-2.921831	-2.288046	4.950224
95	1	0	-4.247956	-3.426401	2.815359
96	1	0	0.167566	-1.618210	-2.188694
97	1	0	-4.338818	-3.306803	0.309563
98	1	0	-5.093785	0.134404	1.594966
99	1	0	-6.380489	1.859096	-0.026699
100	1	0	-6.175937	0.924670	-2.558285
101	1	0	-4.731469	-1.348311	-2.480824
102	1	0	-8.982019	-0.836120	-2.191071
103	1	0	-6.768443	-3.594196	0.360487
104	1	0	-7.445241	-3.045517	-2.194926
105	1	0	-7.914312	-1.743581	1.942029
106	1	0	-10.252651	2.621075	-0.337810
107	1	0	-8.646398	1.997076	-0.813673
108	1	0	-10.124676	1.400086	-1.607255
109	1	0	-9.511880	2.030561	2.409151
110	1	0	-8.921024	0.434111	2.878825
111	1	0	-7.950543	1.435350	1.772185
112	1	0	-1.225707	0.734577	-3.341253
113	1	0	-2.518893	0.761823	-2.126812
114	1	0	-3.085169	2.461117	-3.793110

115	1	0	-1.461262	3.179199	-3.696741
116	1	0	-3.508112	3.053111	-1.412205
117	1	0	-2.970366	4.526741	-2.258277

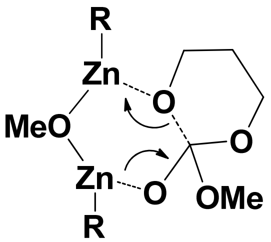

TSII-III[dimer]-TMC

Standard orientation:

Center Number	Atomic Number	Atomic Type	Coordinates (Angstroms) X	Y	Z
1	7	0	-4.578219	1.830268	0.889064
2	7	0	-3.319366	1.723395	1.398276
3	6	0	-3.386634	2.062944	2.696405
4	6	0	-4.701840	2.375972	3.048124
5	6	0	-5.420992	2.214520	1.866925
6	30	0	-1.680314	1.457224	0.146959
7	8	0	-0.324341	2.803957	0.772554
8	6	0	0.811620	2.966036	0.238458
9	8	0	0.904495	2.670814	-1.086706
10	6	0	2.167408	2.896969	-1.732899
11	5	0	-4.961941	1.410137	-0.576058
12	6	0	-4.861889	-0.190124	-0.745281
13	6	0	-4.660111	-0.934342	-1.956991
14	6	0	-4.685707	-2.335673	-1.669537
15	6	0	-4.915016	-2.483392	-0.267836
16	6	0	-5.025601	-1.172229	0.289343
17	26	0	-6.497383	-1.482316	-1.154290
18	6	0	-7.985875	-2.531717	-2.163432
19	6	0	-7.944949	-1.179380	-2.619902
20	6	0	-8.184660	-0.331823	-1.499689
21	6	0	-8.389035	-1.150321	-0.336178
22	6	0	-8.258982	-2.516969	-0.763768
23	15	0	-8.757795	-0.456681	1.318444
24	6	0	-10.621356	-0.645310	1.344708
25	8	0	-0.639568	-0.228947	0.192388
26	6	0	-1.307036	-1.476187	-0.001350

27	30	0	1.317248	-0.256442	0.564068
28	8	0	1.864061	1.608728	0.915573
29	6	0	2.704940	2.047759	1.974459
30	6	0	1.962298	3.078570	2.832194
31	6	0	1.614136	4.322939	2.000879
32	8	0	1.585565	4.023393	0.585238
33	7	0	1.885939	-1.567715	2.089323
34	6	0	1.293583	-1.899294	3.249425
35	6	0	1.911906	-3.021325	3.806564
36	6	0	2.913021	-3.346117	2.895691
37	7	0	2.884238	-2.468130	1.875137
38	5	0	3.909878	-2.370114	0.678227
39	7	0	3.113461	-2.347543	-0.673131
40	7	0	2.167645	-1.410809	-0.962424
41	6	0	1.754045	-1.639492	-2.220334
42	6	0	2.443003	-2.725454	-2.767493
43	6	0	3.290670	-3.142437	-1.745747
44	7	0	-2.664559	2.068501	-1.575159
45	7	0	-4.024053	2.149624	-1.597892
46	6	0	-4.403632	2.866601	-2.673019
47	6	0	-3.272045	3.273210	-3.373688
48	6	0	-2.205386	2.753739	-2.635659
49	6	0	-8.311786	-1.885402	2.432972
50	6	0	4.841823	-1.067075	0.831646
51	6	0	5.708928	-0.785106	1.942220
52	6	0	6.384362	0.454078	1.723041
53	6	0	5.950496	0.961851	0.461884
54	6	0	5.015113	0.028009	-0.082573
55	26	0	6.859832	-0.893670	0.214427
56	6	0	7.378458	-2.733218	-0.587679
57	6	0	8.227212	-2.422007	0.514541
58	6	0	8.919258	-1.212361	0.205562
59	6	0	8.512361	-0.764130	-1.096500
60	6	0	7.548170	-1.715297	-1.573460
61	15	0	9.298650	0.568121	-2.079663
62	6	0	7.826539	1.304127	-2.959013
63	6	0	9.582724	1.870648	-0.774020
64	1	0	5.853232	-1.430750	2.800344
65	1	0	-8.810616	-2.820968	2.153951
66	1	0	-5.017220	-3.418817	0.267447
67	1	0	-3.231930	3.865776	-4.275511
68	1	0	0.978339	-1.022182	-2.653793
69	1	0	3.644551	-4.140969	2.903576
70	1	0	1.667385	-3.528067	4.728149
71	1	0	-7.229395	-2.043154	2.414367
72	1	0	-5.453099	3.037234	-2.863823

73	1	0	-1.139312	2.850466	-2.787367
74	1	0	-4.537533	-0.500839	-2.942312
75	1	0	-4.587401	-3.138388	-2.389586
76	1	0	-7.720640	-0.855089	-3.627833
77	1	0	-8.163930	0.750165	-1.503452
78	1	0	-8.320009	-3.389666	-0.127058
79	1	0	-7.798009	-3.412331	-2.764442
80	1	0	-5.221615	-0.945859	1.330112
81	1	0	-5.079222	2.680660	4.012827
82	1	0	-6.064325	1.861710	-0.768226
83	1	0	-11.000031	-0.369649	2.335799
84	1	0	-11.064928	0.038226	0.613368
85	1	0	-10.944383	-1.666589	1.111178
86	1	0	-8.601761	-1.632927	3.458883
87	1	0	-2.487008	2.075924	3.297408
88	1	0	-6.475603	2.324629	1.659564
89	1	0	-0.588642	-2.256044	-0.287401
90	1	0	-1.819445	-1.800153	0.914567
91	1	0	-2.056710	-1.400617	-0.796754
92	1	0	2.336924	-3.153199	-3.753253
93	1	0	4.008122	-3.949096	-1.708445
94	1	0	0.452937	-1.322483	3.612488
95	1	0	4.519355	-3.413081	0.702306
96	1	0	4.532132	0.115226	-1.047369
97	1	0	6.284186	1.879177	-0.005014
98	1	0	7.117933	0.908100	2.377263
99	1	0	9.623821	-0.708033	0.854174
100	1	0	6.681052	-3.558562	-0.637769
101	1	0	8.299618	-2.976673	1.440973
102	1	0	7.021963	-1.661421	-2.517741
103	1	0	9.915786	2.792555	-1.263612
104	1	0	8.684239	2.075499	-0.182888
105	1	0	10.383599	1.548964	-0.100233
106	1	0	8.150715	2.204506	-3.492819
107	1	0	7.452480	0.595824	-3.705467
108	1	0	7.010924	1.559106	-2.274892
109	1	0	2.064201	2.470992	-2.732487
110	1	0	2.378712	3.967081	-1.800959
111	1	0	2.963808	2.388576	-1.185055
112	1	0	2.391959	5.086350	2.076721
113	1	0	0.655853	4.761415	2.294298
114	1	0	2.580670	3.371922	3.689627
115	1	0	1.050451	2.615539	3.222766
116	1	0	3.625225	2.485983	1.563159
117	1	0	3.003855	1.187042	2.584622

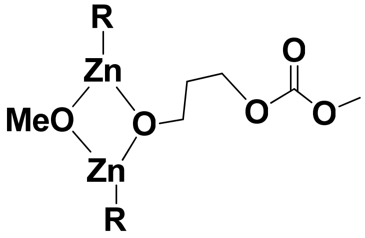

III^{dimer}-TMC

Standard orientation:

Center Number	Atomic Number	Atomic Type	Coordinates (Angstroms) X	Y	Z
1	26	0	7.014163	-1.106044	0.498989
2	6	0	8.879169	-1.869307	1.025413
3	6	0	8.499933	-0.835055	1.933750
4	6	0	8.307531	0.359380	1.181154
5	6	0	5.313481	-0.784169	-0.658260
6	6	0	5.648071	-2.654134	0.646466
7	7	0	3.897731	1.867422	0.053430
8	6	0	5.247019	-1.572297	1.490516
9	6	0	5.026298	-0.396097	0.695453
10	6	0	2.477640	2.313718	-1.537085
11	7	0	2.722217	1.476022	-0.514100
12	30	0	1.571010	0.027190	0.429466
13	7	0	2.379433	0.334327	2.315038
14	7	0	3.591076	0.944375	2.423087
15	5	0	4.591433	1.066725	1.214905
16	6	0	3.793702	1.299146	3.706092
17	6	0	2.689158	0.919673	4.463801
18	6	0	1.826513	0.323993	3.539683
19	30	0	-0.900085	-1.469670	-0.397938
20	7	0	-1.809437	-1.721221	-2.251157
21	7	0	-2.998931	-2.380719	-2.322887
22	5	0	-3.905292	-2.636694	-1.063811
23	6	0	-4.385442	-1.238622	-0.421162
24	26	0	-6.375428	-0.504038	-0.377109
25	6	0	-4.981923	1.011376	-0.149808
26	6	0	-8.184329	0.328066	-0.993516
27	6	0	-8.415256	-0.469701	0.177728
28	6	0	-7.989350	-1.802467	-0.145277
29	15	0	-9.359246	0.032015	1.666647
30	7	0	-1.917070	-3.022320	0.528491
31	7	0	-3.083916	-3.469232	-0.011018
32	6	0	-3.418878	-4.638003	0.567494

33	8	0	-0.430160	0.267399	0.441625
34	6	0	-1.215708	1.329351	0.946866
35	8	0	1.086753	-1.721127	-0.363300
36	6	0	1.865612	-2.791486	-0.859785
37	6	0	3.506194	3.251786	-1.650924
38	6	0	4.383549	2.927285	-0.619903
39	6	0	5.692119	-2.162038	-0.693586
40	6	0	8.928889	-1.308967	-0.285053
41	6	0	8.577201	0.082808	-0.203058
42	6	0	-2.449294	-4.975136	1.507796
43	6	0	-1.524134	-3.930085	1.437855
44	6	0	-3.271538	-2.660020	-3.611424
45	6	0	-2.238477	-2.179316	-4.410946
46	6	0	-1.342249	-1.604986	-3.505694
47	6	0	-4.494228	0.036877	-1.075706
48	6	0	-5.183554	0.352057	1.100958
49	6	0	-4.829144	-1.021525	0.927999
50	6	0	-7.520828	-1.823672	-1.492321
51	6	0	-7.641716	-0.503922	-2.018931
52	15	0	8.462561	1.356472	-1.515046
53	6	0	10.269108	1.825763	-1.674626
54	6	0	-8.406265	-0.827640	3.020834
55	6	0	-8.770537	1.785197	1.908287
56	6	0	8.307256	0.300990	-3.046156
57	1	0	-8.007692	-2.650697	0.526906
58	1	0	9.056639	-2.906470	1.279938
59	1	0	5.150697	-1.622963	2.568346
60	1	0	-5.192551	2.050772	-0.366928
61	1	0	-8.370990	1.390682	-1.079169
62	1	0	-7.332257	-0.177767	-3.003487
63	1	0	-9.189351	2.425926	1.125469
64	1	0	-7.331620	-0.625398	2.974153
65	1	0	-2.419878	-5.848740	2.141693
66	1	0	10.391446	2.517394	-2.516249
67	1	0	-4.273298	0.223003	-2.119607
68	1	0	7.342238	-0.214377	-3.042256
69	1	0	9.106129	-0.444283	-3.136588
70	1	0	2.535875	1.059620	5.523433
71	1	0	5.909536	-3.654042	0.968843
72	1	0	8.339891	-0.949691	2.998018
73	1	0	7.964279	1.308470	1.571578
74	1	0	9.158192	-1.853766	-1.191223
75	1	0	5.985974	-2.724328	-1.570853
76	1	0	5.274788	-0.121720	-1.514168
77	1	0	3.601597	4.050116	-2.371478
78	1	0	5.494578	1.762625	1.609763

79	1	0	10.590451	2.343521	-0.764955
80	1	0	10.918250	0.956256	-1.831070
81	1	0	8.342184	0.952613	-3.926120
82	1	0	1.577594	2.201391	-2.126955
83	1	0	5.327665	3.365438	-0.331040
84	1	0	0.843044	-0.103046	3.685175
85	1	0	4.710395	1.794490	3.991296
86	1	0	-0.600104	-3.785802	1.982010
87	1	0	-4.324617	-5.147146	0.271714
88	1	0	-0.390529	-1.123196	-3.687130
89	1	0	-2.150858	-2.242255	-5.485274
90	1	0	-4.182405	-3.179633	-3.870952
91	1	0	-4.792823	-3.366493	-1.434883
92	1	0	-4.902420	-1.787495	1.690316
93	1	0	-5.555737	0.806378	2.009645
94	1	0	-7.088248	-2.676970	-1.997332
95	1	0	-9.147872	2.152178	2.869168
96	1	0	-7.679539	1.871905	1.889453
97	1	0	-8.798153	-0.498563	3.989735
98	1	0	-8.564884	-1.908835	2.952067
99	1	0	1.375729	-3.754681	-0.653080
100	1	0	2.010901	-2.715100	-1.948501
101	1	0	2.855836	-2.807609	-0.389790
102	6	0	-1.340191	2.470571	-0.068307
103	1	0	-2.216391	0.958569	1.208146
104	1	0	-0.760604	1.711101	1.875452
105	6	0	-2.165131	3.628511	0.470319
106	1	0	-0.338550	2.829597	-0.336211
107	1	0	-1.809317	2.085717	-0.981913
108	8	0	-2.228265	4.633541	-0.566911
109	1	0	-3.182147	3.316914	0.727106
110	1	0	-1.714557	4.073229	1.364845
111	6	0	-2.981045	5.702547	-0.276235
112	8	0	-2.930724	6.540440	-1.325185
113	6	0	-3.697713	7.741740	-1.163761
114	1	0	-3.551412	8.303693	-2.086762
115	1	0	-4.756606	7.508923	-1.021221
116	1	0	-3.340697	8.316566	-0.304820
117	8	0	-3.601343	5.887161	0.749234

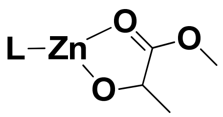

Cat^{monomer}- propagation

Standard orientation:

Center Number	Atomic Number	Atomic Type	Coordinates (Angstroms) X	Y	Z
1	6	0	-2.500633	1.259161	-0.657368
2	6	0	-2.303585	0.482252	-1.847928
3	6	0	-3.523680	-0.161665	-2.217586
4	6	0	-4.506809	0.199215	-1.248142
5	6	0	-3.874480	1.054802	-0.294667
6	26	0	-2.954847	-0.806431	-0.332337
7	6	0	-2.079444	-1.486379	1.420681
8	6	0	-3.462943	-1.816816	1.424989
9	6	0	-3.729294	-2.642043	0.291303
10	6	0	-2.508441	-2.833282	-0.416993
11	6	0	-1.471999	-2.116225	0.276958
12	15	0	0.273559	-1.941923	-0.153032
13	30	0	1.334808	0.340823	0.421472
14	8	0	3.177396	-0.353507	-1.017295
15	6	0	4.169063	-0.362434	-0.290240
16	8	0	5.394846	-0.616845	-0.760283
17	6	0	5.508160	-0.877077	-2.170351
18	5	0	-1.505259	2.358595	-0.010828
19	7	0	-1.036148	2.087331	1.455685
20	7	0	-0.027746	1.240874	1.789330
21	6	0	0.103219	1.283121	3.124605
22	6	0	-0.845052	2.151694	3.677355
23	6	0	-1.537830	2.645257	2.577098
24	7	0	-0.254220	2.603201	-0.928547
25	7	0	0.796475	1.745616	-1.070030
26	6	0	1.567888	2.228186	-2.059076
27	6	0	1.017571	3.402592	-2.579807
28	6	0	-0.135998	3.599305	-1.829119
29	6	0	0.381974	-2.413367	-1.935957
30	6	0	1.100401	-3.383507	0.673059
31	8	0	2.858342	0.282023	1.616390
32	6	0	4.116234	-0.075074	1.217226
33	6	0	4.656686	-1.302067	1.989122
34	1	0	-2.392031	-3.391905	-1.336025
35	1	0	0.890218	0.711179	3.596907
36	1	0	1.401561	4.023687	-3.375450
37	1	0	2.472935	1.703138	-2.329460
38	1	0	-0.886035	4.375185	-1.876270
39	1	0	-4.357156	1.478914	0.577863
40	1	0	-5.531781	-0.148679	-1.216793

41	1	0	-4.195448	-1.458946	2.136418
42	1	0	-1.575736	-0.839357	2.124935
43	1	0	-4.698570	-3.020490	-0.006600
44	1	0	-3.669578	-0.827038	-3.059449
45	1	0	-1.366067	0.408385	-2.384522
46	1	0	-0.991908	2.404663	4.717006
47	1	0	-2.084322	3.422276	0.030102
48	1	0	-2.342118	3.363713	2.511990
49	1	0	0.640656	-4.332862	0.378315
50	1	0	2.162452	-3.397621	0.406368
51	1	0	1.022098	-3.272964	1.758646
52	1	0	0.019735	-3.431551	-2.113785
53	1	0	-0.206694	-1.713986	-2.534427
54	1	0	1.427978	-2.344367	-2.248682
55	1	0	4.849816	0.744860	1.367654
56	1	0	5.682960	-1.556295	1.702751
57	1	0	4.631077	-1.069139	3.057489
58	1	0	4.010714	-2.171873	1.819632
59	1	0	6.567739	-1.061367	-2.348107
60	1	0	4.914911	-1.752065	-2.448840
61	1	0	5.168046	-0.013526	-2.747988

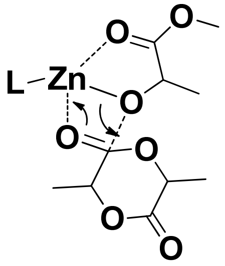

TSI-II^{monomer}- propagation-LA

Standard orientation:

Center Number	Atomic Number	Atomic Type	Coordinates (Angstroms) X	Y	Z
1	6	0	-4.044848	1.757159	-1.555872
2	6	0	-4.861061	0.877797	-2.322956
3	6	0	-4.057205	-0.225473	-2.739997
4	6	0	-2.743003	-0.035882	-2.228794
5	6	0	-2.720296	1.198024	-1.489596
6	26	0	-4.097444	-0.115634	-0.655717
7	6	0	-4.403639	-2.017555	0.116186
8	6	0	-3.427369	-1.367238	0.942295

9	6	0	-4.073916	-0.185527	1.437389
10	6	0	-5.413551	-0.123117	0.947680
11	6	0	-5.621094	-1.269855	0.124563
12	5	0	-2.014844	-1.994246	1.414315
13	7	0	-1.293564	-1.081094	2.463229
14	7	0	-0.594723	0.055563	2.185973
15	6	0	-0.306285	0.636282	3.362046
16	6	0	-0.838016	-0.106483	4.419857
17	6	0	-1.450844	-1.188688	3.798710
18	30	0	0.601624	0.177243	0.437052
19	8	0	1.500883	2.304152	0.855966
20	6	0	2.468374	2.588652	0.156605
21	8	0	3.088491	3.765786	0.225942
22	6	0	2.582856	4.716599	1.185541
23	15	0	-1.230451	1.904787	-0.737472
24	6	0	-0.768686	3.201471	-1.996391
25	8	0	2.265121	-0.641756	1.442532
26	6	0	3.154536	-0.730101	0.545789
27	6	0	4.565019	-0.274870	0.919959
28	8	0	5.482275	-0.290765	-0.201102
29	6	0	5.291653	-1.123740	-1.249306
30	6	0	4.040594	-2.002418	-1.296389
31	8	0	3.181132	-1.921929	-0.157147
32	6	0	5.121626	-1.136597	2.051584
33	6	0	4.411909	-3.470387	-1.495945
34	8	0	6.092155	-1.136623	-2.155108
35	8	0	2.563605	0.355255	-0.734842
36	6	0	3.090830	1.643844	-0.876273
37	6	0	2.870995	2.164430	-2.307655
38	7	0	-0.229932	-1.562684	-0.445615
39	7	0	-1.065293	-2.397226	0.230076
40	6	0	-1.103138	-3.589602	-0.399356
41	6	0	-0.278571	-3.542332	-1.518193
42	6	0	0.254100	-2.250270	-1.491817
43	6	0	-1.872515	2.991899	0.618488
44	1	0	-1.029113	3.539739	1.049951
45	1	0	-4.376490	2.672079	-1.083131
46	1	0	0.974987	-1.783609	-2.148587
47	1	0	-0.776545	0.099246	5.478219
48	1	0	0.284526	1.542274	3.378906
49	1	0	-1.996581	-2.024006	4.212350
50	1	0	-4.237863	-2.929183	-0.445175
51	1	0	-6.520066	-1.502963	-0.432406
52	1	0	-4.399987	-1.084671	-3.301704
53	1	0	-1.914376	-0.722544	-2.327567
54	1	0	-5.919432	1.001584	-2.513675

55	1	0	-6.128247	0.669216	1.132242
56	1	0	-3.611470	0.545073	2.088929
57	1	0	-0.082368	-4.332154	-2.228167
58	1	0	-2.238431	-3.031181	2.001545
59	1	0	-1.713808	-4.389308	-0.006131
60	1	0	-1.613632	3.869083	-2.196788
61	1	0	0.076283	3.798256	-1.636747
62	1	0	-0.478720	2.720870	-2.935739
63	1	0	-2.618002	3.708462	0.258047
64	1	0	-2.314500	2.371516	1.400204
65	1	0	3.255620	3.182918	-2.423386
66	1	0	3.402970	1.507830	-3.001547
67	1	0	1.808897	2.149618	-2.565190
68	1	0	3.253784	5.572746	1.123528
69	1	0	1.561860	5.009932	0.928669
70	1	0	2.596203	4.285188	2.189029
71	1	0	4.173444	1.658419	-0.693979
72	1	0	3.480612	-1.642128	-2.168469
73	1	0	5.019935	-3.579102	-2.396994
74	1	0	4.977009	-3.848523	-0.637762
75	1	0	3.498797	-4.064232	-1.595271
76	1	0	4.536186	0.768741	1.241927
77	1	0	6.142066	-0.820105	2.287855
78	1	0	4.492178	-1.029816	2.938538
79	1	0	5.134627	-2.191662	1.761570

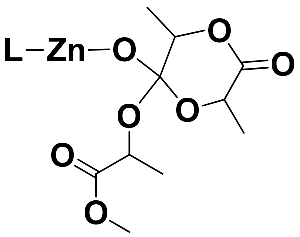

II^{monomer}- propagation-LA

Standard orientation:

Center Number	Atomic Number	Atomic Type	Coordinates (Angstroms) X	Y	Z
1	6	0	-0.310689	-2.645679	1.750021
2	7	0	-0.725151	-1.385975	1.531301
3	7	0	-1.849018	-1.197081	2.275602
4	6	0	-2.131789	-2.333204	2.943536
5	6	0	-1.176505	-3.295493	2.633618

6	30	0	0.255078	0.196017	0.552166
7	8	0	2.158244	0.486149	0.934737
8	6	0	3.031826	-0.338071	0.378426
9	6	0	4.094608	-0.846130	1.378931
10	8	0	4.980341	-1.819530	0.760264
11	6	0	4.599937	-2.570970	-0.289616
12	6	0	3.197340	-2.392233	-0.884256
13	8	0	2.358958	-1.495890	-0.144560
14	8	0	3.710722	0.222952	-0.779297
15	6	0	4.196473	1.536664	-0.617890
16	6	0	5.344045	1.733311	-1.615368
17	6	0	3.504420	-1.442658	2.649332
18	8	0	5.371346	-3.369133	-0.772600
19	6	0	2.489643	-3.739984	-0.993062
20	5	0	-2.717054	0.111918	2.283245
21	7	0	-1.788445	1.355695	2.500531
22	7	0	-0.694470	1.647030	1.744093
23	6	0	-0.192277	2.805327	2.203414
24	6	0	-0.973984	3.291414	3.255267
25	6	0	-1.971436	2.334811	3.409016
26	6	0	-3.702759	0.299243	1.017480
27	6	0	-4.863572	-0.497835	0.736474
28	6	0	-5.645301	0.126986	-0.282783
29	6	0	-4.966911	1.320272	-0.672491
30	6	0	-3.781199	1.414630	0.117703
31	26	0	-3.777372	-0.339988	-1.026690
32	6	0	-2.034090	-0.579352	-2.123124
33	6	0	-3.134051	-0.285218	-3.001664
34	6	0	-4.112114	-1.308345	-2.845451
35	6	0	-3.630256	-2.243143	-1.880994
36	6	0	-2.356453	-1.799299	-1.430551
37	15	0	-0.518869	0.370768	-1.854901
38	6	0	-0.904828	2.070645	-2.456185
39	6	0	0.696213	-0.244904	-3.103620
40	6	0	3.107054	2.574587	-0.892614
41	8	0	3.347790	3.707303	-0.201937
42	6	0	2.473082	4.812603	-0.479682
43	8	0	2.195193	2.454964	-1.685273
44	1	0	0.005494	2.668486	-2.374543
45	1	0	-3.221591	0.578494	-3.646782
46	1	0	0.589282	-2.999574	1.268167
47	1	0	-0.831058	4.193836	3.830780
48	1	0	0.716173	3.204112	1.772401
49	1	0	-2.798296	2.281216	4.101889
50	1	0	-5.108385	-1.435431	1.221247
51	1	0	-6.560430	-0.257830	-0.715054

52	1	0	-4.161726	-3.112666	-1.517176
53	1	0	-1.750772	-2.269097	-0.669077
54	1	0	-5.073754	-1.344080	-3.340951
55	1	0	-5.277118	2.007875	-1.449278
56	1	0	-3.051084	2.211975	0.059001
57	1	0	-1.111980	-4.306605	3.007146
58	1	0	-3.387991	0.035050	3.288340
59	1	0	-2.989324	-2.374882	3.599129
60	1	0	0.322218	-0.098604	-4.122430
61	1	0	1.633193	0.300864	-2.961685
62	1	0	0.876040	-1.310742	-2.939018
63	1	0	-1.235085	2.053923	-3.500276
64	1	0	-1.691440	2.511541	-1.839169
65	1	0	5.755437	2.745146	-1.540134
66	1	0	6.135751	1.009356	-1.403017
67	1	0	4.985985	1.568879	-2.636547
68	1	0	2.857534	5.645457	0.109623
69	1	0	2.486887	5.056271	-1.545385
70	1	0	1.448003	4.576495	-0.180751
71	1	0	4.562935	1.709156	0.400367
72	1	0	3.358063	-1.981671	-1.888680
73	1	0	3.095737	-4.421885	-1.593474
74	1	0	2.342969	-4.187178	-0.003913
75	1	0	1.511226	-3.613616	-1.467890
76	1	0	4.758333	-0.017470	1.642952
77	1	0	4.307751	-1.819793	3.289820
78	1	0	2.941175	-0.679270	3.191585
79	1	0	2.827776	-2.269734	2.414458

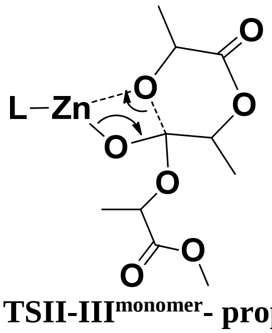

TSII-III^{monomer}- propagation-LA

Standard orientation:

Center Number	Atomic Number	Atomic Type	Coordinates (Angstroms) X	Y	Z
1	6	0	-2.733163	-2.074773	0.678073

2	6	0	-4.105418	-2.438658	0.769414
3	6	0	-4.617726	-2.583345	-0.554786
4	6	0	-3.560894	-2.315901	-1.471500
5	6	0	-2.379989	-1.997092	-0.714499
6	26	0	-3.898461	-0.645441	-0.283251
7	6	0	-4.647090	0.905216	0.881851
8	6	0	-3.402711	1.352532	0.323553
9	6	0	-3.568392	1.256625	-1.098836
10	6	0	-4.883189	0.791703	-1.405407
11	6	0	-5.558729	0.575006	-0.167342
12	5	0	-2.206189	2.081826	1.131696
13	7	0	-1.442843	1.160891	2.148249
14	7	0	-0.503572	0.241278	1.804181
15	6	0	-0.199051	-0.452760	2.913147
16	6	0	-0.952849	0.010872	3.996965
17	6	0	-1.722184	1.039281	3.461925
18	30	0	0.496805	0.262353	-0.076020
19	8	0	1.891393	0.082795	-2.050692
20	6	0	2.931759	-0.223045	-1.457601
21	8	0	3.133298	-1.572547	-1.244226
22	6	0	3.984247	-2.058546	-0.207530
23	6	0	3.227067	-2.136236	1.124244
24	8	0	4.070843	-1.845825	2.133633
25	6	0	3.552296	-2.011850	3.464240
26	15	0	-0.731920	-1.576674	-1.332888
27	6	0	0.132568	-3.203660	-1.495269
28	7	0	-1.200304	2.779471	0.154777
29	7	0	-0.271904	2.131617	-0.607696
30	6	0	0.247678	3.034816	-1.456973
31	6	0	-0.354724	4.280855	-1.265687
32	6	0	-1.262115	4.069450	-0.234577
33	8	0	2.475391	0.314684	0.441100
34	6	0	3.207305	1.395975	0.969749
35	6	0	3.447373	2.472517	-0.104868
36	8	0	3.312758	3.659196	0.067018
37	6	0	2.558364	2.016949	2.206454
38	6	0	-0.996521	-1.094511	-3.094280
39	6	0	4.177313	0.662703	-1.558122
40	8	0	3.828261	2.034941	-1.344475
41	6	0	4.769631	0.577972	-2.963117
42	6	0	4.440139	-3.459774	-0.617711
43	8	0	2.089957	-2.530976	1.270917
44	1	0	-0.461085	-3.901990	-2.094585
45	1	0	4.990184	-3.412423	-1.562158
46	1	0	4.207438	1.025807	1.268240
47	1	0	-3.643969	-2.319332	-2.549990

48	1	0	0.536709	-1.243953	2.856629
49	1	0	-0.145161	5.205308	-1.782353
50	1	0	1.038196	2.738226	-2.132424
51	1	0	-1.954711	4.744480	0.246642
52	1	0	-4.862647	0.825209	1.940843
53	1	0	-6.562271	0.186837	-0.045723
54	1	0	-4.673313	-2.538318	1.685103
55	1	0	-2.075580	-1.859497	1.507759
56	1	0	-5.642305	-2.810934	-0.819450
57	1	0	-5.283216	0.602859	-2.393807
58	1	0	-2.808208	1.511568	-1.826377
59	1	0	-0.937232	-0.337089	5.019332
60	1	0	-2.674565	2.973420	1.804988
61	1	0	-2.447679	1.693174	3.923610
62	1	0	1.105222	-3.034611	-1.964426
63	1	0	0.304451	-3.621551	-0.500434
64	1	0	-1.448442	-1.907957	-3.671628
65	1	0	-1.643575	-0.215083	-3.143643
66	1	0	-0.022493	-0.840490	-3.519516
67	1	0	4.853708	-1.414218	-0.065002
68	1	0	5.094422	-3.885448	0.149970
69	1	0	3.572896	-4.113803	-0.746046
70	1	0	4.408937	-1.897071	4.128908
71	1	0	2.804687	-1.243281	3.675549
72	1	0	3.101237	-3.000553	3.579749
73	1	0	4.931167	0.371352	-0.821587
74	1	0	3.192962	2.808528	2.615235
75	1	0	1.587128	2.455621	1.961041
76	1	0	2.410168	1.245409	2.967381
77	1	0	5.641952	1.233474	-3.038110
78	1	0	5.075623	-0.449168	-3.187253
79	1	0	4.024128	0.892465	-3.698325

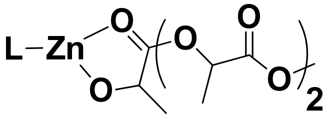

III^{monomer}- propagation-LA

Standard orientation:

Center Number	Atomic Number	Atomic Type	Coordinates (Angstroms) X	Y	Z
1	7	0	-1.063621	0.973258	2.133342

2	7	0	-1.192981	-0.291779	2.621437
3	6	0	-1.166928	-0.250261	3.969113
4	6	0	-1.009942	1.067732	4.382621
5	6	0	-0.942614	1.793842	3.189989
6	5	0	-1.534223	-1.559076	1.762386
7	7	0	-0.496391	-1.718212	0.589441
8	7	0	-0.068627	-0.724392	-0.235130
9	6	0	0.781499	-1.276735	-1.115087
10	6	0	0.900899	-2.651529	-0.880691
11	6	0	0.076408	-2.880577	0.216200
12	30	0	-0.445073	1.337382	0.153539
13	8	0	1.818684	1.455042	0.697521
14	6	0	2.267123	2.486626	0.207443
15	8	0	3.572779	2.782559	0.247437
16	6	0	4.432265	1.791459	0.844485
17	6	0	5.852880	2.345717	0.798983
18	6	0	-3.096104	-1.580668	1.344194
19	6	0	-3.840269	-2.737859	0.932232
20	6	0	-5.235793	-2.433294	0.904602
21	6	0	-5.383056	-1.065792	1.284584
22	6	0	-4.076443	-0.550801	1.544486
23	26	0	-4.230171	-1.219877	-0.430436
24	6	0	-4.031253	-1.985885	-2.362555
25	6	0	-5.275494	-1.298845	-2.234267
26	6	0	-5.001804	0.053922	-1.879037
27	6	0	-3.577031	0.212523	-1.776398
28	6	0	-2.985050	-1.064338	-2.077258
29	15	0	-2.604829	1.665960	-1.306518
30	6	0	-2.180785	2.479485	-2.917287
31	8	0	0.069393	3.108205	-0.490192
32	6	0	1.380157	3.519883	-0.494032
33	6	0	1.915796	3.777581	-1.919748
34	6	0	-3.836805	2.850140	-0.599499
35	6	0	4.343666	0.481881	0.060581
36	8	0	4.087484	0.383880	-1.116849
37	8	0	4.654185	-0.549265	0.873379
38	6	0	4.756811	-1.836306	0.248013
39	6	0	4.731147	-2.881782	1.359721
40	6	0	6.055049	-1.887522	-0.560943
41	8	0	6.952718	-1.079711	-0.496744
42	8	0	6.067436	-2.985462	-1.339843
43	6	0	7.250467	-3.162034	-2.140072
44	1	0	-3.316226	3.784944	-0.369214
45	1	0	-3.852269	0.463493	1.850593
46	1	0	-3.073499	2.793401	-3.469168
47	1	0	3.787508	-2.810476	1.907712

48	1	0	1.522593	4.457554	0.083379
49	1	0	-5.743074	0.817566	-1.685083
50	1	0	1.283571	-0.658644	-1.847468
51	1	0	-0.946941	1.439683	5.394450
52	1	0	-0.802431	2.855516	3.033418
53	1	0	-1.271006	-1.161629	4.539434
54	1	0	-3.407805	-3.697621	0.674972
55	1	0	-6.032334	-3.103100	0.605495
56	1	0	-3.905370	-3.036576	-2.589174
57	1	0	-1.928232	-1.288393	-2.049311
58	1	0	-6.258901	-1.737355	-2.345846
59	1	0	-6.312244	-0.511835	1.331815
60	1	0	1.495926	-3.371580	-1.423172
61	1	0	-1.315645	-2.503350	2.489770
62	1	0	-0.138649	-3.788456	0.760612
63	1	0	-1.542038	3.339350	-2.698551
64	1	0	-1.611902	1.778016	-3.535728
65	1	0	-4.653551	3.062977	-1.297939
66	1	0	-4.253466	2.444792	0.326509
67	1	0	3.913083	-1.973731	-0.433233
68	1	0	4.816829	-3.883464	0.929799
69	1	0	5.557944	-2.722752	2.059229
70	1	0	7.088258	-4.082090	-2.701707
71	1	0	7.380117	-2.313969	-2.817021
72	1	0	8.134091	-3.249756	-1.502511
73	1	0	4.107204	1.604913	1.871113
74	1	0	2.943230	4.155625	-1.912805
75	1	0	1.884865	2.853575	-2.508182
76	1	0	1.266746	4.514284	-2.402450
77	1	0	5.914892	3.268332	1.383384
78	1	0	6.550126	1.609924	1.209129
79	1	0	6.139926	2.558658	-0.234760

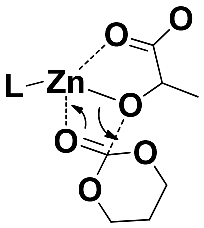

TSI-II^{monomer}- propagation-TMC

Standard orientation:

Center Atomic Atomic Coordinates (Angstroms)

Number	Number	Type	X	Y	Z
1	6	0	-3.597896	2.063711	-1.416935
2	6	0	-4.546486	1.295644	-2.151389
3	6	0	-3.891947	0.120709	-2.629861
4	6	0	-2.539500	0.154521	-2.188538
5	6	0	-2.341317	1.362264	-1.433359
6	26	0	-3.810592	0.188149	-0.546582
7	6	0	-3.659991	0.051934	1.538469
8	6	0	-3.200676	-1.183517	0.971165
9	6	0	-4.304293	-1.681969	0.201309
10	6	0	-5.414528	-0.789408	0.310474
11	6	0	-5.010160	0.295909	1.143637
12	5	0	-1.847550	-1.995374	1.323590
13	7	0	-0.998070	-2.406633	0.067488
14	7	0	-0.090072	-1.627209	-0.580517
15	6	0	0.256765	-2.275619	-1.703167
16	6	0	-0.439287	-3.483487	-1.807005
17	6	0	-1.215341	-3.525319	-0.653962
18	30	0	0.957169	-0.040309	0.374035
19	8	0	2.964880	0.090674	-0.954901
20	6	0	3.638001	1.306760	-0.816077
21	6	0	3.639720	2.074699	-2.149179
22	15	0	-0.750548	1.872070	-0.728064
23	6	0	-1.222914	3.044244	0.627313
24	8	0	2.645362	-0.931415	1.171523
25	6	0	3.417210	-1.122411	0.184444
26	8	0	4.763579	-0.911415	0.456566
27	6	0	5.742966	-1.278031	-0.526202
28	6	0	5.442016	-2.635683	-1.144273
29	6	0	4.002726	-2.597654	-1.636103
30	8	0	3.139059	-2.263546	-0.549341
31	8	0	1.976831	1.937783	0.875340
32	6	0	3.037364	2.164910	0.301763
33	8	0	3.796784	3.219739	0.596767
34	6	0	3.338935	4.075579	1.662239
35	7	0	-0.184141	-0.170737	2.157648
36	7	0	-0.986967	-1.246256	2.394046
37	6	0	-1.094392	-1.434367	3.725563
38	6	0	-0.341558	-0.469923	4.385211
39	6	0	0.218395	0.288666	3.353441
40	6	0	-0.143041	3.078938	-2.013101
41	1	0	-3.800001	2.996194	-0.907162
42	1	0	1.005868	-1.849594	-2.355473
43	1	0	-0.208032	-0.350825	5.450205
44	1	0	0.907079	1.121277	3.399582

45	1	0	-1.706942	-2.236908	4.109520
46	1	0	-4.292192	-2.589865	-0.390007
47	1	0	-6.370963	-0.892287	-0.186987
48	1	0	-4.357368	-0.684569	-3.183163
49	1	0	-1.796276	-0.614098	-2.345044
50	1	0	-5.593486	1.538231	-2.281307
51	1	0	-5.606061	1.164007	1.396860
52	1	0	-3.070162	0.696273	2.178114
53	1	0	-0.374093	-4.228111	-2.586602
54	1	0	-2.160575	-3.044299	1.845378
55	1	0	-1.906349	-4.274170	-0.294982
56	1	0	-0.902057	3.834451	-2.244088
57	1	0	0.760688	3.583321	-1.654736
58	1	0	0.103619	2.541039	-2.934035
59	1	0	-1.875667	3.850584	0.275913
60	1	0	-1.729326	2.489686	1.420200
61	1	0	-0.308538	3.478785	1.042054
62	1	0	4.170036	3.028414	-2.060909
63	1	0	4.138696	1.461462	-2.905317
64	1	0	2.617454	2.263558	-2.488928
65	1	0	4.114345	4.832396	1.777687
66	1	0	2.386275	4.540430	1.395707
67	1	0	3.219217	3.500234	2.582962
68	1	0	4.678080	1.145017	-0.506742
69	1	0	3.656812	-3.570835	-1.994019
70	1	0	3.884101	-1.860672	-2.442405
71	1	0	6.137276	-2.837151	-1.968412
72	1	0	5.555155	-3.427441	-0.395200
73	1	0	6.697845	-1.264358	0.006417
74	1	0	5.780018	-0.512294	-1.315524

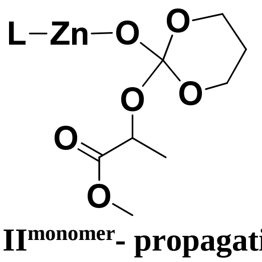

II^{monomer}- propagation-TMC

Standard orientation:

Center Number	Atomic Number	Atomic Type	Coordinates (Angstroms) X	Y	Z

1	6	0	1.508932	-0.607161	4.115799
2	7	0	1.443749	-0.123793	2.858785
3	7	0	0.309347	-0.583172	2.262514
4	6	0	-0.335619	-1.339852	3.164958
5	6	0	0.390842	-1.397640	4.358058
6	5	0	2.520371	0.762288	2.145004
7	6	0	3.433248	-0.097017	1.125994
8	6	0	4.691551	0.318631	0.573368
9	6	0	5.348116	-0.796902	-0.030602
10	6	0	4.492135	-1.929046	0.116890
11	6	0	3.325214	-1.493818	0.815224
12	26	0	3.553458	-0.446806	-0.987367
13	6	0	1.839834	-0.476835	-2.154958
14	6	0	2.867593	-1.276104	-2.764590
15	6	0	3.986998	-0.435042	-3.027390
16	6	0	3.664671	0.885326	-2.591967
17	6	0	2.349328	0.865762	-2.050555
18	15	0	0.208586	-0.983919	-1.561954
19	6	0	-0.943610	-0.755498	-2.988080
20	30	0	-0.467320	0.293481	0.516174
21	8	0	-2.401886	0.237803	0.797497
22	6	0	-3.115733	0.944796	-0.043627
23	8	0	-2.247659	1.699805	-0.906370
24	6	0	-2.909582	2.596000	-1.811025
25	6	0	-3.826747	3.538616	-1.033859
26	6	0	-4.744734	2.705689	-0.139591
27	8	0	-3.978381	1.814691	0.671315
28	8	0	-3.910798	0.149037	-0.953047
29	6	0	-4.650295	-0.865281	-0.302774
30	6	0	-3.778268	-2.095192	-0.039457
31	8	0	-2.906683	-2.515095	-0.773845
32	7	0	1.825618	2.034813	1.538171
33	6	0	2.252453	3.306567	1.668214
34	6	0	1.392350	4.150668	0.973491
35	6	0	0.427960	3.291941	0.438963
36	7	0	0.699430	2.019513	0.774105
37	6	0	0.297370	-2.818703	-1.397659
38	6	0	-5.807383	-1.265259	-1.223431
39	8	0	-4.178904	-2.734118	1.078330
40	6	0	-3.504579	-3.966778	1.369865
41	1	0	2.484675	-2.120381	1.085512
42	1	0	-4.416095	4.153323	-1.726197
43	1	0	2.817196	-2.339541	-2.955634
44	1	0	-0.454788	3.508731	-0.145297
45	1	0	0.137073	-1.920245	5.268297

46	1	0	-1.295580	-1.770592	2.913627
47	1	0	2.345931	-0.353431	4.749670
48	1	0	5.083609	1.328219	0.607232
49	1	0	6.300095	-0.777789	-0.546243
50	1	0	4.324280	1.742740	-2.620266
51	1	0	1.836177	1.703164	-1.598802
52	1	0	4.934448	-0.754605	-3.441804
53	1	0	4.680018	-2.926267	-0.261048
54	1	0	1.448814	5.225680	0.888098
55	1	0	3.227183	1.187239	3.031963
56	1	0	3.132725	3.523177	2.255692
57	1	0	-0.626925	-1.352824	-3.849765
58	1	0	-1.943916	-1.054771	-2.663324
59	1	0	-0.966343	0.300794	-3.268781
60	1	0	0.593797	-3.287297	-2.342303
61	1	0	1.017395	-3.090843	-0.621995
62	1	0	-0.697293	-3.173987	-1.117314
63	1	0	-6.401424	-2.066996	-0.772181
64	1	0	-6.457138	-0.402503	-1.397648
65	1	0	-5.418147	-1.610910	-2.186421
66	1	0	-4.002451	-4.372852	2.250974
67	1	0	-3.583905	-4.660327	0.528233
68	1	0	-2.447044	-3.784437	1.581929
69	1	0	-5.035150	-0.504699	0.656403
70	1	0	-2.113698	3.136047	-2.333254
71	1	0	-3.478061	2.014779	-2.547736
72	1	0	-3.223608	4.210805	-0.411382
73	1	0	-5.305566	3.332981	0.559204
74	1	0	-5.462049	2.141750	-0.752416

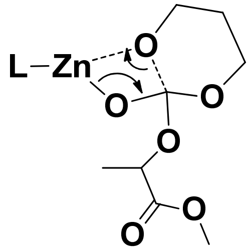

TSII-III^{monomer}- propagation-TMC

Standard orientation:

Center Number	Atomic Number	Atomic Type	Coordinates (Angstroms) X	Y	Z
1	7	0	-0.230574	0.498284	1.760162

2	7	0	-1.288013	1.253828	2.154284
3	6	0	-1.422604	1.167629	3.493970
4	6	0	-0.431658	0.330722	3.996122
5	6	0	0.295869	-0.055325	2.864621
6	5	0	-2.261203	1.995892	1.177864
7	7	0	-1.448747	2.801706	0.103627
8	7	0	-0.461828	2.285636	-0.679814
9	6	0	-0.144992	3.221619	-1.591130
10	6	0	-0.939093	4.358748	-1.413167
11	6	0	-1.748024	4.044510	-0.326368
12	30	0	0.637037	0.550577	-0.199450
13	8	0	2.583342	0.571651	0.459234
14	6	0	3.217070	1.649206	1.116976
15	6	0	3.272262	2.899063	0.224706
16	6	0	4.036896	2.626963	-1.086441
17	8	0	4.236555	1.217217	-1.322489
18	6	0	3.101732	0.463602	-1.353942
19	8	0	3.400045	-0.871350	-1.428052
20	6	0	4.424797	-1.391944	-0.577709
21	6	0	5.023647	-2.609515	-1.280056
22	6	0	-3.385450	1.060836	0.489341
23	6	0	-4.458919	0.395495	1.171017
24	6	0	-5.408108	-0.092031	0.221034
25	6	0	-4.925094	0.244953	-1.078587
26	6	0	-3.689241	0.939154	-0.908031
27	26	0	-3.581309	-0.994627	-0.100622
28	6	0	-3.984071	-3.024157	-0.344783
29	6	0	-3.383093	-2.799023	0.930370
30	6	0	-2.105743	-2.209084	0.721301
31	6	0	-1.900961	-2.070999	-0.695754
32	6	0	-3.076259	-2.579889	-1.348702
33	15	0	-0.389955	-1.406067	-1.432709
34	6	0	0.689108	-2.876710	-1.730448
35	8	0	2.049907	0.843880	-1.923104
36	6	0	3.828896	-1.814416	0.770223
37	8	0	4.683913	-1.498480	1.766403
38	6	0	4.303227	-1.935435	3.079739
39	8	0	2.805679	-2.444029	0.930919
40	6	0	-0.856257	-0.906965	-3.147813
41	1	0	4.237264	1.349092	1.403641
42	1	0	-3.256366	-2.592724	-2.415311
43	1	0	1.163912	-0.694322	2.773389
44	1	0	-0.925039	5.277150	-1.981188
45	1	0	0.643316	3.017565	-2.302673
46	1	0	-2.520941	4.614976	0.167745
47	1	0	-4.535860	0.274465	2.245100

48	1	0	-6.305294	-0.656133	0.443439
49	1	0	-3.840967	-2.992643	1.891551
50	1	0	-1.418743	-1.883584	1.489502
51	1	0	-4.976868	-3.418591	-0.519367
52	1	0	-5.393016	-0.010799	-2.021109
53	1	0	-3.074378	1.328214	-1.709967
54	1	0	-0.258907	0.057752	5.026733
55	1	0	-2.815837	2.834486	1.855182
56	1	0	-2.209494	1.711956	3.995649
57	1	0	0.141352	-3.664775	-2.257776
58	1	0	1.551372	-2.555589	-2.321247
59	1	0	1.063438	-3.250181	-0.774484
60	1	0	-1.240634	-1.753013	-3.727341
61	1	0	-1.613583	-0.119839	-3.114657
62	1	0	0.038933	-0.511832	-3.636923
63	1	0	2.676232	1.882955	2.045350
64	1	0	3.755714	3.721335	0.767617
65	1	0	2.248245	3.218911	0.005822
66	1	0	5.056073	3.018224	-1.045495
67	1	0	3.529215	3.065134	-1.951138
68	1	0	5.188241	-0.633183	-0.405848
69	1	0	5.816725	-3.050030	-0.666517
70	1	0	4.250975	-3.364948	-1.451632
71	1	0	5.448206	-2.309495	-2.242379
72	1	0	5.169253	-1.747605	3.715446
73	1	0	3.443635	-1.359925	3.434618
74	1	0	4.047060	-2.997771	3.075217

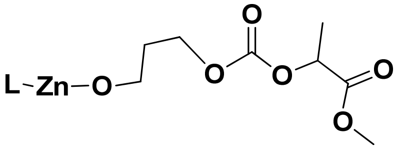

III^{monomer}- propagation-TMC

Standard orientation:

Center Number	Atomic Number	Atomic Type	Coordinates (Angstroms) X	Y	Z
1	7	0	-0.589721	0.973657	1.874738
2	7	0	-1.669669	1.800030	1.859547
3	6	0	-1.974485	2.156980	3.124050
4	6	0	-1.075609	1.553115	3.996421
5	6	0	-0.220403	0.830845	3.158553

6	5	0	-2.488555	2.174198	0.580162
7	7	0	-1.538673	2.666092	-0.567198
8	7	0	-0.436215	2.002177	-1.012965
9	6	0	-0.016702	2.628030	-2.126328
10	6	0	-0.855272	3.708419	-2.419359
11	6	0	-1.802966	3.692495	-1.402209
12	30	0	0.582251	0.549760	0.153664
13	8	0	2.427941	0.726194	0.682051
14	6	0	3.040355	1.943533	0.991516
15	6	0	3.276308	2.838501	-0.244353
16	6	0	4.352673	2.325722	-1.210549
17	8	0	4.519065	0.885989	-1.183126
18	6	0	3.507405	0.137334	-1.626449
19	8	0	3.666612	-1.171184	-1.342883
20	6	0	4.594649	-1.598671	-0.321316
21	6	0	5.070365	-2.993060	-0.713566
22	6	0	-3.506977	1.027872	0.078633
23	6	0	-4.547762	0.430392	0.864671
24	6	0	-5.438275	-0.296463	0.016404
25	6	0	-4.946208	-0.183774	-1.317951
26	6	0	-3.764273	0.615599	-1.271690
27	26	0	-3.548369	-1.114258	-0.102262
28	6	0	-3.807731	-3.181763	-0.053723
29	6	0	-3.351045	-2.720458	1.217837
30	6	0	-2.093896	-2.081962	1.032135
31	6	0	-1.758037	-2.147400	-0.364340
32	6	0	-2.831929	-2.832737	-1.030522
33	15	0	-0.203742	-1.535472	-1.057842
34	6	0	0.859428	-3.039796	-1.191013
35	8	0	2.582323	0.513084	-2.319522
36	6	0	3.873562	-1.645831	1.033272
37	8	0	4.601812	-1.019795	1.972544
38	6	0	4.012054	-0.988214	3.281832
39	8	0	2.853557	-2.263237	1.251327
40	6	0	-0.555146	-1.153544	-2.828210
41	1	0	4.013091	1.747527	1.478004
42	1	0	-2.902492	-3.025633	-2.092793
43	1	0	0.643299	0.227570	3.403721
44	1	0	-0.781778	4.404439	-3.241825
45	1	0	0.865722	2.265123	-2.636301
46	1	0	-2.651974	4.333987	-1.215945
47	1	0	-4.638907	0.510829	1.941347
48	1	0	-6.298922	-0.872057	0.333637
49	1	0	-3.890761	-2.796258	2.152675
50	1	0	-1.506064	-1.592865	1.796254
51	1	0	-4.754072	-3.669216	-0.250358

52	1	0	-5.369634	-0.652855	-2.197413
53	1	0	-3.160384	0.886469	-2.129532
54	1	0	-1.040553	1.638720	5.072368
55	1	0	-3.137181	3.149730	0.893316
56	1	0	-2.806440	2.819748	3.312827
57	1	0	0.314255	-3.846469	-1.692397
58	1	0	1.756251	-2.780415	-1.758857
59	1	0	1.175276	-3.356041	-0.195252
60	1	0	-0.922488	-2.034286	-3.365470
61	1	0	-1.295689	-0.353834	-2.892300
62	1	0	0.377839	-0.811117	-3.285451
63	1	0	2.449293	2.537075	1.715226
64	1	0	3.584218	3.843138	0.079681
65	1	0	2.323521	2.956852	-0.768127
66	1	0	5.344048	2.678941	-0.917631
67	1	0	4.158058	2.636606	-2.241524
68	1	0	5.425130	-0.898498	-0.262566
69	1	0	5.794252	-3.362361	0.020948
70	1	0	4.225583	-3.686435	-0.749651
71	1	0	5.552634	-2.962103	-1.695082
72	1	0	4.746131	-0.499846	3.923510
73	1	0	3.086339	-0.409858	3.240498
74	1	0	3.800346	-2.000652	3.635691

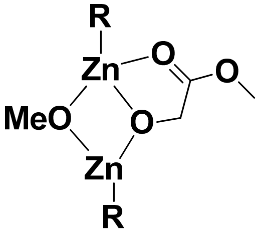

Cat^{dimer}- propagation

Standard orientation:

Center Number	Atomic Number	Atomic Type	Coordinates (Angstroms) X	Y	Z
1	26	0	6.309591	-0.904268	0.681297
2	6	0	4.645782	0.363386	0.305860
3	6	0	4.261714	-1.019746	0.295680
4	6	0	4.571502	-1.615816	1.557874
5	1	0	5.541057	-0.736304	3.381123
6	6	0	5.211965	0.598093	1.603967
7	6	0	5.163384	-0.604975	2.374828

8	5	0	4.506686	1.419599	-0.906032
9	7	0	4.067895	2.831356	-0.371670
10	7	0	2.859769	3.085920	0.200881
11	7	0	3.457761	0.907050	-1.952256
12	1	0	2.327795	1.589211	2.900450
13	30	0	1.217889	1.771532	-0.056292
14	8	0	-0.740853	1.012110	-0.507774
15	8	0	0.765074	0.937571	1.708399
16	6	0	1.593852	0.775031	2.842485
17	30	0	-1.005086	0.163460	1.278665
18	7	0	-2.444392	0.765803	2.658096
19	7	0	-3.469035	-0.079598	2.951810
20	7	0	-1.390513	-1.865893	1.487948
21	7	0	-2.613434	-2.262824	1.939558
22	5	0	-3.847038	-1.290356	2.021374
23	6	0	-5.162945	0.553871	-1.160320
24	6	0	-5.035203	0.411547	0.256708
25	6	0	-4.245568	-0.752046	0.554515
26	6	0	-3.909760	-1.328617	-0.718432
27	6	0	-4.466044	-0.534307	-1.767879
28	6	0	-8.017237	-1.392940	-1.105112
29	6	0	-7.857881	-1.514039	0.317183
30	6	0	-7.023865	-2.637633	0.593796
31	6	0	-6.649287	-3.222575	-0.651520
32	1	0	-7.138438	-2.639462	-2.754189
33	6	0	-7.253396	-2.456914	-1.693598
34	15	0	-9.214781	-0.309683	-1.972598
35	6	0	7.788224	-0.482787	-0.707547
36	6	0	7.395325	-1.860126	-0.826094
37	6	0	7.652488	-2.475350	0.447798
38	6	0	8.198968	-1.492973	1.325245
39	6	0	8.287021	-0.260750	0.608417
40	15	0	6.744847	-2.582383	-2.378344
41	6	0	2.889165	4.356076	0.634999
42	6	0	4.129950	4.938337	0.359519
43	6	0	4.841981	3.930900	-0.283538
44	6	0	-0.598440	-2.950763	1.473957
45	6	0	-1.309000	-4.073074	1.908928
46	6	0	-2.580415	-3.584688	2.194727
47	8	0	-0.027277	3.594906	-0.557365
48	6	0	-1.625787	1.873007	-1.138409
49	6	0	-1.118049	3.304669	-1.046394
50	8	0	-1.947600	4.206985	-1.555258
51	6	0	-1.507339	5.581750	-1.535515
52	6	0	5.796904	-4.059117	-1.743077
53	6	0	8.277520	-3.472973	-2.985792

54	7	0	2.128943	0.808761	-1.675948
55	6	0	1.552037	0.175250	-2.710819
56	6	0	2.513390	-0.156077	-3.670159
57	6	0	3.707695	0.327658	-3.142662
58	6	0	-2.422557	1.712331	3.611052
59	6	0	-3.453998	1.496814	4.529564
60	6	0	-4.087552	0.345537	4.069614
61	26	0	-5.982337	-1.261035	-0.554211
62	6	0	-9.010888	1.293371	-1.040211
63	6	0	-8.280777	0.102425	-3.534196
64	1	0	8.458955	-1.644276	2.365274
65	1	0	5.634756	1.537387	1.939937
66	1	0	-4.395835	-0.737636	-2.828868
67	1	0	-5.987846	-4.068446	-0.787003
68	1	0	-8.214321	-0.786374	-4.170089
69	1	0	-7.963746	1.601743	-0.959432
70	1	0	-3.702568	2.083199	5.401543
71	1	0	8.027671	-4.062114	-3.875824
72	1	0	-3.343471	-2.240748	-0.859590
73	1	0	4.907413	-3.720493	-1.203914
74	1	0	6.390894	-4.698538	-1.079546
75	1	0	4.461419	5.941195	0.584748
76	1	0	8.626242	0.685761	1.009069
77	1	0	7.668315	0.268790	-1.476876
78	1	0	7.439745	-3.503038	0.710971
79	1	0	4.418827	-2.651675	1.834104
80	1	0	3.822559	-1.530423	-0.552297
81	1	0	2.369717	-0.671138	-4.608356
82	1	0	5.530959	1.612935	-1.516877
83	1	0	9.035573	-2.736443	-3.271787
84	1	0	8.704613	-4.138476	-2.226402
85	1	0	5.469599	-4.659760	-2.599022
86	1	0	0.486156	-0.006031	-2.692035
87	1	0	4.718160	0.277158	-3.520998
88	1	0	2.013874	4.785070	1.103531
89	1	0	5.844662	3.918596	-0.685357
90	1	0	-2.643987	1.846517	-0.721768
91	1	0	-1.664621	2.484124	3.586995
92	1	0	-4.935762	-0.198924	4.458649
93	1	0	0.432913	-2.864109	1.158112
94	1	0	-0.954041	-5.088067	2.007091
95	1	0	-3.463985	-4.086704	2.561389
96	1	0	-4.714536	-1.894593	2.605144
97	1	0	-5.478214	1.069800	0.994396
98	1	0	-5.702498	1.336039	-1.678483
99	1	0	-6.681957	-2.946184	1.572510

100	1	0	-8.286064	-0.852399	1.058870
101	1	0	-8.841995	0.862409	-4.089155
102	1	0	-7.269365	0.470408	-3.333935
103	1	0	-9.583280	2.073937	-1.553694
104	1	0	-9.427033	1.189405	-0.032889
105	1	0	0.994872	0.789784	3.765867
106	1	0	2.152301	-0.170305	2.807929
107	1	0	-1.738021	1.644746	-2.213333
108	1	0	-2.305753	6.145616	-2.016704
109	1	0	-1.364268	5.917730	-0.505798
110	1	0	-0.570376	5.686764	-2.087103

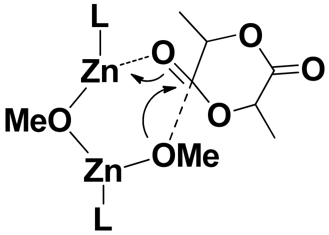

TSI-II^{dimer}- propagation-LA

Standard orientation:

Center Number	Atomic Number	Atomic Type	Coordinates (Angstroms) X	Y	Z
1	6	0	-7.991529	-1.523847	0.591329
2	6	0	-8.513708	-1.303121	-0.715488
3	6	0	-8.235489	-2.463036	-1.500679
4	6	0	-7.548185	-3.400668	-0.674440
5	6	0	-7.391580	-2.829365	0.635584
6	26	0	-6.459315	-1.635295	-0.800265
7	6	0	-5.590292	0.033453	-1.680377
8	6	0	-5.023653	-0.130100	-0.370820
9	6	0	-4.429609	-1.436999	-0.366913
10	6	0	-4.613902	-2.054396	-1.642661
11	6	0	-5.336574	-1.136036	-2.463177
12	5	0	-5.065376	0.929625	0.844412
13	7	0	-4.768366	2.378569	0.300671
14	6	0	-5.673628	3.358901	0.115225
15	6	0	-5.088949	4.388489	-0.615960
16	6	0	-3.783410	3.946078	-0.845848
17	7	0	-3.596091	2.734398	-0.294757
18	30	0	-1.835096	1.690206	0.162837

19	7	0	-2.671258	0.558331	1.720374
20	7	0	-4.014803	0.539562	1.939920
21	6	0	-4.264739	-0.066047	3.117847
22	6	0	-3.058497	-0.450680	3.694660
23	6	0	-2.090085	-0.032047	2.777510
24	15	0	-6.633695	-3.525246	2.150560
25	6	0	-8.006116	-4.673413	2.704775
26	8	0	-0.513777	2.951828	1.059601
27	6	0	-0.481889	4.307280	0.742493
28	6	0	-0.112788	4.560410	-0.718058
29	8	0	0.576330	5.706293	-0.872266
30	6	0	0.882491	6.088805	-2.226794
31	8	0	-1.051786	0.600521	-1.295801
32	6	0	-1.678032	0.537664	-2.571795
33	8	0	-0.435089	3.836909	-1.642698
34	30	0	0.847862	0.174185	-1.029978
35	8	0	0.786145	0.805586	0.915818
36	6	0	1.160976	1.932426	1.343366
37	6	0	1.223356	2.121258	2.851926
38	8	0	1.572598	3.467936	3.235497
39	6	0	2.466788	4.174434	2.519007
40	6	0	2.832956	3.706028	1.112069
41	8	0	2.045050	2.606201	0.584929
42	6	0	2.191344	1.131136	3.503630
43	8	0	2.938352	5.196854	2.954166
44	6	0	4.313665	3.349047	0.996664
45	7	0	2.157732	0.854228	-2.472618
46	7	0	3.110009	0.022417	-2.976963
47	6	0	3.610593	0.562125	-4.104218
48	6	0	2.970567	1.773136	-4.354168
49	6	0	2.059265	1.906790	-3.303042
50	5	0	3.590944	-1.293631	-2.249492
51	6	0	4.313829	-0.947743	-0.854288
52	6	0	3.934757	-1.339137	0.475925
53	6	0	4.876265	-0.816174	1.416191
54	6	0	5.858909	-0.087020	0.679676
55	6	0	5.518558	-0.180062	-0.704548
56	26	0	5.836696	-2.081706	0.085813
57	6	0	7.416698	-3.010531	-0.905474
58	6	0	6.199193	-3.716628	-1.137342
59	6	0	5.697210	-4.151819	0.124760
60	6	0	6.607994	-3.713133	1.131746
61	6	0	7.689463	-3.006345	0.504804
62	15	0	9.257154	-2.505056	1.310523
63	6	0	8.635499	-1.745940	2.897527
64	7	0	1.234569	-1.839255	-1.353434

65	7	0	2.349490	-2.229876	-2.033343
66	6	0	2.220080	-3.523801	-2.382316
67	6	0	0.993320	-3.999500	-1.928254
68	6	0	0.409460	-2.898831	-1.295982
69	6	0	9.696370	-0.962543	0.357202
70	6	0	-5.466532	-4.795756	1.439013
71	1	0	-5.666507	-1.309438	-3.479778
72	1	0	-1.528064	1.468692	-3.136865
73	1	0	6.492351	-3.873844	2.195936
74	1	0	-8.466053	-2.590902	-2.550723
75	1	0	-6.146514	0.900645	-2.015464
76	1	0	4.868399	-0.978923	2.486535
77	1	0	4.767552	-4.679070	0.295291
78	1	0	8.234306	-2.530111	3.547826
79	1	0	8.868392	-0.248473	0.297535
80	1	0	3.136369	2.446584	-5.181960
81	1	0	-7.661580	-5.264786	3.561124
82	1	0	3.090633	-1.970108	0.723584
83	1	0	-4.656332	-4.287575	0.908073
84	1	0	-5.955569	-5.495120	0.750771
85	1	0	-5.543926	5.315739	-0.931166
86	1	0	-8.992738	-0.397864	-1.065226
87	1	0	-8.004162	-0.814339	1.408671
88	1	0	-7.175819	-4.364584	-0.995060
89	1	0	-4.293911	-3.049095	-1.926478
90	1	0	-3.939642	-1.890306	0.485619
91	1	0	-2.911543	-0.959504	4.635630
92	1	0	-6.132208	1.009262	1.405837
93	1	0	-8.865441	-4.078588	3.031389
94	1	0	-8.333405	-5.354374	1.910368
95	1	0	-5.028586	-5.371281	2.262049
96	1	0	-1.013533	-0.127746	2.808747
97	1	0	-5.282046	-0.201978	3.453684
98	1	0	-2.972633	4.419963	-1.381330
99	1	0	-6.674021	3.250375	0.508198
100	1	0	0.199628	4.871840	1.389625
101	1	0	1.332013	2.682365	-3.099629
102	1	0	4.387806	0.047716	-4.650503
103	1	0	-0.556553	-2.804315	-0.817832
104	1	0	0.584635	-4.991607	-2.048963
105	1	0	3.009338	-4.017726	-2.930108
106	1	0	4.310212	-1.847627	-3.046263
107	1	0	6.094729	0.242340	-1.518990
108	1	0	6.721039	0.419535	1.093284
109	1	0	5.718562	-3.847326	-2.097574
110	1	0	8.024981	-2.541766	-1.667943

111	1	0	9.479990	-1.284594	3.421417
112	1	0	7.857062	-0.995247	2.728222
113	1	0	10.554104	-0.484240	0.843006
114	1	0	10.003435	-1.232628	-0.658467
115	1	0	-1.264111	-0.293054	-3.160477
116	1	0	-2.754033	0.363474	-2.461057
117	1	0	-1.476783	4.763710	0.887951
118	1	0	1.344124	7.073412	-2.154014
119	1	0	1.576743	5.373867	-2.676346
120	1	0	-0.029200	6.131744	-2.827642
121	1	0	2.613752	4.557486	0.461676
122	1	0	0.215529	1.977800	3.237072
123	1	0	4.913906	4.206338	1.313005
124	1	0	4.567128	2.483787	1.615539
125	1	0	4.550296	3.100812	-0.041211
126	1	0	2.223646	1.320777	4.580406
127	1	0	1.851816	0.107083	3.328635
128	1	0	3.203735	1.225535	3.098872

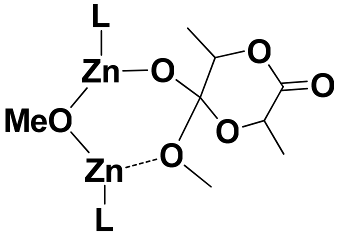

II^{dimer}- propagation-LA

Standard orientation:

Center Number	Atomic Number	Atomic Type	Coordinates (Angstroms) X	Y	Z
1	26	0	-6.439149	-1.675951	-0.866812
2	6	0	-5.008215	-0.181038	-0.398539
3	6	0	-5.619676	0.051992	-1.677048
4	15	0	-6.530553	-3.685633	2.018083
5	6	0	-4.619416	-2.021251	-1.795599
6	6	0	-4.398129	-1.478037	-0.492258
7	6	0	-7.320822	-2.939754	0.543559
8	6	0	-7.496963	-3.463642	-0.783484
9	6	0	-8.216665	-2.507027	-1.558655
10	6	0	-8.495020	-1.381882	-0.724309
11	6	0	-7.940597	-1.642871	0.561712
12	6	0	6.043872	-0.187243	0.719348

13	6	0	4.950943	-0.829157	1.377391
14	6	0	4.441171	-0.709882	-0.906749
15	6	0	6.064203	-3.601733	-1.447484
16	6	0	6.141756	-3.970370	0.826802
17	1	0	8.095328	-2.676089	-1.565600
18	6	0	7.324354	-3.140327	-0.964588
19	15	0	8.881976	-3.240097	1.509932
20	30	0	0.875609	0.497855	-1.123789
21	7	0	2.306266	1.324653	-2.357255
22	7	0	3.289775	0.559337	-2.905336
23	7	0	1.344908	-1.476786	-1.591527
24	6	0	2.353704	-3.028104	-2.794047
25	7	0	2.476414	-1.787588	-2.284670
26	5	0	3.732515	-0.850708	-2.343482
27	6	0	0.515927	-2.530671	-1.688097
28	6	0	1.114203	-3.549178	-2.434466
29	6	0	3.280397	2.502850	-4.003693
30	8	0	-1.052569	0.735127	-1.523651
31	30	0	-1.784444	1.439238	0.170257
32	7	0	-3.491636	2.628599	-0.141893
33	7	0	-4.680411	2.276442	0.420151
34	6	0	-5.550845	3.296464	0.290644
35	6	0	-4.924653	4.348995	-0.370177
36	6	0	-3.632412	3.877198	-0.619282
37	8	0	0.344856	1.012218	0.775421
38	6	0	0.704112	2.167765	1.340299
39	6	0	1.013684	2.014423	2.839901
40	8	0	1.533968	3.247952	3.400624
41	6	0	2.525354	3.896659	2.752518
42	6	0	2.719809	3.637990	1.257817
43	8	0	1.747639	2.773108	0.627210
44	8	0	-0.542376	3.010827	1.216058
45	6	0	-0.448175	4.414086	1.079013
46	6	0	-0.036343	4.839729	-0.328066
47	8	0	-0.308268	4.236680	-1.345724
48	6	0	-5.379165	-1.065589	-2.535491
49	6	0	5.733416	-0.126927	-0.673625
50	6	0	3.974719	-1.145917	0.380997
51	6	0	5.330148	-4.115336	-0.337750
52	6	0	7.391141	-3.371272	0.450820
53	6	0	2.287247	2.494133	-3.020882
54	6	0	3.886322	1.254367	-3.892001
55	5	0	-5.022993	0.806989	0.875670
56	7	0	-3.980136	0.329897	1.942635
57	7	0	-2.638918	0.316655	1.707174
58	6	0	-2.062338	-0.324750	2.738032

59	6	0	-3.032437	-0.744177	3.651857
60	6	0	-4.233800	-0.306457	3.102198
61	26	0	5.766575	-2.108499	-0.037274
62	6	0	9.710487	-1.723133	0.808698
63	6	0	8.170328	-2.525396	3.079751
64	6	0	1.955599	0.859532	3.160489
65	6	0	4.126601	3.134816	0.936304
66	8	0	3.188483	4.730906	3.319897
67	6	0	-1.703016	0.681382	-2.780969
68	6	0	-7.881740	-4.871529	2.543951
69	6	0	-5.359209	-4.914409	1.243114
70	8	0	0.610834	6.016494	-0.292998
71	6	0	0.954706	6.590885	-1.570023
72	1	0	4.250114	3.074310	-0.148170
73	1	0	-5.741502	-1.183417	-3.548964
74	1	0	-1.588431	1.625895	-3.333279
75	1	0	5.855284	-4.254325	1.831423
76	1	0	-8.468989	-2.599175	-2.607442
77	1	0	-6.197510	0.929262	-1.942402
78	1	0	4.891079	-1.070135	2.431199
79	1	0	4.318566	-4.498917	-0.364941
80	1	0	7.535339	-3.271001	3.569235
81	1	0	9.029031	-0.870920	0.718756
82	1	0	3.519390	3.290479	-4.702818
83	1	0	-7.519037	-5.490802	3.372566
84	1	0	3.050579	-1.680074	0.561204
85	1	0	-4.553938	-4.379794	0.730957
86	1	0	-5.847633	-5.586723	0.527957
87	1	0	-5.346062	5.308445	-0.631256
88	1	0	-8.995718	-0.472067	-1.028922
89	1	0	-7.944793	-0.964151	1.405047
90	1	0	-7.117374	-4.409239	-1.147325
91	1	0	-4.297364	-2.992378	-2.149750
92	1	0	-3.875077	-1.975347	0.314865
93	1	0	-2.889973	-1.288334	4.573478
94	1	0	-6.087295	0.883952	1.441681
95	1	0	-8.744685	-4.301373	2.903344
96	1	0	-8.209668	-5.525666	1.727603
97	1	0	-4.913477	-5.522369	2.038228
98	1	0	-0.988875	-0.448857	2.755346
99	1	0	-5.250591	-0.423609	3.445955
100	1	0	-2.800852	4.353318	-1.120121
101	1	0	-6.557646	3.198035	0.669771
102	1	0	0.210851	4.858040	1.827750
103	1	0	1.553049	3.245351	-2.760247
104	1	0	4.702119	0.813195	-4.445912

105	1	0	-0.464106	-2.488722	-1.231714
106	1	0	0.706312	-4.516314	-2.687934
107	1	0	3.155861	-3.455478	-3.377671
108	1	0	4.467002	-1.304423	-3.188980
109	1	0	6.385965	0.270404	-1.441823
110	1	0	6.954113	0.163557	1.187641
111	1	0	5.711017	-3.519093	-2.466437
112	1	0	8.993485	-2.293486	3.764805
113	1	0	7.579674	-1.621358	2.900788
114	1	0	10.546979	-1.450783	1.461750
115	1	0	10.125612	-1.950815	-0.178460
116	1	0	-1.281260	-0.127005	-3.395287
117	1	0	-2.773614	0.481647	-2.654935
118	1	0	-1.456491	4.805713	1.257183
119	1	0	1.378045	7.568871	-1.343116
120	1	0	1.690258	5.964079	-2.080376
121	1	0	0.064545	6.687375	-2.196055
122	1	0	2.593897	4.626428	0.800271
123	1	0	0.061290	1.873211	3.352964
124	1	0	4.860807	3.834003	1.346092
125	1	0	4.308878	2.139894	1.350270
126	1	0	2.173516	0.862401	4.232898
127	1	0	1.487948	-0.090685	2.893256
128	1	0	2.897275	0.930338	2.609421

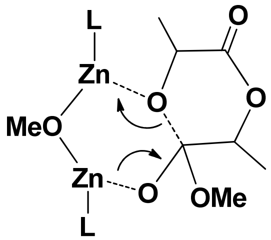

TSII-III^{dimer}- propagation-LA

Standard orientation:

Center Number	Atomic Number	Atomic Type	Coordinates (Angstroms) X	Y	Z
1	6	0	-2.360794	2.856715	-1.985050
2	7	0	-2.859980	2.103044	-0.988714
3	7	0	-4.219529	2.149374	-1.088916
4	6	0	-4.557753	2.910449	-2.146596
5	6	0	-3.400151	3.383162	-2.756465
6	30	0	-1.990118	1.431643	0.775962
7	8	0	-1.034962	-0.237068	1.225293

8	30	0	0.957233	-0.381710	1.188210
9	7	0	1.736908	-1.859502	2.448755
10	7	0	2.531560	-2.838594	1.934496
11	6	0	2.685109	-3.811071	2.853659
12	6	0	1.973900	-3.472574	4.000941
13	6	0	1.391428	-2.241137	3.691199
14	5	0	3.245733	-2.738063	0.531369
15	6	0	4.309555	-1.528664	0.517557
16	6	0	4.576855	-0.607933	-0.553229
17	6	0	5.605949	0.302989	-0.156891
18	6	0	6.008417	-0.048630	1.165985
19	6	0	5.218700	-1.169321	1.572044
20	26	0	6.328180	-1.649618	-0.117847
21	6	0	8.289494	-2.318941	0.093721
22	6	0	8.104505	-1.835760	-1.245546
23	6	0	7.045834	-2.617513	-1.820086
24	6	0	6.599556	-3.568505	-0.854615
25	6	0	7.369838	-3.384390	0.330767
26	15	0	9.216124	-0.682101	-2.136195
27	6	0	9.472777	0.656262	-0.861639
28	5	0	-5.180206	1.368598	-0.127563
29	6	0	-4.980040	-0.223432	-0.275638
30	6	0	-4.311598	-0.938503	-1.327680
31	6	0	-4.397565	-2.344587	-1.083191
32	6	0	-5.128187	-2.526797	0.129634
33	6	0	-5.487736	-1.230795	0.614517
34	26	0	-6.298348	-1.551661	-1.274376
35	6	0	-7.883755	-0.459504	-2.036022
36	6	0	-8.378189	-1.483086	-1.157082
37	6	0	-7.946002	-2.740920	-1.703751
38	6	0	-7.206183	-2.489351	-2.896814
39	6	0	-7.171293	-1.077238	-3.104931
40	15	0	-9.368877	-1.125662	0.342833
41	6	0	-9.145210	-2.701769	1.317101
42	7	0	-3.675540	1.747424	1.945786
43	6	0	-3.828268	2.114253	3.229901
44	6	0	-5.165394	2.423943	3.488514
45	6	0	-5.805356	2.227130	2.267650
46	7	0	-4.900335	1.826659	1.355185
47	8	0	-0.500189	2.769526	1.176425
48	6	0	0.615037	2.779773	0.609339
49	6	0	1.520025	3.998380	0.839139
50	6	0	1.151922	4.773018	2.100115
51	8	0	0.627335	2.131853	-0.602388
52	6	0	1.809471	2.015023	-1.391328
53	6	0	1.986486	3.206614	-2.321415

54	8	0	3.088354	3.038067	-3.065686
55	6	0	3.385529	4.098752	-3.994969
56	8	0	1.238485	4.157991	-2.396870
57	6	0	-11.090510	-1.445383	-0.322796
58	6	0	-1.763526	-1.456359	1.375962
59	8	0	1.697159	1.424732	1.536356
60	6	0	2.878826	1.709936	2.247850
61	6	0	3.572702	2.972039	1.726930
62	8	0	4.703196	3.258646	2.034220
63	7	0	1.290225	-1.480427	-0.571754
64	7	0	2.154991	-2.530876	-0.578033
65	6	0	1.942899	-3.274214	-1.681246
66	6	0	0.911001	-2.704995	-2.421455
67	6	0	0.528321	-1.587017	-1.674310
68	6	0	2.676876	1.760961	3.770069
69	6	0	8.022241	0.179919	-3.281868
70	8	0	2.933858	3.732069	0.776791
71	1	0	5.310322	-1.686375	2.519763
72	1	0	-9.404418	-3.601795	0.747113
73	1	0	-5.388408	-3.475711	0.581331
74	1	0	-3.324246	4.023087	-3.622862
75	1	0	1.351747	4.643100	-0.029256
76	1	0	2.371300	0.771220	4.124386
77	1	0	-0.259108	-0.867886	-1.858755
78	1	0	3.292475	-4.675584	2.628421
79	1	0	1.889607	-4.037849	4.917013
80	1	0	-8.106783	-2.782549	1.651972
81	1	0	-5.599212	3.063367	-2.389416
82	1	0	-1.292986	3.000513	-2.076477
83	1	0	-3.846961	-0.481376	-2.192645
84	1	0	-4.010450	-3.129793	-1.720239
85	1	0	-6.653970	-0.564714	-3.905581
86	1	0	-7.995137	0.604750	-1.874984
87	1	0	-8.117355	-3.715343	-1.265966
88	1	0	-6.718720	-3.235546	-3.511192
89	1	0	-6.072868	-1.033589	1.504470
90	1	0	-5.605768	2.747798	4.419667
91	1	0	-6.295411	1.757153	-0.376236
92	1	0	-11.814418	-1.382624	0.497945
93	1	0	-11.345298	-0.672481	-1.055289
94	1	0	-11.180061	-2.426990	-0.802748
95	1	0	-9.786990	-2.658952	2.203942
96	1	0	-2.971392	2.143075	3.890182
97	1	0	-6.842880	2.336543	1.986864
98	1	0	-2.812635	-1.253878	1.616204
99	1	0	-1.737870	-2.053345	0.455185

100	1	0	-1.336825	-2.058789	2.189699
101	1	0	0.496231	-3.056171	-3.354552
102	1	0	2.539791	-4.156194	-1.862333
103	1	0	0.740890	-1.615911	4.288961
104	1	0	3.716621	-3.833509	0.338378
105	1	0	4.094353	-0.624791	-1.522687
106	1	0	6.015394	1.106312	-0.754238
107	1	0	6.781121	0.436041	1.747780
108	1	0	8.997701	-1.928491	0.813273
109	1	0	5.779893	-4.263220	-0.978023
110	1	0	7.246592	-3.921477	1.262159
111	1	0	6.639966	-2.494371	-2.815863
112	1	0	10.034825	1.476220	-1.322489
113	1	0	8.531972	1.042799	-0.457053
114	1	0	10.078001	0.267131	-0.036374
115	1	0	8.545699	1.009812	-3.769552
116	1	0	7.699150	-0.511607	-4.066883
117	1	0	7.138534	0.563232	-2.761806
118	1	0	1.844542	5.612792	2.208939
119	1	0	0.133211	5.158736	2.020188
120	1	0	1.205561	4.148398	2.994604
121	1	0	3.611943	2.036134	4.267725
122	1	0	1.899060	2.478037	4.050527
123	1	0	3.621993	0.925506	2.047105
124	1	0	1.678814	1.112137	-1.993815
125	1	0	2.693751	1.875222	-0.770561
126	1	0	4.296799	3.789283	-4.505880
127	1	0	2.565187	4.222329	-4.706282
128	1	0	3.543232	5.037435	-3.458431

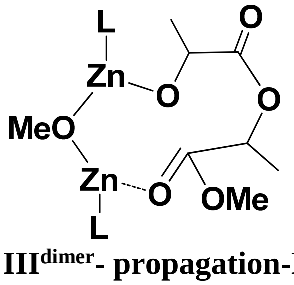

III^{dimer}- propagation-LA

Standard orientation:

Center Number	Atomic Number	Atomic Type	Coordinates (Angstroms) X	Y	Z

1	26	0	-6.369700	-1.171401	-1.398475
2	6	0	-5.024627	-0.186561	-0.090394
3	6	0	-5.615261	-1.337686	0.534633
4	6	0	-4.355108	-0.683608	-1.261227
5	6	0	-4.523148	-2.100729	-1.346154
6	6	0	-5.306183	-2.509257	-0.224646
7	6	0	-7.903297	0.136160	-1.872076
8	6	0	-8.451307	-1.077179	-1.331380
9	6	0	-8.023026	-2.144485	-2.194447
10	6	0	-7.233126	-1.591622	-3.245455
11	6	0	-7.163069	-0.179167	-3.048760
12	7	0	-4.697776	1.467105	1.924370
13	7	0	-4.258180	2.292390	-0.453515
14	7	0	-2.897785	2.205869	-0.507221
15	30	0	-1.854474	1.196044	0.979509
16	8	0	-0.996006	-0.606206	0.964393
17	30	0	0.964338	-0.707591	0.855123
18	7	0	1.779344	-2.110777	2.169853
19	6	0	1.336847	-2.563159	3.357384
20	6	0	1.965885	-3.766474	3.683319
21	6	0	2.807056	-4.015553	2.602462
22	7	0	2.681217	-3.019779	1.705225
23	7	0	1.548475	-1.708707	-0.875276
24	6	0	1.466857	-2.904426	-2.779478
25	7	0	2.513088	-2.669253	-0.822124
26	5	0	3.514020	-2.814305	0.380756
27	6	0	4.460753	-1.520883	0.511142
28	6	0	5.334053	-1.219127	1.613690
29	6	0	6.005410	0.018907	1.370868
30	6	0	5.562844	0.505791	0.105353
31	6	0	4.628558	-0.440366	-0.421524
32	26	0	6.474290	-1.353164	-0.120049
33	6	0	8.138491	-1.226861	-1.416251
34	6	0	7.828551	-2.891331	0.183544
35	6	0	8.530847	-1.684357	-0.113025
36	6	0	6.987118	-3.191392	-0.927362
37	15	0	8.942513	0.105439	-2.385293
38	8	0	-0.264318	2.263124	1.547259
39	8	0	1.790157	1.136052	1.390485
40	6	0	2.692017	1.405405	2.479462
41	6	0	3.529833	2.642355	2.161495
42	8	0	4.584389	2.858005	2.705971
43	6	0	2.013655	1.460096	3.850303
44	8	0	3.078916	3.447695	1.168070
45	6	0	1.646847	3.606931	0.976581
46	6	0	1.098140	4.589103	2.005786

47	6	0	0.860993	2.255601	0.909078
48	8	0	0.647355	1.796673	-0.447355
49	6	0	1.725554	1.835668	-1.372039
50	6	0	1.565873	2.961447	-2.386584
51	8	0	2.460513	2.804828	-3.376823
52	6	0	2.453673	3.823529	-4.395242
53	6	0	7.172276	-2.169462	-1.906100
54	6	0	0.906008	-1.854607	-2.048766
55	6	0	2.478868	-3.391884	-1.957327
56	6	0	-4.671941	3.250107	-1.304813
57	6	0	-3.563965	3.812076	-1.930595
58	6	0	-2.471701	3.128162	-1.388020
59	6	0	-5.489785	1.697378	2.988412
60	6	0	-4.722538	1.639666	4.148641
61	6	0	-3.431345	1.364166	3.692179
62	7	0	-3.423560	1.252729	2.352846
63	6	0	9.214678	1.404164	-1.074198
64	6	0	7.485444	0.846684	-3.284937
65	6	0	-1.742607	-1.819931	1.025279
66	5	0	-5.143624	1.338372	0.416310
67	15	0	-9.488758	-1.124296	0.178641
68	6	0	-11.189194	-1.238065	-0.599640
69	8	0	0.770321	3.874193	-2.322020
70	6	0	-9.297053	-2.905737	0.701218
71	1	0	5.483283	-1.849908	2.482056
72	1	0	-9.542291	-3.618404	-0.095113
73	1	0	-5.635972	-3.518065	-0.010161
74	1	0	-3.551105	4.606559	-2.661853
75	1	0	1.569043	4.072146	-0.006334
76	1	0	1.591118	0.479149	4.088895
77	1	0	0.074079	-1.207469	-2.294419
78	1	0	3.488781	-4.831659	2.411927
79	1	0	1.829050	-4.371300	4.567196
80	1	0	-8.267877	-3.081568	1.027633
81	1	0	-5.726143	3.464361	-1.404221
82	1	0	-1.413578	3.261320	-1.569723
83	1	0	-3.833888	-0.070601	-1.985848
84	1	0	-4.155061	-2.743607	-2.135925
85	1	0	-6.603323	0.520983	-3.655196
86	1	0	-8.000945	1.115838	-1.422999
87	1	0	-8.231547	-3.196673	-2.053718
88	1	0	-6.737253	-2.150697	-4.028776
89	1	0	-6.226610	-1.314488	1.428784
90	1	0	-5.053160	1.783458	5.166477
91	1	0	-6.265779	1.781696	0.380221
92	1	0	-11.937927	-1.395194	0.185418

93	1	0	-11.422063	-0.293297	-1.101677
94	1	0	-11.263438	-2.051611	-1.330829
95	1	0	-9.961967	-3.096124	1.550883
96	1	0	-2.510768	1.251046	4.249624
97	1	0	-6.544591	1.882765	2.846209
98	1	0	-2.784314	-1.614264	1.291042
99	1	0	-1.738330	-2.338849	0.057642
100	1	0	-1.322867	-2.498206	1.781974
101	1	0	1.178169	-3.264740	-3.755490
102	1	0	3.176545	-4.203815	-2.101969
103	1	0	0.587651	-2.006482	3.905079
104	1	0	4.097651	-3.857345	0.208768
105	1	0	4.144144	-0.371224	-1.387254
106	1	0	5.887179	1.421427	-0.370312
107	1	0	6.726587	0.496520	2.020384
108	1	0	9.231117	-1.186770	0.545250
109	1	0	6.284064	-4.011411	-0.988464
110	1	0	7.889532	-3.450789	1.107946
111	1	0	6.656329	-2.107857	-2.855584
112	1	0	9.562112	2.323849	-1.557824
113	1	0	8.309657	1.616625	-0.496132
114	1	0	10.003008	1.076425	-0.388684
115	1	0	7.820793	1.747840	-3.810437
116	1	0	7.121701	0.141871	-4.039833
117	1	0	6.659876	1.102848	-2.613477
118	1	0	1.690320	5.508361	1.964105
119	1	0	0.055810	4.821160	1.778291
120	1	0	1.140383	4.192464	3.023535
121	1	0	2.761367	1.708672	4.609895
122	1	0	1.208983	2.197394	3.878305
123	1	0	3.403341	0.575432	2.465710
124	1	0	1.751384	0.883520	-1.909852
125	1	0	2.692106	1.941531	-0.868844
126	1	0	3.230974	3.533084	-5.101646
127	1	0	1.478702	3.862605	-4.887263
128	1	0	2.674972	4.800214	-3.957586

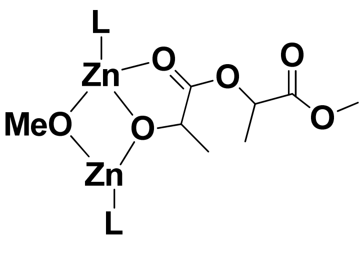

IV^{dimer}- propagation-LA

Standard orientation:

Center Number	Atomic Number	Atomic Type	Coordinates (Angstroms) X	Y	Z
1	6	0	4.484444	-0.702227	-3.019145
2	7	0	4.022138	0.131801	-2.067165
3	7	0	2.705769	-0.131978	-1.839447
4	6	0	2.352673	-1.131344	-2.665819
5	6	0	3.447876	-1.532544	-3.435835
6	5	0	4.874875	1.094396	-1.169505
7	6	0	5.123214	0.435216	0.283186
8	6	0	5.584130	1.099010	1.469606
9	6	0	5.702202	0.153646	2.535765
10	6	0	5.325048	-1.124168	2.021233
11	6	0	4.977211	-0.946030	0.645628
12	26	0	6.957970	-0.382692	0.985725
13	6	0	8.884030	-0.461362	1.771681
14	6	0	8.547515	-1.712026	1.174792
15	6	0	8.258735	-1.493415	-0.216438
16	6	0	8.417595	-0.085178	-0.455449
17	6	0	8.808031	0.544924	0.761232
18	15	0	7.808136	-2.684002	-1.532746
19	6	0	7.070755	-4.083299	-0.542681
20	30	0	1.505941	1.025833	-0.563418
21	7	0	2.866170	2.642539	-0.621772
22	7	0	4.149295	2.482333	-1.048006
23	6	0	4.711336	3.693781	-1.228413
24	6	0	3.780089	4.678845	-0.916399
25	6	0	2.636141	3.964678	-0.549317
26	30	0	-0.554811	-0.602414	0.964757
27	8	0	-0.305671	-0.086310	-0.948562
28	6	0	-1.279553	0.520121	-1.744521
29	6	0	-1.236939	0.017111	-3.199152
30	7	0	-0.842399	-2.577271	1.542429
31	7	0	-2.022367	-2.936871	2.123066
32	6	0	-1.909479	-4.187234	2.610610
33	6	0	-0.628647	-4.665050	2.351111
34	6	0	0.002737	-3.612204	1.683193
35	5	0	-3.305419	-2.027720	2.089737
36	7	0	-2.956970	-0.653153	2.775663
37	7	0	-1.974171	0.154396	2.300012
38	6	0	-1.925488	1.236564	3.093251
39	6	0	-2.906214	1.148267	4.088176
40	6	0	-3.531221	-0.073490	3.845803

41	6	0	-3.792075	-1.782555	0.573826
42	6	0	-4.688944	-0.755188	0.120197
43	6	0	-4.881895	-0.876653	-1.290854
44	6	0	-4.118637	-1.998355	-1.736477
45	6	0	-3.455170	-2.547718	-0.595756
46	26	0	-5.516956	-2.614637	-0.335937
47	6	0	-6.024166	-4.612593	-0.102985
48	6	0	-6.369485	-3.878032	1.069377
49	6	0	-7.308365	-2.871583	0.695739
50	6	0	-7.563902	-2.975496	-0.713609
51	6	0	-6.751603	-4.055909	-1.197639
52	15	0	-8.900021	-2.124220	-1.635916
53	6	0	-8.762666	-0.384382	-0.976769
54	6	0	-8.115769	-1.904126	-3.314343
55	8	0	1.097826	0.430589	1.302679
56	6	0	1.884353	0.569186	2.469847
57	8	0	-0.049201	2.545539	-1.233390
58	6	0	-1.055508	2.029396	-1.716956
59	8	0	-2.034797	2.741942	-2.267187
60	6	0	-1.905798	4.184084	-2.227617
61	6	0	-2.025226	4.726876	-0.806213
62	8	0	-2.706834	3.883127	0.001753
63	6	0	-3.091456	4.412875	1.264410
64	6	0	-3.021889	4.754061	-3.098307
65	6	0	9.487814	-3.438303	-1.879909
66	8	0	-1.602808	5.808964	-0.475230
67	1	0	9.114565	-0.297861	2.816804
68	1	0	5.831047	2.151722	1.538513
69	1	0	-4.073293	-2.380237	-2.748310
70	1	0	-6.687088	-4.385025	-2.226631
71	1	0	-5.304699	-5.418885	-0.163425
72	1	0	-8.032525	-2.875553	-3.812470
73	1	0	-7.741223	0.005995	-1.023757
74	1	0	-3.114037	1.846202	4.886117
75	1	0	-0.252324	0.202600	-3.641958
76	1	0	-2.002467	0.505003	-3.810951
77	1	0	9.375377	-4.263065	-2.593245
78	1	0	-2.818568	-3.423632	-0.604820
79	1	0	6.119160	-3.762920	-0.109043
80	1	0	7.727161	-4.432376	0.262998
81	1	0	3.910555	5.749918	-0.960871
82	1	0	6.051554	0.360891	3.539533
83	1	0	8.968207	1.605538	0.905524
84	1	0	8.220035	0.414424	-1.394671
85	1	0	8.490704	-2.659524	1.694114
86	1	0	5.334580	-2.060478	2.565269

87	1	0	4.669549	-1.732944	-0.031772
88	1	0	3.487647	-2.308344	-4.185953
89	1	0	5.889949	1.320576	-1.784612
90	1	0	10.136211	-2.684012	-2.337638
91	1	0	9.975799	-3.816185	-0.973645
92	1	0	6.873822	-4.924713	-1.216311
93	1	0	1.335156	-1.494776	-2.652848
94	1	0	5.522384	-0.667446	-3.315654
95	1	0	1.658418	4.327569	-0.264538
96	1	0	5.733835	3.768594	-1.568809
97	1	0	-2.295336	0.347496	-1.358205
98	1	0	-1.177031	1.997289	2.915230
99	1	0	-4.339598	-0.569491	4.363235
100	1	0	1.013069	-3.541996	1.302103
101	1	0	-0.217805	-5.628846	2.612015
102	1	0	-2.749401	-4.651434	3.106689
103	1	0	-4.112969	-2.557633	2.814498
104	1	0	-5.159385	-0.012587	0.753157
105	1	0	-5.505053	-0.242179	-1.907279
106	1	0	-5.948934	-4.014454	2.056522
107	1	0	-7.745076	-2.140639	1.363811
108	1	0	-8.771633	-1.275962	-3.927271
109	1	0	-7.122211	-1.447778	-3.260562
110	1	0	-9.427410	0.264310	-1.557987
111	1	0	-9.103985	-0.356460	0.063169
112	1	0	-1.421580	-1.060549	-3.198254
113	1	0	-0.918784	4.464802	-2.601117
114	1	0	-2.928090	5.842669	-3.140160
115	1	0	-2.944136	4.349917	-4.111719
116	1	0	-4.000021	4.504639	-2.678601
117	1	0	-3.260010	3.548820	1.911588
118	1	0	-2.288843	5.030027	1.680252
119	6	0	-4.369235	5.243698	1.094811
120	1	0	1.245387	0.602683	3.365752
121	1	0	2.595007	-0.259849	2.582548
122	1	0	2.467237	1.498694	2.433632
123	8	0	-4.870709	5.455270	0.020103
124	8	0	-4.933647	5.742129	2.217224
125	6	0	-4.346991	5.521527	3.505590
126	1	0	-4.998398	6.036627	4.212305
127	1	0	-4.316082	4.455947	3.759061
128	1	0	-3.340841	5.949811	3.569430

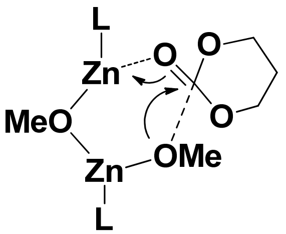

TSI-II^{dimer}- propagation-TMC

Standard orientation:

Center Number	Atomic Number	Atomic Type	Coordinates (Angstroms) X	Y	Z
1	8	0	1.253637	1.331692	2.934404
2	6	0	1.207341	1.705995	1.638910
3	8	0	2.147417	2.608053	1.242529
4	6	0	3.235435	2.799540	2.178616
5	6	0	2.697173	3.250379	3.533455
6	6	0	1.502912	2.352031	3.923527
7	8	0	0.815488	0.815914	0.834757
8	30	0	0.981215	0.649293	-1.179172
9	7	0	2.382937	1.643621	-2.321120
10	6	0	2.338840	2.850640	-2.910710
11	6	0	3.359951	2.957484	-3.858848
12	6	0	4.010909	1.727875	-3.804508
13	7	0	3.412412	0.951196	-2.882414
14	5	0	3.858097	-0.487420	-2.402466
15	6	0	4.403167	-0.438589	-0.889509
16	6	0	5.635089	0.175213	-0.477082
17	6	0	5.792715	0.033903	0.935508
18	6	0	4.661546	-0.688664	1.425862
19	6	0	3.815833	-0.973562	0.308986
20	26	0	5.682332	-1.841493	0.037370
21	6	0	7.294474	-2.782309	-0.891821
22	6	0	6.062263	-3.308893	-1.379884
23	6	0	5.354206	-3.866129	-0.274382
24	6	0	6.154382	-3.683823	0.892891
25	6	0	7.371042	-3.017336	0.522762
26	15	0	8.855656	-2.814174	1.577563
27	6	0	9.566179	-1.220121	0.917176
28	30	0	-1.601250	1.698351	0.445356
29	8	0	-0.349446	2.827734	1.619552
30	6	0	-0.219870	4.205883	1.526829
31	6	0	0.089483	4.698987	0.115711
32	8	0	-0.157143	4.083118	-0.905024
33	7	0	-2.436265	0.361827	1.821797
34	6	0	-1.846127	-0.328374	2.811303

35	6	0	-2.812834	-0.901876	3.643147
36	6	0	-4.025266	-0.500037	3.092378
37	7	0	-3.782605	0.260931	2.006665
38	5	0	-4.835806	0.793031	0.979407
39	6	0	-4.799863	-0.083075	-0.375177
40	6	0	-4.171053	-1.359024	-0.564852
41	6	0	-4.388547	-1.810031	-1.903489
42	6	0	-5.165835	-0.815222	-2.570200
43	6	0	-5.419941	0.233219	-1.631223
44	26	0	-6.213744	-1.561559	-0.952536
45	6	0	-7.090757	-2.920958	0.371670
46	6	0	-7.253071	-3.364061	-0.986312
47	6	0	-7.978087	-2.369184	-1.706007
48	6	0	-8.272432	-1.299819	-0.806010
49	6	0	-7.723831	-1.633919	0.465682
50	15	0	-6.309766	-3.755650	1.802365
51	6	0	-5.084282	-4.885819	0.963210
52	7	0	-3.388017	2.789220	0.165790
53	7	0	-4.562028	2.313921	0.663713
54	6	0	-5.484472	3.295939	0.636968
55	6	0	-4.908754	4.448901	0.112260
56	6	0	-3.590270	4.075701	-0.164202
57	8	0	-0.947378	0.992903	-1.301160
58	6	0	-1.641325	1.245022	-2.513032
59	6	0	-7.633072	-5.023188	2.195089
60	7	0	1.439694	-1.228828	-1.937582
61	7	0	2.633839	-1.456156	-2.551975
62	6	0	2.576129	-2.634306	-3.201546
63	6	0	1.316645	-3.196926	-3.015407
64	6	0	0.638105	-2.269395	-2.219741
65	6	0	8.106976	-2.203147	3.174000
66	8	0	0.621513	5.933456	0.136489
67	6	0	0.882666	6.538506	-1.141810
68	1	0	-5.530519	-0.864982	-3.588504
69	1	0	-1.550246	2.298217	-2.817108
70	1	0	5.879914	-3.985804	1.895492
71	1	0	-8.222653	-2.399185	-2.760322
72	1	0	-6.012655	1.118197	-1.830295
73	1	0	4.493877	-1.003053	2.448407
74	1	0	4.365948	-4.305918	-0.305680
75	1	0	7.535405	-3.010137	3.643992
76	1	0	8.817839	-0.424944	0.837610
77	1	0	3.590505	3.796829	-4.498100
78	1	0	-7.270633	-5.695378	2.981544
79	1	0	2.898641	-1.545314	0.356525
80	1	0	-4.286027	-4.290431	0.511109

81	1	0	-5.538965	-5.514489	0.188391
82	1	0	-5.377314	5.409441	-0.043112
83	1	0	-8.779539	-0.377393	-1.057703
84	1	0	-7.733021	-1.005598	1.346848
85	1	0	-6.864033	-4.283565	-1.403653
86	1	0	-4.054377	-2.748630	-2.327496
87	1	0	-3.630206	-1.898585	0.202322
88	1	0	-2.659724	-1.518562	4.516299
89	1	0	-5.902971	0.781016	1.545360
90	1	0	-8.524623	-4.512382	2.573448
91	1	0	-7.916063	-5.619719	1.319789
92	1	0	-4.636116	-5.540204	1.719222
93	1	0	-0.767276	-0.357028	2.873199
94	1	0	-5.040866	-0.722438	3.384283
95	1	0	-2.780237	4.652133	-0.589294
96	1	0	-6.488085	3.102789	0.986827
97	1	0	0.565231	4.595735	2.187507
98	1	0	1.571101	3.555321	-2.620114
99	1	0	4.859721	1.352450	-4.357358
100	1	0	-0.377267	-2.282179	-1.846027
101	1	0	0.948282	-4.133240	-3.407280
102	1	0	3.433058	-2.990586	-3.754443
103	1	0	4.691325	-0.844683	-3.201093
104	1	0	6.348628	0.647542	-1.141536
105	1	0	6.632482	0.382630	1.522407
106	1	0	5.707860	-3.244129	-2.399547
107	1	0	8.041435	-2.275763	-1.489078
108	1	0	8.916912	-1.931100	3.860086
109	1	0	7.446972	-1.341934	3.029054
110	1	0	10.374333	-0.895211	1.581882
111	1	0	10.003804	-1.393116	-0.071379
112	1	0	-1.238250	0.617876	-3.321385
113	1	0	-2.704712	1.004502	-2.402303
114	1	0	-1.156266	4.703046	1.834079
115	1	0	1.201617	7.557829	-0.923217
116	1	0	1.676277	5.996571	-1.663303
117	1	0	-0.019098	6.540733	-1.759453
118	1	0	3.875575	3.551502	1.715579
119	1	0	3.792688	1.860861	2.253579
120	1	0	3.498084	3.189278	4.278660
121	1	0	2.385073	4.298335	3.478860
122	1	0	1.691001	1.789027	4.838762
123	1	0	0.581006	2.925500	4.045257

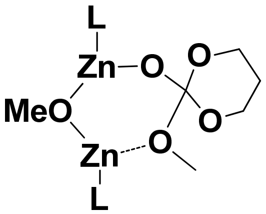

II^{dimer}- propagation-TMC

Standard orientation:

Center Number	Atomic Number	Atomic Type	Coordinates (Angstroms) X	Y	Z
1	26	0	-6.273438	-1.558100	-0.970202
2	6	0	-5.444836	0.232931	-1.617881
3	6	0	-4.822430	-0.125433	-0.374240
4	6	0	-4.224456	-1.411546	-0.597643
5	6	0	-4.463449	-1.827270	-1.944007
6	6	0	-7.784544	-1.616643	0.448021
7	6	0	-7.170300	-2.912117	0.345728
8	6	0	-7.339172	-3.344016	-1.015092
9	6	0	-8.049388	-2.333912	-1.728362
10	6	0	-8.328027	-1.266179	-0.821501
11	15	0	-6.399009	-3.765143	1.770970
12	5	0	-4.822214	0.724564	0.996767
13	7	0	-4.505766	2.242177	0.700216
14	7	0	-3.328232	2.681900	0.177977
15	6	0	-3.489657	3.979854	-0.129324
16	6	0	-4.785534	4.396755	0.189233
17	6	0	-5.390752	3.257885	0.712268
18	1	0	-1.563969	2.136587	-3.118379
19	7	0	-2.422375	0.222900	1.768737
20	6	0	-2.766392	-1.012286	3.613500
21	6	0	-1.813694	-0.467819	2.747103
22	6	0	-3.987800	-0.591680	3.095785
23	6	0	5.267116	-3.902542	-0.387817
24	6	0	5.996958	-3.313649	-1.462477
25	6	0	6.075987	-3.821737	0.784819
26	6	0	7.252177	-2.870301	-0.950299
27	1	0	8.019783	-2.363402	-1.520442
28	15	0	8.810873	-3.100021	1.511271
29	6	0	5.873989	-0.101513	1.043516
30	6	0	5.729430	0.129535	-0.358675
31	6	0	4.473278	-0.402230	-0.809420
32	6	0	3.857174	-0.980862	0.354261
33	6	0	4.710827	-0.801317	1.487876
34	30	0	-1.603684	1.485588	0.335015

35	30	0	0.998448	0.781488	-1.159327
36	7	0	1.507493	-1.078410	-1.940064
37	7	0	2.706717	-1.278951	-2.553870
38	6	0	2.646173	-2.413239	-3.277324
39	6	0	1.379893	-2.974508	-3.140931
40	6	0	0.700198	-2.091522	-2.297203
41	7	0	2.491823	1.806077	-2.146025
42	7	0	3.528928	1.134669	-2.718799
43	6	0	4.185776	1.968893	-3.546168
44	6	0	3.566587	3.215928	-3.525362
45	6	0	2.502515	3.058776	-2.633380
46	8	0	0.499210	1.056068	0.774498
47	6	0	0.850597	2.037131	1.596729
48	8	0	1.134006	1.497179	2.848981
49	8	0	1.879654	2.876929	1.140644
50	6	0	1.742403	2.380094	3.800400
51	6	0	3.068526	2.959248	3.251109
52	6	0	3.156083	2.625470	1.761066
53	8	0	-0.367770	2.898082	1.687225
54	6	0	-0.242105	4.303929	1.710848
55	6	0	0.087187	4.891340	0.343107
56	8	0	0.603087	6.122644	0.478067
57	6	0	0.890308	6.827629	-0.743662
58	6	0	-5.223121	-0.800235	-2.581551
59	7	0	-3.763729	0.152712	1.995508
60	6	0	7.319439	-3.187656	0.448520
61	6	0	-5.201348	-4.918276	0.923234
62	6	0	-7.746023	-5.005747	2.168808
63	8	0	-0.942909	1.005182	-1.478067
64	6	0	-1.639036	1.119443	-2.704454
65	5	0	3.941402	-0.341435	-2.326428
66	26	0	5.684599	-1.914718	0.035652
67	6	0	9.582189	-1.501293	0.937099
68	6	0	8.085439	-2.550302	3.140169
69	8	0	-0.138785	4.353748	-0.722227
70	1	0	-5.597617	-0.819417	-3.597299
71	1	0	5.789311	-4.165907	1.770223
72	1	0	-8.294067	-2.353435	-2.782888
73	1	0	-6.017750	1.135861	-1.792467
74	1	0	4.522453	-1.162738	2.490822
75	1	0	4.260476	-4.296272	-0.440231
76	1	0	7.482985	-3.359650	3.565445
77	1	0	8.866778	-0.673152	0.907025
78	1	0	3.844781	4.098375	-4.082329
79	1	0	-7.393950	-5.685244	2.953653
80	1	0	2.914461	-1.512085	0.364992

81	1	0	-4.391359	-4.339271	0.470597
82	1	0	-5.672172	-5.534109	0.147778
83	1	0	-5.222367	5.375896	0.059358
84	1	0	-8.821528	-0.334789	-1.067142
85	1	0	-7.785813	-0.994793	1.333813
86	1	0	-6.962904	-4.265943	-1.438739
87	1	0	-4.154599	-2.763700	-2.391256
88	1	0	-3.692765	-1.983043	0.152700
89	1	0	-2.598635	-1.621255	4.489325
90	1	0	-5.880537	0.732301	1.578279
91	1	0	-8.625423	-4.477071	2.551031
92	1	0	-8.044754	-5.596369	1.294714
93	1	0	-4.765221	-5.585075	1.675381
94	1	0	-0.733780	-0.509815	2.777078
95	1	0	-4.998336	-0.789054	3.421427
96	1	0	-2.667902	4.529728	-0.566643
97	1	0	-6.391601	3.096714	1.085568
98	1	0	0.501918	4.645776	2.432675
99	1	0	1.740241	3.758802	-2.317331
100	1	0	5.050433	1.618671	-4.090961
101	1	0	-0.320580	-2.116879	-1.939519
102	1	0	1.007982	-3.880905	-3.594917
103	1	0	3.505933	-2.742485	-3.842348
104	1	0	4.780629	-0.660134	-3.135330
105	1	0	6.466371	0.607256	-0.993049
106	1	0	6.723868	0.177954	1.652110
107	1	0	5.642481	-3.171222	-2.473939
108	1	0	8.905009	-2.348561	3.838974
109	1	0	7.459454	-1.657372	3.045064
110	1	0	10.405764	-1.247308	1.613664
111	1	0	10.008403	-1.636067	-0.062345
112	1	0	-1.228245	0.419337	-3.446733
113	1	0	-2.700578	0.879849	-2.570835
114	1	0	-1.220355	4.690670	2.019835
115	1	0	1.196134	7.828039	-0.437762
116	1	0	1.699668	6.330234	-1.284432
117	1	0	0.003036	6.871064	-1.379999
118	1	0	3.864740	3.265477	1.232455
119	1	0	3.442503	1.583082	1.599143
120	1	0	3.934877	2.535369	3.771365
121	1	0	3.098074	4.045080	3.397361
122	1	0	1.900401	1.760647	4.685266
123	1	0	1.035729	3.172338	4.075231

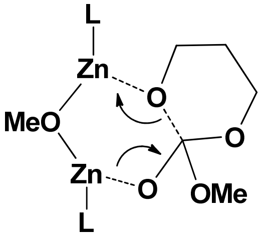

TSII-III^{dimer}- propagation-TMC

Standard orientation:

Center Number	Atomic Number	Atomic Type	Coordinates (Angstroms) X	Y	Z
1	6	0	3.646521	-0.717629	3.321432
2	7	0	3.519417	-0.906842	1.997450
3	7	0	4.722300	-1.349157	1.536641
4	6	0	5.592423	-1.414932	2.562155
5	6	0	4.948036	-1.017502	3.730601
6	30	0	1.817377	-1.022299	0.814980
7	8	0	0.506685	-1.814613	2.140441
8	6	0	-0.672493	-2.166208	1.870346
9	8	0	-0.899369	-2.602224	0.600530
10	6	0	-2.231707	-2.970360	0.263062
11	6	0	-2.315717	-3.081239	-1.248188
12	8	0	-3.598457	-3.255276	-1.610783
13	6	0	-3.836293	-3.375860	-3.027074
14	5	0	5.028637	-1.576356	0.014066
15	6	0	5.042182	-0.165467	-0.768076
16	6	0	4.912932	0.043932	-2.182454
17	6	0	5.010291	1.439767	-2.473692
18	6	0	5.214836	2.124660	-1.237511
19	6	0	5.236712	1.139785	-0.200080
20	26	0	6.758364	0.789609	-1.577563
21	6	0	8.612772	0.828244	-0.621064
22	6	0	8.551394	1.840227	-1.640599
23	6	0	8.313974	1.207644	-2.896543
24	6	0	8.227256	-0.199916	-2.672618
25	6	0	8.403451	-0.432951	-1.278178
26	15	0	8.918669	0.976291	1.178780
27	6	0	10.781940	1.173610	1.175521
28	8	0	0.831060	0.576838	0.181415
29	6	0	1.438511	1.451268	-0.771407
30	30	0	-1.134784	0.766008	0.442201
31	8	0	-1.657582	-0.576507	1.793178
32	6	0	-2.379546	-0.329719	2.991434
33	6	0	-1.560655	-0.804821	4.197634

34	6	0	-1.328318	-2.321736	4.137050
35	8	0	-1.429166	-2.822071	2.780826
36	7	0	-2.048596	0.801356	-1.447230
37	7	0	-2.998889	1.741009	-1.717321
38	6	0	-3.218485	1.779458	-3.045261
39	6	0	-2.394945	0.847411	-3.669808
40	6	0	-1.673898	0.265818	-2.621873
41	5	0	-3.761785	2.531093	-0.597370
42	7	0	-2.710589	3.319600	0.275106
43	7	0	-1.686605	2.718305	0.940554
44	6	0	-1.048864	3.674909	1.637074
45	6	0	-1.667056	4.912162	1.439365
46	6	0	-2.713972	4.634667	0.565081
47	6	0	-4.637076	1.533460	0.316769
48	6	0	-4.910053	0.139949	0.098394
49	6	0	-5.744065	-0.355397	1.148829
50	6	0	-6.015621	0.731052	2.033410
51	6	0	-5.342495	1.881617	1.518903
52	26	0	-6.720384	1.173474	0.132682
53	6	0	-8.438013	0.463806	-0.871425
54	6	0	-7.591367	1.206624	-1.762087
55	6	0	-7.442392	2.532919	-1.258362
56	6	0	-8.188024	2.627474	-0.046974
57	6	0	-8.794408	1.357903	0.194400
58	15	0	-9.174344	-1.179458	-1.212473
59	6	0	-7.681987	-2.161260	-1.757199
60	6	0	-9.398162	-1.856348	0.511401
61	7	0	2.615455	-2.314480	-0.590108
62	7	0	3.959046	-2.549779	-0.604823
63	6	0	4.204177	-3.630578	-1.370493
64	6	0	3.000763	-4.121273	-1.867285
65	6	0	2.030174	-3.263480	-1.341654
66	6	0	8.449907	2.754504	1.496221
67	8	0	-1.386676	-3.031070	-2.024094
68	1	0	-5.383390	2.873810	1.952424
69	1	0	8.961111	3.459487	0.830134
70	1	0	5.356271	3.190927	-1.113564
71	1	0	2.852215	-4.977192	-2.508723
72	1	0	-0.922741	-0.511539	-2.648715
73	1	0	-3.461031	5.282131	0.129482
74	1	0	-1.393818	5.868200	1.860375
75	1	0	7.369388	2.877291	1.378391
76	1	0	5.219617	-3.971554	-1.511166
77	1	0	0.953883	-3.275924	-1.458363
78	1	0	4.786417	-0.739988	-2.919535
79	1	0	4.976901	1.891933	-3.456874

80	1	0	8.016393	-0.951777	-3.422032
81	1	0	8.340151	-1.392396	-0.781698
82	1	0	8.634293	2.907250	-1.482023
83	1	0	8.179432	1.710425	-3.845853
84	1	0	5.396904	1.340063	0.852192
85	1	0	5.364177	-0.960379	4.725291
86	1	0	6.073251	-2.180160	-0.030730
87	1	0	11.125857	1.393810	2.192826
88	1	0	11.246040	0.233468	0.859839
89	1	0	11.118634	1.972971	0.505000
90	1	0	8.711407	3.006063	2.530019
91	1	0	2.794559	-0.391595	3.902939
92	1	0	6.613777	-1.719614	2.387593
93	1	0	0.993003	2.453701	-0.705057
94	1	0	2.512512	1.547372	-0.582317
95	1	0	1.303450	1.085096	-1.798443
96	1	0	-2.319703	0.635567	-4.726073
97	1	0	-3.945740	2.466616	-3.452748
98	1	0	-0.180132	3.423350	2.231414
99	1	0	-4.403915	3.385109	-1.161008
100	1	0	-4.558929	-0.442093	-0.743930
101	1	0	-6.117455	-1.366329	1.245459
102	1	0	-6.646226	0.698083	2.912947
103	1	0	-9.406762	1.105925	1.050415
104	1	0	-6.819432	3.307998	-1.683819
105	1	0	-8.243797	3.491225	0.602821
106	1	0	-7.128884	0.820100	-2.661217
107	1	0	-9.694503	-2.908547	0.437483
108	1	0	-8.491203	-1.777288	1.119730
109	1	0	-10.210276	-1.321577	1.014642
110	1	0	-7.985366	-3.204080	-1.904925
111	1	0	-7.329035	-1.779198	-2.721008
112	1	0	-6.857998	-2.118970	-1.037804
113	1	0	-2.497612	-3.933081	0.714294
114	1	0	-2.934204	-2.211754	0.617792
115	1	0	-2.114832	-2.869838	4.660780
116	1	0	-0.357875	-2.609261	4.551999
117	1	0	-2.081196	-0.553944	5.130262
118	1	0	-0.603124	-0.274389	4.198129
119	1	0	-3.348342	-0.849155	2.959197
120	1	0	-2.594124	0.742482	3.078697
121	1	0	-4.909781	-3.534146	-3.126790
122	1	0	-3.532402	-2.458392	-3.537054
123	1	0	-3.278775	-4.221749	-3.436761

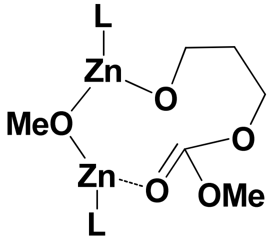

III^{dimer}- propagation-TMC

Standard orientation:

Center Number	Atomic Number	Atomic Type	Coordinates (Angstroms) X	Y	Z
1	6	0	-3.646295	1.197053	3.154714
2	7	0	-3.530978	1.224459	1.816133
3	7	0	-4.762000	1.518605	1.311838
4	6	0	-5.635471	1.653041	2.327458
5	6	0	-4.965882	1.454823	3.532126
6	30	0	-1.854757	1.282929	0.598191
7	8	0	-0.702431	2.496679	1.835222
8	6	0	0.380177	3.008845	1.524368
9	8	0	0.661617	3.234297	0.245556
10	6	0	2.031622	3.465891	-0.111626
11	5	0	-5.086267	1.515379	-0.225348
12	6	0	-4.998625	0.014653	-0.808237
13	6	0	-4.870764	-0.376329	-2.184321
14	6	0	-4.849504	-1.802958	-2.278099
15	6	0	-4.977583	-2.323639	-0.955048
16	6	0	-5.071362	-1.211230	-0.062957
17	26	0	-6.636236	-1.180655	-1.438664
18	6	0	-8.474133	-1.198794	-0.447144
19	6	0	-8.336623	-2.366290	-1.274948
20	6	0	-8.162766	-1.947468	-2.627216
21	6	0	-8.191798	-0.519970	-2.654793
22	6	0	-8.375373	-0.060178	-1.318860
23	15	0	-8.756932	-1.051085	1.356319
24	6	0	-10.595351	-1.404808	1.425909
25	8	0	-0.721963	-0.263753	0.181920
26	6	0	-1.336498	-1.329807	-0.548971
27	30	0	1.254221	-0.486104	0.562145
28	8	0	1.837808	1.044202	1.578586
29	6	0	2.389547	1.007900	2.865170
30	6	0	1.404840	1.579215	3.906048
31	6	0	1.165404	3.084828	3.767095
32	8	0	1.222932	3.548861	2.380622

33	7	0	2.108806	-0.876912	-1.327463
34	7	0	2.967625	-1.922400	-1.485008
35	6	0	3.192739	-2.117428	-2.798424
36	6	0	2.466666	-1.180948	-3.528069
37	6	0	1.796526	-0.430122	-2.556002
38	5	0	3.640656	-2.669601	-0.280454
39	7	0	2.510282	-3.221720	0.672211
40	7	0	1.591166	-2.426827	1.284654
41	6	0	0.863659	-3.213794	2.094738
42	6	0	1.316386	-4.534169	2.025581
43	6	0	2.360848	-4.487740	1.107086
44	6	0	4.642463	-1.709425	0.533503
45	6	0	4.933635	-0.318244	0.328489
46	6	0	5.879795	0.120734	1.305210
47	6	0	6.202748	-0.998462	2.129152
48	6	0	5.449908	-2.113649	1.651144
49	26	0	6.704448	-1.407900	0.156219
50	6	0	8.448822	-0.756816	-0.838657
51	6	0	7.473745	-1.244038	-1.773352
52	6	0	7.182585	-2.607248	-1.466752
53	6	0	7.966260	-2.978845	-0.335497
54	6	0	8.739493	-1.843817	0.053632
55	15	0	9.370613	0.822202	-0.962436
56	6	0	8.004509	2.017613	-1.398894
57	6	0	9.620341	1.253003	0.835758
58	7	0	-2.729959	2.317954	-0.958553
59	7	0	-4.086008	2.465589	-0.983737
60	6	0	-4.404830	3.404454	-1.895023
61	6	0	-3.238748	3.890246	-2.479009
62	6	0	-2.211779	3.179685	-1.851617
63	6	0	-8.129426	-2.695829	1.975765
64	1	0	5.502161	-3.119297	2.050808
65	1	0	-8.584367	-3.551400	1.462914
66	1	0	-5.025912	-3.370342	-0.682891
67	1	0	-3.150310	4.649072	-3.242188
68	1	0	1.117192	0.403379	-2.673414
69	1	0	3.009627	-5.267172	0.734766
70	1	0	0.940916	-5.396371	2.556325
71	1	0	-7.043979	-2.746223	1.850510
72	1	0	-5.440929	3.656905	-2.068092
73	1	0	-1.139473	3.237183	-1.988917
74	1	0	-4.822327	0.305434	-3.024863
75	1	0	-4.788268	-2.383330	-3.190052
76	1	0	-8.048412	0.102660	-3.528328
77	1	0	-8.390418	0.973548	-0.998652
78	1	0	-8.327547	-3.391415	-0.929479

79	1	0	-7.992895	-2.597298	-3.476249
80	1	0	-5.196985	-1.276545	1.010474
81	1	0	-5.378449	1.495044	4.529095
82	1	0	-6.168952	2.036685	-0.339951
83	1	0	-10.915596	-1.462629	2.472695
84	1	0	-11.141233	-0.582386	0.952080
85	1	0	-10.861987	-2.340017	0.919788
86	1	0	-8.353701	-2.775639	3.045220
87	1	0	-2.775627	1.005888	3.767778
88	1	0	-6.676343	1.856959	2.124133
89	1	0	-0.613064	-1.788015	-1.235572
90	1	0	-1.709749	-2.109475	0.128628
91	1	0	-2.181098	-0.967321	-1.145771
92	1	0	2.418211	-1.074413	-4.601720
93	1	0	3.852288	-2.909247	-3.122378
94	1	0	0.052526	-2.791864	2.673774
95	1	0	4.172996	-3.652960	-0.739027
96	1	0	4.516604	0.297113	-0.457730
97	1	0	6.290417	1.117815	1.394120
98	1	0	6.916019	-1.009121	2.943645
99	1	0	9.419081	-1.805480	0.895250
100	1	0	6.452370	-3.230971	-1.964142
101	1	0	7.945020	-3.936340	0.168622
102	1	0	7.021339	-0.668646	-2.570587
103	1	0	10.040503	2.263004	0.899690
104	1	0	8.692645	1.206677	1.415220
105	1	0	10.348443	0.566235	1.279793
106	1	0	8.404565	3.037143	-1.361267
107	1	0	7.671258	1.831708	-2.425435
108	1	0	7.143352	1.937272	-0.728164
109	6	0	2.151783	3.251640	-1.608667
110	1	0	2.337236	4.482379	0.153892
111	1	0	2.647431	2.733688	0.419401
112	1	0	1.968337	3.660640	4.231277
113	1	0	0.208062	3.398591	4.192414
114	1	0	1.784342	1.408081	4.923229
115	1	0	0.457513	1.037230	3.815922
116	1	0	3.327061	1.592550	2.901105
117	1	0	2.660770	-0.014142	3.176788
118	8	0	1.252499	2.954061	-2.364224
119	8	0	3.430944	3.424892	-1.972075
120	6	0	3.726238	3.194919	-3.364654
121	1	0	4.785222	3.425860	-3.476228
122	1	0	3.530563	2.150524	-3.620123
123	1	0	3.117614	3.846709	-3.995939

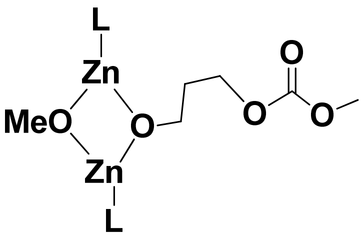

IV^{dimer}- propagation-TMC

Standard orientation:

Center Number	Atomic Number	Atomic Type	Coordinates (Angstroms) X	Y	Z
1	6	0	6.959063	-1.320097	2.119882
2	6	0	7.593588	-0.461461	1.172747
3	6	0	7.973254	-1.237265	0.025761
4	6	0	7.547313	-2.584612	0.283036
5	6	0	6.931392	-2.635131	1.568537
6	26	0	5.887562	-1.326096	0.344147
7	6	0	4.473508	-1.931648	-1.063635
8	6	0	3.899750	-2.049365	0.248252
9	6	0	3.953786	-0.728056	0.814326
10	6	0	4.535656	0.178168	-0.125807
11	6	0	4.851113	-0.573547	-1.298597
12	5	0	3.340804	-3.397274	0.927145
13	7	0	2.400220	-4.161460	-0.082006
14	7	0	1.263546	-3.608870	-0.587527
15	6	0	0.756966	-4.481329	-1.475458
16	6	0	1.575327	-5.610141	-1.564843
17	6	0	2.603396	-5.359984	-0.659867
18	15	0	9.069377	-0.689565	-1.337616
19	6	0	8.440163	1.038550	-1.649342
20	30	0	0.461048	-1.942111	0.361387
21	8	0	-1.533390	-1.887144	0.584717
22	6	0	-2.405762	-2.868921	1.107865
23	30	0	-1.875784	-0.226946	-0.433590
24	8	0	0.136162	-0.219342	-0.577443
25	6	0	0.969063	0.670393	-1.293870
26	6	0	1.197973	1.973309	-0.517126
27	6	0	2.013442	2.983130	-1.316886
28	8	0	2.131919	4.239692	-0.604216
29	6	0	3.190046	4.373028	0.196295
30	8	0	4.083246	3.571411	0.374863
31	7	0	-2.829695	1.438060	0.351333
32	7	0	-3.979087	1.884763	-0.228872
33	6	0	-4.332469	3.056246	0.332837

34	6	0	-3.391267	3.398743	1.299937
35	6	0	-2.466699	2.352318	1.268110
36	5	0	-4.803341	1.032983	-1.262784
37	7	0	-3.874375	0.647975	-2.471898
38	7	0	-2.732197	-0.075714	-2.320288
39	6	0	-2.233666	-0.296917	-3.548448
40	6	0	-3.064259	0.273664	-4.516774
41	6	0	-4.089890	0.863911	-3.783384
42	6	0	-5.382181	-0.291552	-0.548912
43	6	0	-5.683020	-0.453890	0.847021
44	6	0	-6.218978	-1.759551	1.075005
45	6	0	-6.262656	-2.430954	-0.184367
46	6	0	-5.755515	-1.531020	-1.171992
47	26	0	-7.434596	-0.725455	-0.246548
48	6	0	-8.827671	0.742197	0.262986
49	6	0	-8.562738	0.757557	-1.149487
50	6	0	-8.914385	-0.510432	-1.695876
51	6	0	-9.390239	-1.331093	-0.628780
52	6	0	-9.338819	-0.565403	0.573229
53	15	0	-8.541449	2.192455	1.345422
54	6	0	-8.429592	1.395048	3.028867
55	7	0	1.384878	-2.302874	2.184564
56	7	0	2.513725	-3.063026	2.220695
57	6	0	2.791424	-3.379640	3.499269
58	6	0	1.822595	-2.820338	4.328015
59	6	0	0.957888	-2.160500	3.450360
60	6	0	8.325455	-1.577617	-2.800591
61	6	0	-10.287992	2.859475	1.463003
62	8	0	3.100120	5.577054	0.796813
63	6	0	4.190844	5.895223	1.658334
64	1	0	7.658740	-3.422275	-0.393241
65	1	0	-9.695385	-2.366690	-0.709620
66	1	0	-5.690561	-1.739997	-2.232983
67	1	0	4.712407	1.233886	0.033502
68	1	0	7.741910	0.603947	1.291558
69	1	0	6.533837	-1.014684	3.067142
70	1	0	8.724874	1.690592	-0.817241
71	1	0	7.246675	-1.415265	-2.889526
72	1	0	1.442366	-6.481073	-2.189107
73	1	0	-10.312576	3.688697	2.179455
74	1	0	3.638102	-0.466777	1.816713
75	1	0	-7.508948	0.808375	3.099936
76	1	0	-9.282499	0.744523	3.255276
77	1	0	-2.940404	0.264563	-5.589389
78	1	0	-6.645164	-3.427738	-0.363967
79	1	0	-8.795708	-0.814245	-2.727906

80	1	0	-8.118013	1.582333	-1.690658
81	1	0	-9.608830	-0.924402	1.557515
82	1	0	-6.561798	-2.155740	2.022488
83	1	0	-5.547749	0.310584	1.602033
84	1	0	-3.380900	4.276813	1.928044
85	1	0	-5.638437	1.776266	-1.715603
86	1	0	-10.593635	3.249435	0.486626
87	1	0	-11.009491	2.096136	1.777038
88	1	0	-8.388731	2.185110	3.786856
89	1	0	-1.562648	2.211108	1.845597
90	1	0	-5.238585	3.554140	0.019999
91	1	0	-1.308101	-0.845072	-3.665279
92	1	0	-4.958865	1.420107	-4.103673
93	1	0	-0.165599	-4.254002	-1.993326
94	1	0	3.466657	-5.950862	-0.390055
95	1	0	0.054907	-1.600880	3.655408
96	1	0	1.755568	-2.887634	5.403596
97	1	0	3.658881	-3.980084	3.731792
98	1	0	4.181847	-4.199112	1.255340
99	1	0	4.611802	-2.749854	-1.760167
100	1	0	5.309393	-0.185832	-2.198888
101	1	0	6.470309	-3.506717	2.014070
102	1	0	8.921145	1.428490	-2.553420
103	1	0	7.352837	1.074951	-1.769185
104	1	0	8.823175	-1.228910	-3.712454
105	1	0	8.514421	-2.652589	-2.713694
106	1	0	-2.616509	-3.659008	0.371020
107	1	0	-1.962318	-3.344945	1.994895
108	1	0	-3.363836	-2.424247	1.403868
109	1	0	1.935054	0.191696	-1.501395
110	1	0	0.505687	0.903322	-2.267447
111	1	0	0.226963	2.417436	-0.263565
112	1	0	1.718520	1.743302	0.419850
113	1	0	3.013818	2.605733	-1.540967
114	1	0	1.509588	3.252511	-2.249037
115	6	0	5.487145	6.270885	0.948996
116	1	0	3.856896	6.756333	2.242409
117	1	0	4.413264	5.067569	2.335944
118	8	0	6.517056	6.454032	1.558148
119	8	0	5.340472	6.404912	-0.376343
120	6	0	6.538915	6.752568	-1.093065
121	1	0	6.239671	6.831833	-2.138074
122	1	0	6.937207	7.703581	-0.730620
123	1	0	7.296091	5.974787	-0.965534

References

1. Foroozandeh, M.; Castañar, L.; Martins, L. G.; Sinnaeve, D.; Poggetto, G. D.; Tormena, C. F.; Adams, R. W.; Morris, G. A.; Nilsson, M., Ultrahigh-Resolution Diffusion-Ordered Spectroscopy. *Angew. Chem. Int. Ed.* **2016,** *55* (50), 15579-15582.

2. Frisch, M. J. T., G. W.; Schlegel, H. B.; Scuseria, G. E.; Robb, M. A.; Cheeseman, J. R.; Scalmani, G.; Barone, V.; Mennucci, B.; Petersson, G. A.; Nakatsuji, H.; Caricato, M.; Li, X.; Hratchian, H.P.; Izmaylov, A. F.; Bloino, J.; Zheng, G.; Sonnenberg, J. L.; Hada, M.; Ehara, M.; Toyota, K.; Fukuda, R.; Hasegawa, J.; Ishida, M.; Nakajima, T.; Honda, Y.; Kitao, O.; Nakai, H.; Vreven, T.; Montgomery, Jr., J. A.; Peralta, J. E.; Ogliaro, F.; Bearpark, M.; Heyd, J. J.; Brothers, E.; Kudin, K. N.; Staroverov, V. N.; Kobayashi, R.; Normand, J.; Raghavachari, K.; Rendell, A.; Burant, J. C.; Iyengar, S. S.; Tomasi, J.; Cossi, M.; Rega, N.; Millam, N. J.; Klene, M.; Knox, J. E.; Cross, J. B.; Bakken, V.; Adamo, C.; Jaramillo, J.; Gomperts, R.; Stratmann, R. E.; Yazyev, O.; Austin, A. J.; Cammi, R.; Pomelli, C.; Ochterski, J. W.; Martin, R. L.; Morokuma, K.; Zakrzewski, V. G.; Voth, G. A.; Salvador, P.; Dannenberg, J. J.; Dapprich, S.; Daniels, A. D.; Farkas, Ö.; Foresman, J. B.; Ortiz, J. V.; Cioslowski, J.; Fox, D. J. *Gaussian 09*, Gaussian Inc.: Wallingford, CT, 2010.
